# Genetic variation and microbiota in bumble bees cross-infected by different strains of *C*. *bombi*

**DOI:** 10.1371/journal.pone.0277041

**Published:** 2022-11-28

**Authors:** Seth M. Barribeau, Paul Schmid-Hempel, Jean-Claude Walser, Stefan Zoller, Martina Berchtold, Regula Schmid-Hempel, Niklaus Zemp

**Affiliations:** 1 Institute of Integrative Biology (IBZ), ETH Zürich, Zürich, Switzerland; 2 Genetic Diversity Centre, ETH Zürich, Zürich, Switzerland; Universidade Federal do Rio de Janeiro, BRAZIL

## Abstract

The bumblebee *Bombus terrestris* is commonly infected by a trypanosomatid gut parasite *Crithidia bombi*. This system shows a striking degree of genetic specificity where host genotypes are susceptible to different genotypes of parasite. To a degree, variation in host gene expression underlies these differences, however, the effects of standing genetic variation has not yet been explored. Here we report on an extensive experiment where workers of twenty colonies of *B*. *terrestris* were each infected by one of twenty strains of *C*. *bombi*. To elucidate the host’s genetic bases of susceptibility to infection (measured as infection intensity), we used a low-coverage (~2 x) genome-wide association study (GWAS), based on *angsd*, and a standard high-coverage (~15x) GWAS (with a reduced set from a 8 x 8 interaction matrix, selected from the full set of twenty). The results from the low-coverage approach remained ambiguous. The high-coverage approach suggested potentially relevant genetic variation in cell surface and adhesion processes. In particular, *mucin*, a surface mucoglycoprotein, potentially affecting parasite binding to the host gut epithelia, emerged as a candidate. Sequencing the gut microbial community of the same bees showed that the abundance of bacterial taxa, such as *Gilliamella*, *Snodgrassella*, or *Lactobacillus*, differed between ’susceptible’ and ’resistant’ microbiota, in line with earlier studies. Our study suggests that the constitutive microbiota and binding processes at the cell surface are candidates to affect infection intensity after the first response (captured by gene expression) has run its course. We also note that a low-coverage approach may not be powerful enough to analyse such complex traits. Furthermore, testing large interactions matrices (as with the full 20 x 20 combinations) for the effect of interaction terms on infection intensity seems to blur the specific host x parasite interaction effects, likely because the outcome of an infection is a highly non-linear process dominated by variation in individually different pathways of host defence (immune) responses.

## Introduction

Genetics and genetic variation affect infection outcomes in almost any study of host-parasite systems so far. For example, genetic sequences encode epitopes on the surface of parasites, which are recognized by receptor molecules that are encoded by host genes [1]. Extensive databases, such as the Immune Epitope Database (IEDB) [2], archive the enormous diversity of parasitic sequences; vice versa, host genetic variation in putative defence genes, e.g., receptor molecules, is likewise accessible in data repositories (e.g., the pattern-recognition data base, PRDDB [3] and various others, accessible via Immunological Databases and Tools). For non-model organisms, the knowledge is far less sophisticated than can be found in these databases. In fact, while homology offers powerful insights into shared function, to understand the genetic determinants of host-parasite interactions requires directly querying these systems with associative approaches, such as with genome-wide association studies (GWAS).

The genetic basis for host resistance or tolerance [4] can be very different. For example, the resistance of the water flea, *Daphnia magna*, against the bacterial pathogen *Pasteuria ramosa*, depends on a single genetic locus (a QTL) that explains over 50% of observed variation. By contrast, its resistance against the microsporidian, *Hamiltosporidium tvaerminensis*, follows a quantitative genetic pattern where multiple loci with weak effects add up to the overall variation (explaining a total of 38%; [5–7]). Hence, even when genes are identified that encode important elements of the host defence, their contribution to observed variation in resistance at the population level can be quite different. Furthermore, the actual molecular and physiological mechanisms underlying these interactions often are complex and diverse. Accordingly, the answers from associative studies will not always be simple and robust, but inevitably reflect the complexity of the processes themselves. Nevertheless, the identification of genes associated with a resistance phenotype, such as infection intensity, is helpful as a start for understanding the mechanistic bases of host-parasite interactions.

The system studied here, bumblebees (*Bombus terrestris*), infected by the common trypanosomatid parasite, *Crithidia bombi*, has been studied for some time, and considerable knowledge on the ecology, evolution, or genetics has been accumulated. *B*. *terrestris* is a social insect with colonies headed by a single, and singly-mated queen, that reach sizes of a few dozen to perhaps one hundred workers. During the active season, the majority of workers are infected by one to several ’strains’ (genotypes) of this parasite [8, 9]. Earlier studies have provided evidence that, both, the host and the parasite genotype (the ’strain’) are important determinants of infection outcome. For example, the resulting infection intensity varies with the paternal genetic line even among sister workers living within the same colony [10]. Furthermore, when a cocktail of different strains is infected, these will be ’filtered’ out—according to bee and parasite genotype—allowing only a few strains to transmit further [11, 12]. The study of host gene expression upon exposure to *C*. *bombi* reveals that the host responds by activating genes known to be involved in defence (e.g., anti-microbial peptides, [13]), and host gene expression patterns carry the signature of the specific host-parasite interaction [14].

Here, we employ a genome-wide association study (GWAS) approach to elucidate the genomic basis for the observed host-parasite interaction in this system. For this purpose, we used a cross-infection (fully factorial) design with worker bees of 20 host colonies exposed to 20 different strains of the parasite, amounting to a total 1,200 bees in the experiment (Table 1). We use infection intensity (i.e., the number of parasite cells per bee) as our measure of host resistance and ask what genes or genetic regions associate with this variable. We report results from two different methods: a high-coverage genetic analysis using a selected subset in an 8 x 8 (host colony x parasite strain) matrix, and a low-coverage approach for the full 20 x 20 interaction matrix design. The latter approach was motivated by the development of new analytical tools that can handle many samples each having a low coverage of their genomic sequence [15]. This presents an attractive cost-effective alternative by using many rather than few samples, with the inevitable compromise in terms of sequence coverage. Here we take the opportunity to provide data and compare these two design philosophies.

**Table 1:** Experimental design.

	Strain																			
Colony	A	B	C	D	E	F	G	H	I	J	K	L	M	N	O	P	Q	R	S	T
**Code**	**8.068**	**8.075**	**8.076**	**8.091**	**8.161**	**10.027**	**10.132**	**10.175**	**10.290**	**10.486**	**12.246**	**12.444**	**12.248**	**12.448**	**12.450**	**14.065**	**14.149**	**14.172**	**14.255**	**14.330**
**15**	3	3	3	3	3	3	3	3	3	3	3	3	3	3	3	3	3	3	3	3
**20**	3	3	2	3	3	3	3	3	3	3	3	3	3	3	3	3	3	3	3	3
**25**	3	3	3	3	3	3	3	3	3	3	3	3	2	3	3	3	3	3	3	3
**33**	3	3	3	3	3	3	3	3	3	3	3	3	3	3	3	3	3	3	3	2
**39**	3	3	3	3	3	3	3	3	3	3	3	3	3	3	3	3	3	3	3	3
**47**	3	3	3	3	3	3	3	3	3	3	3	3	3	3	3	3	3	3	3	1
**59**	3	3	3	3	3	3	3	3	3	3	3	3	3	3	3	3	3	3	3	3
**81**	3	3	3	3	3	3	3	3	3	3	3	3	3	3	3	3	3	3	3	3
**82**	3	3	3	3	3	3	3	3	3	3	3	3	3	3	3	3	3	3	3	3
**90**	3	3	3	3	3	3	3	3	3	3	3	2	3	3	3	3	3	3	3	3
**91**	3	3	3	3	3	3	3	3	3	3	3	3	3	3	3	3	3	3	3	3
**95**	2	3	3	3	3	3	3	3	3	3	3	3	3	3	3	3	3	3	3	3
**124**	3	3	3	3	1	3	3	3	3	3	3	3	3	3	3	3	2	3	3	3
**141**	3	3	3	3	3	3	3	3	3	3	3	3	3	3	3	3	3	3	3	3
**152**	3	2	3	3	3	3	3	3	3	3	3	3	3	3	3	3	3	3	3	3
**183**	3	3	3	3	3	3	3	3	3	3	3	3	3	3	3	3	3	3	3	3
**194**	3	3	3	3	2	3	3	3	3	3	3	3	3	3	3	3	3	2	3	3
**225**	3	3	3	3	3	3	3	3	3	3	3	3	3	3	3	3	3	3	3	3
**269**	3	3	3	3	3	3	3	3	3	3	3	3	3	3	3	3	3	2	3	2
**319**	3	3	3	3	3	3	3	3	3	3	3	3	3	3	3	3	3	3	3	2

The cross-infection matrix shows the number of replicates (bees) that were exposed to infection for a given combination of Colony (rows; project code, D of colony: 15,…, 319) and Strain (columns; project code of parasite strain: A,…, T). Standard code of the parasite strains (second row) indicates the label used for the long-term studies in this system. In a few cases, only two instead of three replicates could be evaluated. Total sample size is *N* = 1,198 bees for the entire matrix (corresponding to the low-coverage study), and *n* = 192 bees for the high-coverage study (8x8 matrix), indicated by the shaded cells. For choice of the subset, see text.

Previous studies also have clarified that the bees’ microbiota varies among individuals and colonies and provides protection against infections by *C*. *bombi* to varying degrees [16]; it also adds to the specific interactions in this host-parasite system [17]. Further studies show that the structure and presence of certain bacteria in the microbiota, notably *Gilliamella* and *Snodgrassella*, affect the degree of protection [18–23]. We therefore sequenced the bacterial gut metagenome for each bee (its bacterial microbiota) in the high-coverage (8 x 8) matrix and ask what microbial differences predict infection outcome and how these microbial communities correlate with host genomic variation. We finally discuss methodological lessons from this work and the problems associated with a diffuse genetic architecture of host resistance and the relevance of the interaction subsets in terms of variable defence processes.

## Material and methods

### General methods

Spring queens of *Bombus terrestris* were collected in March and April 2014 in northern Switzerland and raised to colony in the lab following our standard protocols (Table S1 in S12 File). All queens were tested for the possible presence of *Crithida* infections, using qPCR with primers (CriRTF2, CriRTR2) amplifying a 56-bp fragment of the 18S rRNA gene, see [12]; only uninfected queens and their colony were included in the experiment. The qPCR-procedure also measured infection intensity (see below). The parasites used for the experimental infections were taken from our strain collection of *C*. *bombi* clones derived from single-cell isolates from bees sampled in the field, and as used before [24, 25] (Table S2 in S12 File). Here, these clones are called ’strains’. In practice, strains are defined by their multi-locus genotype based on polymorphic microsatellites at five loci (e.g. [11]).

### Infection protocol and assessment of success

As routinely used and already described in earlier studies [11, 26], randomly selected workers (representing a snapshot of the colony resistance overall) were collected from the colonies and randomly assigned to treatment, i.e. exposure to one of the strains. For infection, the workers were starved for 2 h, before offering them a droplet of sugar water with the inoculum. For each colony x strain combination, five to six bees were exposed to the same inoculum for infection. Among those, we picked three at random to be used for the analyses. The infective dose was set at 10,000 cells per bee. The bees were sacrificed on day 7 post-exposure, the resulting infection intensity and body (wing) size measured. Both, infective dose and post-exposure time, were chosen based on the results of earlier studies assessing the effects of dose [9] and the time-dependent success of further transmission [11]. Our experiments were carried out over four weeks, with the host x strain combinations matched over this period (i.e., Monday workers were collected from colonies 1 to 5 and exposed to all parasite strains, then the corresponding scheme with Tuesday workers and colonies 6 to10, etc.) In each case, the bee’s abdomen was separated and prepared as samples for storage and for the later DNA extraction (see below). As already mentioned above, workers from twenty colonies were exposed to twenty parasite strains in a cross-infection design (Table 1). Infection intensity was assessed by quantitative PCR, using a master mix (with 212.0 μl) EvaGreen mixed with 42.4 μl Primer Cri-RT forward/reverse (5 μM each) and 593.6 μl H_2_O prepared for 96 microtiter plate; 8 μl mix plus 2 μl DNA were used per well. This method of quantifying infection intensity was already validated in an earlier study in the same system by means of dilution series [12]. Validation for the current study is shown in S12 File (assessing infection intensiy).

### DNA isolation and library preparation

For each bee, the separated abdomen was put individually in a tube and snap-frozen in liquid nitrogen. The tube was then knocked on the bench to break open the abdomen. Subsequently, the sample was transferred to a new tube provided with 20 small glass beads (0.1–0.15 mm in diameter; for homogenization and later centrifugation) and stored at -20°C until processing. We extracted the DNA from aseptically dissected whole guts, by first adding 1 ml *Buffer ATL* with *proteinase K* to each sample (manufactured by Qiagen). The buffer was previously prepared as mix of 90 ml *ATL* with 10 ml *proteinase K* to yield a volume of 100 ml per plate. After buffer was added, we homogenized the samples using a bead mill homogenizer (Bead Ruptor), cooled with liquid nitrogen to keep temperature between 15° and 25°C, for 4 x 45 s with a 20 s pause in between. The samples were then centrifuged at 12,000 g for 3 min. We subsequently took 200 μl of liquid from the middle of the tubes and transferred these extracts into a 96-well analysis plate (’blue bottom’) from Qiagen’s DNeasy Blood and Tissue Kit. The plates were processed according to the manufacturer’s instructions by incubating them at 56°C for 2 h in a plate shaker at 250 rpm, followed by adding 410 μl of *Buffer AL* with *EtOH* (mixed from 40 ml *AL* with 41.8 ml *EtOH*). Finally, the samples were eluted with 200 μl *Buffer AE* for further processing. For the low-coverage study, we reduced the volumes of the Illumina Nextera to reduce costs, and have followed the protocol given in [27]. High-coverage library preparation and sequencing was done at the Functional Genomics Centre Zurich (FGCZ) on a HiSeq 4000, using the respective Illumina Truseq kit.

### Bioinformatics

#### Low-coverage study (20 x 20 matrix)

Low-coverage libraries were sequenced (paired-end 150 nt, PE150) at an expected 2–3 x coverage on a *NovaSeq* instrument at NovoGene (Oxford and Beijing). Since the quality of the *NovaSeq* data were already very high, we did not further quality-filter the data prior to mapping. Sequences were mapped against the reference genome from Ensemble Metazoa; assembly ’Bter_1.0’, using *BWA* (v 0.0.17). Low-read, low-quality reads (e.g., strongly clipped reads, or those containing adapters) were removed (*MAQ10*), using *sambamba* (v0.6.6), and PCR-duplicates removed using *Picard Tools* (v 2.18.27). Overlapping reads were clipped using *bamUtil* (v 1.0.14). We then locally realigned indels using *gatk* (v 3.8). Samples with less than 0.5 x-coverage (a total of 10 samples) were removed (see also Fig A1 in S12 File, for the distribution of coverage). We have only used placed scaffold for the following analyses.

Genotype (SNP-) likelihoods were then calculated with *angsd* (v 0.925) [15] and, subsequently, the covariance matrix was estimated using *PCangsd* (v 0.98) [28]. We added an extra sample (e.g., DNA from a single, haploid male) to each library pool as an internal control. We also added a couple of replicates of individuals used in the GWAS. However, these samples were for controls and checking of procedures only; they were not used for the actual analyses. Among all other replicates, we chose the three samples with highest coverage for further analyses. Individuals that were not grouped according to population were removed as outliers, resulting in *n* = 1,170 individuals (i.e., a few missing due to technical difficulties). In a further step, the phenotypic data (infection intensity, using sqrt-transformed intensities to normalize variances) was corrected for the parasite strain effect with the *lm-*function in R based on approximation to normality of data. Based on the first results of the high- and low coverage study (that did not yield any host x parasite interaction effect), we focussed on the effect of host genotypic background (i.e., the ’colony’ term) alone. This interprets the parasite strain effect as the ecological background (see Discussion for justification of this rationale). For the GWAS, we then used 19 dimensions of the covariance matrix to account for population structure [29] (this number of dimensions proved to be the most effective), and the corrected phenotypic data (i.e., the residuals from the strain effect), using the score test of *angsd*. We have considered only variants with a minor frequency of more than 5% and used a total of 1.9 million sites. The genome-wide significance level was set to *P* = 0.1 after Bonferroni correction. Manhattan Plots and qqplots were generated with the R-package *GWASTools* [30]. Only the top twenty SNPs with the highest significance for an association with the phenotype are discussed here.

#### High-coverage study (8 x 8 matrix)

There are a total of over 125,000 possible combinations to form an 8 x 8-matrix from of our full (20 x 20) design. A randomly picked set of 100,000 such matrices were statistically analysed for the effect of the host x parasite interaction term on infection intensity. We chose one of these picks with the strong effect of the interaction terms for our 8 x 8 subset, with the idea to maximize the chances to find a corresponding signal with GWAS (see Supporting Information, Methods, for further details). As with the low-coverage data, and based on the first results, we removed the parasite strain effect for the further analyses and applied sqrt-transformed intensities to normalize variances. High-coverage libraries were sequenced (paired-end 150 nt, PE150) at circa 15 x-coverage on a Illumina HiSeq4000 instrument at the FGCZ. Raw reads were trimmed, using *Trimmomatic* (v 0.35), and then mapped against the reference genome (Bter_1.0), using *BWA* (v 0.0.17). Like the low-coverage study, low-quality reads were removed (*MAQ10*) and PCR-duplicates marked, using *Picard Tools* (v 2.0.1). The SNPs were then called using *freebayes* (v 1.0.2) adopting the default settings. The resulting raw variant table subsequently was filtered, using *vctools* (v1.0.1) and *vcflib* (v 1.0.1). We retained a SNP-mapping quality > 20, a mean depth > 10, minimum depth > 3, and a minor allele frequency of at least 5%. SNPs with too high coverage (more than 30 x), complex SNPs, and indels were removed. We allow for a maximum of missing sites of 5% per locus. Furthermore, all SNPs on unplaced scaffold were ignored. To correct for highly-linked SNPs, we applied a LD-pruning procedure, using *plink* (v 2), with window size of 200 bp, step size 50 bp, and a correlation threshold of *r*^2^ > 0.75. Eventually, we ended up with 504,111 high-quality SNPs across the 192 samples from the 8 x 8 matrix.

GWAS was then done on these corrected phenotypic data (as before, residuals from the parasite strain effect) whilst additionally correcting for the host relatedness effect due to similar genotypes with a given colony, using *gemma* (v 0.98.4) [31]. Again, we used a genome-wide significance level of *P* = 0.1 after Bonferroni correction, and Manhattan Plots and qqplots with the R-package *GWASTools*.

#### Analysis of the microbiota

This was done for the 8 x 8 high coverage matrix. The preparation of samples and DNA extraction for microbiota sequencing followed the protocols described above and, for example, in [32]. Samples were prepared for 16S Amplicon Sequencing. The libraries were sequenced on an Illumina MiSeq using a 600-cycle sequencing kit with paired-end 300- cycle option (PE300), using universal primers for the V3/V4 region. Because of technical difficulties with the readout of machine, we pooled reads from 4 different runs from the same Illumina MiSeq batch. In total, 48,415,850 paired-end raw reads that passed Illumina internal filtering (PF). Microbiota raw data are deposited with ENA, see also S10 File. We divided the data processing into steps according to the computing resource requirements. A detailed description of the AmpSeq workflow can be found in the file ’*4-DataPrep_ Workflow_Report’* in Appendix C of S12 File. In short, the paired-end reads were filtered (e.g., PhiX and low complexity), 3’-end trimmed, overlapping pairs were merged and the primer regions were removed. In a next step, the data was subjected to a size range, GC content range and quality filtering. The loss of data during each of these processing steps is also listed in the file. The cleaned data were then (a) clustered using UPARSE 2 (for OTUs) with a 97%-identity criterion, which produced 772 OTUs; and (b) error-corrected and zero-radius clustered (z) using UNOISE3 (for zOTUs) [33], which produced 997 zOTUs. The results were similar, but we use the latter for the analyses reported here, based on the widely accepted advantages of this latter method in terms of improved OTU identification [34–37].

We used SINTAX with the 16S reference SILVA (v128) to predict the taxonomic association for the clusters. We consider SILVA as a conservative, but reliable database. Because the critical zOTUs (see below) are of prime importance, we additionally have blasted those items against the NCBI-database. The latter database offers more taxonomic resolution, but its entries are also considered to be more error-prone.

## Results

We first report our findings from the ’low-coverage’ study, where all individuals in the entire experiment were included (n = 1,170 bees) but with lower sequencing depth. We refer to this part as the 20 x 20 (full) matrix (colonies x parasite strains) study. In the subsequent sections, the ’high-coverage’ studies are covered, which refers to bees sequenced at a widely accepted coverage level, e.g. [38]. This sample was also screened for the microbiota. We refer to this part as the 8 x 8 matrix study.

### Genetic associations from the low-coverage study (20 x 20 matrix)

#### Appendix A in supporting information

The overall variation of infection intensities per bee, across colonies or parasite strains, is shown in Fig S1 of S12 File. All of these samples were sequenced with an estimated coverage of 3.4x. After dropping all unplaced scaffolds, this value dropped to 2.1x, presumably because sequences were not covered at random and the unplaced scaffold contained many repetitive sequences. Using square-root-transformed infection intensities corrected for strain effects (see Methods; labelled as ’lmm_sqrt_res’ in the Appendix), we could use 1,782,441 SNPs to test for any association with infection intensity. None of these SNPs reached significance at the genome-wide level (Fig A2 in S12 File). However, to have at least some insights, the top twenty SNPs (i.e., with the highest levels of association) are shown in Table 2.

**Table 2:** GWAS from low-coverage study (20 x 20 matrix).

Rank	Code	SNP^c^	LG^d^	Position	Gene	Annotation^e^	Characteristic
1	CM001178.1	G/A	B10	4206573	LOC100647628	kinesin-like protein	intronic
2	CM001179.1	T/A	B09	9599615	LOC100650970	nephrin	intronic
3	CM001177.1	A/G	B11	5529605	LOC100645564	peripheral plasma membrane protein; splice variants	intronic
4	CM001169.1	C/T	B01	1931967	LOC100651512	B-cell receptor CD22	intronic
5	CM001179.1	G/A	B11	13429222	N/A	N/A	N/A
6	CM001171.1	G/T	B03	13298216	LOC105667178	uncharacterized	intronic
7	CM001175.1	G/A	B07	8603884	LOC100644761	phosphoinositide-dependent protein kinase	intronic
8	CM001181.1	G/A	B13	3266810	N/A	N/A	N/A
9	CM001171.1	C/T	B03	10561994	N/A	N/A	N/A
10	CM001180.1	T/G	B12	1072389	LOC100643982	disks large 1 tumor suppressor protein	intronic
11	CM001175.1	G/A	B07	6138344	N/A	N/A	N/A
12	CM001181.1	T/A	B13	3899059	N/A	N/A	N/A
13	CM001175.1	A/G	B07	6104364	N/A	N/A	N/A
14	CM001181.1	G/T	B13	648719	LOC100643707	transcription factor HES1	intronic
15	CM001169.1	A/G	B01	16216739	N/A	N/A	N/A
16	CM001177.1	C/T	B09	11148524	LOC100644210	dopamine D2-like receptor	intronic
17	CM001181.1	G/A	B13	702677	LOC100643707	transcription factor HES1	intronic
18	CM001179.1	G/A	B11	5045960	LOC105666228	histone-lysine N-methyltransferase SETMAR-like (gene 6179)	exon
19	CM00176.1	G/C	B08	2507890	LOC100647422	basement membrane-specific heparan sulfate proteoglycan protein	intronic
20	CM001169.1	G/A	B01	3251298	N/A	N/A	N/A

Top 20 SNPs that associate with infection intensity^a,b^. Recovery of transcripts, genes, and automated annotations.

^a^ Square-root transformed data, corrected for colony relatedness structure and strain effect. See file ’2-TopSNP_LowCoverage’ (Appendix A in S12 File).

^b^ None reaches genome-wide significance level (cutoff *P* = 0.1) after Bonferroni correction.

^c^ Major/minor allele.

^d^ Linkage group.

^e^ based on assembly Bter_1.0.

The top twenty SNPs are distributed in annotated and uncharacterized regions (’NA’, 8 out of 20 SNPs). Among the annotated ones are kinesin-like proteins, membrane proteins, or genes assigned with names similar to B-cell receptors (cells that do not exist in insects). Furthermore, the top SNPs are spread over several chromosomes, but are more common on chromosomes B09 and B11 (*Bombus* has *n* = 18 chromosomes). All but one of the specified top SNPs are intronic; some are close but many more distant from the next exon. None of these SNPs are therefore expected to be found in a transcribed product and to have a direct effect on a functioning protein. Nevertheless, their presence may affect the transcription process in some way (see Discussion). The exception to this pattern is the SNP at rank 18 that is located within a predicted exon (Table 2). A SNP in LOC10064v3982 (rank 10), annotated as *disks 1 large 1 tumor suppressor protein* is remarkable as it shows up again in the high coverage study below. The annotations (if any) for the top SNPs of this analysis are hard to reconcile with obvious functions in defences against infections. We return to these findings in the general discussion.

### Genetic associations from the high-coverage study (8x 8 matrix)

#### Appendix B in supporting information

**Fig 1:**
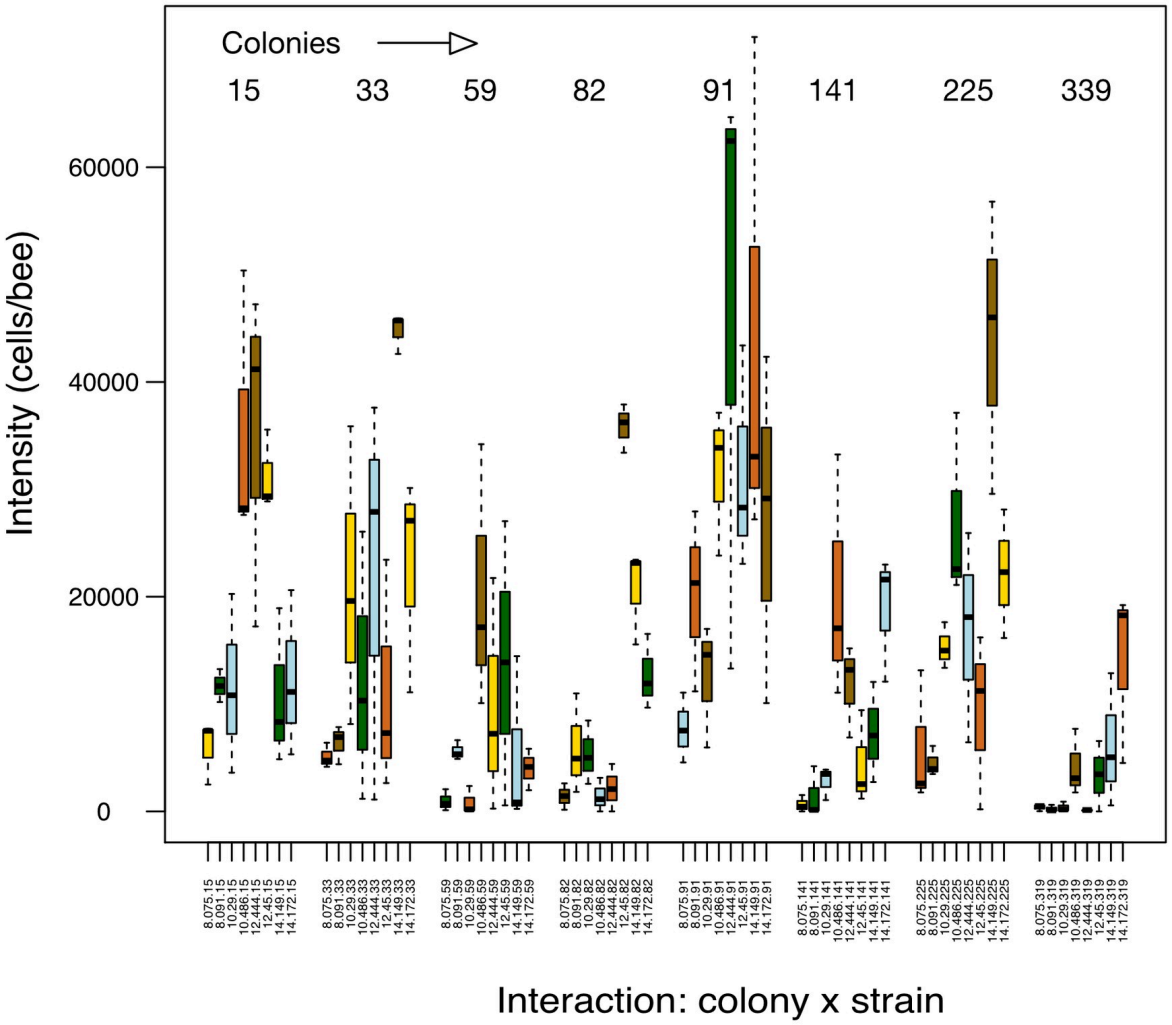
Boxplot showing the interactions in the chosen envelope of 8 x 8 colonies vs. parasite strains. Shown are the infection intensities (y-axis) and, on the x-axis, the 8 blocks (colonies, nrs. 15, 33, 59, 82, 91, 141, 225, 319) with the 8 parasite strains within each block (nrs. 8.075, 8.091, 10.290, 10.486, 12.444, 12.450, 14,149, 14.172); see also Table 1 in text. Each combination is indicated by labels with a string such as 8.075.15, indicating the combination of strain ’8.075’ with colony ’15’. Colours only chosen to aid clarity. The overall interaction term has a significance of *P* = 7.97 10^−7^.

For this section, the 8 x 8 matrix was analysed. There was considerable variation in infection intensities per bee among colonies, parasite strains, and their interaction (Fig 1). As described in Methods, infection intensities were square-root-transformed and values corrected for strain effects (label: ’lmm_sqrt_res’) based on their approximation to normality of data. In addition, we pruned the SNPs for linkage disequilibria among pairs to correct for over-representation of non-informative couplings. A total of 504,111 SNPs were available for analysis.

**Fig 2:**
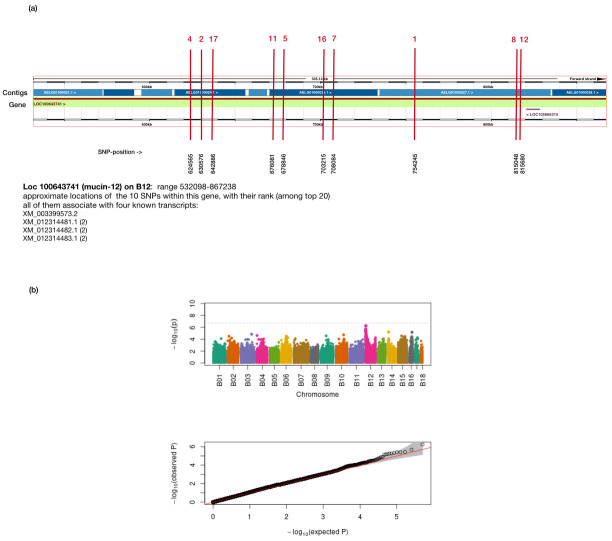
GWAS for the high-coverage study (8 x 8 matrix). **(a)** Genomic region annotated as *mucin-12* (LOC100643471). The vertical red lines indicate the position of SNPs associating with infection intensity. Red numbers above line refer to rank listed in Table 3. All SNPs are intronic and refer to four transcripts: XM_003399573.2, XM_012314481.1, XM_012314481.2, and XM_012314481.3. The region shown here extends from pos. 532,098 to 867,238. The blue band refers to contigs, the green band to the predicted gene, *mucin-12*. **(b)** Manhattan plot for the high-coverage study; data as described in text. Top panel: Probability (*p*) of association with infection intensity for SNPs along the genome (with chromosome B01 to B18 in different colours). No SNP reached the genome-wide threshold (dashed line corresponds to basic cutoff-value of *P* = 0.1 after Bonferroni correction). Bottom panel: comparing expected to observed probabilities. The calculations used sqrt-transformed infection intensities (case ’lmm_sqrt_res’ above, pruned for LD > 0.75), and using p_lrt as measure.

None of the twenty SNPs with the highest association levels (Table 3) reached the threshold level for a genome-wide association, although those on chromosome B12 came close (Fig 2; numerical value of parameter ’*p_lrt’* in Suppl Info, file *3-TopSNP_HighCoverage*). Hence, we tentatively discuss SNPs presumably associated with infection intensity. Interestingly, among the top twenty SNPs, two annotated genomic regions stand out: *mucin-12*, followed by *disks large 1 tumor suppressor protein*, both, on chromosome B12 (Table 3). It is quite striking that 12 out of the top twenty SNPs are in one of these two regions, with 10 entries alone in the vicinity of *mucin-12*. The genomic region of *mucin-12* is shown in more detail in Fig 2. A closer look shows that all SNPs of Table 3 are intronic, that is, do not fall into a genomic region translated into a protein.

**Table 3:** GWAS from high-coverage study (8 x 8matrix).

Rank	Code	SNP^c^	LG^d^	Position	Gene	Annotation^e^	Characteristic
1	CM001180.1	A/G	B12	754245	LOC100643741	mucin-12	intronic
2	CM001180.1	A/C	B12	630576	LOC100643741	mucin-12	intronic
3	CM001180.1	A/G	B12	199640	LOC100642650	elongation of very long chain fatty acids protein AAEL008004	intronic
4	CM001180.1	T/C	B12	624565	LOC100643741	mucin-12	intronic
5	CM001180.1	T/C	B12	678846	LOC100643741	mucin-12	intronic
6	CM001180.1	G/A	B12	907647	LOC100643982	disks large 1 tumor suppressor protein	intronic
7	CM001180.1	C/G	B12	708084	LOC100643741	mucin-12	intronic
8	CM001180.1	T/C	B12	815048	LOC100643741	mucin-12	intronic
9	CM001180.1	A/G	B14	1274461	LOC100645175	transcription factor 12	intronic
10	CM001184.1	A/G	B16	3562604	N/A	N/A	N/A
11	CM001180.1	G/T	B12	676081	LOC100643741	mucin-12	intronic
12	CM001180.1	A/G	B12	815680	LOC100643741	mucin-12	intronic
13	CM001180.1	A/G	B12	1382663	LOC100643982	disks large 1 tumor suppressor protein	intronic
14	CM001171.1	T/G	B03	11841846	N/A	N/A	N/A
15	CM001178.1	C/T	B10	9502791	LOC100643455	beta-1 syntrophin	intronic
16	CM001180.1	A/G	B12	703215	LOC100643741	mucin-12	intronic
17	CM001180.1	G/A	B12	642886	LOC100643741	mucin-12	intronic
18	CM001172.1	C/T	B04	640996	N/A	N/A	N/A
19	CM001177.1	A/G	B09	6824389	LOC100648443	lachesin-like transcript	intronic
20	CM001183.1	T/C	B15	4658505	N/A	N/A	N/A

Top 20 SNPs that associate with infection intensity^a,b^. Recovery of transcripts, genes, and automated annotations.

^a^ None reaches genome-wide significance level (cutoff *P* = 0.1) after Bonferroni correction. See file ’3-TopSNP_HighCoverage’ (Appendix B in S12 File).

^b^ square-root transformed data, corrected for colony relatedness structure and strain effect, and using LD-pruned data; criterion 0.75 (i.e. pairs of SNPs dropped for LD > 0.75).

^c^ Major/minor allele.

^d^ Linkage group.

^e^ based on assembly Bter_1.0.

### Role of the microbiota

#### Appendix C in supporting information

For the bees of the high coverage study (8 x 8-matrix, a total of *n* = 192 bees), we screened their gut microbiota using 16S RNA-amplicon sequencing. The reads were clustered into zOTUs (zero-radius Operational Taxonomic Units; for a technical report see files ’*4_DataPrep_Workflow_Report*’, and ’*C1_Amplicon_Technical_Report*’, Suppl Info). A list of the metagenomic data deposited with ENA (accession PRJEB52013) is given in S11 File. From the total of 192 possible samples (three replicates each), two samples were excluded for technical problems, one additional sample was included as negative control (i.e., a ’blank’ sample, with no bee material added), leaving a total of *n* = 191 samples. The blank control produced reads of zOTUs. However, compared to an average of 204,062.7 total reads per sample from the experimental bees, the control only produced 8,589 reads in total, with very low reads for all zOTUs with a slight exception: zOTU26 annotated as Bifidobacteriaceae (raw counts: *n* = 1,009, compared to *n* = 125,103 reads in the experimental bees). We conclude, therefore, that the results for the experimental bees were not significantly distorted by procedure-related errors [39]. The nucleotide sequences of all zOTUs can be found in file ’*9_16S_ZOTU*.*fa*’ of Appendix D in S12 File.

In all, each of the 8 x 8 combinations is thus represented by three bees (replicates), except for the two cases where only two replicates could be used (colony 15 exposed to strain B, and colony 319 to Q). Library sizes per sample varied, with a significant effect of the depth of coverage (number of reads) on the number of zOTUs per sample (*R* = 0.089, *F*_1,188_ = 19.53, p < 0.0001) and on zOTU diversity (rarefaction plots, Fig C1 in S12 File). The number of reads did not vary with colony or strain, but zOTU- diversity increased with sequencing depth (Fig C2 in S12 File) and varied among colonies (Fig C3 in S12 File). For the entire set, and after processing the raw data, we retrieved 997 zOTUs. Of those, only three could be identified to the species level, but half (52%) to the genus, and the majority (80% and more) to the level of family and above. Note that although we use a stringent confidence threshold of 85%, these assignments are based on homology and must therefore be treated with some caution.

**Fig 3:**
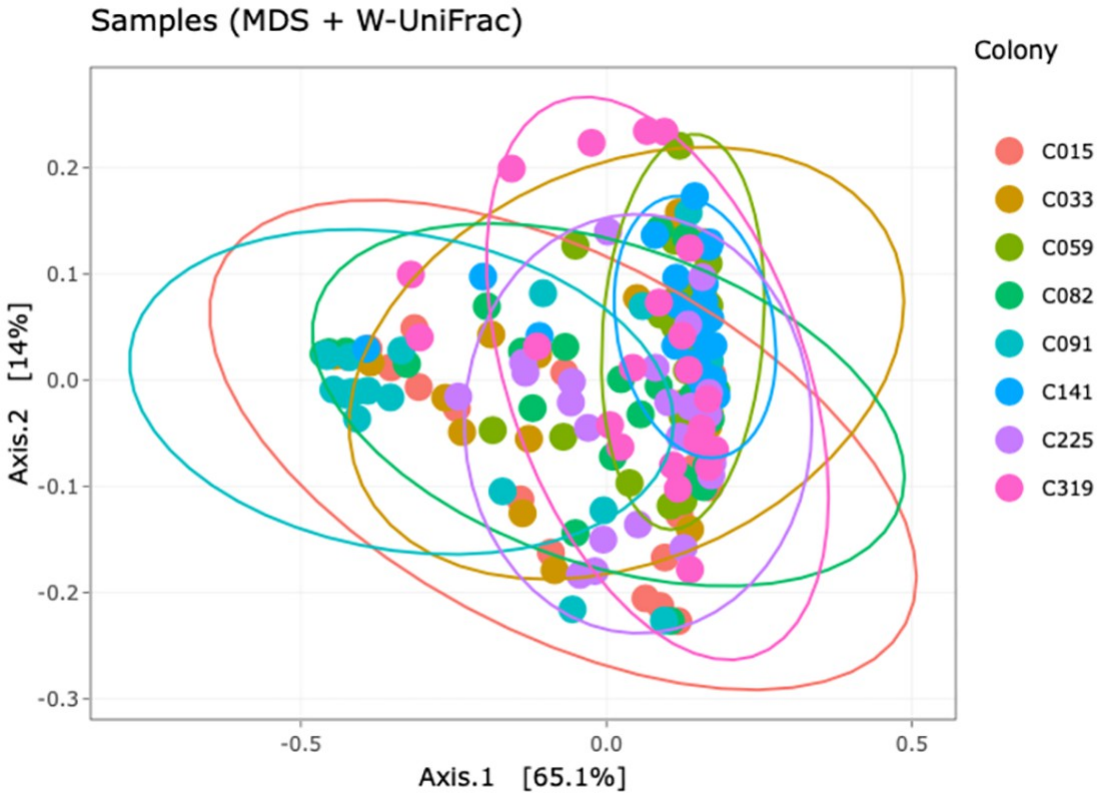
Ordination (multidimensional scaling, MDS) of microbiota samples by colony. Shown are the two primary axes (Axis.1, Axis.2) of a Principal Component Analysis (PCoA), based on weighted unifrac distances (*wuf*) between zOTUs. Numbers in brackets indicate percentage of total variation explained by the axis. Colours indicate colony (legend on the right). Samples (n = 7) with less than 10,000 reads were not considered. Colony 141 (C141, in blue) seems somewhat apart from the others, but no significant differences exist among colonies.

We analysed the pairwise distances of all samples with MDS (Multi-Dimensional Scaling) to identify the clustering of samples based on microbial community composition and abundance. We also extracted the corresponding ordination axes for further analysis. Among the various distance measures used for this purpose, ’*weighted Unifrac distanc*e’ (*wuf*) and ’*Bray-Curtis distance*’ (*bc*) were closest to normality. Finally, *wuf* was chosen for further discussion, but the conclusions would remain the same with *bc*. Fig 3 shows the results of the MDS-analysis. We find no significant difference in the zOTU-composition among colonies, with most zOTUs belonging to the Beta- and Gamma-Proteobacteria, and to a lesser degree also to the Alphaproteobacteria.

Rarefaction plots showed that for almost all samples, the zOTU richness reached a plateau with low to moderate library sizes, with only a few samples not saturating within the limits of the coverage (Fig C1 in S12 File). Because of the heterogeneity among samples, we additionally used an improved method (see [40, 41]) to derive species richness and diversity for further analyses. With this method, the three replicates per colony x strain combination served to estimate a mean and variance in these measures and for this combination. In all, we wanted to know whether the richness or diversity of zOTUs correlates with infection intensity in a given bee. For this analysis, we used the R-package ’*breakaway*’ [42] and ’*divNet*’ [43], and worked with a reduced set of 772 OTUs from the UNIPARSE procedure (see Methods). The results were as follows.

*Species (zOTU) richness*. Richness (alpha diversity) estimates with the Chao1-index are shown in Fig C2a in S12 File. A few cases (e.g., colony 141 infected by strain L) stand out, but there is no overall significant variation among samples. Diversity per sample was estimated with the Shannon-Wiener Index (package ’*divNet*’ [43], Fig C2b in S12 File). Alpha diversity varied across samples but not at a significant level. But a significant variation existed among the microbiota from different colonies (Fig C4 in S12 File). A negative relationship between infection intensity and microbiota diversity may exist but there is no clear statistical support from our data (Fig C5 in S12 File).

### Classification by microbiota composition

#### Appendix D in supporting information

In a further step, we also grouped the samples by their microbiota composition, motivated by the pattern shown in Fig 3 (i.e., a dominant spread of the data along Axis.1), and by previous studies showing that the microbiota affects infection by *Crithidia* [16, 19]. We utilized the pairwise taxonomic distances (*wuf*) between the microbiotas of the samples, leaving a total of 183 individuals in the set. As Fig 3 indicates, Axis 1 of the multi-dimensional scaling adds most to the separation of cases, explaining roughly two thirds of the total variation in the composition of the microbiota. We therefore classified samples according to whether their Axis 1 is equal or higher than zero (’positive’) or smaller than zero (’negative’). In fact, using a classification by both axes, Axis 1 and Axis 2, did not add more information (see Fig D1 in S12 File).

We were interested to see whether there are host genomic associations with microbiota composition in our data. Here again we analysed using GWAS but where the phenotype is not infection intensity but the value of Axis 1 or Axis 2, respectively. Files ’*5-TopSNP_MBiota_Ax1*’ (a total of 502, 953 SNPs) and ’*6-TopSNP_MBiota_Ax2*’ (Appendix C in S12 File) summarize the results (showing the 20 SNPs with the highest association levels). As these analyses show, SNPs that have the highest association levels with microbiota composition are either located in uncharacterized genomic regions or have annotations not directly associated with immune defences, such as *adenylate cyclase*, EH domain-binding proteins (typically involved in vesicle trafficking), mannosidases, or transporters. These genomic associations to Axis 1 and Axis 2 are difficult to understand and we cannot see a direct connection between microbiota composition and genomic regions of these SNPs.

**Fig 4:**
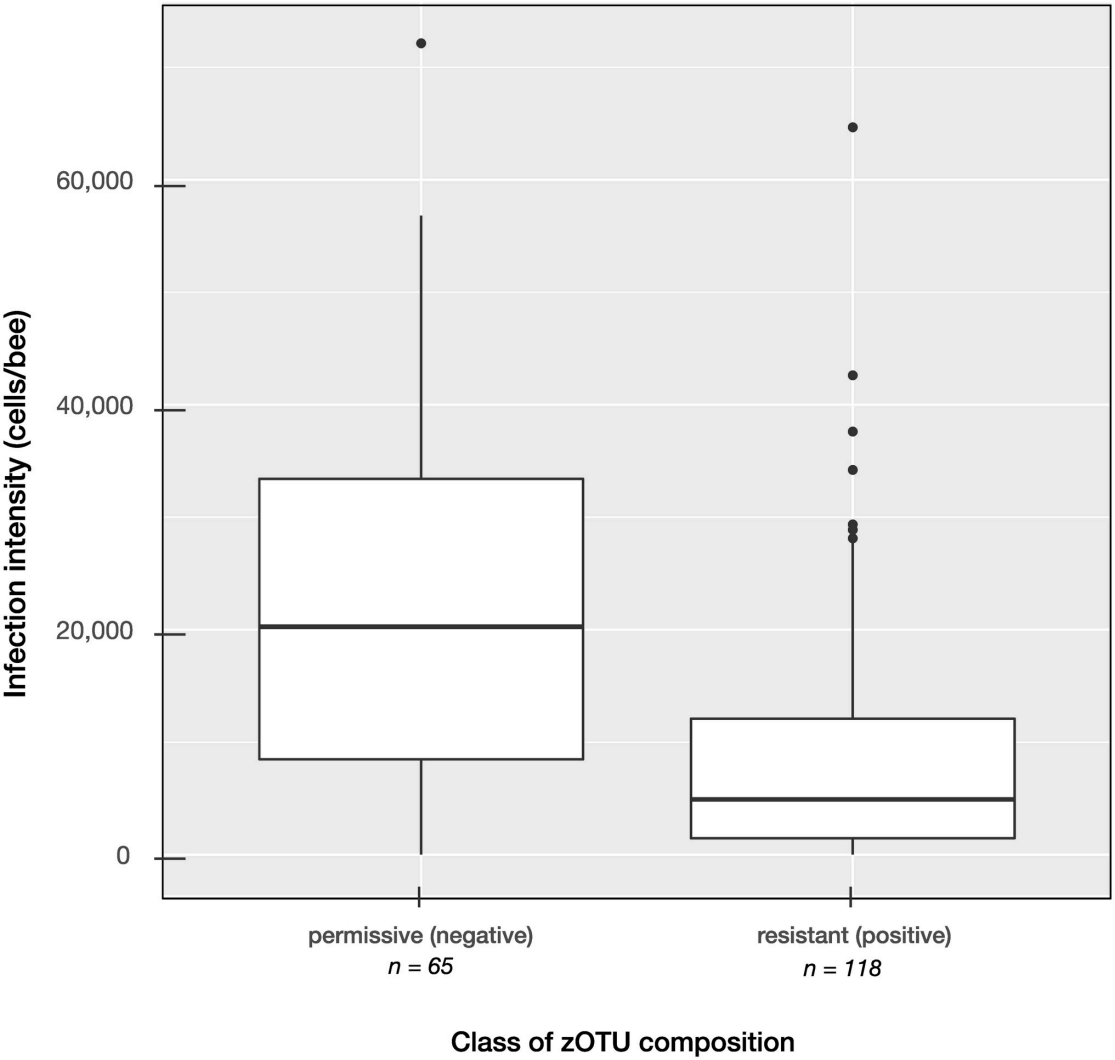
Infection intensity with respect to zOTU composition group. Samples were separated by multi-dimensional scaling (see Fig 3) and grouped by the value of their first component (Axis 1 < 0, or Axis 1 ≥ 0). The ’negative’ group (’permissive’) had significantly higher infection intensities than the ’positive’ (’resistant’) group (Walsh’s *t* = 6.12, *p* ≪ 0.0001, *df* = 95.6). zOTU-distances based on *wuf*.

The situation became clearer by looking at the GWAS-associations of, again, infection intensity as the phenotype, but for individuals grouped according to microbiota composition along Axis 1 (which covers 65.1% of the total variation, Fig 3). In fact, Fig 4 shows that infection intensity significantly differs between two groups, which we call ’resistant’ and ’susceptible’, respectively. In our case, samples with a microbiota composition that is characterized by having values of Axis 1 ≥ 0 (’positive’, ’resistant’, *n* = 118 individuals) were less infected compared to the ’negative group’ (with values Axis 1 < 0; ’permissive’, n = 65 individuals). The grouping itself is somewhat arbitrary by selecting the *wuf* distance measure, but virtually the same results would emerge from using *bc*. More importantly, Fig 4 demonstrates again that infection intensity is not independent of the microbiota and its taxonomic structure. For the two separate GWAS, the twenty SNPs with the strongest association level to infection intensity are shown in Tables 4 and 5.

**Table 4:** Top 20 SNPs associated with infection intensity in samples having Axis.1 < 0.

Rank	Location	SNP^c^	LG^d^	Position	Gene	Annotation^e^	Characteristic
1	CM001182.1	G/A	B14	5686065	LOC100642385	nucleosome assembly protein	intronic
2	CM001171.1	T/C	B03	11840228	N/A	N/A	non-coding
3	CM001171.1	A/G	B03	12218710	LOC100648315	phosphatase and actin regulator	intronic
4	CM001171.1	A/G	B03	12383573	LOC100648013	putative serine/threonine-protein kinase S6KL	intronic
5	CM001171.1	T/C	B03	12748227	N/A	N/A	N/A
6	CM001171.1	G/A	B03	13158328	LOC100650315	division abnormally delayed protein	intronic
7	CM001179.1	T/A	B11	9688234	LOC100650970	nephrin	intronic
8	CM001180.1	T/A	B12	1233258	LOC100643982	disks large 1 tumor suppressor protein	intronic
9	CM001178.1	T/C	B10	8653227	N/A	N/A	N/A
10	CM001179.1	G/A	B11	6727668	LOC100649546	uncharacterized	intronic
11	CM001183.1	T/C	B15	7927222	LOC100651177	serine protease stubble	intronic
12	CM001179.1	G/A	B11	9857303	LOC100650970	nephrin	intronic
13	CM001183.1	G/A	B15	7905861	LOC100651422	uncharacterized	intronic
14	CM001183.1	C/A	B15	8246283	N/A	N/A	N/A
15	CM001183.1	A/G	B15	8914728	LOC100646518	uncharacterized	intronic
16	CM001175.1	T/C	B07	8825700	LOC100644761	3-phosphoinositide-dependent protein kinas	non-translated region of 1 transcript
17	CM001170.1	T/C	B02	5742310	LOC100645882	protein dead ringer homolog	intronic
18	CM001183.1	G/A	B15	7466300	LOC100644824	max dimerization protein 1-like	intronic
19	CM001171.1	T/G	B03	14156904	N/A	N/A	N/A
20	CM001177.1	G/A	B09	5694646	LOC100645564	peripheral plasma membrane protein CASK	intronic

’Permissive’, grouped by multi-dimensional scaling, based on *wuf* ^a^. Phenotype is infection intensity (sqrt-transformed values, corrected for colony relatedness structure and strain effect)^b^. High-coverage study (8 x 8 matrix).

^a^ compare Fig 2; None reaches genome-wide significance level (cutoff *P* = 0.1) after Bonferroni correction. Fom file: *7-TopSNP_ MBiota_negative* (Appendix D in S12 File).

^b^ using LD-pruned data; criterion 0.75 (i.e., pairs of SNPs dropped for LD > 0.75).

^c^ Major/minor allele.

^d^ Linkage group.

^e^ based on assembly Bter_1.0.

**Table 5:** Top 20 SNPs associated with infection intensity in samples having Axis.1 ≥ 0.

Rank	Location	SNP^c^	LG^d^	Position	Gene	Annotation^e^	Characteristic
1	CM001182.1	T/C	B14	10846214	LOC100646390	lachesin	intronic
2	CM001181.1	T/C	B13	1802533	N/A	N/A	N/A
3	CM00179.1	G/A	B11	13129707	LOC105666237 LOC100642498	uncharacterized	intronic
4	CM001182.1	T/G	B14	9825985	N/A	N/A	N/A
5	CM001184.1	A/G	B16	3562604	N/A	N/A	N/A
6	CM00180.1	A/G	B12	815680	LOC100643741	mucin-12	
7	CM001178.1	A/G	B10	13330302	LOC105666188	CCR4-NOT transcription complex subunit 6-like	intronic
8	CM001182.1	T/C	B14	11198348	LOC100649003	cyclin-dependent kinase-like	exon
9	CM001184.1	G/T	B16	2728789	N/A	N/A	N/A
10	CM001178.1	G/A	B10	10428051	N/A	N/A	N/A
11	CM00171.1	T/G	B03	11841846	N/A	N/A	N/A
12	CM001184.1	G/T	B16	2967795	N/A	N/A	N/A
13	CM001184.1	G/A	B16	2991827	N/A	N/A	N/A
14	CM001184.1	G/A	B16	3277554	LOC100642943	nuclear RNA export factor 1-like	exon
15	CM00184.1	G/T	B16	2553260	LOC100648917	sorting nexin-13-like protein	exon
16	CM001178.1	T/C	B10	10745738	LOC100647933	leucine-rich repeat-containing protein	intronic
17	CM001178.1	C/T	B10	10615741	LOC100651334	solute carrier organic anion transport family member 2A1	intronic, close to exon
18	CM001172.1	G/T	B04	6651056	LOC100648473	2-acylglycerol O-acetyltransferase 1-like	in exon of 2 transcripts; intronic for 3 transcripts; rest in non-coding regions
19	CM001171.1	G/A	B03	7486034	LOC100650996	lachesin in *A*. *mellifera* (Ig-Superfamily)	intronic
20	CM001171.1	A/G	B03	7515414	LOC100650996	lachesin in *A*. *mellifera* (Ig-Superfamily)	intronic

’Resistant’, grouped by multi-dimensional scaling, based on *wuf* ^a^. Phenotype is infection intensity (sqrt-transformed values, corrected for colony relatedness structure and strain effect)^b^. High-coverage study (8 x 8 matrix).

^a^ compare Fig 2; None reaches genome-wide significance level (cutoff *P* = 0.1) after Bonferroni correction. From file: *8-TopSNP_MBiota_positiv*e (Appendix D in S12 File*)*.

^b^ using LD-pruned data; criterion 0.75 (i.e. pairs of SNPs dropped for LD > 0.75).

^c^ Major/minor allele.

^d^ Linkage group.

^e^ based on Bter_1.0.

When comparing the two tables, it seems that among the twenty SNPs (out of a total of 420,567 SNPs in the set from 65 bees) with the highest association levels with the value of Axis 1, there are more SNPs located in genes with an annotation for the permissive group (’negative’ group, Table 4) than for the resistant group (’positive group’, with 450,319 SNPs from 116 bees; Table 5). In the former group, all SNPs are intronic. Among those, the SNP at rank 8 refers to *disks large 1 tumor suppressor protein*, which was identified in the high-coverage analysis before (Table 3). Remarkably, among the SNPs of the resistant group that are in regions with an annotation, 4 out of 12 are in an exon. These are those in LOC1000649003, LOC100642943, LOC100648917, and LOC100648473 (Table 5). When looking at the annotations, two cases stand out even though they are intronic. On one hand, the SNP at rank 6 (Table 5) is in the genomic region for *mucin-12*, already prominent in the high-coverage analysis above (LOC100643741, Table 3). On the other, several SNPs locate to *lachesin*, an Ig-superfamily protein that, for example, is associated with neurogenesis and antiviral responses in insects.

**Fig 5:**
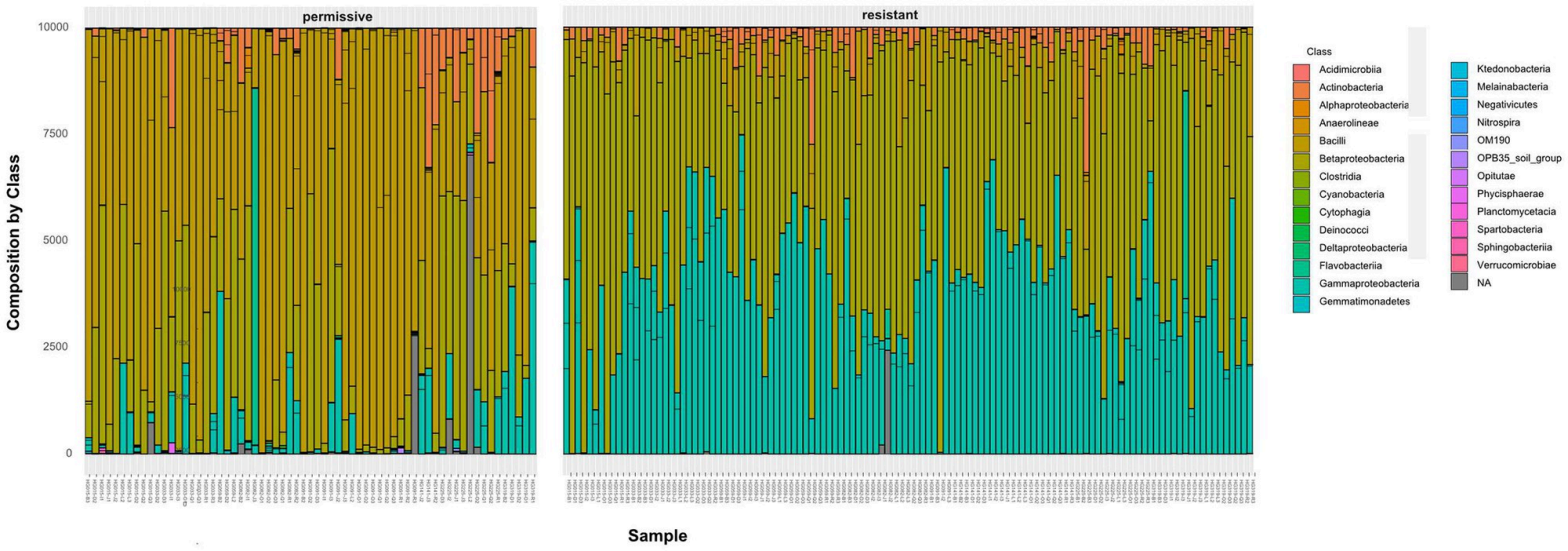
zOTU-composition at the level of systematic class. Shown are the samples in the ’permissive’ (negative; left panel) and the ’resistant’ (positive; right panel) group (see text). Bacilli/Betaproteobacteria and Gammaproteobacteria make a difference (see colour code). Distances for ordination based on weighted unifrac (*wuf*).

The zOTU-composition of resistant and permissive samples was found to be different for all diversity measures, except for the Inverse Simpson index (Fig D2 in S12 File). Resistant samples had lower zOTU-diversity in terms of Species Richness and Chao1-indices, whereas the opposite was observed when using the Shannon-Index, a finding similar to a previously reported case [22]. Similarly, the taxonomic zOTU-composition of the two groups showed differences at the level of Class (Fig 5), Phyla or Order (Figs D3 and D4 in S12 File).

Summary indices, such as Chao1 or Shannon-Wiener, are obviously very crude. A closer inspection provides more information. For this purpose, we scrutinized the abundance of the zOTUs in the resistant (positive Axis 1) vs the permissive (negative Axis 1) group. The samples were filtered as described in Methods and to exclude rare taxa (i.e., with counts less than ten). Subsequently, the difference between groups was calculated as a log-fold change (using DESeq2) in abundance between the two groups, and samples filtered further by only accepting significant changes at an adjusted *p*_adj_ < 0.1. This procedure resulted in eleven ’critical’ zOTUs that differed between groups, described by the available taxonomic information (Table 6). In particular, *Lactobacillus* was more abundant in the permissive group, whereas *Snodgrassella*, *Gilliamella*, and the (candidate) genus *Schmidhempelia* was more abundant in resistant.

**Table 6:** Critical zOTUs in individual microbiotas of resistant and permissive groups.

**(a)** zOTUs associated with Resistant							
zOTU	Kingdom	Phylum	Class	Order	Family	Genus	Species^a^
ZOTU1	Bacteria (1.00)	Proteobacteria (1.00)	Betaproteobacteria (1.00)	Neisseriales (1.00)	Neisseriaceae (1.00)	*Snodgrassella* (0.99)	Snodgrassella gandavensis*, *S*. communis*, *S*.alvi*
ZOTU2	Bacteria (1.00)	Proteobacteria (1.00)	Gammaproteobacteria (1.00)	Orbales (1.00)	Orbaceae (1.00)	--	Gilliamella bombi*, *G*. bombicola*, *G*. apis*
ZOTU4	Bacteria (1.00)	Proteobacteria (1.00)	Gammaproteobacteria (1.00)	Orbales (1.00)	Orbaceae (1.00)	*Gilliamella* (0.75)	Gilliamella bombi*, *G*. bombicola*, *G*. *apicola (*?*)*, *G*. mensalis*
ZOTU7	Bacteria (1.00)	Proteobacteria (1.00)	Gammaproteobacteria (1.00)	Orbales (1.00)	Orbaceae (1.00)	Cand_Schmidhempelia (1.00)	Gilliamella bombi*, *G*. bombicola*, *G*. apicola*
ZOTU30	Bacteria (1.00)	Proteobacteria (1.00)	Gammaproteobacteria (1.00)	Orbales (1.00)	Orbaceae	*Gilliamella* (0.99)	Gilliamella bombicola*, *G*. mensalis*, *G*. apicola*
**(b)** zOTUs associated with Permissive							
zOTU	Kingdom	Phylum	Class	Order	Family	Genus	Species^a^
ZOTU3	Bacteria (1.00)	Firmicutes (1.00)	Bacilli (1.00)	Lactobacillales (1.00)	Lactobacillaceae (1.00)	*Lactobacillus* (1.00)	Lactobacillus bombicola*, *L*. kullabergensis*^b^, *L*.kimbladi*^2^, *L*. huanngpiensis*, *L*. apis*, *L*. helsingborgensis*^b^
ZOTU6	Bacteria (1.00)	Firmicutes (1.00)	Bacilli (1.00)	Lactobacillales (1.00)	Lactobacillaceae (1.00)	*Lactobacillus* (1.00)	*Lactobacillus bombi*, Bombilactobacillus bombis*, *B*.mellis*, Apilactobacillus kunkeei*^b^
ZOTU13	Bacteria (1.00)	Firmicutes (1.00)	Bacilli (1.00)	Lactobacillales (1.00)	Lactobacillaceae (1.00)	*Lactobacillus* (1.00)	Lactobacillus apis*
ZOTU69	unresolved	--	--	--	--	--	

The samples were processed and filtered as described in Methods and contain read counts > 10, and refer to an adjusted significance level of *p*_adj_ < 0.1. Taxonomic information is from the used libraries (see text). Numbers in parentheses are bootstrap confidence levels.

^a^ additional matches from blast to the NCBI nucleotide database indicated by asterisk (*). Only matches with full (100%) sequence identity of the submitted string, and only those with an assigned regular (binomial) taxonomic name shown here.

^b^ recently described species described from honeybees.

All of the zOTUs of Table 6 were additionally blasted against the NCBI nucleotide database. This resulted in a more detailed taxonomic resolution for all these zOTUs. At the same time, however, the results are muddled by matches to several taxa. A part of these multiple matches can be explained by our reads not covering the complete sequences of the NBI database. However, an equally important source of ambiguity are the burgeoning recent changes in taxonomic nomenclature. For example, among those critical for resistant phenoytpes, zOTU1 (via NCBI) now matches *Snodgrassella gandavensis*, *S*. *communis* and *S*. *alvi*. The first two of those are very recently proposed taxa, restricted to *Snodgrassella* in bumblebees [44]. zOTU2 and zOTU7 match *Gilliamella bombis*, *G*.*bombicola*, and *G*. *apicola*. Again, the former two are recently erected taxa for *Gilliamella* found in bumble bees [45]. Similar matches are found for zOTU4, including the new species *G*. *mensalis* [45]. We note that zOTU69 is a relatively short amplicon (304bp vs. other zOTUS with over 420 bp), and perhaps a product of false priming, as it relates (via blasting) to the host genome. Excluding this zOTU does not, however, change the conclusions.

For the permissive group, the taxon zOTU3 matches several *Lactobacillus* taxa identified with he NCBI blasts. Several of those have been proposed as new species associated with honeybees (*L*. *kimbladi*, *L*. *kullabergensis*, *L*. *helsingborgensis*) [46] showing complex phylogenetic relationships [47], and are therefore unlikely to be true symbionts of bumblebees. zOTU6 is variably identified with *Lactobacillus bombi*, *Bombilactobacillus bombi*, and *B*. *mellis*, whereby *Lactobacillus* is the basonym and *Bombilactobacillus* refers to a newly defined genus found in bumblebees [48], similar to the new genus *Apilactobacillu*s in honeybees [49]. zOTU13 and zOTU30 match with *Lactobacillus apis*, which according to the revised nomenclature would refer to taxa found in honeybees only [47]. Matches are found variably on chromosomes 14 (*B*. *terrestris*), but also on chromosomes 6, 9, 13, 15, and 20 (in other *Bombus* species).

### Varying the interaction matrix

#### Appendix E in supporting information

We started this study with the goal to uncover the effects of genomic variation on the prevalence or intensity of an infection (’the infection phenotype’) due to the host x parasite genotype interaction. For this system, such interaction effects have been amply demonstrated in previous studies, e.g. [11, 12, 50], including effects on gene expression, e.g. [14, 51]. Here, we use ’host’ as a token for the genotypic background of a given host colony, whereas the parasite is defined as a *C*. *bombi* clonal genotype, based on polymorphic microsatellite loci (note that the strains used here later were also fully sequenced [25]).

**Fig 6:**
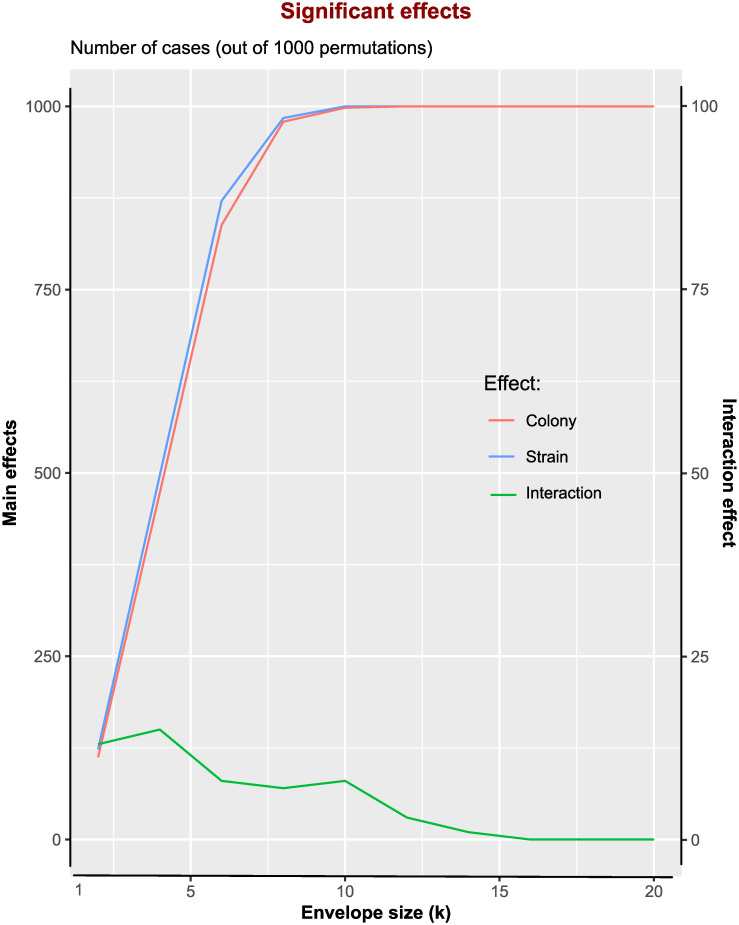
Prevalence of significant effects. Shown are the number of cases in relation to envelope size (k), and per 1,000 random permutations, where either the main effects for Colony or Strain, or the Interaction effects became significant (at level, *p*_crit_ = 0.01) in ANOVAs. Envelope size k defines the size of the analysed matrix (k x k). Infection intensity was transformed with exp = 1/3 to normalize variances. Here, all cases (incl. bees with zero infection) were used.

Contrary to these expectations, analysing the full (20 x 20)-matrix, showed that infection intensity was strongly affected by the main effects of colony and strain, but not by their interaction (ANOVA for transformed infection intensities. Colony effect: *F*_19,784_ = 20.795, *P* ≪ 0.001, Strain effect: *F*_19,784_ = 19.878, *P* ≪ 0.001, interaction: *F*_361,784_ = 0.981, *P* = 0.58). We therefore scrutinized the situation further by selecting sub-matrices of smaller dimensions, such as an 8 x 8-matrix (one of which was chosen for the high-coverage study) and then calculated the corresponding ANOVAs; we here call the respective matrix dimension (e.g., *k* = 8) the envelope size. As immediately obvious, there are many different possible sets of *k* = 8 out of *N* = 20 colonies; in fact, the exact number of possible sets is: $\left(\begin{array}{c} N\\ k \end{array}\right)=\left(\begin{array}{c} 20\\ 8 \end{array}\right)=125,970$ permutations. The same is true for selecting other values of *k* out of N strains. These must then be combined with the set of selected colonies, which together rapidly generates very large numbers of possible combinations. For this reason, we used 1,000 random picks (permutations with no replacement) of Colony x Strain set combinations, wherever the possible number of permutations exceed this value, and calculated an ANOVA for each of them. The results are summarized in Fig 6.

Regardless of the significance criterion used (i.e., *p*_crit_ = 0.05 or *p*_crit_ = 0.01) and regardless of using all cases or only infected individuals, the pattern was the same: as the envelope size, *k*, increases (i.e., the dimension of the matrix becomes larger), the main effects became more prominent whereas the interaction effects became less likely to become statistically significant. Hence, contrary to the usual expectation that with higher degrees of freedom for the interaction term, it should be more likely to find a significant interaction effect, it was actually less likely. Similarly, ANOVA estimates interaction term after the main effects have been estimated, such that less of the total variance remains available for the estimates. But this artefact cannot explain the situation here, as we discuss below.

## Discussion

### Genetic basis of infection phenotype

The work reported here attempted to identify SNPs of the host, *B*. *terrestris*, that predispose an individual to infection by *Crithidia bombi* in a cross-infection matrix. Two GWAS-methods were used on the same material—analysing a full 20 x 20 cross-infection matrix at low coverage [15], and analysing a reduced 8 x 8 matrix at high coverage. As Tables 2 and 3 show, both methods identify candidate SNPs, but the particular SNPs vary considerably. In both cases, we noted that the analysis could not separately retrieve the main (by Colony, by Strain) and interaction (Colony x Strain) effects, which would have allowed us to identify SNPs that characterize the well-described genotypic matching with those reported earlier studies [14, 51]. We therefore ignored the effect of parasite strain in the reported analysis (i.e., leading to data corrected for colony structure and strain effects). This mimics a situation where colonies would be exposed to a variety of strains, which is, in fact, what would happen in the field situation. Thus, we are capturing genomic variation that determines general susceptibility or resistance to infection by *C*. *bombi*.

The low-coverage study identified a considerable number of SNPs in uncharacterized loci or in non-coding regions. Among all loci listed in Table 2, LOC100645564 (rank 3), annotated as a possible splice variant of a plasma protein, and *disks large 1 tumor suppressor protein* (rank 10), might be involved in immune defences, whereas all other loci refer to genes with no clear link to immunological function. For the high-coverage study (8 x 8 matrix), the twenty SNPs that have the highest association with infection intensity are shown in Table 3. Note that our interpretations must be subject to the proviso that none of these SNPs reached a genome-wide level of significance. The overall picture is nevertheless telling because most of the SNPs with the highest levels of association are in annotated genomic regions. Of those, the majority (9 out of 16) are located within the gene LOC100643741, whose product is annotated as *mucin-12* (Fig 2). Another two SNPs are within LOC100645175, annotated as *disks large 1 tumor suppressor protein*, and one is similar to *lachesin*, an Ig-superfamily protein.

*Mucin-12* is identified by homology with vertebrates (mammals); usually it encodes a membrane glycoprotein as a member of the mucin family. These are very ubiquitous proteins. In many organisms, mucins form a protective mucous layer on epithelial surfaces—including the gut—and also play a role in intra-cellular signalling [52–54]. In most insects, the gut is lined with the peritrophic membrane, which is thought to derive from mucus-forming mucins, and which acts as a protective barrier [52]. In *Drosophila* [55] or locusts [56], for example, mucins are also involved in development and organ morphogenesis, including the formation of the peritrophic membrane of the midgut. Moreover, in *Drosophila*, the build-up of gut mucus is affected by infection, and the renewal of the gut epithelium after an infection-induced damage is essential for further protection and tolerance to infection [57]. Mucins in the tsetse fly are involved in the production of the peritrophic matrix and play a role in protection against *Trypanosoma* infections [58].

In *Trypanosoma cruzi* (causing Chagas disease in humans), the infective stages selectively bind to gastric mucin, mediated by *gp82*, a stage-specific putative mucin-surface protein of the parasite itself [59–61]. Interestingly, *gp63* is a surface protease of most trypanosomatids that is also involved in adhesion to the host cells [62], for example, in the closely related trypanosomatid, *Leptomonas* [63]. It is also considered a virulence factor of *Crithidia mellificae* and *Lotmaria passim* in honeybees [64], and is essential for the diversity of infections by *T*. *cruzi* [65]. The four *gp63*-variants found in *C*. *bombi* are very divergent and distinct from its close ally, *C*. *expoeki*.

Although there is no firm knowledge on the role of parasite surface proteases and host mucins in bumble bees, it is reasonable to assume that mucins are also important for the infection, establishment, and longer-term outcome of an infection by *C*. *bombi*. The sequence of *B*. *terrestris mucin-12* contains ankyrin-repeats, which typically are involved in protein-protein interactions and are among the most common protein structures more generally. A closer look, however, suggests that this *mucin-12* is probably not involved in the peritrophic matrix as it lacks mucin or chitin binding of the tsetse fly ortholog [66, 67]. Nevertheless, *mucin-12* (now re-labelled as *mucin-5AC* in the NCBI database) may remain an interesting candidate in bumblebees, as *Crithidia* needs to attach with its flagellum to the gut epithelium to establish a successful infection [68], similar to some but not all trypanosomatids [69, 70].

*Disks large 1 tumor suppressor protein* (Table 3) is also a member of a common protein family and found in many organisms. A common function seems to be in embryonic neuronal development during embryonic stages. It also serves in cellular adhesion [71–73]. Again, nothing is known about its function in bees. However, with respect to infection by *C*. *bombi*, this gene remains an interesting candidate for the same reason (adherence to the gut wall) as *mucin-12*. Both, *mucin-12* and *disks 1* are furthermore known to have several splice variants (see the annotations for assembly Bter_1.0, available via Ensemble Metazoa) whose functions are not known. Interestingly, too, a genome-wide study of the adaptive evolution of bees found that *disks large 1 tumor suppressor protein* shows signs of positive selection in social bee clades, potentially indicating a role in defence against parasites or the evolution towards sociality [74]. Finally, *lachesin* that appears in Tables 3 and 5 is typically a cell surface recognition/adhesion immunoglobulin (Ig) protein involved in insect development [75], but also in wound repair [76], and presumably in immune defences [77].

Characteristically, the vast majority of SNPs found here are intronic, with few exceptions (see Tables 2–5). In some cases, intronic regions are known to affect the phenotype [78], or the affect the process of alternative splicing process [79]. Among those, non-coding RNAs may play an important role [80]. However, as yet, nothing is known about these processes in *B*. *terrestris*. Due to close proximity, the identified SNPs genetically are linked to the expressed sequences in these genomic regions, but nothing is known about these effects either. We also note that the analyses with the low-coverage approach may not be very suitable for a complex trait, or one that is based on differential gene expression rather than genetic variation in structural genes as may be the case in our study.

### Microbiota

For the high-coverage study (8 x8 matrix) we screened the bees for their microbiota seven days post-parasite exposure. A total of 997 zOTUs entered the analyses. Not surprisingly, only half of them can only be identified at the genus level or above. To clarify the role of the microbiota further, we split the samples according to their zOTU composition using multidimensional scaling (see Fig 3), defining a ’resistant’ and a ’permissive’ group of bees (Fig 4). Regardless of the currently changing taxonomy, it seems clear from Table 6 that the bacterial genera *Snodgrassella* and *Gilliamella* are associated with a resistant phenotype, whereas susceptible phenotypes associate with *Lacotbacillus* and *Bombilactobacillus*, respectively. The taxa identified here overlap with those reported in earlier studies (Table 7). In particular, the more resistant phenotypes in our study (Table 6) as well as in previous studies (Table 7) are associated with *Gilliamella* [18, 19, 21], *Snodgrassella* [18]. These two taxa are representatives of a larger range of bacterial microbiota that shows activity against pathogens such as *C*. *bombi* but also against American foulbrood (*Paenibacillus larvae*) and others in honeybees [81]. *Lactobacillus* associates with higher infection (susceptible) in our study; the reverse is found by [21] or [23]. In addition, *Bombiscardova* has effects in other studies, being less abundant in resistant phenotypes (Table 7) [20]. *Snodgrassella alvi* is known to protect honeybees from infection by the opportunistic pathogen, *Serratia marcescens* [82].

**Table 7:** Studies on the protective effects of microbiota against *Crithidia* infections in bumble bees.

Study type ^a^	Finding ^b^	Remarks	Source
Microbiota cross-transplanted. Bacteria by TRFLP-profiles. Assessed 7 d post-exposure.	Infection affected by donor microbiota. Major effects: *Snodgrassella alvi*, *Gilliamella apicola*, *Lactobacillus*.		[17]
Field-based experimental colonies. *In situ* OTU-composition and infection status.	High OTU diversity associated with higher infection intensity. Associated with lower infection: *Gilliamella apicola*, *Snodgrassella alvi*.	Colonies under different food supply and immune-challenge treatments (with no effects).	[18]
*in situ* OTU-composition in bees from agricultural and semi-natural sites. Infections observed. Species: *B*. *bimaculatus*, *B*. *griseocollis*, *B*. *imaptiens*.	Higher richness of core bacteria associated with lower infection, but reverse for non-core bacteria. Associated with lower infection: *Gilliamella*; with higher infection: *Acetobacteraceae*,	Core bacteria defined as taxa associated with *Apis* or *Bombus*.	[19]
18 h post-exposure: (a) OTU composition. (b) Gene expression when microbiota is cross-transplanted (whole-genome transcripts).	(a) Total of159 OTUs; 19 common OTUs account for 99% of reads. Donor effect mediated by: *Snodgrassella alvi* (Neisseriaceae), *Bartonella apis*, *Bradyrhizobium erythrophlei*, *Sphingomonas*, *Bombiscadovia* (Bifidobacteriaceae), uncultured bacteria. Recipient effect mediated by: *Lactobacillus apis*, *Bartonella apis*, *Gilliamella apicola*, *Sphingomonas*, *Bombiscadovia* (Bifidobacteriaceae). (b) Effects on gene expression, more strongly affected by donors: by donor microbiota: *relish*, *hopscotch*, *vitellogenin*, *apolipophorin III*, *peroxiredoxin 5*, *jafrac*, *ctsup*, *serpin 27a*. by recipient microbiota: *defensin*, *TEPA*, *PPOP*, *serpin 27a*. - No effect of donor x recipient interaction.	Host colonies grouped as resistant or susceptible, depending on infection intensities after exposure.	[20]
*in situ* OTU-composition in bees from the wild and two habitats (urban, forest). Infections observed.	OTUs with suspected effect: *Gilliamella*, *Snodgrassella*. No association of OTU-diversity with infection.	Urban microbiota dominated by core bacteria (i.e. those commonly associated with bees). Forest microbiota by environmental bacteria.	[101]
OTU-composition before and after (7 d post-exposure).	High pre-infection OTU diversity associated with higher infection intensity. OTUs with effects: *Snodgrassella*, *Gilliamella*, *Lactobacillus*, *Orbus*, *Bombiscardovia*. Post-infection OTU diversity not changed and not related to infection outcome.		[22]
Microbiota cross-transplanted from queens into naïve wild and commercial worker bees. Infection 7 d post-transplant, assessed 7 d post-exposure. Species: *B*. *impatiens*.	High OTU diversity associated with lower infection intensity. Associated with lower infection: *Apibacter*, *Lactobacillus* Firm-5, *Gilliamella*. No association: *Snodgrassella alvi*.	Subtle differences in microbiota affect infection.	[21]
Inhibition of *Crithidia* in culture, originating from wild bees in Vermont, Illinois. Bacteria isolated from European *B*. *lapidarius*.	Associated with lower infection: *Lactobacillus* (Firm-5) *bombicola* at low pH.	Resistance mediated by pH of the gut. pH affected by microbiota, especially *Lactobacillus*.	[23]

^a^ the cited studies used *Bombus terrestris*, except where mentioned. All studies by metagenomics, except where mentioned.

^b^ likely taxonomic classification at the time of study (closest BLAST hits).

The corresponding GWAS-analyses for the two groups found many of the top 20 SNPs associated with the permissive group in regions with uncharacterized loci or non-coding regions (Table 4). For the resistant group, where 12 of 20 top SNPs were located in annotated regions, four SNPs located within an exon (Table 5). Interestingly, across analyses, the genomic region of *mucin-12* (see Tables 3 and 5), *disks large 1 tumor suppressor protein* (Tables 2–4) and *lachesin* (Tables 3 and 5) appeared repeatedly. Nothing is known about the function of these genes in bumble bees. Yet, a possible significance of these genes for directly influencing infections by *Crithidia*, or indirectly by supporting microbial communities that protect against an infection is likely.

Published gene expression studies (Table 8) in this system (and elsewhere) tend to use early phases of infection to examine expression, typically 18 h to 48 h post-exposure. This time window covers the early responses against the infection, which, in bumble bees, extends over hours to a few days [83–85]. By contrast, GWAS typically addresses the ’static’ genetic architecture of the defence machinery, as no ongoing expression is measured. Moreover, in our study, GWAS is assessed by a phenotype indicating the long-term outcome of infection, i.e., infection intensity 7 d post-exposure. This time interval corresponds to the development of a full infection by *C*. *bombi* [86, 87] (doubling time of *C*. *bombi* in the host is estimated at 10 to 14 h [24]). So far, there seems to be no GWAS study that analyses long-term infection intensity as we do here. Rather, several GWAS studies in bumble bees address morphological traits, such as colouration [88, 89], or tongue length [90]. A direct comparison with other studies is therefore not yet feasible.

**Table 8:** Synopsis of results from various expression studies on *Crithidia* infections in bumble bees.

Study type ^a^	Finding, significant effects upon infection ^b^	Remarks ^c^	Source
Candidate gene expression. 24 h / 48 h post-exposure.	Upregulation: *abaecin*, *hymenoptaecin*, *defensin*.	Clear effect of colony. GxG interaction (but not for *abaecin*).	[51]
Candidate gene expression. 10 d post-exposure.	Upregulation: *hemomucin*, *relish*. No effect: M*yD88*, *TEP*	4 candidate genes.	[102]
Candidate gene expression. Time series (1,2,3,4,5,24,26, 48 h) post-exposure.	Upregulation (5 genes): *IK2*, *peroxidase*, *calcineurin*, *Tamo-like*, *plexin A*. No effect: *vitellogenin*, *HDLBP*, *fat-spondin*, *cathespin*, *serpin*, *aconitase*, *Sur8*, *Hsp90*, *Ran-type molecule*.	14 candidate genes. Focus on temporal course of expression (from 1… 24 h post-infection).	[85]
Candidate gene expression. 18 h post-exposure.	Upregulation: *abaecin*, *lysozyme*, *serpin 27a*. No effect (in most genes): *hemomucin*, *relish*, *basket*, *defensin*, *hymenoptaecin*, *peroxidase*, *transferrin*, *ferritin*, *TEP A*.	28 candidate genes. Bees from two localities; slight differences found. Effects of colony pervasive.	[103]
Candidate gene expression. 18 h post-exposure.	Upregulation: *BGRP1*, *hopscotch*, *abaecin*, *hymenoptaecin*, *ferritin*, *lysozyme 3*, *peroxiredoxin 5*, *cytochrome P450*. Downregulation: TEP A.	26 candidate genes. Upregulation in many receptor and effector genes, partly in signalling and metabolic genes. Strong effect of parasite clone (strain). Effect of colony and colony x strain interaction (GxG). Expression of genes correlated.	[26]
Whole-genome transcripts. 18 h post-exposure.	Effect of GxG exposure: *apidaecin*, *abaecin*, *estersae FF4*, *limkain-b1*, *LRR GPCR4*, *SPN3*. Effect of infecting strain: *putative AMP*, *exonuclease*, *synacptic vesc*. *glycoprotein*, *MELK*, *cytochrome P450*, *apidemin 2*, *maelstrom*, *Spatzle 4*, four uncharacterized loci.	Focus on expression effects under GxG Interaction	[14]
Whole-genome transcripts. 24h / 48 h post-exposure.	Differential expression (up/down) according to broad function for: peritrophic membrane (*fibrillin*, *chitinases*, *peritrophin*), receptors (*Dscam* variants, *GNBP*s,), serpins (proteases, inhibitors), signalling (S*paetzle*, *myD88*, *dorsal*,…), effectors (*defensin*, *hymenoptaecin*, *apidaecin*, *hemolectin*, *TPX4*, *eater*,…), metabolism (*cytochrom P450*), and with various functions (*royal jelly protei*n,…).	A total of 489 genes affected. 591 genes affected specifically under a GxG interaction. 24 h post-infection: 165 transcripts upregulated, 324 downregulated. Several genes are alternatively spliced and expressed.	[93]

^a^ The cited studies used *Bombus terrestris*.

^b^ For specification of genes, see source.

^c^ GxG interaction refers to host genotype x parasite genotype differential expression.

Interestingly, gene expression studies of the *Bombus-Crithidia* system typically identify changes in the expression of anti-microbial peptides, such as *abaecin*, *apidaecin*, *hymenoptaecin*, or *defensin*, but also genes expressed for the recognition of parasites (*GNBP*, *Dscam*), for immune signalling (*Spaetzle*, *myD88*), or transcription factors and activators (*relish*, *dorsal*; Table 8). As mentioned above, these genes are involved in the early response to infection when the expression is also measured. By contrast, our ’static’ GWAS uncovers a more permanent genetic trace, where *mucin-12* ranks prominently.

For the longer-term outcome of an infection, the microbiota is an essential determinant. Among the critical taxa identified here (Table 6), *Snodgrassella* and *Gilliamella* line the ileum as a biofilm [91] and are suspected to competitively inhibit the attachment of trypanosomatids to the gut wall [92]. It is therefore conceivable that the *mucin-*microbiota-*Crithidia* nexus is the key to the establishment and control of infection in *Bombus* after the early responses (characterized by the expression anti-microbial peptides, reactive oxygen species, or changes in metabolism) have run their course. Indeed, the possible functions of the most prominent genes found here seems to revolve around cell surface and binding properties. Adhesion to host cells is a key requirement for *C*. *bombi* to successfully infect [68]. Similarly, the parasite-genotype x host-genotype interaction signature can be retrieved for gene expression in the early responses [14, 51, 93], but was no longer present in our static genetic analyses by GWAS. At the same time, the rapidly expressed anti-microbial peptides (AMPs) are often evolutionarily conservative and evolve slower than other immune genes [94]. However, they can be expressed differently and hence used as a tailor-made cocktail of compounds, whose effects on infecting parasites, such as *C*. *bombi*, potentiates and depends on the quantitative combination of the single AMPs [95, 96]. The importance of this flexible strategy is plausible by the need for a quick and effective response as early as possible in the course of an infection [97]. This differential use of AMPs is what is likely visible in the early responses, and emerges in form of a variable expression profile (Table 8). By contrast, the main process in the longer-term responses may be binding of parasite cells to host cell surfaces, where the more constitutive protective mucus layer and the established microbiota become the key players. At the time being, this remains a hypothetical conjecture but opens interesting perspectives.

### The interaction matrix

We did not find a parasite-genotype x host-genotype interaction effect when statistically analysing the infection phenotype (i.e., infection intensity) in the full 20 x 20 matrix, whereas the main effects of colony (host genotypic background) and parasite strain were highly significant (Table E1 in S12 File). We propose that the pattern of losing an interaction effect as more colony x strain combinations are included in a matrix (Fig 6) likely reflects a biological effect rather than a statistical artefact. In a basic scenario, when there is a high degree of specificity in the host-parasite interaction, the interaction effect should indeed become more prominent as more combinations are tested, i.e., when the interaction matrix has a larger envelope (more host-genotypes by more parasite-genotypes). However, this is only true when the specificities are ’homogeneous in kind’, that is, they function in the same way; in the extreme case, when different colonies only succumb to one, but different parasite strain each. However, there are at least two elements that change this basic scenario.

Firstly, the interaction of a particular colony with a particular parasite strain is specific in the sense that, primarily, the trajectory of the developing infection in host varies among hosts, among infecting parasite strains and their combination. This can be conceptually illustrated by the infection trajectory in the host’s ’disease space’—which, for example, may be defined by the axes of host health status and infection intensity [98]. As a result, the outcome—that is, the infection intensity as measured here—might be extremely variable due to, for instance, different defence mechanisms becoming activated along the specific trajectory. To put it figuratively, in a given host-parasite combination *A*, the infection may result in upregulated host metabolic activity, whereas in a different host-parasite combination *B*, infection may instead alter the expression of anti-microbial peptides. This amounts to the scenario that multiple or alternative genetic pathways are recruited in different host x parasite combinations. Such phenomena have been documented in our system [14, 26]. As a result, the trajectories of health and disease may unfold in very different ways and, therefore, generate very non-linear, non-homogeneous infection outcomes (with their associated genetic architecture) despite an otherwise ordered matrix of host-parasite interactions. Secondly, in the *Bombus-Crithidia* system, the situation is also different from the basic scenario because the colony specificities act more like a quantitative filter, i.e., colonies are susceptible to different range of parasite strains; for example, a colony may be susceptible to few or to many different strains on top of being susceptible to some more than to others. Note that interaction effects still exist—a particular colony will interact with a particular parasite strain in specific ways, different from any other combination. It is just that the above mentioned deviations from the basic scenario eventually will undermine the statistical interaction effect as more colonies and parasite strains are included in the analysed interaction matrix.

## Conclusion

Any host colony is typically exposed to a large number of parasite strains over its life, e.g. [99, 100], which is the relevant level of selection in its environment (although we put aside the common and important situation of simultaneous infections with multiple parasite genotypes [24]). In this situation, averaged over the life span of colonies, the specific host-parasite interaction effects add to eventual fitness and, for example, support a role of rapidly recruited anti-microbial peptides (e.g., as revealed in expression studies). At the same time, the basic overall genetic architecture of a colony is associated with a general susceptibility to infection or the maintenance of the microbiota (and can be identified by GWAS). Based on our results, we propose that, for this effect, parasite adhesion to host cell surfaces may be the key process.

## Supporting information

S1 File BasicData. The file contains data from all of the *N* = 1,200 bees that were tested.
(XLSX)

S2 File TopSNP_LowCoverage. The file contains the top 20 SNPs associated with infection intensity in the 20 x 20 matrix.
(XLSX)

S3 File TopSNP_HighCoverage. The file contains the top 20 SNPs associated with infection intensity in the 8 x 8 matrix, using sqrt-transformed phenotype data, corrected for the strain effect, and pruned from linkage disequilibria *LD* > 0.75.
(XLSX)

S4 File DataPrep_Workflow_Report. The file contains the detailed report on the Amplicon sequencing procedure.
(PDF)

S5 File TopSNP_MBiota_Ax1. The file contains data from the top 20 SNPs associated with Axis.1 of the Multi-Dimensional Scaling (MDS) of zOTUs. Values for Axis.1 were LD-pruned with LD > 0.75.
(XLSX)

S6 File TopSNP_MBiota_Ax2. The file contains data from the top 20 SNPs associated with Axis.2 of MDS-ordination of OTUs. Values for Axis.2 were LD-pruned with LD > 0.75.
(XLSX)

S7 File TopSNP_MBiota_negative. The file contains data from the top 20 SNPs associated with cases where Axis.1 < 0 (’permissive’ group, from MDS-ordination of zOTUs. Values for Axis.1 corrected for structure, sqrt-transformed, and LD-pruned.
(XLSX)

S8 File TopSNP_MBiota_positive. The file contains data from the top 20 SNPs associated with cases where Axis.1 ≥ 0 (’resistant group’), from MDS-ordination of zOTUs. Values for Axis.1 corrected for structure, sqrt-transformed, and LD-pruned.
(XLSX)

S9 File 16S_ZOTU.fa. The file contains the nucleotide sequences of all identified zOTUs in the project.
(FA) Click here for additional data file.

S10 File Amplicon_Technical_Report. This file contains the technical report for the sequencing of the bacterial metagenome.
(TXT)

S11 File fastq2_Samples. This file contains a list of all samples from which the bacterial metagenome was typed and deposited with ENA (accession PRJEB52013). We used universal primers for the V3/V4 region of 16S RNA. A key to sample numbering is given.
(PDF)

S12 File Explanations. File contains several sections. Methods: assessing infection intensity, selecting the 8x8 sub-matrix. Basic data: explanations, lists of colonies and parasite strains. Variation of infection intensity across colonies and strains. Explanations and supporting results for data in Appendices A to E.
(PDF)
