# Supplementary material for: Genetic variation and microbiota in bumble bees cross-infected by different strains of C. bombi
Source: PLoS One. 2022 Nov 28;17(11):e0277041. doi: 10.1371/journal.pone.0277041 (PMC9704641; doi:10.1371/journal.pone.0277041)
Supplement: S4 File — The file contains the detailed report on the Amplicon sequencing procedure. (PDF) [file pone.0277041.s004.pdf]

```
==========================================================================
============
 P R O J E C T - H E L P - F I L E
==========================================================================
============
Project  : p257 (HolyGrail)
Run      : Run160404 / Run160415 / run160429 / run160502
Platfom  : Illumina MiSeq
Provider : GDC, ETHZ
Data Typ : PE301
Target   : 16S
N(Sample): 191
==========================================================================
============
Step A | Data and Parameter Evaluation
==========================================================================
============
START_Workflow_StepA: 15:12:11 07/01/2021
>> HPC Euler, ETH Zurich
--------------------------------------------------------------------------
------------
Application: usearch v11.0.667_i86linux64
--------------------------------------------------------------------------
------------
N(reads)= 48,415,850
N(IDs): 4 (@M01072:142/@M01072:146/@M01072:150/@M01072:151)
Primer Site Matches: 0.86 / 0.86
Merging: 91.58% (FLASH v1.2.11)
- - -
Primer Site Error Rate:
   N(merged): 44340180 (-4075670)
   Mismatch: 0-0 / U341F / U806R / n: 35906611
   Mismatch: 0-0 / U806R / U341F / n: 24
   Mismatch: 0-1 / U341F / U806R / n: 1379631
   Mismatch: 0-1 / U806R / U341F / n: 5
   Mismatch: 0-2 / U341F / U806R / n: 194411
   Mismatch: 0-2 / U806R / U341F / n: 2
   Mismatch: 0-3 / U341F / U806R / n: 260297
   Mismatch: 1-0 / U341F / U806R / n: 1808085
   Mismatch: 1-0 / U806R / U341F / n: 9
   Mismatch: 1-1 / U341F / U806R / n: 145695
   Mismatch: 1-2 / U341F / U806R / n: 541985
   Mismatch: 1-3 / U341F / U806R / n: 460841
   Mismatch: 2-0 / U341F / U806R / n: 114027
   Mismatch: 2-0 / U806R / U341F / n: 1
   Mismatch: 2-1 / U341F / U806R / n: 11836
   Mismatch: 2-2 / U341F / U806R / n: 42835
   Mismatch: 2-3 / U341F / U806R / n: 81863
   Mismatch: 3-0 / U341F / U806R / n: 136895
   Mismatch: 3-0 / U806R / U341F / n: 1
   Mismatch: 3-1 / U341F / U806R / n: 7486
   Mismatch: 3-2 / U341F / U806R / n: 3974
   Mismatch: 3-3 / U341F / U806R / n: 12227
   Mismatch: 3-3 / U806R / U341F / n: 1
```

```
– – –
Primer Site Evaluation:
   ➜ PF:
       3742 ..........G....A
       4252 CC..............
       4771 ...A.........G..
       6181 .............GC.
       6862 .............G.A
       6914 .............GCA
       6946 C...........W...
       9655 .CT.............
      10831 C..............A
      12696 ...A...........A
      14528 ........D.......
      22135 ..T.............
      36622 .C..............
      45227 C.T.............
      55923 ...........C....
      60351 ....C...........
      68515 .....G..........
      68731 ......G.........
      77722 ..............CA
      84096 .........G......
     120003 ..............C.
     126905 ..........G.....
     127788 C.TA............
     129070 .......G........
     169279 .............G..
     173062 ............W...
     376601 C...............
     408271 ...A............
    1042514 ...............A
   37740950 ................
   ➜ PR:
      20451 .................A.T.
      21831 ...................TC
      22387 ........V............
      24176 ....................C
      25350 ...................T.
      26728 ................TAA..
      28530 ................T....
      31380 ................T..TC
      35711 G.AC.................
      37281 ..............T......
      40862 ....T................
      45900 .............M.......
      46321 ............T........
      46624 .................A.TC
      50002 .G...................
      55785 ......C..............
      63675 ..........G..........
      68089 G.A..................
      80695 .........G...........
     102761 ...........G.........
```

```
      108246 .....A..............
      114899 .................AA.C
      128086 G...................
      193150 ..................A..
      200288 ..................ATC
      226839 ..A.................
      231296 .................A...
      275383 .................AAT.
      517133 .................AA..
    37965618 ....................
- - -
PhiX: < 0.01%
- - -
Read Length Distribution:
   Lengths min 35, lo_quartile 301, median 301, hi_quartile 301, max
301
     20  0.003638
     40  0.000049
     60  0.000096
     80  0.000180
    100  0.000891
    120  0.000229
    140  0.001890
    160  0.001325
    180  0.003932
    200  0.001228
    220  0.001292
    240  0.001168
    260  0.001123
    280 ******** 0.110886
    300 ************************************************************
0.872073
- - -
Error Distribution:
   R1 (EE mean 1.8; min 0.0, lo_quartile 0.2, median 0.8,
hi_quartile 2.1, max 30.0)
```

Length	MaxEE 0.50	MaxEE 1.00	MaxEE 2.00
50	47981934(99.1%)	48187398(99.5%)	48235889(99.6%)
60	47751821(98.6%)	48144286(99.4%)	48226146(99.6%)
70	47221980(97.5%)	48049563(99.2%)	48207288(99.6%)
80	46961658(97.0%)	47949056(99.0%)	48179051(99.5%)
90	46640767(96.3%)	47793978(98.7%)	48136726(99.4%)
100	46288315(95.6%)	47596462(98.3%)	48086491(99.3%)
110	45827959(94.7%)	47310053(97.7%)	47979492(99.1%)
120	45334310(93.6%)	47007066(97.1%)	47854436(98.8%)
130	44822607(92.6%)	46634670(96.3%)	47671710(98.5%)
140	44358079(91.6%)	46276728(95.6%)	47466325(98.0%)
150	43912539(90.7%)	45941668(94.9%)	47243072(97.6%)
160	43362086(89.6%)	45527097(94.0%)	46947549(97.0%)
170	42878207(88.6%)	45191216(93.3%)	46723120(96.5%)
180	42329765(87.4%)	44804815(92.5%)	46481162(96.0%)
190	41590442(85.9%)	44256996(91.4%)	46089826(95.2%)
200	40834289(84.3%)	43729185(90.3%)	45773418(94.5%)

```
      210   39811417( 82.2%)   43038423( 88.9%)   45352880( 93.7%)
      220   38737586( 80.0%)   42277103( 87.3%)   44888895( 92.7%)
      230   37579248( 77.6%)   41472734( 85.7%)   44401721( 91.7%)
      240   35935190( 74.2%)   40455170( 83.6%)   43811260( 90.5%) <
      250   34040706( 70.3%)   39001908( 80.6%)   42877325( 88.6%)
      260   32387246( 66.9%)   37733612( 77.9%)   42036392( 86.8%)
      270   27697969( 57.2%)   33740002( 69.7%)   40197262( 83.0%)
      280   24702939( 51.0%)   31512116( 65.1%)   38754682( 80.0%)
      290   22876176( 47.2%)   29587277( 61.1%)   37394374( 77.2%)
      300   17813187( 36.8%)   23765687( 49.1%)   31200044( 64.4%)
   R2 (EE mean 5.5; min 0.0, lo_quartile 2.4, median 4.4,
hi_quartile 7.2, max 39.5)
   Length        MaxEE 0.50         MaxEE 1.00         MaxEE 2.00
   ------   ----------------   ----------------   ----------------
       50   46253502( 95.5%)   47275845( 97.6%)   47949498( 99.0%)
       60   45833178( 94.7%)   47007854( 97.1%)   47744448( 98.6%)
       70   45274475( 93.5%)   46744296( 96.5%)   47567395( 98.2%)
       80   44679400( 92.3%)   46390370( 95.8%)   47372136( 97.8%)
       90   44223273( 91.3%)   46083529( 95.2%)   47207207( 97.5%)
      100   43740763( 90.3%)   45764811( 94.5%)   47042887( 97.2%)
      110   43104738( 89.0%)   45344873( 93.7%)   46810273( 96.7%)
      120   42410083( 87.6%)   44946471( 92.8%)   46604304( 96.3%)
      130   41589709( 85.9%)   44458552( 91.8%)   46339899( 95.7%)
      140   40559302( 83.8%)   43861317( 90.6%)   46036142( 95.1%)
      150   39189686( 80.9%)   43085975( 89.0%)   45621863( 94.2%)
      160   37745468( 78.0%)   42152854( 87.1%)   45078127( 93.1%)
      170   36544682( 75.5%)   41379134( 85.5%)   44630611( 92.2%)
      180   34960388( 72.2%)   40341689( 83.3%)   44029404( 90.9%) <
      190   33345437( 68.9%)   39206567( 81.0%)   43315174( 89.5%)
      200   30938215( 63.9%)   37605690( 77.7%)   42456230( 87.7%)
      210   28241665( 58.3%)   35631069( 73.6%)   41342819( 85.4%)
      220   24731330( 51.1%)   32916543( 68.0%)   39779487( 82.2%)
      230   21475147( 44.4%)   30010925( 62.0%)   37896566( 78.3%)
      240   16360230( 33.8%)   24004071( 49.6%)   34023104( 70.3%)
      250   12677557( 26.2%)   20403972( 42.1%)   30679469( 63.4%)
      260    9430017( 19.5%)   16613890( 34.3%)   26750848( 55.3%)
      270    6344935( 13.1%)   12545807( 25.9%)   22317200( 46.1%)
      280    4416524(  9.1%)    9579561( 19.8%)   18515206( 38.2%)
      290    2362578(  4.9%)    5978826( 12.3%)   13533684( 28.0%)
      300    1159064(  2.4%)    3439484(  7.1%)    9256917( 19.1%)
- - -
   Nucleotide Composition
   R1: G 31.8%, A 26.2%, T 21.0%, C 20.9%, N 0.1%
   R2: C 33.5%, T 27.2%, G 19.7%, A 19.5%, N 0.1%
....................................................................
............
END_Workflow_StepA: 15:27:04 07/01/2021
====================================================================
============
Step B | Quality Filtering and Read Pair Merging
====================================================================
============
Workflow Overview:
◇ B1  - Check-Point: Verify Sample Names
```

```
◇ B2  - Check-Point: Sample File Resolution
◇ B3  - GZ-2-FQ and Renaming Files
◇ B4  - Remove PhiX
.....................................................................
............
       Application: usearch v11.0.667_i86linux64
           Command: filter_phix
.....................................................................
............
◇ B5  - Remove Low Complexity Sequences
.....................................................................
............
       Application: usearch v11.0.667_i86linux64
           Command: filter_lowc
         Threshold: 25
.....................................................................
............
◇ B6a - Trim Read End
.....................................................................
............
       Application: usearch v11.0.667_i86linux64
           Command: fastx_truncate
        Trim R1/R2: 30 / 70
.....................................................................
............
◇ B6b - Sync Read-Pairs
       Application: usearch v11.0.667_i86linux64
           Command: fastx_syncpairs
.....................................................................
............
◇ B6c - Merge Read Pairs (Bayesian PE Read Merger)
.....................................................................
............
       Application: usearch v11.0.667_i86linux64
           Command: fastq_mergepairs
       Min Overlap: 20
     Min %Identity: 60
 Min Merged Length: 100
Min Merged Quality: 0
---------------------------------------------------------------------
------------
START_Workflow_StepB: 15:54:52 07/01/2021
---------------------------------------------------------------------
------------
 Start_B1_UniqueSampleNames_Check: 15:54:52 07/01/2021
  ✔ PASS - Unique Sample Names (N=191)
 End_B1_UniqueSampleNames_Check: 15:54:52 07/01/2021
.....................................................................
............
 Start_B2_SampleResolutionCheck: 15:54:52 07/01/2021
  ✔ PASS - Sample-Name Resolution Check (n=191)
 End_B2_SmpleResolutionCheck: 15:54:55 07/01/2021
.....................................................................
............
```

Start_B3_GZ-2-FQ: 15:54:55 07/01/2021
 ➸ N(FilesR1/FilesR2): 191 / 191
End_B3_GZ-2-FQ: 16:05:25 07/01/2021
....
Start_B4_RemovePhiX: 16:05:25 07/01/2021
 HG015-B1: N(PhiX_free)= 252217 (-2)
 HG015-B3: N(PhiX_free)= 198881 (-69)
 HG015-D1: N(PhiX_free)= 221524 (-7)
 HG015-D2: N(PhiX_free)= 307840 (-8)
 HG015-D3: N(PhiX_free)= 174075 (-2)
 HG015-I1: N(PhiX_free)= 120254 (-11)
 HG015-I2: N(PhiX_free)= 190259 (-7)
 HG015-I3: N(PhiX_free)= 152872 (-13)
 HG015-J1: N(PhiX_free)= 113674 (-4)
 HG015-J2: N(PhiX_free)= 274010 (-6)
 HG015-J3: N(PhiX_free)= 273721 (-5)
 HG015-L1: N(PhiX_free)= 425471 (-11)
 HG015-L2: N(PhiX_free)= 288798 (-10)
 HG015-L3: N(PhiX_free)= 190507 (-4)
 HG015-O1: N(PhiX_free)= 240184 (-11)
 HG015-O2: N(PhiX_free)= 180641 (-6)
 HG015-O3: N(PhiX_free)= 232907 (-21)
 HG015-Q1: N(PhiX_free)= 249188 (-6)
 HG015-Q2: N(PhiX_free)= 156765 (-5)
 HG015-Q3: N(PhiX_free)= 322559 (-5)
 HG015-R1: N(PhiX_free)= 388432 (-12)
 HG015-R2: N(PhiX_free)= 47815 (-4)
 HG015-R3: N(PhiX_free)= 188638 (-6)
 HG033-B1: N(PhiX_free)= 296622 (-9)
 HG033-B2: N(PhiX_free)= 343220 (-5)
 HG033-B3: N(PhiX_free)= 160750 (-4)
 HG033-D1: N(PhiX_free)= 212814 (-9)
 HG033-D2: N(PhiX_free)= 307077 (-2)
 HG033-D3: N(PhiX_free)= 279065 (-8)
 HG033-I1: N(PhiX_free)= 258128 (-8)
 HG033-I2: N(PhiX_free)= 239659 (-9)
 HG033-I3: N(PhiX_free)= 764976 (-28)
 HG033-J1: N(PhiX_free)= 375315 (-11)
 HG033-J2: N(PhiX_free)= 214912 (-6)
 HG033-J3: N(PhiX_free)= 205540 (-5)
 HG033-L1: N(PhiX_free)= 261055 (-8)
 HG033-L2: N(PhiX_free)= 129671 (-2)
 HG033-L3: N(PhiX_free)= 1545390 (-30)
 HG033-O1: N(PhiX_free)= 407862 (-12)
 HG033-O2: N(PhiX_free)= 312165 (-8)
 HG033-O3: N(PhiX_free)= 168126 (-2)
 HG033-Q1: N(PhiX_free)= 235322 (-5)
 HG033-Q2: N(PhiX_free)= 187425 (-3)
 HG033-Q3: N(PhiX_free)= 170531 (-2)
 HG033-R1: N(PhiX_free)= 313004 (-17)
 HG033-R2: N(PhiX_free)= 181946 (-11)
 HG033-R3: N(PhiX_free)= 175434 (-3)
 HG059-B1: N(PhiX_free)= 312116 (-9)

```
HG059-B2: N(PhiX_free)= 293473 (-5)
HG059-B3: N(PhiX_free)= 178001 (-1)
HG059-D1: N(PhiX_free)= 381369 (-9)
HG059-D2: N(PhiX_free)= 427496 (-10)
HG059-D3: N(PhiX_free)= 362243 (-3)
HG059-I1: N(PhiX_free)= 583611 (-15)
HG059-I2: N(PhiX_free)= 179484 (-5)
HG059-I3: N(PhiX_free)= 211435 (-3)
HG059-J1: N(PhiX_free)= 276309 (-5)
HG059-J2: N(PhiX_free)= 309567 (-9)
HG059-J3: N(PhiX_free)= 1035294 (-21)
HG059-L1: N(PhiX_free)= 175580 (-6)
HG059-L2: N(PhiX_free)= 315005 (-8)
HG059-L3: N(PhiX_free)= 245625 (-52)
HG059-O1: N(PhiX_free)= 394279 (-3)
HG059-O2: N(PhiX_free)= 324085 (-7)
HG059-O3: N(PhiX_free)= 139654 (-2)
HG059-Q1: N(PhiX_free)= 613205 (-22)
HG059-Q2: N(PhiX_free)= 148920 (-4)
HG059-Q3: N(PhiX_free)= 177412 (-3)
HG059-R1: N(PhiX_free)= 345365 (-10)
HG059-R2: N(PhiX_free)= 211002 (-4)
HG059-R3: N(PhiX_free)= 268159 (-3)
HG082-B1: N(PhiX_free)= 240026 (-4)
HG082-B2: N(PhiX_free)= 437360 (-7)
HG082-B3: N(PhiX_free)= 189896 (-4)
HG082-D1: N(PhiX_free)= 206698 (-2)
HG082-D2: N(PhiX_free)= 249285 (-4)
HG082-D3: N(PhiX_free)= 191613 (-6)
HG082-I1: N(PhiX_free)= 152331 (-3)
HG082-I2: N(PhiX_free)= 265045 (-3)
HG082-I3: N(PhiX_free)= 303790 (-4)
HG082-J1: N(PhiX_free)= 261045 (-3)
HG082-J2: N(PhiX_free)= 237293 (-12)
HG082-J3: N(PhiX_free)= 161564 (-2)
HG082-L1: N(PhiX_free)= 280477 (-7)
HG082-L2: N(PhiX_free)= 703755 (-23)
HG082-L3: N(PhiX_free)= 140477 (-2)
HG082-O1: N(PhiX_free)= 208384 (-5)
HG082-O2: N(PhiX_free)= 175377 (-7)
HG082-O3: N(PhiX_free)= 308661 (-7)
HG082-Q1: N(PhiX_free)= 219109 (-10)
HG082-Q2: N(PhiX_free)= 161784 (-20)
HG082-Q3: N(PhiX_free)= 282241 (-5)
HG082-R1: N(PhiX_free)= 336139 (-3)
HG082-R2: N(PhiX_free)= 185457 (-2)
HG082-R3: N(PhiX_free)= 414345 (-6)
HG091-B1: N(PhiX_free)= 36476 (-16)
HG091-B2: N(PhiX_free)= 174737 (-4)
HG091-B3: N(PhiX_free)= 305832 (-17)
HG091-D1: N(PhiX_free)= 73347 (-45)
HG091-D2: N(PhiX_free)= 212235 (-12)
HG091-D3: N(PhiX_free)= 96928 (-2)
HG091-I1: N(PhiX_free)= 262684 (-6)
```

HG091-I2: N(PhiX_free)= 306011 (-9)
HG091-I3: N(PhiX_free)= 166510 (-4)
HG091-J1: N(PhiX_free)= 17763 (-6)
HG091-J2: N(PhiX_free)= 269285 (-5)
HG091-J3: N(PhiX_free)= 162559 (-3)
HG091-L1: N(PhiX_free)= 30504 (-27)
HG091-L2: N(PhiX_free)= 289620 (-9)
HG091-L3: N(PhiX_free)= 134829 (-1)
HG091-O1: N(PhiX_free)= 417370 (-9)
HG091-O2: N(PhiX_free)= 116783 (-6)
HG091-O3: N(PhiX_free)= 182870 (-3)
HG091-Q1: N(PhiX_free)= 259622 (-12)
HG091-Q2: N(PhiX_free)= 172329 (0)
HG091-Q3: N(PhiX_free)= 432484 (-14)
HG091-R1: N(PhiX_free)= 214747 (-12)
HG091-R2: N(PhiX_free)= 172206 (-9)
HG091-R3: N(PhiX_free)= 244822 (-7)
HG141-B1: N(PhiX_free)= 154521 (-41)
HG141-B2: N(PhiX_free)= 263815 (-2)
HG141-B3: N(PhiX_free)= 326045 (-3)
HG141-D1: N(PhiX_free)= 56601 (-5)
HG141-D2: N(PhiX_free)= 393449 (-13)
HG141-D3: N(PhiX_free)= 251107 (0)
HG141-I1: N(PhiX_free)= 134140 (-17)
HG141-I2: N(PhiX_free)= 261786 (-7)
HG141-I3: N(PhiX_free)= 173765 (-6)
HG141-J1: N(PhiX_free)= 184838 (-14)
HG141-J2: N(PhiX_free)= 358961 (-3)
HG141-J3: N(PhiX_free)= 289182 (-25)
HG141-L1: N(PhiX_free)= 254047 (-9)
HG141-L2: N(PhiX_free)= 296478 (-5)
HG141-L3: N(PhiX_free)= 162736 (0)
HG141-O1: N(PhiX_free)= 652841 (-16)
HG141-O2: N(PhiX_free)= 254313 (-4)
HG141-O3: N(PhiX_free)= 224305 (-3)
HG141-Q1: N(PhiX_free)= 206390 (-2)
HG141-Q2: N(PhiX_free)= 145669 (-2)
HG141-Q3: N(PhiX_free)= 381505 (-9)
HG141-R1: N(PhiX_free)= 275726 (-8)
HG141-R2: N(PhiX_free)= 114628 (-4)
HG141-R3: N(PhiX_free)= 250212 (-21)
HG225-B1: N(PhiX_free)= 91002 (-34)
HG225-B2: N(PhiX_free)= 325066 (-6)
HG225-B3: N(PhiX_free)= 258914 (-2)
HG225-D1: N(PhiX_free)= 68573 (-18)
HG225-D2: N(PhiX_free)= 370042 (-30)
HG225-D3: N(PhiX_free)= 208821 (-2)
HG225-I1: N(PhiX_free)= 80742 (-4)
HG225-I2: N(PhiX_free)= 254693 (-7)
HG225-I3: N(PhiX_free)= 196791 (-6)
HG225-J1: N(PhiX_free)= 50025 (-26)
HG225-J2: N(PhiX_free)= 396779 (-12)
HG225-J3: N(PhiX_free)= 222743 (-4)
HG225-L1: N(PhiX_free)= 348537 (-13)

```
  HG225-L2: N(PhiX_free)= 328671 (-9)
  HG225-L3: N(PhiX_free)= 172491 (-6)
  HG225-O1: N(PhiX_free)= 289499 (-12)
  HG225-O2: N(PhiX_free)= 104994 (-15)
  HG225-O3: N(PhiX_free)= 159952 (-25)
  HG225-Q1: N(PhiX_free)= 226636 (-3)
  HG225-Q2: N(PhiX_free)= 188118 (-9)
  HG225-Q3: N(PhiX_free)= 114550 (-5)
  HG225-R1: N(PhiX_free)= 201433 (-2)
  HG225-R2: N(PhiX_free)= 308514 (-4)
  HG225-R3: N(PhiX_free)= 41318 (-8)
  HG319-B1: N(PhiX_free)= 138771 (-12)
  HG319-B2: N(PhiX_free)= 217641 (-3)
  HG319-B3: N(PhiX_free)= 332202 (-7)
  HG319-D1: N(PhiX_free)= 102774 (-23)
  HG319-D2: N(PhiX_free)= 331918 (-8)
  HG319-D3: N(PhiX_free)= 224961 (-29)
  HG319-I1: N(PhiX_free)= 308834 (-1)
  HG319-I2: N(PhiX_free)= 246931 (-15)
  HG319-I3: N(PhiX_free)= 262147 (-5)
  HG319-J1: N(PhiX_free)= 247677 (-7)
  HG319-J2: N(PhiX_free)= 398859 (-9)
  HG319-J3: N(PhiX_free)= 164013 (-7)
  HG319-L1: N(PhiX_free)= 357268 (-10)
  HG319-L2: N(PhiX_free)= 473565 (-7)
  HG319-L3: N(PhiX_free)= 211978 (-2)
  HG319-O1: N(PhiX_free)= 285886 (-14)
  HG319-O2: N(PhiX_free)= 173316 (-5)
  HG319-O3: N(PhiX_free)= 145922 (-3)
  HG319-Q2: N(PhiX_free)= 169820 (-3)
  HG319-Q3: N(PhiX_free)= 92848 (-5)
  HG319-R1: N(PhiX_free)= 258557 (-8)
  HG319-R2: N(PhiX_free)= 204155 (-3)
  HG319-R3: N(PhiX_free)= 64983 (-6)
  HGnegA: N(PhiX_free)= 12497 (-5)
 End_B4_RemovePhiX: 16:25:07 07/01/2021
..................................................................
............
 Start_B5_RemoveLowComplexity: 16:25:07 07/01/2021
  HG015-B1: N(NoLowComplexity)= 251561 (-656)
  HG015-B3: N(NoLowComplexity)= 197005 (-1876)
  HG015-D1: N(NoLowComplexity)= 220650 (-874)
  HG015-D2: N(NoLowComplexity)= 307115 (-725)
  HG015-D3: N(NoLowComplexity)= 173068 (-1007)
  HG015-I1: N(NoLowComplexity)= 118454 (-1800)
  HG015-I2: N(NoLowComplexity)= 189778 (-481)
  HG015-I3: N(NoLowComplexity)= 152448 (-424)
  HG015-J1: N(NoLowComplexity)= 113104 (-570)
  HG015-J2: N(NoLowComplexity)= 272773 (-1237)
  HG015-J3: N(NoLowComplexity)= 272238 (-1483)
  HG015-L1: N(NoLowComplexity)= 424604 (-867)
  HG015-L2: N(NoLowComplexity)= 287343 (-1455)
  HG015-L3: N(NoLowComplexity)= 189202 (-1305)
  HG015-O1: N(NoLowComplexity)= 239601 (-583)
```

```
HG015-O2: N(NoLowComplexity)= 179010 (-1631)
HG015-O3: N(NoLowComplexity)= 232107 (-800)
HG015-Q1: N(NoLowComplexity)= 248775 (-413)
HG015-Q2: N(NoLowComplexity)= 156016 (-749)
HG015-Q3: N(NoLowComplexity)= 321584 (-975)
HG015-R1: N(NoLowComplexity)= 387558 (-874)
HG015-R2: N(NoLowComplexity)= 46544 (-1271)
HG015-R3: N(NoLowComplexity)= 188242 (-396)
HG033-B1: N(NoLowComplexity)= 295727 (-895)
HG033-B2: N(NoLowComplexity)= 342417 (-803)
HG033-B3: N(NoLowComplexity)= 160171 (-579)
HG033-D1: N(NoLowComplexity)= 212381 (-433)
HG033-D2: N(NoLowComplexity)= 306522 (-555)
HG033-D3: N(NoLowComplexity)= 277562 (-1503)
HG033-I1: N(NoLowComplexity)= 255665 (-2463)
HG033-I2: N(NoLowComplexity)= 239021 (-638)
HG033-I3: N(NoLowComplexity)= 758462 (-6514)
HG033-J1: N(NoLowComplexity)= 374646 (-669)
HG033-J2: N(NoLowComplexity)= 213409 (-1503)
HG033-J3: N(NoLowComplexity)= 204687 (-853)
HG033-L1: N(NoLowComplexity)= 260590 (-465)
HG033-L2: N(NoLowComplexity)= 128124 (-1547)
HG033-L3: N(NoLowComplexity)= 1540254 (-5136)
HG033-O1: N(NoLowComplexity)= 406566 (-1296)
HG033-O2: N(NoLowComplexity)= 311255 (-910)
HG033-O3: N(NoLowComplexity)= 167599 (-527)
HG033-Q1: N(NoLowComplexity)= 232585 (-2737)
HG033-Q2: N(NoLowComplexity)= 186093 (-1332)
HG033-Q3: N(NoLowComplexity)= 169903 (-628)
HG033-R1: N(NoLowComplexity)= 311235 (-1769)
HG033-R2: N(NoLowComplexity)= 180865 (-1081)
HG033-R3: N(NoLowComplexity)= 173630 (-1804)
HG059-B1: N(NoLowComplexity)= 310822 (-1294)
HG059-B2: N(NoLowComplexity)= 292918 (-555)
HG059-B3: N(NoLowComplexity)= 177485 (-516)
HG059-D1: N(NoLowComplexity)= 380226 (-1143)
HG059-D2: N(NoLowComplexity)= 426597 (-899)
HG059-D3: N(NoLowComplexity)= 361303 (-940)
HG059-I1: N(NoLowComplexity)= 582330 (-1281)
HG059-I2: N(NoLowComplexity)= 179036 (-448)
HG059-I3: N(NoLowComplexity)= 210754 (-681)
HG059-J1: N(NoLowComplexity)= 275628 (-681)
HG059-J2: N(NoLowComplexity)= 308187 (-1380)
HG059-J3: N(NoLowComplexity)= 1032240 (-3054)
HG059-L1: N(NoLowComplexity)= 175188 (-392)
HG059-L2: N(NoLowComplexity)= 314106 (-899)
HG059-L3: N(NoLowComplexity)= 244041 (-1584)
HG059-O1: N(NoLowComplexity)= 393515 (-764)
HG059-O2: N(NoLowComplexity)= 323238 (-847)
HG059-O3: N(NoLowComplexity)= 139387 (-267)
HG059-Q1: N(NoLowComplexity)= 611406 (-1799)
HG059-Q2: N(NoLowComplexity)= 148228 (-692)
HG059-Q3: N(NoLowComplexity)= 176989 (-423)
HG059-R1: N(NoLowComplexity)= 344576 (-789)
```

HG059-R2: N(NoLowComplexity)= 210474 (-528)
HG059-R3: N(NoLowComplexity)= 267428 (-731)
HG082-B1: N(NoLowComplexity)= 239523 (-503)
HG082-B2: N(NoLowComplexity)= 435962 (-1398)
HG082-B3: N(NoLowComplexity)= 189287 (-609)
HG082-D1: N(NoLowComplexity)= 205870 (-828)
HG082-D2: N(NoLowComplexity)= 248475 (-810)
HG082-D3: N(NoLowComplexity)= 191068 (-545)
HG082-I1: N(NoLowComplexity)= 150867 (-1464)
HG082-I2: N(NoLowComplexity)= 264554 (-491)
HG082-I3: N(NoLowComplexity)= 302867 (-923)
HG082-J1: N(NoLowComplexity)= 260631 (-414)
HG082-J2: N(NoLowComplexity)= 236741 (-552)
HG082-J3: N(NoLowComplexity)= 161152 (-412)
HG082-L1: N(NoLowComplexity)= 280066 (-411)
HG082-L2: N(NoLowComplexity)= 701688 (-2067)
HG082-L3: N(NoLowComplexity)= 140094 (-383)
HG082-O1: N(NoLowComplexity)= 207693 (-691)
HG082-O2: N(NoLowComplexity)= 173112 (-2265)
HG082-O3: N(NoLowComplexity)= 307859 (-802)
HG082-Q1: N(NoLowComplexity)= 217905 (-1204)
HG082-Q2: N(NoLowComplexity)= 161127 (-657)
HG082-Q3: N(NoLowComplexity)= 281483 (-758)
HG082-R1: N(NoLowComplexity)= 335367 (-772)
HG082-R2: N(NoLowComplexity)= 184983 (-474)
HG082-R3: N(NoLowComplexity)= 412642 (-1703)
HG091-B1: N(NoLowComplexity)= 29399 (-7077)
HG091-B2: N(NoLowComplexity)= 172447 (-2290)
HG091-B3: N(NoLowComplexity)= 304641 (-1191)
HG091-D1: N(NoLowComplexity)= 54730 (-18617)
HG091-D2: N(NoLowComplexity)= 211220 (-1015)
HG091-D3: N(NoLowComplexity)= 95738 (-1190)
HG091-I1: N(NoLowComplexity)= 262085 (-599)
HG091-I2: N(NoLowComplexity)= 305115 (-896)
HG091-I3: N(NoLowComplexity)= 165879 (-631)
HG091-J1: N(NoLowComplexity)= 11800 (-5963)
HG091-J2: N(NoLowComplexity)= 268044 (-1241)
HG091-J3: N(NoLowComplexity)= 160633 (-1926)
HG091-L1: N(NoLowComplexity)= 19722 (-10782)
HG091-L2: N(NoLowComplexity)= 288763 (-857)
HG091-L3: N(NoLowComplexity)= 134005 (-824)
HG091-O1: N(NoLowComplexity)= 415995 (-1375)
HG091-O2: N(NoLowComplexity)= 114980 (-1803)
HG091-O3: N(NoLowComplexity)= 182181 (-689)
HG091-Q1: N(NoLowComplexity)= 258594 (-1028)
HG091-Q2: N(NoLowComplexity)= 171874 (-455)
HG091-Q3: N(NoLowComplexity)= 429770 (-2714)
HG091-R1: N(NoLowComplexity)= 211326 (-3421)
HG091-R2: N(NoLowComplexity)= 170820 (-1386)
HG091-R3: N(NoLowComplexity)= 242637 (-2185)
HG141-B1: N(NoLowComplexity)= 136567 (-17954)
HG141-B2: N(NoLowComplexity)= 263020 (-795)
HG141-B3: N(NoLowComplexity)= 324994 (-1051)
HG141-D1: N(NoLowComplexity)= 53344 (-3257)

```
HG141-D2: N(NoLowComplexity)= 392148 (-1301)
HG141-D3: N(NoLowComplexity)= 250505 (-602)
HG141-I1: N(NoLowComplexity)= 129064 (-5076)
HG141-I2: N(NoLowComplexity)= 261250 (-536)
HG141-I3: N(NoLowComplexity)= 173244 (-521)
HG141-J1: N(NoLowComplexity)= 181864 (-2974)
HG141-J2: N(NoLowComplexity)= 357698 (-1263)
HG141-J3: N(NoLowComplexity)= 286418 (-2764)
HG141-L1: N(NoLowComplexity)= 250975 (-3072)
HG141-L2: N(NoLowComplexity)= 295855 (-623)
HG141-L3: N(NoLowComplexity)= 162190 (-546)
HG141-O1: N(NoLowComplexity)= 650971 (-1870)
HG141-O2: N(NoLowComplexity)= 253490 (-823)
HG141-O3: N(NoLowComplexity)= 223762 (-543)
HG141-Q1: N(NoLowComplexity)= 205873 (-517)
HG141-Q2: N(NoLowComplexity)= 145171 (-498)
HG141-Q3: N(NoLowComplexity)= 380246 (-1259)
HG141-R1: N(NoLowComplexity)= 274981 (-745)
HG141-R2: N(NoLowComplexity)= 113054 (-1574)
HG141-R3: N(NoLowComplexity)= 249195 (-1017)
HG225-B1: N(NoLowComplexity)= 69713 (-21289)
HG225-B2: N(NoLowComplexity)= 324121 (-945)
HG225-B3: N(NoLowComplexity)= 258166 (-748)
HG225-D1: N(NoLowComplexity)= 61773 (-6800)
HG225-D2: N(NoLowComplexity)= 368638 (-1404)
HG225-D3: N(NoLowComplexity)= 208008 (-813)
HG225-I1: N(NoLowComplexity)= 74073 (-6669)
HG225-I2: N(NoLowComplexity)= 253435 (-1258)
HG225-I3: N(NoLowComplexity)= 196213 (-578)
HG225-J1: N(NoLowComplexity)= 38949 (-11076)
HG225-J2: N(NoLowComplexity)= 394992 (-1787)
HG225-J3: N(NoLowComplexity)= 221435 (-1308)
HG225-L1: N(NoLowComplexity)= 345492 (-3045)
HG225-L2: N(NoLowComplexity)= 327885 (-786)
HG225-L3: N(NoLowComplexity)= 171937 (-554)
HG225-O1: N(NoLowComplexity)= 287476 (-2023)
HG225-O2: N(NoLowComplexity)= 102484 (-2510)
HG225-O3: N(NoLowComplexity)= 159275 (-677)
HG225-Q1: N(NoLowComplexity)= 226044 (-592)
HG225-Q2: N(NoLowComplexity)= 186268 (-1850)
HG225-Q3: N(NoLowComplexity)= 110826 (-3724)
HG225-R1: N(NoLowComplexity)= 200820 (-613)
HG225-R2: N(NoLowComplexity)= 307230 (-1284)
HG225-R3: N(NoLowComplexity)= 35836 (-5482)
HG319-B1: N(NoLowComplexity)= 130552 (-8219)
HG319-B2: N(NoLowComplexity)= 216782 (-859)
HG319-B3: N(NoLowComplexity)= 331297 (-905)
HG319-D1: N(NoLowComplexity)= 96098 (-6676)
HG319-D2: N(NoLowComplexity)= 330649 (-1269)
HG319-D3: N(NoLowComplexity)= 224269 (-692)
HG319-I1: N(NoLowComplexity)= 308215 (-619)
HG319-I2: N(NoLowComplexity)= 246203 (-728)
HG319-I3: N(NoLowComplexity)= 261575 (-572)
HG319-J1: N(NoLowComplexity)= 246878 (-799)
```

```
  HG319-J2: N(NoLowComplexity)= 398232 (-627)
  HG319-J3: N(NoLowComplexity)= 163435 (-578)
  HG319-L1: N(NoLowComplexity)= 356604 (-664)
  HG319-L2: N(NoLowComplexity)= 472322 (-1243)
  HG319-L3: N(NoLowComplexity)= 211512 (-466)
  HG319-O1: N(NoLowComplexity)= 284402 (-1484)
  HG319-O2: N(NoLowComplexity)= 171165 (-2151)
  HG319-O3: N(NoLowComplexity)= 145484 (-438)
  HG319-Q2: N(NoLowComplexity)= 169222 (-598)
  HG319-Q3: N(NoLowComplexity)= 90591 (-2257)
  HG319-R1: N(NoLowComplexity)= 257162 (-1395)
  HG319-R2: N(NoLowComplexity)= 203672 (-483)
  HG319-R3: N(NoLowComplexity)= 62459 (-2524)
  HGnegA: N(NoLowComplexity)= 11091 (-1406)
 End_B5_RemoveLowComplexity: 16:39:10 07/01/2021
..................................................................
............
 Start_B6_Trim_and_Merge: 16:39:10 07/01/2021
  ‣ HG015-B1 - Merging Summary
    Merging Rate: 240977 / 251560 (95.8%)
    Median Merged Length: 472

    251560  Pairs (251.6k)
    240977  Merged (241.0k, 95.79%)
    171916  Alignments with zero diffs (68.34%)
       485  Too many diffs (> 10) (0.19%)
         1  Fwd too short (< 64) after tail trimming (0.00%)
         2  Rev too short (< 64) after tail trimming (0.00%)
     10086  No alignment found (4.01%)
         9  Alignment too short (< 20) (0.00%)
         0  Merged too short (< 100)
         0  Min Q too low (<0) (0.00%)
        37  Staggered pairs (0.01%) merged & trimmed
     31.63  Mean alignment length
    470.06  Mean merged length
      0.55  Mean fwd expected errors
      0.69  Mean rev expected errors
      0.80  Mean merged expected errors
-----
  ‣ HG015-B3 - Merging Summary
    Merging Rate: 139361 / 196999 (70.7%)
    Median Merged Length: 449

    196999  Pairs (197.0k)
    139361  Merged (139.4k, 70.74%)
     40836  Alignments with zero diffs (20.73%)
     19977  Too many diffs (> 10) (10.14%)
        10  Fwd too short (< 64) after tail trimming (0.01%)
       190  Rev too short (< 64) after tail trimming (0.10%)
     37454  No alignment found (19.01%)
         5  Alignment too short (< 20) (0.00%)
         2  Merged too short (< 100)
         0  Min Q too low (<0) (0.00%)
      3519  Staggered pairs (1.79%) merged & trimmed
```

```
     93.92  Mean alignment length
    382.31  Mean merged length
      1.55  Mean fwd expected errors
      1.64  Mean rev expected errors
      1.04  Mean merged expected errors
-----
  ‣ HG015-D1 - Merging Summary
    Merging Rate: 204950 / 220649 (92.9%)
    Median Merged Length: 472

    220649  Pairs (220.6k)
    204950  Merged (204.9k, 92.89%)
    144660  Alignments with zero diffs (65.56%)
      1575  Too many diffs (> 10) (0.71%)
         5  Fwd too short (< 64) after tail trimming (0.00%)
        56  Rev too short (< 64) after tail trimming (0.03%)
     14056  No alignment found (6.37%)
         7  Alignment too short (< 20) (0.00%)
         0  Merged too short (< 100)
         0  Min Q too low (<0) (0.00%)
       325  Staggered pairs (0.15%) merged & trimmed
     38.28  Mean alignment length
    460.46  Mean merged length
      0.55  Mean fwd expected errors
      0.68  Mean rev expected errors
      0.75  Mean merged expected errors
-----
  ‣ HG015-D2 - Merging Summary
    Merging Rate: 294777 / 307114 (96.0%)
    Median Merged Length: 472

    307114  Pairs (307.1k)
    294777  Merged (294.8k, 95.98%)
    183042  Alignments with zero diffs (59.60%)
      3623  Too many diffs (> 10) (1.18%)
         1  Fwd too short (< 64) after tail trimming (0.00%)
         7  Rev too short (< 64) after tail trimming (0.00%)
      8705  No alignment found (2.83%)
         1  Alignment too short (< 20) (0.00%)
         0  Merged too short (< 100)
         0  Min Q too low (<0) (0.00%)
       101  Staggered pairs (0.03%) merged & trimmed
     38.22  Mean alignment length
    462.60  Mean merged length
      0.63  Mean fwd expected errors
      0.88  Mean rev expected errors
      0.83  Mean merged expected errors
-----
  ‣ HG015-D3 - Merging Summary
    Merging Rate: 150899 / 173068 (87.2%)
    Median Merged Length: 472

    173068  Pairs (173.1k)
    150899  Merged (150.9k, 87.19%)
```

```
     66830  Alignments with zero diffs (38.61%)
      3748  Too many diffs (> 10) (2.17%)
         2  Fwd too short (< 64) after tail trimming (0.00%)
        14  Rev too short (< 64) after tail trimming (0.01%)
     18264  No alignment found (10.55%)
       141  Alignment too short (< 20) (0.08%)
         0  Merged too short (< 100)
         0  Min Q too low (<0) (0.00%)
       327  Staggered pairs (0.19%) merged & trimmed
     45.54  Mean alignment length
    453.77  Mean merged length
      1.02  Mean fwd expected errors
      1.15  Mean rev expected errors
      1.09  Mean merged expected errors
-----
  ‣ HG015-I1 - Merging Summary
    Merging Rate: 99848 / 118390 (84.3%)
    Median Merged Length: 449

    118390  Pairs (118.4k)
     99848  Merged (99.8k, 84.34%)
     51474  Alignments with zero diffs (43.48%)
      7336  Too many diffs (> 10) (6.20%)
        48  Fwd too short (< 64) after tail trimming (0.04%)
       447  Rev too short (< 64) after tail trimming (0.38%)
     10699  No alignment found (9.04%)
         9  Alignment too short (< 20) (0.01%)
         3  Merged too short (< 100)
         0  Min Q too low (<0) (0.00%)
       568  Staggered pairs (0.48%) merged & trimmed
     98.80  Mean alignment length
    391.27  Mean merged length
      0.56  Mean fwd expected errors
      1.06  Mean rev expected errors
      0.54  Mean merged expected errors
-----
  ‣ HG015-I2 - Merging Summary
    Merging Rate: 182941 / 189772 (96.4%)
    Median Merged Length: 472

    189772  Pairs (189.8k)
    182941  Merged (182.9k, 96.40%)
    127586  Alignments with zero diffs (67.23%)
       575  Too many diffs (> 10) (0.30%)
         2  Fwd too short (< 64) after tail trimming (0.00%)
        23  Rev too short (< 64) after tail trimming (0.01%)
      6223  No alignment found (3.28%)
         7  Alignment too short (< 20) (0.00%)
         1  Merged too short (< 100)
         0  Min Q too low (<0) (0.00%)
        45  Staggered pairs (0.02%) merged & trimmed
     32.61  Mean alignment length
    468.91  Mean merged length
      0.55  Mean fwd expected errors
```

```
      0.74  Mean rev expected errors
      0.80  Mean merged expected errors
-----
  ‣ HG015-I3 - Merging Summary
    Merging Rate: 145382 / 152447 (95.4%)
    Median Merged Length: 472

    152447  Pairs (152.4k)
    145382  Merged (145.4k, 95.37%)
     65481  Alignments with zero diffs (42.95%)
       533  Too many diffs (> 10) (0.35%)
         1  Fwd too short (< 64) after tail trimming (0.00%)
         7  Rev too short (< 64) after tail trimming (0.00%)
      6524  No alignment found (4.28%)
         0  Alignment too short (< 20) (0.00%)
         0  Merged too short (< 100)
         0  Min Q too low (<0) (0.00%)
        67  Staggered pairs (0.04%) merged & trimmed
     31.28  Mean alignment length
    470.09  Mean merged length
      1.05  Mean fwd expected errors
      1.14  Mean rev expected errors
      1.19  Mean merged expected errors
-----
  ‣ HG015-J1 - Merging Summary
    Merging Rate: 102813 / 113098 (90.9%)
    Median Merged Length: 472

    113098  Pairs (113.1k)
    102813  Merged (102.8k, 90.91%)
     63183  Alignments with zero diffs (55.87%)
      2666  Too many diffs (> 10) (2.36%)
        14  Fwd too short (< 64) after tail trimming (0.01%)
       116  Rev too short (< 64) after tail trimming (0.10%)
      7475  No alignment found (6.61%)
        13  Alignment too short (< 20) (0.01%)
         1  Merged too short (< 100)
         0  Min Q too low (<0) (0.00%)
       182  Staggered pairs (0.16%) merged & trimmed
     48.36  Mean alignment length
    449.65  Mean merged length
      0.70  Mean fwd expected errors
      1.00  Mean rev expected errors
      0.90  Mean merged expected errors
-----
  ‣ HG015-J2 - Merging Summary
    Merging Rate: 255837 / 272752 (93.8%)
    Median Merged Length: 472

    272752  Pairs (272.8k)
    255837  Merged (255.8k, 93.80%)
    178497  Alignments with zero diffs (65.44%)
      2000  Too many diffs (> 10) (0.73%)
        11  Fwd too short (< 64) after tail trimming (0.00%)
```

```
        96  Rev too short (< 64) after tail trimming (0.04%)
     14796  No alignment found (5.42%)
        10  Alignment too short (< 20) (0.00%)
         2  Merged too short (< 100)
         0  Min Q too low (<0) (0.00%)
       241  Staggered pairs (0.09%) merged & trimmed
     35.96  Mean alignment length
    463.19  Mean merged length
      0.62  Mean fwd expected errors
      0.70  Mean rev expected errors
      0.79  Mean merged expected errors
-----
  ‣ HG015-J3 - Merging Summary
    Merging Rate: 249792 / 272233 (91.8%)
    Median Merged Length: 449

    272233  Pairs (272.2k)
    249792  Merged (249.8k, 91.76%)
     71435  Alignments with zero diffs (26.24%)
     10816  Too many diffs (> 10) (3.97%)
         7  Fwd too short (< 64) after tail trimming (0.00%)
        49  Rev too short (< 64) after tail trimming (0.02%)
     11562  No alignment found (4.25%)
         7  Alignment too short (< 20) (0.00%)
         0  Merged too short (< 100)
         0  Min Q too low (<0) (0.00%)
       154  Staggered pairs (0.06%) merged & trimmed
     53.43  Mean alignment length
    447.61  Mean merged length
      1.03  Mean fwd expected errors
      1.52  Mean rev expected errors
      0.96  Mean merged expected errors
-----
  ‣ HG015-L1 - Merging Summary
    Merging Rate: 409392 / 424601 (96.4%)
    Median Merged Length: 472

    424601  Pairs (424.6k)
    409392  Merged (409.4k, 96.42%)
    286888  Alignments with zero diffs (67.57%)
      1616  Too many diffs (> 10) (0.38%)
         1  Fwd too short (< 64) after tail trimming (0.00%)
        10  Rev too short (< 64) after tail trimming (0.00%)
     13576  No alignment found (3.20%)
         6  Alignment too short (< 20) (0.00%)
         0  Merged too short (< 100)
         0  Min Q too low (<0) (0.00%)
       140  Staggered pairs (0.03%) merged & trimmed
     33.95  Mean alignment length
    467.30  Mean merged length
      0.53  Mean fwd expected errors
      0.74  Mean rev expected errors
      0.79  Mean merged expected errors
-----
```

```
▸ HG015-L2 - Merging Summary
  Merging Rate: 266521 / 287325 (92.8%)
  Median Merged Length: 472

  287325  Pairs (287.3k)
  266521  Merged (266.5k, 92.76%)
  183138  Alignments with zero diffs (63.74%)
    2814  Too many diffs (> 10) (0.98%)
      11  Fwd too short (< 64) after tail trimming (0.00%)
     142  Rev too short (< 64) after tail trimming (0.05%)
   17826  No alignment found (6.20%)
      10  Alignment too short (< 20) (0.00%)
       1  Merged too short (< 100)
       0  Min Q too low (<0) (0.00%)
     567  Staggered pairs (0.20%) merged & trimmed
   39.53  Mean alignment length
  458.38  Mean merged length
    0.56  Mean fwd expected errors
    0.77  Mean rev expected errors
    0.79  Mean merged expected errors
-----
▸ HG015-L3 - Merging Summary
  Merging Rate: 172388 / 189183 (91.1%)
  Median Merged Length: 453

  189183  Pairs (189.2k)
  172388  Merged (172.4k, 91.12%)
   63125  Alignments with zero diffs (33.37%)
    5257  Too many diffs (> 10) (2.78%)
      37  Fwd too short (< 64) after tail trimming (0.02%)
     161  Rev too short (< 64) after tail trimming (0.09%)
   11334  No alignment found (5.99%)
       4  Alignment too short (< 20) (0.00%)
       2  Merged too short (< 100)
       0  Min Q too low (<0) (0.00%)
     248  Staggered pairs (0.13%) merged & trimmed
   45.64  Mean alignment length
  453.99  Mean merged length
    1.11  Mean fwd expected errors
    1.35  Mean rev expected errors
    1.06  Mean merged expected errors
-----
▸ HG015-O1 - Merging Summary
  Merging Rate: 226687 / 239596 (94.6%)
  Median Merged Length: 472

  239596  Pairs (239.6k)
  226687  Merged (226.7k, 94.61%)
  149823  Alignments with zero diffs (62.53%)
    2062  Too many diffs (> 10) (0.86%)
       7  Fwd too short (< 64) after tail trimming (0.00%)
      60  Rev too short (< 64) after tail trimming (0.03%)
   10763  No alignment found (4.49%)
      17  Alignment too short (< 20) (0.01%)
```

```
         0  Merged too short (< 100)
         0  Min Q too low (<0) (0.00%)
       248  Staggered pairs (0.10%) merged & trimmed
     37.24  Mean alignment length
    462.65  Mean merged length
      0.53  Mean fwd expected errors
      0.89  Mean rev expected errors
      0.87  Mean merged expected errors
-----
  ▸ HG015-O2 - Merging Summary
    Merging Rate: 137466 / 179005 (76.8%)
    Median Merged Length: 449

    179005  Pairs (179.0k)
    137466  Merged (137.5k, 76.79%)
     68279  Alignments with zero diffs (38.14%)
      7968  Too many diffs (> 10) (4.45%)
         5  Fwd too short (< 64) after tail trimming (0.00%)
       116  Rev too short (< 64) after tail trimming (0.06%)
     33423  No alignment found (18.67%)
        27  Alignment too short (< 20) (0.02%)
         0  Merged too short (< 100)
         0  Min Q too low (<0) (0.00%)
       698  Staggered pairs (0.39%) merged & trimmed
     88.89  Mean alignment length
    402.19  Mean merged length
      0.70  Mean fwd expected errors
      0.96  Mean rev expected errors
      0.58  Mean merged expected errors
-----
  ▸ HG015-O3 - Merging Summary
    Merging Rate: 213442 / 232105 (92.0%)
    Median Merged Length: 472

    232105  Pairs (232.1k)
    213442  Merged (213.4k, 91.96%)
     89036  Alignments with zero diffs (38.36%)
      3164  Too many diffs (> 10) (1.36%)
         5  Fwd too short (< 64) after tail trimming (0.00%)
        18  Rev too short (< 64) after tail trimming (0.01%)
     15453  No alignment found (6.66%)
        23  Alignment too short (< 20) (0.01%)
         0  Merged too short (< 100)
         0  Min Q too low (<0) (0.00%)
       299  Staggered pairs (0.13%) merged & trimmed
     37.14  Mean alignment length
    463.15  Mean merged length
      1.05  Mean fwd expected errors
      1.28  Mean rev expected errors
      1.19  Mean merged expected errors
-----
  ▸ HG015-Q1 - Merging Summary
    Merging Rate: 242059 / 248764 (97.3%)
    Median Merged Length: 472
```

```
   248764  Pairs (248.8k)
   242059  Merged (242.1k, 97.30%)
   167955  Alignments with zero diffs (67.52%)
      251  Too many diffs (> 10) (0.10%)
        3  Fwd too short (< 64) after tail trimming (0.00%)
      219  Rev too short (< 64) after tail trimming (0.09%)
     6232  No alignment found (2.51%)
        0  Alignment too short (< 20) (0.00%)
        0  Merged too short (< 100)
        0  Min Q too low (<0) (0.00%)
      124  Staggered pairs (0.05%) merged & trimmed
    30.44  Mean alignment length
   470.54  Mean merged length
     0.55  Mean fwd expected errors
     0.79  Mean rev expected errors
     0.86  Mean merged expected errors
-----
 ▸ HG015-Q2 - Merging Summary
   Merging Rate: 147031 / 155931 (94.3%)
   Median Merged Length: 472

   155931  Pairs (155.9k)
   147031  Merged (147.0k, 94.29%)
    70214  Alignments with zero diffs (45.03%)
      856  Too many diffs (> 10) (0.55%)
       24  Fwd too short (< 64) after tail trimming (0.02%)
      275  Rev too short (< 64) after tail trimming (0.18%)
     7739  No alignment found (4.96%)
        2  Alignment too short (< 20) (0.00%)
        4  Merged too short (< 100)
        0  Min Q too low (<0) (0.00%)
      224  Staggered pairs (0.14%) merged & trimmed
    32.37  Mean alignment length
   466.45  Mean merged length
     1.11  Mean fwd expected errors
     1.08  Mean rev expected errors
     1.16  Mean merged expected errors
-----
 ▸ HG015-Q3 - Merging Summary
   Merging Rate: 301223 / 321584 (93.7%)
   Median Merged Length: 451

   321584  Pairs (321.6k)
   301223  Merged (301.2k, 93.67%)
   108967  Alignments with zero diffs (33.88%)
     8510  Too many diffs (> 10) (2.65%)
        0  Fwd too short (< 64) after tail trimming (0.00%)
        2  Rev too short (< 64) after tail trimming (0.00%)
    11848  No alignment found (3.68%)
        1  Alignment too short (< 20) (0.00%)
        0  Merged too short (< 100)
        0  Min Q too low (<0) (0.00%)
       20  Staggered pairs (0.01%) merged & trimmed
```

```
     43.79  Mean alignment length
    457.44  Mean merged length
      1.12  Mean fwd expected errors
      1.34  Mean rev expected errors
      1.06  Mean merged expected errors
-----
  ▸ HG015-R1 - Merging Summary
    Merging Rate: 376108 / 387551 (97.0%)
    Median Merged Length: 472

    387551  Pairs (387.6k)
    376108  Merged (376.1k, 97.05%)
    262701  Alignments with zero diffs (67.78%)
      1142  Too many diffs (> 10) (0.29%)
         5  Fwd too short (< 64) after tail trimming (0.00%)
       142  Rev too short (< 64) after tail trimming (0.04%)
     10149  No alignment found (2.62%)
         5  Alignment too short (< 20) (0.00%)
         0  Merged too short (< 100)
         0  Min Q too low (<0) (0.00%)
       318  Staggered pairs (0.08%) merged & trimmed
     32.57  Mean alignment length
    467.98  Mean merged length
      0.53  Mean fwd expected errors
      0.73  Mean rev expected errors
      0.79  Mean merged expected errors
-----
  ▸ HG015-R2 - Merging Summary
    Merging Rate: 26769 / 46487 (57.6%)
    Median Merged Length: 437

     46487  Pairs (46.5k)
     26769  Merged (26.8k, 57.58%)
      5703  Alignments with zero diffs (12.27%)
      8518  Too many diffs (> 10) (18.32%)
        75  Fwd too short (< 64) after tail trimming (0.16%)
       304  Rev too short (< 64) after tail trimming (0.65%)
     10813  No alignment found (23.26%)
         3  Alignment too short (< 20) (0.01%)
         5  Merged too short (< 100)
         0  Min Q too low (<0) (0.00%)
      1432  Staggered pairs (3.08%) merged & trimmed
     97.33  Mean alignment length
    370.91  Mean merged length
      1.25  Mean fwd expected errors
      2.53  Mean rev expected errors
      1.11  Mean merged expected errors
-----
  ▸ HG015-R3 - Merging Summary
    Merging Rate: 178194 / 188241 (94.7%)
    Median Merged Length: 472

    188241  Pairs (188.2k)
    178194  Merged (178.2k, 94.66%)
```

```
     78209  Alignments with zero diffs (41.55%)
      1438  Too many diffs (> 10) (0.76%)
         1  Fwd too short (< 64) after tail trimming (0.00%)
         9  Rev too short (< 64) after tail trimming (0.00%)
      8597  No alignment found (4.57%)
         2  Alignment too short (< 20) (0.00%)
         0  Merged too short (< 100)
         0  Min Q too low (<0) (0.00%)
       111  Staggered pairs (0.06%) merged & trimmed
     33.29  Mean alignment length
    467.77  Mean merged length
      1.06  Mean fwd expected errors
      1.16  Mean rev expected errors
      1.21  Mean merged expected errors
-----
  ▸ HG033-B1 - Merging Summary
    Merging Rate: 279554 / 295720 (94.5%)
    Median Merged Length: 470

    295720  Pairs (295.7k)
    279554  Merged (279.6k, 94.53%)
    163971  Alignments with zero diffs (55.45%)
      4346  Too many diffs (> 10) (1.47%)
         7  Fwd too short (< 64) after tail trimming (0.00%)
       152  Rev too short (< 64) after tail trimming (0.05%)
     11649  No alignment found (3.94%)
        10  Alignment too short (< 20) (0.00%)
         2  Merged too short (< 100)
         0  Min Q too low (<0) (0.00%)
       380  Staggered pairs (0.13%) merged & trimmed
     43.69  Mean alignment length
    455.79  Mean merged length
      0.64  Mean fwd expected errors
      0.87  Mean rev expected errors
      0.79  Mean merged expected errors
-----
  ▸ HG033-B2 - Merging Summary
    Merging Rate: 330747 / 342417 (96.6%)
    Median Merged Length: 472

    342417  Pairs (342.4k)
    330747  Merged (330.7k, 96.59%)
    240312  Alignments with zero diffs (70.18%)
       813  Too many diffs (> 10) (0.24%)
         2  Fwd too short (< 64) after tail trimming (0.00%)
        28  Rev too short (< 64) after tail trimming (0.01%)
     10812  No alignment found (3.16%)
        14  Alignment too short (< 20) (0.00%)
         1  Merged too short (< 100)
         0  Min Q too low (<0) (0.00%)
       197  Staggered pairs (0.06%) merged & trimmed
     32.57  Mean alignment length
    468.04  Mean merged length
      0.52  Mean fwd expected errors
```

```
      0.67  Mean rev expected errors
      0.76  Mean merged expected errors
-----
  ▸ HG033-B3 - Merging Summary
    Merging Rate: 149653 / 160169 (93.4%)
    Median Merged Length: 470

    160169  Pairs (160.2k)
    149653  Merged (149.7k, 93.43%)
     59448  Alignments with zero diffs (37.12%)
      3029  Too many diffs (> 10) (1.89%)
         0  Fwd too short (< 64) after tail trimming (0.00%)
         1  Rev too short (< 64) after tail trimming (0.00%)
      7483  No alignment found (4.67%)
         3  Alignment too short (< 20) (0.00%)
         0  Merged too short (< 100)
         0  Min Q too low (<0) (0.00%)
        64  Staggered pairs (0.04%) merged & trimmed
     41.78  Mean alignment length
    459.46  Mean merged length
      1.09  Mean fwd expected errors
      1.19  Mean rev expected errors
      1.05  Mean merged expected errors
-----
  ▸ HG033-D1 - Merging Summary
    Merging Rate: 205847 / 212376 (96.9%)
    Median Merged Length: 472

    212376  Pairs (212.4k)
    205847  Merged (205.8k, 96.93%)
    143073  Alignments with zero diffs (67.37%)
       537  Too many diffs (> 10) (0.25%)
        10  Fwd too short (< 64) after tail trimming (0.00%)
       123  Rev too short (< 64) after tail trimming (0.06%)
      5853  No alignment found (2.76%)
         6  Alignment too short (< 20) (0.00%)
         0  Merged too short (< 100)
         0  Min Q too low (<0) (0.00%)
       174  Staggered pairs (0.08%) merged & trimmed
     32.23  Mean alignment length
    468.24  Mean merged length
      0.56  Mean fwd expected errors
      0.76  Mean rev expected errors
      0.84  Mean merged expected errors
-----
  ▸ HG033-D2 - Merging Summary
    Merging Rate: 297394 / 306519 (97.0%)
    Median Merged Length: 472

    306519  Pairs (306.5k)
    297394  Merged (297.4k, 97.02%)
    213293  Alignments with zero diffs (69.59%)
       769  Too many diffs (> 10) (0.25%)
         1  Fwd too short (< 64) after tail trimming (0.00%)
```

```
        28  Rev too short (< 64) after tail trimming (0.01%)
      8324  No alignment found (2.72%)
         2  Alignment too short (< 20) (0.00%)
         1  Merged too short (< 100)
         0  Min Q too low (<0) (0.00%)
       117  Staggered pairs (0.04%) merged & trimmed
     31.57  Mean alignment length
    468.59  Mean merged length
      0.55  Mean fwd expected errors
      0.70  Mean rev expected errors
      0.78  Mean merged expected errors
-----
  ▸ HG033-D3 - Merging Summary
    Merging Rate: 235771 / 277555 (84.9%)
    Median Merged Length: 449

    277555  Pairs (277.6k)
    235771  Merged (235.8k, 84.95%)
     69564  Alignments with zero diffs (25.06%)
     15815  Too many diffs (> 10) (5.70%)
         1  Fwd too short (< 64) after tail trimming (0.00%)
        48  Rev too short (< 64) after tail trimming (0.02%)
     25919  No alignment found (9.34%)
         1  Alignment too short (< 20) (0.00%)
         0  Merged too short (< 100)
         0  Min Q too low (<0) (0.00%)
       926  Staggered pairs (0.33%) merged & trimmed
     64.42  Mean alignment length
    433.51  Mean merged length
      1.03  Mean fwd expected errors
      1.59  Mean rev expected errors
      0.95  Mean merged expected errors
-----
  ▸ HG033-I1 - Merging Summary
    Merging Rate: 201374 / 255655 (78.8%)
    Median Merged Length: 452

    255655  Pairs (255.7k)
    201374  Merged (201.4k, 78.77%)
    106451  Alignments with zero diffs (41.64%)
     14455  Too many diffs (> 10) (5.65%)
        16  Fwd too short (< 64) after tail trimming (0.01%)
       256  Rev too short (< 64) after tail trimming (0.10%)
     39486  No alignment found (15.45%)
        68  Alignment too short (< 20) (0.03%)
         0  Merged too short (< 100)
         0  Min Q too low (<0) (0.00%)
      1302  Staggered pairs (0.51%) merged & trimmed
     89.46  Mean alignment length
    400.96  Mean merged length
      0.60  Mean fwd expected errors
      1.02  Mean rev expected errors
      0.57  Mean merged expected errors
-----
```

```
 ‣ HG033-I2 - Merging Summary
   Merging Rate: 231141 / 239008 (96.7%)
   Median Merged Length: 472

   239008  Pairs (239.0k)
   231141  Merged (231.1k, 96.71%)
   150082  Alignments with zero diffs (62.79%)
      967  Too many diffs (> 10) (0.40%)
       11  Fwd too short (< 64) after tail trimming (0.00%)
       69  Rev too short (< 64) after tail trimming (0.03%)
     6817  No alignment found (2.85%)
        3  Alignment too short (< 20) (0.00%)
        0  Merged too short (< 100)
        0  Min Q too low (<0) (0.00%)
      185  Staggered pairs (0.08%) merged & trimmed
    33.08  Mean alignment length
   467.71  Mean merged length
     0.83  Mean fwd expected errors
     0.74  Mean rev expected errors
     0.98  Mean merged expected errors
-----
 ‣ HG033-I3 - Merging Summary
   Merging Rate: 665944 / 757937 (87.9%)
   Median Merged Length: 472

   757937  Pairs (757.9k)
   665944  Merged (665.9k, 87.86%)
   286029  Alignments with zero diffs (37.74%)
    17349  Too many diffs (> 10) (2.29%)
      602  Fwd too short (< 64) after tail trimming (0.08%)
     4846  Rev too short (< 64) after tail trimming (0.64%)
    69065  No alignment found (9.11%)
      114  Alignment too short (< 20) (0.02%)
       17  Merged too short (< 100)
        0  Min Q too low (<0) (0.00%)
     5488  Staggered pairs (0.72%) merged & trimmed
    44.90  Mean alignment length
   447.51  Mean merged length
     1.09  Mean fwd expected errors
     1.24  Mean rev expected errors
     1.12  Mean merged expected errors
-----
 ‣ HG033-J1 - Merging Summary
   Merging Rate: 365038 / 374644 (97.4%)
   Median Merged Length: 472

   374644  Pairs (374.6k)
   365038  Merged (365.0k, 97.44%)
   257188  Alignments with zero diffs (68.65%)
      495  Too many diffs (> 10) (0.13%)
        1  Fwd too short (< 64) after tail trimming (0.00%)
       10  Rev too short (< 64) after tail trimming (0.00%)
     9097  No alignment found (2.43%)
        3  Alignment too short (< 20) (0.00%)
```

```
         0  Merged too short (< 100)
         0  Min Q too low (<0) (0.00%)
        76  Staggered pairs (0.02%) merged & trimmed
     30.99  Mean alignment length
    470.44  Mean merged length
      0.56  Mean fwd expected errors
      0.72  Mean rev expected errors
      0.82  Mean merged expected errors
-----
  ▸ HG033-J2 - Merging Summary
    Merging Rate: 194658 / 213379 (91.2%)
    Median Merged Length: 472

    213379  Pairs (213.4k)
    194658  Merged (194.7k, 91.23%)
    129054  Alignments with zero diffs (60.48%)
      3450  Too many diffs (> 10) (1.62%)
        24  Fwd too short (< 64) after tail trimming (0.01%)
       167  Rev too short (< 64) after tail trimming (0.08%)
     15059  No alignment found (7.06%)
        21  Alignment too short (< 20) (0.01%)
         0  Merged too short (< 100)
         0  Min Q too low (<0) (0.00%)
       307  Staggered pairs (0.14%) merged & trimmed
     46.01  Mean alignment length
    452.83  Mean merged length
      0.53  Mean fwd expected errors
      0.80  Mean rev expected errors
      0.74  Mean merged expected errors
-----
  ▸ HG033-J3 - Merging Summary
    Merging Rate: 187287 / 204649 (91.5%)
    Median Merged Length: 472

    204649  Pairs (204.6k)
    187287  Merged (187.3k, 91.52%)
     83094  Alignments with zero diffs (40.60%)
      1860  Too many diffs (> 10) (0.91%)
        33  Fwd too short (< 64) after tail trimming (0.02%)
       293  Rev too short (< 64) after tail trimming (0.14%)
     15157  No alignment found (7.41%)
        17  Alignment too short (< 20) (0.01%)
         2  Merged too short (< 100)
         0  Min Q too low (<0) (0.00%)
       487  Staggered pairs (0.24%) merged & trimmed
     35.55  Mean alignment length
    463.64  Mean merged length
      1.09  Mean fwd expected errors
      1.13  Mean rev expected errors
      1.20  Mean merged expected errors
-----
  ▸ HG033-L1 - Merging Summary
    Merging Rate: 253954 / 260589 (97.5%)
    Median Merged Length: 472
```

```
    260589  Pairs (260.6k)
    253954  Merged (254.0k, 97.45%)
    173783  Alignments with zero diffs (66.69%)
       494  Too many diffs (> 10) (0.19%)
         0  Fwd too short (< 64) after tail trimming (0.00%)
        15  Rev too short (< 64) after tail trimming (0.01%)
      6124  No alignment found (2.35%)
         2  Alignment too short (< 20) (0.00%)
         0  Merged too short (< 100)
         0  Min Q too low (<0) (0.00%)
        37  Staggered pairs (0.01%) merged & trimmed
     31.28  Mean alignment length
    470.31  Mean merged length
      0.56  Mean fwd expected errors
      0.80  Mean rev expected errors
      0.86  Mean merged expected errors
-----
  ‣ HG033-L2 - Merging Summary
    Merging Rate: 105089 / 128048 (82.1%)
    Median Merged Length: 472

    128048  Pairs (128.0k)
    105089  Merged (105.1k, 82.07%)
     61934  Alignments with zero diffs (48.37%)
      5522  Too many diffs (> 10) (4.31%)
        52  Fwd too short (< 64) after tail trimming (0.04%)
       421  Rev too short (< 64) after tail trimming (0.33%)
     16957  No alignment found (13.24%)
         4  Alignment too short (< 20) (0.00%)
         3  Merged too short (< 100)
         0  Min Q too low (<0) (0.00%)
       896  Staggered pairs (0.70%) merged & trimmed
     75.30  Mean alignment length
    413.14  Mean merged length
      0.55  Mean fwd expected errors
      0.93  Mean rev expected errors
      0.63  Mean merged expected errors
-----
  ‣ HG033-L3 - Merging Summary
    Merging Rate: 1424209 / 1540090 (92.5%)
    Median Merged Length: 472

   1540090  Pairs (1.5M)
   1424209  Merged (1.4M, 92.48%)
    585335  Alignments with zero diffs (38.01%)
     20344  Too many diffs (> 10) (1.32%)
       188  Fwd too short (< 64) after tail trimming (0.01%)
      1976  Rev too short (< 64) after tail trimming (0.13%)
     93332  No alignment found (6.06%)
        34  Alignment too short (< 20) (0.00%)
         7  Merged too short (< 100)
         0  Min Q too low (<0) (0.00%)
      2793  Staggered pairs (0.18%) merged & trimmed
```

```
    37.38  Mean alignment length
   461.93  Mean merged length
     1.13  Mean fwd expected errors
     1.20  Mean rev expected errors
     1.18  Mean merged expected errors
-----
  ‣ HG033-O1 - Merging Summary
    Merging Rate: 383562 / 406560 (94.3%)
    Median Merged Length: 453

    406560  Pairs (406.6k)
    383562  Merged (383.6k, 94.34%)
    221018  Alignments with zero diffs (54.36%)
      7010  Too many diffs (> 10) (1.72%)
         2  Fwd too short (< 64) after tail trimming (0.00%)
        33  Rev too short (< 64) after tail trimming (0.01%)
     15942  No alignment found (3.92%)
        11  Alignment too short (< 20) (0.00%)
         0  Merged too short (< 100)
         0  Min Q too low (<0) (0.00%)
       269  Staggered pairs (0.07%) merged & trimmed
     44.99  Mean alignment length
    455.45  Mean merged length
      0.58  Mean fwd expected errors
      0.98  Mean rev expected errors
      0.79  Mean merged expected errors
-----
  ‣ HG033-O2 - Merging Summary
    Merging Rate: 294874 / 311251 (94.7%)
    Median Merged Length: 449

    311251  Pairs (311.3k)
    294874  Merged (294.9k, 94.74%)
    159304  Alignments with zero diffs (51.18%)
      5897  Too many diffs (> 10) (1.89%)
         0  Fwd too short (< 64) after tail trimming (0.00%)
        31  Rev too short (< 64) after tail trimming (0.01%)
     10446  No alignment found (3.36%)
         3  Alignment too short (< 20) (0.00%)
         0  Merged too short (< 100)
         0  Min Q too low (<0) (0.00%)
       153  Staggered pairs (0.05%) merged & trimmed
     46.16  Mean alignment length
    454.84  Mean merged length
      0.71  Mean fwd expected errors
      0.91  Mean rev expected errors
      0.80  Mean merged expected errors
-----
  ‣ HG033-O3 - Merging Summary
    Merging Rate: 156360 / 167597 (93.3%)
    Median Merged Length: 472

    167597  Pairs (167.6k)
    156360  Merged (156.4k, 93.30%)
```

```
      62878  Alignments with zero diffs (37.52%)
       2867  Too many diffs (> 10) (1.71%)
          2  Fwd too short (< 64) after tail trimming (0.00%)
         19  Rev too short (< 64) after tail trimming (0.01%)
       8349  No alignment found (4.98%)
          0  Alignment too short (< 20) (0.00%)
          0  Merged too short (< 100)
          0  Min Q too low (<0) (0.00%)
        173  Staggered pairs (0.10%) merged & trimmed
      39.42  Mean alignment length
     461.16  Mean merged length
       1.09  Mean fwd expected errors
       1.27  Mean rev expected errors
       1.16  Mean merged expected errors
-----
  ▸ HG033-Q1 - Merging Summary
    Merging Rate: 207323 / 232391 (89.2%)
    Median Merged Length: 472

     232391  Pairs (232.4k)
     207323  Merged (207.3k, 89.21%)
     139144  Alignments with zero diffs (59.87%)
       4199  Too many diffs (> 10) (1.81%)
        255  Fwd too short (< 64) after tail trimming (0.11%)
       1159  Rev too short (< 64) after tail trimming (0.50%)
      19400  No alignment found (8.35%)
         47  Alignment too short (< 20) (0.02%)
          8  Merged too short (< 100)
          0  Min Q too low (<0) (0.00%)
       1124  Staggered pairs (0.48%) merged & trimmed
      48.67  Mean alignment length
     441.70  Mean merged length
       0.55  Mean fwd expected errors
       0.81  Mean rev expected errors
       0.73  Mean merged expected errors
-----
  ▸ HG033-Q2 - Merging Summary
    Merging Rate: 166797 / 186036 (89.7%)
    Median Merged Length: 449

     186036  Pairs (186.0k)
     166797  Merged (166.8k, 89.66%)
      52220  Alignments with zero diffs (28.07%)
       7338  Too many diffs (> 10) (3.94%)
         79  Fwd too short (< 64) after tail trimming (0.04%)
        367  Rev too short (< 64) after tail trimming (0.20%)
      11446  No alignment found (6.15%)
          8  Alignment too short (< 20) (0.00%)
          1  Merged too short (< 100)
          0  Min Q too low (<0) (0.00%)
        615  Staggered pairs (0.33%) merged & trimmed
      53.53  Mean alignment length
     444.39  Mean merged length
       1.05  Mean fwd expected errors
```

```
      1.51  Mean rev expected errors
      1.02  Mean merged expected errors
-----
  ▸ HG033-Q3 - Merging Summary
    Merging Rate: 154685 / 169903 (91.0%)
    Median Merged Length: 452

    169903  Pairs (169.9k)
    154685  Merged (154.7k, 91.04%)
     52385  Alignments with zero diffs (30.83%)
      6006  Too many diffs (> 10) (3.53%)
         2  Fwd too short (< 64) after tail trimming (0.00%)
        26  Rev too short (< 64) after tail trimming (0.02%)
      9181  No alignment found (5.40%)
         3  Alignment too short (< 20) (0.00%)
         0  Merged too short (< 100)
         0  Min Q too low (<0) (0.00%)
       128  Staggered pairs (0.08%) merged & trimmed
     48.04  Mean alignment length
    452.18  Mean merged length
      1.09  Mean fwd expected errors
      1.52  Mean rev expected errors
      1.12  Mean merged expected errors
-----
  ▸ HG033-R1 - Merging Summary
    Merging Rate: 269946 / 311161 (86.8%)
    Median Merged Length: 472

    311161  Pairs (311.2k)
    269946  Merged (269.9k, 86.75%)
    175166  Alignments with zero diffs (56.29%)
      6930  Too many diffs (> 10) (2.23%)
       222  Fwd too short (< 64) after tail trimming (0.07%)
      1400  Rev too short (< 64) after tail trimming (0.45%)
     32573  No alignment found (10.47%)
        85  Alignment too short (< 20) (0.03%)
         5  Merged too short (< 100)
         0  Min Q too low (<0) (0.00%)
      1800  Staggered pairs (0.58%) merged & trimmed
     54.87  Mean alignment length
    433.70  Mean merged length
      0.54  Mean fwd expected errors
      0.84  Mean rev expected errors
      0.70  Mean merged expected errors
-----
  ▸ HG033-R2 - Merging Summary
    Merging Rate: 129788 / 180853 (71.8%)
    Median Merged Length: 472

    180853  Pairs (180.9k)
    129788  Merged (129.8k, 71.76%)
     12430  Alignments with zero diffs (6.87%)
      2532  Too many diffs (> 10) (1.40%)
        12  Fwd too short (< 64) after tail trimming (0.01%)
```

```
        73  Rev too short (< 64) after tail trimming (0.04%)
     48439  No alignment found (26.78%)
         5  Alignment too short (< 20) (0.00%)
         4  Merged too short (< 100)
         0  Min Q too low (<0) (0.00%)
       772  Staggered pairs (0.43%) merged & trimmed
     31.13  Mean alignment length
    469.03  Mean merged length
      1.39  Mean fwd expected errors
      5.03  Mean rev expected errors
      4.44  Mean merged expected errors
-----
  ‣ HG033-R3 - Merging Summary
    Merging Rate: 130757 / 173627 (75.3%)
    Median Merged Length: 472

    173627  Pairs (173.6k)
    130757  Merged (130.8k, 75.31%)
     49222  Alignments with zero diffs (28.35%)
     13374  Too many diffs (> 10) (7.70%)
        11  Fwd too short (< 64) after tail trimming (0.01%)
        96  Rev too short (< 64) after tail trimming (0.06%)
     29376  No alignment found (16.92%)
        12  Alignment too short (< 20) (0.01%)
         1  Merged too short (< 100)
         0  Min Q too low (<0) (0.00%)
      1112  Staggered pairs (0.64%) merged & trimmed
     73.58  Mean alignment length
    418.46  Mean merged length
      1.03  Mean fwd expected errors
      1.49  Mean rev expected errors
      0.96  Mean merged expected errors
-----
  ‣ HG059-B1 - Merging Summary
    Merging Rate: 299558 / 310805 (96.4%)
    Median Merged Length: 472

    310805  Pairs (310.8k)
    299558  Merged (299.6k, 96.38%)
    217396  Alignments with zero diffs (69.95%)
       733  Too many diffs (> 10) (0.24%)
        11  Fwd too short (< 64) after tail trimming (0.00%)
       154  Rev too short (< 64) after tail trimming (0.05%)
     10341  No alignment found (3.33%)
         7  Alignment too short (< 20) (0.00%)
         1  Merged too short (< 100)
         0  Min Q too low (<0) (0.00%)
       297  Staggered pairs (0.10%) merged & trimmed
     32.63  Mean alignment length
    467.32  Mean merged length
      0.51  Mean fwd expected errors
      0.66  Mean rev expected errors
      0.75  Mean merged expected errors
-----
```

```
▸ HG059-B2 - Merging Summary
  Merging Rate: 285424 / 292915 (97.4%)
  Median Merged Length: 472

  292915  Pairs (292.9k)
  285424  Merged (285.4k, 97.44%)
  203629  Alignments with zero diffs (69.52%)
     526  Too many diffs (> 10) (0.18%)
       1  Fwd too short (< 64) after tail trimming (0.00%)
      18  Rev too short (< 64) after tail trimming (0.01%)
    6936  No alignment found (2.37%)
      10  Alignment too short (< 20) (0.00%)
       0  Merged too short (< 100)
       0  Min Q too low (<0) (0.00%)
     119  Staggered pairs (0.04%) merged & trimmed
   31.44  Mean alignment length
  469.34  Mean merged length
    0.56  Mean fwd expected errors
    0.68  Mean rev expected errors
    0.78  Mean merged expected errors
-----
▸ HG059-B3 - Merging Summary
  Merging Rate: 166789 / 177485 (94.0%)
  Median Merged Length: 449

  177485  Pairs (177.5k)
  166789  Merged (166.8k, 93.97%)
   52528  Alignments with zero diffs (29.60%)
    4870  Too many diffs (> 10) (2.74%)
       0  Fwd too short (< 64) after tail trimming (0.00%)
       5  Rev too short (< 64) after tail trimming (0.00%)
    5820  No alignment found (3.28%)
       1  Alignment too short (< 20) (0.00%)
       0  Merged too short (< 100)
       0  Min Q too low (<0) (0.00%)
      36  Staggered pairs (0.02%) merged & trimmed
   46.92  Mean alignment length
  454.58  Mean merged length
    1.05  Mean fwd expected errors
    1.53  Mean rev expected errors
    1.05  Mean merged expected errors
-----
▸ HG059-D1 - Merging Summary
  Merging Rate: 365561 / 380203 (96.1%)
  Median Merged Length: 472

  380203  Pairs (380.2k)
  365561  Merged (365.6k, 96.15%)
  255640  Alignments with zero diffs (67.24%)
    1563  Too many diffs (> 10) (0.41%)
      27  Fwd too short (< 64) after tail trimming (0.01%)
     274  Rev too short (< 64) after tail trimming (0.07%)
   12765  No alignment found (3.36%)
      13  Alignment too short (< 20) (0.00%)
```

```
         0  Merged too short (< 100)
         0  Min Q too low (<0) (0.00%)
       566  Staggered pairs (0.15%) merged & trimmed
     34.93  Mean alignment length
    463.66  Mean merged length
      0.54  Mean fwd expected errors
      0.69  Mean rev expected errors
      0.75  Mean merged expected errors
-----
  ‣ HG059-D2 - Merging Summary
    Merging Rate: 414002 / 426597 (97.0%)
    Median Merged Length: 472

    426597  Pairs (426.6k)
    414002  Merged (414.0k, 97.05%)
    287888  Alignments with zero diffs (67.48%)
      1565  Too many diffs (> 10) (0.37%)
         1  Fwd too short (< 64) after tail trimming (0.00%)
        26  Rev too short (< 64) after tail trimming (0.01%)
     11001  No alignment found (2.58%)
         2  Alignment too short (< 20) (0.00%)
         0  Merged too short (< 100)
         0  Min Q too low (<0) (0.00%)
       163  Staggered pairs (0.04%) merged & trimmed
     32.26  Mean alignment length
    468.23  Mean merged length
      0.58  Mean fwd expected errors
      0.75  Mean rev expected errors
      0.81  Mean merged expected errors
-----
  ‣ HG059-D3 - Merging Summary
    Merging Rate: 340879 / 361301 (94.3%)
    Median Merged Length: 472

    361301  Pairs (361.3k)
    340879  Merged (340.9k, 94.35%)
    141135  Alignments with zero diffs (39.06%)
      3366  Too many diffs (> 10) (0.93%)
         2  Fwd too short (< 64) after tail trimming (0.00%)
         9  Rev too short (< 64) after tail trimming (0.00%)
     17035  No alignment found (4.71%)
        10  Alignment too short (< 20) (0.00%)
         0  Merged too short (< 100)
         0  Min Q too low (<0) (0.00%)
       215  Staggered pairs (0.06%) merged & trimmed
     34.58  Mean alignment length
    466.50  Mean merged length
      1.10  Mean fwd expected errors
      1.25  Mean rev expected errors
      1.24  Mean merged expected errors
-----
  ‣ HG059-I1 - Merging Summary
    Merging Rate: 570580 / 582329 (98.0%)
    Median Merged Length: 472
```

```
   582329  Pairs (582.3k)
   570580  Merged (570.6k, 97.98%)
   410431  Alignments with zero diffs (70.48%)
      769  Too many diffs (> 10) (0.13%)
        2  Fwd too short (< 64) after tail trimming (0.00%)
        6  Rev too short (< 64) after tail trimming (0.00%)
    10969  No alignment found (1.88%)
        2  Alignment too short (< 20) (0.00%)
        1  Merged too short (< 100)
        0  Min Q too low (<0) (0.00%)
       69  Staggered pairs (0.01%) merged & trimmed
    30.81  Mean alignment length
   470.76  Mean merged length
     0.53  Mean fwd expected errors
     0.67  Mean rev expected errors
     0.78  Mean merged expected errors
-----
  ▸ HG059-I2 - Merging Summary
   Merging Rate: 171100 / 179032 (95.6%)
   Median Merged Length: 472

   179032  Pairs (179.0k)
   171100  Merged (171.1k, 95.57%)
   115335  Alignments with zero diffs (64.42%)
     1209  Too many diffs (> 10) (0.68%)
        1  Fwd too short (< 64) after tail trimming (0.00%)
       54  Rev too short (< 64) after tail trimming (0.03%)
     6664  No alignment found (3.72%)
        4  Alignment too short (< 20) (0.00%)
        0  Merged too short (< 100)
        0  Min Q too low (<0) (0.00%)
      148  Staggered pairs (0.08%) merged & trimmed
    35.88  Mean alignment length
   464.46  Mean merged length
     0.57  Mean fwd expected errors
     0.77  Mean rev expected errors
     0.81  Mean merged expected errors
-----
  ▸ HG059-I3 - Merging Summary
   Merging Rate: 200160 / 210752 (95.0%)
   Median Merged Length: 472

   210752  Pairs (210.8k)
   200160  Merged (200.2k, 94.97%)
    92200  Alignments with zero diffs (43.75%)
      450  Too many diffs (> 10) (0.21%)
        2  Fwd too short (< 64) after tail trimming (0.00%)
       10  Rev too short (< 64) after tail trimming (0.00%)
    10128  No alignment found (4.81%)
        2  Alignment too short (< 20) (0.00%)
        0  Merged too short (< 100)
        0  Min Q too low (<0) (0.00%)
       61  Staggered pairs (0.03%) merged & trimmed
```

```
     30.62  Mean alignment length
    470.76  Mean merged length
      1.14  Mean fwd expected errors
      1.03  Mean rev expected errors
      1.21  Mean merged expected errors
-----
  ‣ HG059-J1 - Merging Summary
    Merging Rate: 264832 / 275618 (96.1%)
    Median Merged Length: 472

    275618  Pairs (275.6k)
    264832  Merged (264.8k, 96.09%)
    169676  Alignments with zero diffs (61.56%)
      2618  Too many diffs (> 10) (0.95%)
         7  Fwd too short (< 64) after tail trimming (0.00%)
        51  Rev too short (< 64) after tail trimming (0.02%)
      8104  No alignment found (2.94%)
         5  Alignment too short (< 20) (0.00%)
         1  Merged too short (< 100)
         0  Min Q too low (<0) (0.00%)
       121  Staggered pairs (0.04%) merged & trimmed
     37.61  Mean alignment length
    463.37  Mean merged length
      0.61  Mean fwd expected errors
      0.80  Mean rev expected errors
      0.79  Mean merged expected errors
-----
  ‣ HG059-J2 - Merging Summary
    Merging Rate: 279293 / 308184 (90.6%)
    Median Merged Length: 472

    308184  Pairs (308.2k)
    279293  Merged (279.3k, 90.63%)
    190547  Alignments with zero diffs (61.83%)
      4294  Too many diffs (> 10) (1.39%)
         7  Fwd too short (< 64) after tail trimming (0.00%)
       113  Rev too short (< 64) after tail trimming (0.04%)
     24453  No alignment found (7.93%)
        24  Alignment too short (< 20) (0.01%)
         0  Merged too short (< 100)
         0  Min Q too low (<0) (0.00%)
       691  Staggered pairs (0.22%) merged & trimmed
     45.42  Mean alignment length
    452.11  Mean merged length
      0.54  Mean fwd expected errors
      0.74  Mean rev expected errors
      0.73  Mean merged expected errors
-----
  ‣ HG059-J3 - Merging Summary
    Merging Rate: 979581 / 1032201 (94.9%)
    Median Merged Length: 472

   1032201  Pairs (1.0M)
    979581  Merged (979.6k, 94.90%)
```

```
   452191  Alignments with zero diffs (43.81%)
     4483  Too many diffs (> 10) (0.43%)
       42  Fwd too short (< 64) after tail trimming (0.00%)
      857  Rev too short (< 64) after tail trimming (0.08%)
    47206  No alignment found (4.57%)
       32  Alignment too short (< 20) (0.00%)
        0  Merged too short (< 100)
        0  Min Q too low (<0) (0.00%)
      959  Staggered pairs (0.09%) merged & trimmed
    32.22  Mean alignment length
   468.23  Mean merged length
     1.08  Mean fwd expected errors
     1.05  Mean rev expected errors
     1.16  Mean merged expected errors
-----
  ▸ HG059-L1 - Merging Summary
    Merging Rate: 169866 / 175186 (97.0%)
    Median Merged Length: 470

   175186  Pairs (175.2k)
   169866  Merged (169.9k, 96.96%)
   102526  Alignments with zero diffs (58.52%)
     1689  Too many diffs (> 10) (0.96%)
        3  Fwd too short (< 64) after tail trimming (0.00%)
       13  Rev too short (< 64) after tail trimming (0.01%)
     3611  No alignment found (2.06%)
        3  Alignment too short (< 20) (0.00%)
        1  Merged too short (< 100)
        0  Min Q too low (<0) (0.00%)
       29  Staggered pairs (0.02%) merged & trimmed
    39.88  Mean alignment length
   461.64  Mean merged length
     0.57  Mean fwd expected errors
     0.88  Mean rev expected errors
     0.79  Mean merged expected errors
-----
  ▸ HG059-L2 - Merging Summary
    Merging Rate: 292870 / 314100 (93.2%)
    Median Merged Length: 472

   314100  Pairs (314.1k)
   292870  Merged (292.9k, 93.24%)
   207900  Alignments with zero diffs (66.19%)
     1977  Too many diffs (> 10) (0.63%)
        4  Fwd too short (< 64) after tail trimming (0.00%)
       72  Rev too short (< 64) after tail trimming (0.02%)
    19027  No alignment found (6.06%)
      150  Alignment too short (< 20) (0.05%)
        0  Merged too short (< 100)
        0  Min Q too low (<0) (0.00%)
      570  Staggered pairs (0.18%) merged & trimmed
    37.42  Mean alignment length
   460.85  Mean merged length
     0.59  Mean fwd expected errors
```

```
      0.65  Mean rev expected errors
      0.74  Mean merged expected errors
-----
  ‣ HG059-L3 - Merging Summary
    Merging Rate: 226404 / 244038 (92.8%)
    Median Merged Length: 453

    244038  Pairs (244.0k)
    226404  Merged (226.4k, 92.77%)
     81515  Alignments with zero diffs (33.40%)
      5892  Too many diffs (> 10) (2.41%)
         1  Fwd too short (< 64) after tail trimming (0.00%)
        22  Rev too short (< 64) after tail trimming (0.01%)
     11714  No alignment found (4.80%)
         5  Alignment too short (< 20) (0.00%)
         0  Merged too short (< 100)
         0  Min Q too low (<0) (0.00%)
       153  Staggered pairs (0.06%) merged & trimmed
     44.00  Mean alignment length
    456.81  Mean merged length
      1.04  Mean fwd expected errors
      1.41  Mean rev expected errors
      1.07  Mean merged expected errors
-----
  ‣ HG059-O1 - Merging Summary
    Merging Rate: 380858 / 393513 (96.8%)
    Median Merged Length: 472

    393513  Pairs (393.5k)
    380858  Merged (380.9k, 96.78%)
    262235  Alignments with zero diffs (66.64%)
      1311  Too many diffs (> 10) (0.33%)
         0  Fwd too short (< 64) after tail trimming (0.00%)
        18  Rev too short (< 64) after tail trimming (0.00%)
     11324  No alignment found (2.88%)
         1  Alignment too short (< 20) (0.00%)
         1  Merged too short (< 100)
         0  Min Q too low (<0) (0.00%)
       130  Staggered pairs (0.03%) merged & trimmed
     32.87  Mean alignment length
    468.24  Mean merged length
      0.56  Mean fwd expected errors
      0.75  Mean rev expected errors
      0.82  Mean merged expected errors
-----
  ‣ HG059-O2 - Merging Summary
    Merging Rate: 308175 / 323235 (95.3%)
    Median Merged Length: 449

    323235  Pairs (323.2k)
    308175  Merged (308.2k, 95.34%)
    148498  Alignments with zero diffs (45.94%)
      7125  Too many diffs (> 10) (2.20%)
         1  Fwd too short (< 64) after tail trimming (0.00%)
```

```
         6  Rev too short (< 64) after tail trimming (0.00%)
      7927  No alignment found (2.45%)
         1  Alignment too short (< 20) (0.00%)
         0  Merged too short (< 100)
         0  Min Q too low (<0) (0.00%)
        29  Staggered pairs (0.01%) merged & trimmed
     49.91  Mean alignment length
    451.73  Mean merged length
      0.73  Mean fwd expected errors
      1.02  Mean rev expected errors
      0.81  Mean merged expected errors
-----
  ‣ HG059-O3 - Merging Summary
    Merging Rate: 133989 / 139386 (96.1%)
    Median Merged Length: 472

    139386  Pairs (139.4k)
    133989  Merged (134.0k, 96.13%)
     61755  Alignments with zero diffs (44.31%)
       212  Too many diffs (> 10) (0.15%)
         1  Fwd too short (< 64) after tail trimming (0.00%)
         3  Rev too short (< 64) after tail trimming (0.00%)
      5181  No alignment found (3.72%)
         0  Alignment too short (< 20) (0.00%)
         0  Merged too short (< 100)
         0  Min Q too low (<0) (0.00%)
        11  Staggered pairs (0.01%) merged & trimmed
     30.12  Mean alignment length
    471.43  Mean merged length
      1.07  Mean fwd expected errors
      1.06  Mean rev expected errors
      1.19  Mean merged expected errors
-----
  ‣ HG059-Q1 - Merging Summary
    Merging Rate: 594120 / 611333 (97.2%)
    Median Merged Length: 472

    611333  Pairs (611.3k)
    594120  Merged (594.1k, 97.18%)
    386923  Alignments with zero diffs (63.29%)
      3792  Too many diffs (> 10) (0.62%)
        25  Fwd too short (< 64) after tail trimming (0.00%)
       209  Rev too short (< 64) after tail trimming (0.03%)
     13185  No alignment found (2.16%)
         2  Alignment too short (< 20) (0.00%)
         0  Merged too short (< 100)
         0  Min Q too low (<0) (0.00%)
       168  Staggered pairs (0.03%) merged & trimmed
     34.90  Mean alignment length
    466.33  Mean merged length
      0.58  Mean fwd expected errors
      0.81  Mean rev expected errors
      0.82  Mean merged expected errors
-----
```

```
  ▸ HG059-Q2 - Merging Summary
    Merging Rate: 136361 / 148164 (92.0%)
    Median Merged Length: 472

    148164  Pairs (148.2k)
    136361  Merged (136.4k, 92.03%)
     52976  Alignments with zero diffs (35.75%)
      3499  Too many diffs (> 10) (2.36%)
        25  Fwd too short (< 64) after tail trimming (0.02%)
       372  Rev too short (< 64) after tail trimming (0.25%)
      7902  No alignment found (5.33%)
         5  Alignment too short (< 20) (0.00%)
         0  Merged too short (< 100)
         0  Min Q too low (<0) (0.00%)
       389  Staggered pairs (0.26%) merged & trimmed
     41.23  Mean alignment length
    456.87  Mean merged length
      1.25  Mean fwd expected errors
      1.35  Mean rev expected errors
      1.22  Mean merged expected errors
-----
  ▸ HG059-Q3 - Merging Summary
    Merging Rate: 169720 / 176989 (95.9%)
    Median Merged Length: 472

    176989  Pairs (177.0k)
    169720  Merged (169.7k, 95.89%)
     75353  Alignments with zero diffs (42.57%)
       274  Too many diffs (> 10) (0.15%)
         0  Fwd too short (< 64) after tail trimming (0.00%)
         1  Rev too short (< 64) after tail trimming (0.00%)
      6994  No alignment found (3.95%)
         0  Alignment too short (< 20) (0.00%)
         0  Merged too short (< 100)
         0  Min Q too low (<0) (0.00%)
        14  Staggered pairs (0.01%) merged & trimmed
     30.12  Mean alignment length
    471.57  Mean merged length
      1.20  Mean fwd expected errors
      1.10  Mean rev expected errors
      1.30  Mean merged expected errors
-----
  ▸ HG059-R1 - Merging Summary
    Merging Rate: 336322 / 344562 (97.6%)
    Median Merged Length: 472

    344562  Pairs (344.6k)
    336322  Merged (336.3k, 97.61%)
    241358  Alignments with zero diffs (70.05%)
       521  Too many diffs (> 10) (0.15%)
         6  Fwd too short (< 64) after tail trimming (0.00%)
       125  Rev too short (< 64) after tail trimming (0.04%)
      7585  No alignment found (2.20%)
         3  Alignment too short (< 20) (0.00%)
```

```
         0  Merged too short (< 100)
         0  Min Q too low (<0) (0.00%)
       136  Staggered pairs (0.04%) merged & trimmed
     30.80  Mean alignment length
    470.38  Mean merged length
      0.53  Mean fwd expected errors
      0.69  Mean rev expected errors
      0.79  Mean merged expected errors
-----
  ▸ HG059-R2 - Merging Summary
    Merging Rate: 202030 / 210472 (96.0%)
    Median Merged Length: 472

    210472  Pairs (210.5k)
    202030  Merged (202.0k, 95.99%)
     95229  Alignments with zero diffs (45.25%)
       246  Too many diffs (> 10) (0.12%)
         3  Fwd too short (< 64) after tail trimming (0.00%)
        51  Rev too short (< 64) after tail trimming (0.02%)
      8138  No alignment found (3.87%)
         4  Alignment too short (< 20) (0.00%)
         0  Merged too short (< 100)
         0  Min Q too low (<0) (0.00%)
        78  Staggered pairs (0.04%) merged & trimmed
     30.46  Mean alignment length
    470.74  Mean merged length
      1.07  Mean fwd expected errors
      1.02  Mean rev expected errors
      1.17  Mean merged expected errors
-----
  ▸ HG059-R3 - Merging Summary
    Merging Rate: 251615 / 267427 (94.1%)
    Median Merged Length: 453

    267427  Pairs (267.4k)
    251615  Merged (251.6k, 94.09%)
     90875  Alignments with zero diffs (33.98%)
      6087  Too many diffs (> 10) (2.28%)
         1  Fwd too short (< 64) after tail trimming (0.00%)
        11  Rev too short (< 64) after tail trimming (0.00%)
      9704  No alignment found (3.63%)
         9  Alignment too short (< 20) (0.00%)
         0  Merged too short (< 100)
         0  Min Q too low (<0) (0.00%)
        83  Staggered pairs (0.03%) merged & trimmed
     42.71  Mean alignment length
    458.64  Mean merged length
      1.09  Mean fwd expected errors
      1.35  Mean rev expected errors
      1.08  Mean merged expected errors
-----
  ▸ HG082-B1 - Merging Summary
    Merging Rate: 233156 / 239513 (97.3%)
    Median Merged Length: 472
```

```
    239513  Pairs (239.5k)
    233156  Merged (233.2k, 97.35%)
    165675  Alignments with zero diffs (69.17%)
       449  Too many diffs (> 10) (0.19%)
         8  Fwd too short (< 64) after tail trimming (0.00%)
       185  Rev too short (< 64) after tail trimming (0.08%)
      5713  No alignment found (2.39%)
         2  Alignment too short (< 20) (0.00%)
         0  Merged too short (< 100)
         0  Min Q too low (<0) (0.00%)
       148  Staggered pairs (0.06%) merged & trimmed
     31.54  Mean alignment length
    468.97  Mean merged length
      0.58  Mean fwd expected errors
      0.66  Mean rev expected errors
      0.79  Mean merged expected errors
-----
  ▸ HG082-B2 - Merging Summary
    Merging Rate: 407738 / 435960 (93.5%)
    Median Merged Length: 449

    435960  Pairs (436.0k)
    407738  Merged (407.7k, 93.53%)
    136947  Alignments with zero diffs (31.41%)
     12276  Too many diffs (> 10) (2.82%)
         0  Fwd too short (< 64) after tail trimming (0.00%)
         8  Rev too short (< 64) after tail trimming (0.00%)
     15936  No alignment found (3.66%)
         2  Alignment too short (< 20) (0.00%)
         0  Merged too short (< 100)
         0  Min Q too low (<0) (0.00%)
        74  Staggered pairs (0.02%) merged & trimmed
     46.60  Mean alignment length
    454.74  Mean merged length
      1.07  Mean fwd expected errors
      1.39  Mean rev expected errors
      1.03  Mean merged expected errors
-----
  ▸ HG082-B3 - Merging Summary
    Merging Rate: 179790 / 189287 (95.0%)
    Median Merged Length: 472

    189287  Pairs (189.3k)
    179790  Merged (179.8k, 94.98%)
     78107  Alignments with zero diffs (41.26%)
      1267  Too many diffs (> 10) (0.67%)
         1  Fwd too short (< 64) after tail trimming (0.00%)
         6  Rev too short (< 64) after tail trimming (0.00%)
      8221  No alignment found (4.34%)
         2  Alignment too short (< 20) (0.00%)
         0  Merged too short (< 100)
         0  Min Q too low (<0) (0.00%)
        47  Staggered pairs (0.02%) merged & trimmed
```

```
     33.36  Mean alignment length
    467.92  Mean merged length
      1.17  Mean fwd expected errors
      1.10  Mean rev expected errors
      1.21  Mean merged expected errors
-----
  ‣ HG082-D1 - Merging Summary
    Merging Rate: 196308 / 205849 (95.4%)
    Median Merged Length: 472

    205849  Pairs (205.8k)
    196308  Merged (196.3k, 95.37%)
    132341  Alignments with zero diffs (64.29%)
      1464  Too many diffs (> 10) (0.71%)
        34  Fwd too short (< 64) after tail trimming (0.02%)
       332  Rev too short (< 64) after tail trimming (0.16%)
      7701  No alignment found (3.74%)
         7  Alignment too short (< 20) (0.00%)
         3  Merged too short (< 100)
         0  Min Q too low (<0) (0.00%)
       385  Staggered pairs (0.19%) merged & trimmed
     37.64  Mean alignment length
    460.38  Mean merged length
      0.59  Mean fwd expected errors
      0.75  Mean rev expected errors
      0.79  Mean merged expected errors
-----
  ‣ HG082-D2 - Merging Summary
    Merging Rate: 235969 / 248475 (95.0%)
    Median Merged Length: 472

    248475  Pairs (248.5k)
    235969  Merged (236.0k, 94.97%)
    103888  Alignments with zero diffs (41.81%)
      2234  Too many diffs (> 10) (0.90%)
         5  Fwd too short (< 64) after tail trimming (0.00%)
        37  Rev too short (< 64) after tail trimming (0.01%)
     10230  No alignment found (4.12%)
         0  Alignment too short (< 20) (0.00%)
         0  Merged too short (< 100)
         0  Min Q too low (<0) (0.00%)
        78  Staggered pairs (0.03%) merged & trimmed
     34.61  Mean alignment length
    466.45  Mean merged length
      1.08  Mean fwd expected errors
      1.13  Mean rev expected errors
      1.13  Mean merged expected errors
-----
  ‣ HG082-D3 - Merging Summary
    Merging Rate: 181678 / 191066 (95.1%)
    Median Merged Length: 472

    191066  Pairs (191.1k)
    181678  Merged (181.7k, 95.09%)
```

```
     80331  Alignments with zero diffs (42.04%)
      1179  Too many diffs (> 10) (0.62%)
         2  Fwd too short (< 64) after tail trimming (0.00%)
        22  Rev too short (< 64) after tail trimming (0.01%)
      8182  No alignment found (4.28%)
         3  Alignment too short (< 20) (0.00%)
         0  Merged too short (< 100)
         0  Min Q too low (<0) (0.00%)
       102  Staggered pairs (0.05%) merged & trimmed
     33.49  Mean alignment length
    467.25  Mean merged length
      1.16  Mean fwd expected errors
      1.08  Mean rev expected errors
      1.19  Mean merged expected errors
-----
  ▸ HG082-I1 - Merging Summary
    Merging Rate: 132160 / 150856 (87.6%)
    Median Merged Length: 471

    150856  Pairs (150.9k)
    132160  Merged (132.2k, 87.61%)
     78005  Alignments with zero diffs (51.71%)
      6473  Too many diffs (> 10) (4.29%)
         8  Fwd too short (< 64) after tail trimming (0.01%)
       216  Rev too short (< 64) after tail trimming (0.14%)
     11888  No alignment found (7.88%)
       111  Alignment too short (< 20) (0.07%)
         0  Merged too short (< 100)
         0  Min Q too low (<0) (0.00%)
       537  Staggered pairs (0.36%) merged & trimmed
     73.02  Mean alignment length
    420.74  Mean merged length
      0.56  Mean fwd expected errors
      0.92  Mean rev expected errors
      0.63  Mean merged expected errors
-----
  ▸ HG082-I2 - Merging Summary
    Merging Rate: 258250 / 264553 (97.6%)
    Median Merged Length: 472

    264553  Pairs (264.6k)
    258250  Merged (258.2k, 97.62%)
    179644  Alignments with zero diffs (67.90%)
       619  Too many diffs (> 10) (0.23%)
         0  Fwd too short (< 64) after tail trimming (0.00%)
        12  Rev too short (< 64) after tail trimming (0.00%)
      5669  No alignment found (2.14%)
         3  Alignment too short (< 20) (0.00%)
         0  Merged too short (< 100)
         0  Min Q too low (<0) (0.00%)
        34  Staggered pairs (0.01%) merged & trimmed
     31.79  Mean alignment length
    469.84  Mean merged length
      0.58  Mean fwd expected errors
```

```
      0.70  Mean rev expected errors
      0.80  Mean merged expected errors
-----
  ▸ HG082-I3 - Merging Summary
    Merging Rate: 285979 / 302858 (94.4%)
    Median Merged Length: 472

    302858  Pairs (302.9k)
    285979  Merged (286.0k, 94.43%)
    113611  Alignments with zero diffs (37.51%)
      4673  Too many diffs (> 10) (1.54%)
         3  Fwd too short (< 64) after tail trimming (0.00%)
        50  Rev too short (< 64) after tail trimming (0.02%)
     12146  No alignment found (4.01%)
         6  Alignment too short (< 20) (0.00%)
         1  Merged too short (< 100)
         0  Min Q too low (<0) (0.00%)
       205  Staggered pairs (0.07%) merged & trimmed
     38.32  Mean alignment length
    462.74  Mean merged length
      1.14  Mean fwd expected errors
      1.23  Mean rev expected errors
      1.14  Mean merged expected errors
-----
  ▸ HG082-J1 - Merging Summary
    Merging Rate: 254121 / 260628 (97.5%)
    Median Merged Length: 472

    260628  Pairs (260.6k)
    254121  Merged (254.1k, 97.50%)
    169333  Alignments with zero diffs (64.97%)
      1256  Too many diffs (> 10) (0.48%)
         0  Fwd too short (< 64) after tail trimming (0.00%)
        15  Rev too short (< 64) after tail trimming (0.01%)
      5233  No alignment found (2.01%)
         2  Alignment too short (< 20) (0.00%)
         1  Merged too short (< 100)
         0  Min Q too low (<0) (0.00%)
        43  Staggered pairs (0.02%) merged & trimmed
     34.52  Mean alignment length
    467.11  Mean merged length
      0.59  Mean fwd expected errors
      0.73  Mean rev expected errors
      0.78  Mean merged expected errors
-----
  ▸ HG082-J2 - Merging Summary
    Merging Rate: 224331 / 236740 (94.8%)
    Median Merged Length: 449

    236740  Pairs (236.7k)
    224331  Merged (224.3k, 94.76%)
     99446  Alignments with zero diffs (42.01%)
      5515  Too many diffs (> 10) (2.33%)
         0  Fwd too short (< 64) after tail trimming (0.00%)
```

```
       14  Rev too short (< 64) after tail trimming (0.01%)
     6867  No alignment found (2.90%)
       13  Alignment too short (< 20) (0.01%)
        0  Merged too short (< 100)
        0  Min Q too low (<0) (0.00%)
       40  Staggered pairs (0.02%) merged & trimmed
    50.04  Mean alignment length
   451.40  Mean merged length
     0.88  Mean fwd expected errors
     1.05  Mean rev expected errors
     0.88  Mean merged expected errors
-----
  ‣ HG082-J3 - Merging Summary
    Merging Rate: 154087 / 161146 (95.6%)
    Median Merged Length: 467

   161146  Pairs (161.1k)
   154087  Merged (154.1k, 95.62%)
    70834  Alignments with zero diffs (43.96%)
      459  Too many diffs (> 10) (0.28%)
        1  Fwd too short (< 64) after tail trimming (0.00%)
       21  Rev too short (< 64) after tail trimming (0.01%)
     6577  No alignment found (4.08%)
        1  Alignment too short (< 20) (0.00%)
        0  Merged too short (< 100)
        0  Min Q too low (<0) (0.00%)
       96  Staggered pairs (0.06%) merged & trimmed
    34.53  Mean alignment length
   466.03  Mean merged length
     1.38  Mean fwd expected errors
     0.93  Mean rev expected errors
     1.22  Mean merged expected errors
-----
  ‣ HG082-L1 - Merging Summary
    Merging Rate: 273920 / 280065 (97.8%)
    Median Merged Length: 472

   280065  Pairs (280.1k)
   273920  Merged (273.9k, 97.81%)
   196547  Alignments with zero diffs (70.18%)
      236  Too many diffs (> 10) (0.08%)
        2  Fwd too short (< 64) after tail trimming (0.00%)
       16  Rev too short (< 64) after tail trimming (0.01%)
     5890  No alignment found (2.10%)
        1  Alignment too short (< 20) (0.00%)
        0  Merged too short (< 100)
        0  Min Q too low (<0) (0.00%)
       49  Staggered pairs (0.02%) merged & trimmed
    30.22  Mean alignment length
   471.42  Mean merged length
     0.55  Mean fwd expected errors
     0.68  Mean rev expected errors
     0.80  Mean merged expected errors
-----
```

```
  ‣ HG082-L2 - Merging Summary
    Merging Rate: 649099 / 701676 (92.5%)
    Median Merged Length: 472

    701676  Pairs (701.7k)
    649099  Merged (649.1k, 92.51%)
    447736  Alignments with zero diffs (63.81%)
      6538  Too many diffs (> 10) (0.93%)
         9  Fwd too short (< 64) after tail trimming (0.00%)
       150  Rev too short (< 64) after tail trimming (0.02%)
     45765  No alignment found (6.52%)
       112  Alignment too short (< 20) (0.02%)
         3  Merged too short (< 100)
         0  Min Q too low (<0) (0.00%)
      1370  Staggered pairs (0.20%) merged & trimmed
     41.72  Mean alignment length
    456.28  Mean merged length
      0.53  Mean fwd expected errors
      0.72  Mean rev expected errors
      0.73  Mean merged expected errors
-----
  ‣ HG082-L3 - Merging Summary
    Merging Rate: 132337 / 140089 (94.5%)
    Median Merged Length: 472

    140089  Pairs (140.1k)
    132337  Merged (132.3k, 94.47%)
     57919  Alignments with zero diffs (41.34%)
       691  Too many diffs (> 10) (0.49%)
         2  Fwd too short (< 64) after tail trimming (0.00%)
        12  Rev too short (< 64) after tail trimming (0.01%)
      7045  No alignment found (5.03%)
         2  Alignment too short (< 20) (0.00%)
         0  Merged too short (< 100)
         0  Min Q too low (<0) (0.00%)
       133  Staggered pairs (0.09%) merged & trimmed
     32.35  Mean alignment length
    468.31  Mean merged length
      1.04  Mean fwd expected errors
      1.23  Mean rev expected errors
      1.24  Mean merged expected errors
-----
  ‣ HG082-O1 - Merging Summary
    Merging Rate: 193907 / 207691 (93.4%)
    Median Merged Length: 472

    207691  Pairs (207.7k)
    193907  Merged (193.9k, 93.36%)
    129806  Alignments with zero diffs (62.50%)
      2767  Too many diffs (> 10) (1.33%)
         3  Fwd too short (< 64) after tail trimming (0.00%)
        55  Rev too short (< 64) after tail trimming (0.03%)
     10936  No alignment found (5.27%)
        23  Alignment too short (< 20) (0.01%)
```

```
         0  Merged too short (< 100)
         0  Min Q too low (<0) (0.00%)
       265  Staggered pairs (0.13%) merged & trimmed
     39.48  Mean alignment length
    459.34  Mean merged length
      0.56  Mean fwd expected errors
      0.90  Mean rev expected errors
      0.83  Mean merged expected errors
-----
  ‣ HG082-O2 - Merging Summary
    Merging Rate: 113897 / 173083 (65.8%)
    Median Merged Length: 307

    173083  Pairs (173.1k)
    113897  Merged (113.9k, 65.80%)
     31810  Alignments with zero diffs (18.38%)
     29319  Too many diffs (> 10) (16.94%)
        64  Fwd too short (< 64) after tail trimming (0.04%)
       974  Rev too short (< 64) after tail trimming (0.56%)
     28788  No alignment found (16.63%)
        40  Alignment too short (< 20) (0.02%)
         1  Merged too short (< 100)
         0  Min Q too low (<0) (0.00%)
      3177  Staggered pairs (1.84%) merged & trimmed
    132.73  Mean alignment length
    338.75  Mean merged length
      0.83  Mean fwd expected errors
      2.00  Mean rev expected errors
      0.49  Mean merged expected errors
-----
  ‣ HG082-O3 - Merging Summary
    Merging Rate: 285431 / 307851 (92.7%)
    Median Merged Length: 472

    307851  Pairs (307.9k)
    285431  Merged (285.4k, 92.72%)
    109896  Alignments with zero diffs (35.70%)
      4810  Too many diffs (> 10) (1.56%)
        23  Fwd too short (< 64) after tail trimming (0.01%)
       223  Rev too short (< 64) after tail trimming (0.07%)
     17361  No alignment found (5.64%)
         3  Alignment too short (< 20) (0.00%)
         0  Merged too short (< 100)
         0  Min Q too low (<0) (0.00%)
       439  Staggered pairs (0.14%) merged & trimmed
     35.33  Mean alignment length
    464.26  Mean merged length
      1.11  Mean fwd expected errors
      1.59  Mean rev expected errors
      1.39  Mean merged expected errors
-----
  ‣ HG082-Q1 - Merging Summary
    Merging Rate: 196236 / 217864 (90.1%)
    Median Merged Length: 472
```

```
    217864  Pairs (217.9k)
    196236  Merged (196.2k, 90.07%)
    128060  Alignments with zero diffs (58.78%)
      5423  Too many diffs (> 10) (2.49%)
        92  Fwd too short (< 64) after tail trimming (0.04%)
      1717  Rev too short (< 64) after tail trimming (0.79%)
     14383  No alignment found (6.60%)
        12  Alignment too short (< 20) (0.01%)
         1  Merged too short (< 100)
         0  Min Q too low (<0) (0.00%)
      1202  Staggered pairs (0.55%) merged & trimmed
     53.04  Mean alignment length
    435.96  Mean merged length
      0.60  Mean fwd expected errors
      0.78  Mean rev expected errors
      0.70  Mean merged expected errors
-----
  ▸ HG082-Q2 - Merging Summary
    Merging Rate: 151567 / 161098 (94.1%)
    Median Merged Length: 471

    161098  Pairs (161.1k)
    151567  Merged (151.6k, 94.08%)
     60070  Alignments with zero diffs (37.29%)
      2954  Too many diffs (> 10) (1.83%)
        17  Fwd too short (< 64) after tail trimming (0.01%)
       187  Rev too short (< 64) after tail trimming (0.12%)
      6370  No alignment found (3.95%)
         2  Alignment too short (< 20) (0.00%)
         1  Merged too short (< 100)
         0  Min Q too low (<0) (0.00%)
       168  Staggered pairs (0.10%) merged & trimmed
     40.26  Mean alignment length
    459.47  Mean merged length
      1.07  Mean fwd expected errors
      1.27  Mean rev expected errors
      1.10  Mean merged expected errors
-----
  ▸ HG082-Q3 - Merging Summary
    Merging Rate: 264501 / 281454 (94.0%)
    Median Merged Length: 472

    281454  Pairs (281.5k)
    264501  Merged (264.5k, 93.98%)
    112637  Alignments with zero diffs (40.02%)
      2151  Too many diffs (> 10) (0.76%)
        61  Fwd too short (< 64) after tail trimming (0.02%)
       296  Rev too short (< 64) after tail trimming (0.11%)
     14431  No alignment found (5.13%)
        12  Alignment too short (< 20) (0.00%)
         2  Merged too short (< 100)
         0  Min Q too low (<0) (0.00%)
       488  Staggered pairs (0.17%) merged & trimmed
```

```
    33.54  Mean alignment length
   465.97  Mean merged length
     1.04  Mean fwd expected errors
     1.26  Mean rev expected errors
     1.26  Mean merged expected errors
-----
  ‣ HG082-R1 - Merging Summary
    Merging Rate: 326687 / 335360 (97.4%)
    Median Merged Length: 472

    335360  Pairs (335.4k)
    326687  Merged (326.7k, 97.41%)
    231430  Alignments with zero diffs (69.01%)
       816  Too many diffs (> 10) (0.24%)
        10  Fwd too short (< 64) after tail trimming (0.00%)
       163  Rev too short (< 64) after tail trimming (0.05%)
      7682  No alignment found (2.29%)
         2  Alignment too short (< 20) (0.00%)
         0  Merged too short (< 100)
         0  Min Q too low (<0) (0.00%)
       154  Staggered pairs (0.05%) merged & trimmed
     31.16  Mean alignment length
    469.39  Mean merged length
      0.57  Mean fwd expected errors
      0.72  Mean rev expected errors
      0.82  Mean merged expected errors
-----
  ‣ HG082-R2 - Merging Summary
    Merging Rate: 176402 / 184982 (95.4%)
    Median Merged Length: 472

    184982  Pairs (185.0k)
    176402  Merged (176.4k, 95.36%)
     74769  Alignments with zero diffs (40.42%)
      1167  Too many diffs (> 10) (0.63%)
         1  Fwd too short (< 64) after tail trimming (0.00%)
         8  Rev too short (< 64) after tail trimming (0.00%)
      7404  No alignment found (4.00%)
         0  Alignment too short (< 20) (0.00%)
         0  Merged too short (< 100)
         0  Min Q too low (<0) (0.00%)
        32  Staggered pairs (0.02%) merged & trimmed
     33.12  Mean alignment length
    468.31  Mean merged length
      1.07  Mean fwd expected errors
      1.07  Mean rev expected errors
      1.09  Mean merged expected errors
-----
  ‣ HG082-R3 - Merging Summary
    Merging Rate: 377585 / 412604 (91.5%)
    Median Merged Length: 472

    412604  Pairs (412.6k)
    377585  Merged (377.6k, 91.51%)
```

```
   167800  Alignments with zero diffs (40.67%)
     7242  Too many diffs (> 10) (1.76%)
      153  Fwd too short (< 64) after tail trimming (0.04%)
     1580  Rev too short (< 64) after tail trimming (0.38%)
    26032  No alignment found (6.31%)
       11  Alignment too short (< 20) (0.00%)
        1  Merged too short (< 100)
        0  Min Q too low (<0) (0.00%)
     1516  Staggered pairs (0.37%) merged & trimmed
    38.17  Mean alignment length
   458.46  Mean merged length
     1.04  Mean fwd expected errors
     1.19  Mean rev expected errors
     1.18  Mean merged expected errors
-----
  ‣ HG091-B1 - Merging Summary
   Merging Rate: 25786 / 29180 (88.4%)
   Median Merged Length: 472

    29180  Pairs (29.2k)
    25786  Merged (25.8k, 88.37%)
    16376  Alignments with zero diffs (56.12%)
      453  Too many diffs (> 10) (1.55%)
      132  Fwd too short (< 64) after tail trimming (0.45%)
      449  Rev too short (< 64) after tail trimming (1.54%)
     2320  No alignment found (7.95%)
       24  Alignment too short (< 20) (0.08%)
       16  Merged too short (< 100)
        0  Min Q too low (<0) (0.00%)
      228  Staggered pairs (0.78%) merged & trimmed
    50.08  Mean alignment length
   436.82  Mean merged length
     0.56  Mean fwd expected errors
     0.84  Mean rev expected errors
     0.73  Mean merged expected errors
-----
  ‣ HG091-B2 - Merging Summary
   Merging Rate: 151554 / 172312 (88.0%)
   Median Merged Length: 473

   172312  Pairs (172.3k)
   151554  Merged (151.6k, 87.95%)
    56153  Alignments with zero diffs (32.59%)
     4100  Too many diffs (> 10) (2.38%)
       84  Fwd too short (< 64) after tail trimming (0.05%)
      295  Rev too short (< 64) after tail trimming (0.17%)
    16252  No alignment found (9.43%)
       20  Alignment too short (< 20) (0.01%)
        7  Merged too short (< 100)
        0  Min Q too low (<0) (0.00%)
     1460  Staggered pairs (0.85%) merged & trimmed
    45.58  Mean alignment length
   445.16  Mean merged length
     1.09  Mean fwd expected errors
```

```
      1.28  Mean rev expected errors
      1.07  Mean merged expected errors
-----
  ▸ HG091-B3 - Merging Summary
    Merging Rate: 286803 / 304640 (94.1%)
    Median Merged Length: 472

    304640  Pairs (304.6k)
    286803  Merged (286.8k, 94.14%)
    120600  Alignments with zero diffs (39.59%)
      3719  Too many diffs (> 10) (1.22%)
         2  Fwd too short (< 64) after tail trimming (0.00%)
         7  Rev too short (< 64) after tail trimming (0.00%)
     14108  No alignment found (4.63%)
         1  Alignment too short (< 20) (0.00%)
         0  Merged too short (< 100)
         0  Min Q too low (<0) (0.00%)
       150  Staggered pairs (0.05%) merged & trimmed
     35.95  Mean alignment length
    465.18  Mean merged length
      1.13  Mean fwd expected errors
      1.16  Mean rev expected errors
      1.17  Mean merged expected errors
-----
  ▸ HG091-D1 - Merging Summary
    Merging Rate: 47676 / 54456 (87.5%)
    Median Merged Length: 449

     54456  Pairs (54.5k)
     47676  Merged (47.7k, 87.55%)
     18223  Alignments with zero diffs (33.46%)
      1852  Too many diffs (> 10) (3.40%)
       268  Fwd too short (< 64) after tail trimming (0.49%)
       549  Rev too short (< 64) after tail trimming (1.01%)
      3980  No alignment found (7.31%)
        87  Alignment too short (< 20) (0.16%)
        44  Merged too short (< 100)
         0  Min Q too low (<0) (0.00%)
       396  Staggered pairs (0.73%) merged & trimmed
     59.99  Mean alignment length
    431.49  Mean merged length
      0.54  Mean fwd expected errors
      1.54  Mean rev expected errors
      0.88  Mean merged expected errors
-----
  ▸ HG091-D2 - Merging Summary
    Merging Rate: 187918 / 211182 (89.0%)
    Median Merged Length: 472

    211182  Pairs (211.2k)
    187918  Merged (187.9k, 88.98%)
     78147  Alignments with zero diffs (37.00%)
      4249  Too many diffs (> 10) (2.01%)
        75  Fwd too short (< 64) after tail trimming (0.04%)
```

```
      485  Rev too short (< 64) after tail trimming (0.23%)
    18446  No alignment found (8.73%)
        6  Alignment too short (< 20) (0.00%)
        3  Merged too short (< 100)
        0  Min Q too low (<0) (0.00%)
     1291  Staggered pairs (0.61%) merged & trimmed
    42.82  Mean alignment length
   450.79  Mean merged length
     1.07  Mean fwd expected errors
     1.20  Mean rev expected errors
     1.12  Mean merged expected errors
-----
  ‣ HG091-D3 - Merging Summary
    Merging Rate: 68643 / 95735 (71.7%)
    Median Merged Length: 470

    95735  Pairs (95.7k)
    68643  Merged (68.6k, 71.70%)
    25588  Alignments with zero diffs (26.73%)
     7123  Too many diffs (> 10) (7.44%)
       11  Fwd too short (< 64) after tail trimming (0.01%)
       73  Rev too short (< 64) after tail trimming (0.08%)
    19861  No alignment found (20.75%)
       24  Alignment too short (< 20) (0.03%)
        0  Merged too short (< 100)
        0  Min Q too low (<0) (0.00%)
      838  Staggered pairs (0.88%) merged & trimmed
    95.12  Mean alignment length
   390.23  Mean merged length
     0.91  Mean fwd expected errors
     1.35  Mean rev expected errors
     0.72  Mean merged expected errors
-----
  ‣ HG091-I1 - Merging Summary
    Merging Rate: 249744 / 262081 (95.3%)
    Median Merged Length: 473

   262081  Pairs (262.1k)
   249744  Merged (249.7k, 95.29%)
   160865  Alignments with zero diffs (61.38%)
     1205  Too many diffs (> 10) (0.46%)
        3  Fwd too short (< 64) after tail trimming (0.00%)
       33  Rev too short (< 64) after tail trimming (0.01%)
    11091  No alignment found (4.23%)
        5  Alignment too short (< 20) (0.00%)
        0  Merged too short (< 100)
        0  Min Q too low (<0) (0.00%)
      102  Staggered pairs (0.04%) merged & trimmed
    31.90  Mean alignment length
   468.56  Mean merged length
     0.58  Mean fwd expected errors
     0.74  Mean rev expected errors
     0.76  Mean merged expected errors
-----
```

```
  ‣ HG091-I2 - Merging Summary
    Merging Rate: 284437 / 305104 (93.2%)
    Median Merged Length: 472

    305104  Pairs (305.1k)
    284437  Merged (284.4k, 93.23%)
    192760  Alignments with zero diffs (63.18%)
      3555  Too many diffs (> 10) (1.17%)
         9  Fwd too short (< 64) after tail trimming (0.00%)
       101  Rev too short (< 64) after tail trimming (0.03%)
     16995  No alignment found (5.57%)
         7  Alignment too short (< 20) (0.00%)
         0  Merged too short (< 100)
         0  Min Q too low (<0) (0.00%)
       493  Staggered pairs (0.16%) merged & trimmed
     42.22  Mean alignment length
    456.33  Mean merged length
      0.55  Mean fwd expected errors
      0.73  Mean rev expected errors
      0.72  Mean merged expected errors
-----
  ‣ HG091-I3 - Merging Summary
    Merging Rate: 149848 / 165878 (90.3%)
    Median Merged Length: 449

    165878  Pairs (165.9k)
    149848  Merged (149.8k, 90.34%)
     44026  Alignments with zero diffs (26.54%)
      6965  Too many diffs (> 10) (4.20%)
         2  Fwd too short (< 64) after tail trimming (0.00%)
         7  Rev too short (< 64) after tail trimming (0.00%)
      9053  No alignment found (5.46%)
         3  Alignment too short (< 20) (0.00%)
         0  Merged too short (< 100)
         0  Min Q too low (<0) (0.00%)
       134  Staggered pairs (0.08%) merged & trimmed
     51.55  Mean alignment length
    448.96  Mean merged length
      1.14  Mean fwd expected errors
      1.59  Mean rev expected errors
      1.09  Mean merged expected errors
-----
  ‣ HG091-J1 - Merging Summary
    Merging Rate: 7902 / 11134 (71.0%)
    Median Merged Length: 307

     11134  Pairs (11.1k)
      7902  Merged (7902, 70.97%)
      4035  Alignments with zero diffs (36.24%)
       790  Too many diffs (> 10) (7.10%)
       202  Fwd too short (< 64) after tail trimming (1.81%)
      1024  Rev too short (< 64) after tail trimming (9.20%)
      1175  No alignment found (10.55%)
        25  Alignment too short (< 20) (0.22%)
```

```
        16  Merged too short (< 100)
         0  Min Q too low (<0) (0.00%)
       328  Staggered pairs (2.95%) merged & trimmed
    106.86  Mean alignment length
    343.30  Mean merged length
      0.55  Mean fwd expected errors
      1.22  Mean rev expected errors
      0.52  Mean merged expected errors
-----
  ‣ HG091-J2 - Merging Summary
    Merging Rate: 238298 / 268037 (88.9%)
    Median Merged Length: 449

    268037  Pairs (268.0k)
    238298  Merged (238.3k, 88.90%)
    122782  Alignments with zero diffs (45.81%)
      7617  Too many diffs (> 10) (2.84%)
         5  Fwd too short (< 64) after tail trimming (0.00%)
        76  Rev too short (< 64) after tail trimming (0.03%)
     22020  No alignment found (8.22%)
        20  Alignment too short (< 20) (0.01%)
         1  Merged too short (< 100)
         0  Min Q too low (<0) (0.00%)
       650  Staggered pairs (0.24%) merged & trimmed
     61.40  Mean alignment length
    435.61  Mean merged length
      0.63  Mean fwd expected errors
      0.96  Mean rev expected errors
      0.68  Mean merged expected errors
-----
  ‣ HG091-J3 - Merging Summary
    Merging Rate: 116176 / 160605 (72.3%)
    Median Merged Length: 471

    160605  Pairs (160.6k)
    116176  Merged (116.2k, 72.34%)
     39909  Alignments with zero diffs (24.85%)
     10700  Too many diffs (> 10) (6.66%)
        21  Fwd too short (< 64) after tail trimming (0.01%)
       174  Rev too short (< 64) after tail trimming (0.11%)
     33471  No alignment found (20.84%)
        61  Alignment too short (< 20) (0.04%)
         2  Merged too short (< 100)
         0  Min Q too low (<0) (0.00%)
      1313  Staggered pairs (0.82%) merged & trimmed
     78.38  Mean alignment length
    411.70  Mean merged length
      1.00  Mean fwd expected errors
      1.49  Mean rev expected errors
      0.89  Mean merged expected errors
-----
  ‣ HG091-L1 - Merging Summary
    Merging Rate: 13088 / 19063 (68.7%)
    Median Merged Length: 363
```

```
      19063  Pairs (19.1k)
      13088  Merged (13.1k, 68.66%)
       7474  Alignments with zero diffs (39.21%)
        994  Too many diffs (> 10) (5.21%)
        354  Fwd too short (< 64) after tail trimming (1.86%)
       1030  Rev too short (< 64) after tail trimming (5.40%)
       3529  No alignment found (18.51%)
         43  Alignment too short (< 20) (0.23%)
         25  Merged too short (< 100)
          0  Min Q too low (<0) (0.00%)
        362  Staggered pairs (1.90%) merged & trimmed
      95.99  Mean alignment length
     359.98  Mean merged length
       0.54  Mean fwd expected errors
       1.02  Mean rev expected errors
       0.51  Mean merged expected errors
-----
```

▸ HG091-L2 - Merging Summary

```
     Merging Rate: 273265 / 288762 (94.6%)
     Median Merged Length: 449

     288762  Pairs (288.8k)
     273265  Merged (273.3k, 94.63%)
     147499  Alignments with zero diffs (51.08%)
       5482  Too many diffs (> 10) (1.90%)
          4  Fwd too short (< 64) after tail trimming (0.00%)
         80  Rev too short (< 64) after tail trimming (0.03%)
       9924  No alignment found (3.44%)
          7  Alignment too short (< 20) (0.00%)
          0  Merged too short (< 100)
          0  Min Q too low (<0) (0.00%)
        360  Staggered pairs (0.12%) merged & trimmed
      49.56  Mean alignment length
     449.89  Mean merged length
       0.60  Mean fwd expected errors
       0.96  Mean rev expected errors
       0.74  Mean merged expected errors
-----
```

▸ HG091-L3 - Merging Summary

```
     Merging Rate: 119610 / 134001 (89.3%)
     Median Merged Length: 472

     134001  Pairs (134.0k)
     119610  Merged (119.6k, 89.26%)
      51787  Alignments with zero diffs (38.65%)
       2146  Too many diffs (> 10) (1.60%)
          1  Fwd too short (< 64) after tail trimming (0.00%)
         18  Rev too short (< 64) after tail trimming (0.01%)
      12219  No alignment found (9.12%)
          5  Alignment too short (< 20) (0.00%)
          2  Merged too short (< 100)
          0  Min Q too low (<0) (0.00%)
        251  Staggered pairs (0.19%) merged & trimmed
```

```
     38.47  Mean alignment length
    461.05  Mean merged length
      1.11  Mean fwd expected errors
      1.18  Mean rev expected errors
      1.23  Mean merged expected errors
-----
  ‣ HG091-O1 - Merging Summary
    Merging Rate: 392016 / 415984 (94.2%)
    Median Merged Length: 449

    415984  Pairs (416.0k)
    392016  Merged (392.0k, 94.24%)
    210664  Alignments with zero diffs (50.64%)
      9179  Too many diffs (> 10) (2.21%)
         6  Fwd too short (< 64) after tail trimming (0.00%)
       148  Rev too short (< 64) after tail trimming (0.04%)
     14627  No alignment found (3.52%)
         7  Alignment too short (< 20) (0.00%)
         1  Merged too short (< 100)
         0  Min Q too low (<0) (0.00%)
       487  Staggered pairs (0.12%) merged & trimmed
     50.00  Mean alignment length
    449.02  Mean merged length
      0.61  Mean fwd expected errors
      1.06  Mean rev expected errors
      0.79  Mean merged expected errors
-----
  ‣ HG091-O2 - Merging Summary
    Merging Rate: 95205 / 114713 (83.0%)
    Median Merged Length: 471

    114713  Pairs (114.7k)
     95205  Merged (95.2k, 82.99%)
     31894  Alignments with zero diffs (27.80%)
      5072  Too many diffs (> 10) (4.42%)
       276  Fwd too short (< 64) after tail trimming (0.24%)
      1494  Rev too short (< 64) after tail trimming (1.30%)
     12634  No alignment found (11.01%)
        17  Alignment too short (< 20) (0.01%)
        15  Merged too short (< 100)
         0  Min Q too low (<0) (0.00%)
      1537  Staggered pairs (1.34%) merged & trimmed
     54.05  Mean alignment length
    430.87  Mean merged length
      1.04  Mean fwd expected errors
      1.53  Mean rev expected errors
      1.06  Mean merged expected errors
-----
  ‣ HG091-O3 - Merging Summary
    Merging Rate: 163001 / 182177 (89.5%)
    Median Merged Length: 449

    182177  Pairs (182.2k)
    163001  Merged (163.0k, 89.47%)
```

```
    51836  Alignments with zero diffs (28.45%)
     6745  Too many diffs (> 10) (3.70%)
        6  Fwd too short (< 64) after tail trimming (0.00%)
       81  Rev too short (< 64) after tail trimming (0.04%)
    12337  No alignment found (6.77%)
        7  Alignment too short (< 20) (0.00%)
        0  Merged too short (< 100)
        0  Min Q too low (<0) (0.00%)
      324  Staggered pairs (0.18%) merged & trimmed
    51.33  Mean alignment length
   447.48  Mean merged length
     1.12  Mean fwd expected errors
     1.37  Mean rev expected errors
     0.99  Mean merged expected errors
-----
  ‣ HG091-Q1 - Merging Summary
    Merging Rate: 238257 / 258554 (92.1%)
    Median Merged Length: 472

   258554  Pairs (258.6k)
   238257  Merged (238.3k, 92.15%)
   158371  Alignments with zero diffs (61.25%)
     5599  Too many diffs (> 10) (2.17%)
       95  Fwd too short (< 64) after tail trimming (0.04%)
     1229  Rev too short (< 64) after tail trimming (0.48%)
    13350  No alignment found (5.16%)
       21  Alignment too short (< 20) (0.01%)
        3  Merged too short (< 100)
        0  Min Q too low (<0) (0.00%)
     1095  Staggered pairs (0.42%) merged & trimmed
    46.24  Mean alignment length
   447.55  Mean merged length
     0.57  Mean fwd expected errors
     0.88  Mean rev expected errors
     0.78  Mean merged expected errors
-----
  ‣ HG091-Q2 - Merging Summary
    Merging Rate: 161089 / 171874 (93.7%)
    Median Merged Length: 473

   171874  Pairs (171.9k)
   161089  Merged (161.1k, 93.73%)
    70189  Alignments with zero diffs (40.84%)
      756  Too many diffs (> 10) (0.44%)
        0  Fwd too short (< 64) after tail trimming (0.00%)
        3  Rev too short (< 64) after tail trimming (0.00%)
    10021  No alignment found (5.83%)
        5  Alignment too short (< 20) (0.00%)
        0  Merged too short (< 100)
        0  Min Q too low (<0) (0.00%)
       42  Staggered pairs (0.02%) merged & trimmed
    30.53  Mean alignment length
   470.22  Mean merged length
     1.17  Mean fwd expected errors
```

```
      1.02  Mean rev expected errors
      1.14  Mean merged expected errors
-----
  ‣ HG091-Q3 - Merging Summary
    Merging Rate: 379087 / 429663 (88.2%)
    Median Merged Length: 449

    429663  Pairs (429.7k)
    379087  Merged (379.1k, 88.23%)
     97807  Alignments with zero diffs (22.76%)
     20964  Too many diffs (> 10) (4.88%)
       187  Fwd too short (< 64) after tail trimming (0.04%)
       455  Rev too short (< 64) after tail trimming (0.11%)
     28934  No alignment found (6.73%)
        31  Alignment too short (< 20) (0.01%)
         5  Merged too short (< 100)
         0  Min Q too low (<0) (0.00%)
      1247  Staggered pairs (0.29%) merged & trimmed
     54.19  Mean alignment length
    444.14  Mean merged length
      1.07  Mean fwd expected errors
      1.72  Mean rev expected errors
      1.07  Mean merged expected errors
-----
  ‣ HG091-R1 - Merging Summary
    Merging Rate: 153917 / 210920 (73.0%)
    Median Merged Length: 311

    210920  Pairs (210.9k)
    153917  Merged (153.9k, 72.97%)
     81977  Alignments with zero diffs (38.87%)
     14485  Too many diffs (> 10) (6.87%)
       577  Fwd too short (< 64) after tail trimming (0.27%)
      3503  Rev too short (< 64) after tail trimming (1.66%)
     38270  No alignment found (18.14%)
       151  Alignment too short (< 20) (0.07%)
        17  Merged too short (< 100)
         0  Min Q too low (<0) (0.00%)
      3901  Staggered pairs (1.85%) merged & trimmed
    110.44  Mean alignment length
    351.86  Mean merged length
      0.49  Mean fwd expected errors
      1.07  Mean rev expected errors
      0.43  Mean merged expected errors
-----
  ‣ HG091-R2 - Merging Summary
    Merging Rate: 131980 / 170809 (77.3%)
    Median Merged Length: 471

    170809  Pairs (170.8k)
    131980  Merged (132.0k, 77.27%)
     41529  Alignments with zero diffs (24.31%)
     15675  Too many diffs (> 10) (9.18%)
        25  Fwd too short (< 64) after tail trimming (0.01%)
```

```
       676  Rev too short (< 64) after tail trimming (0.40%)
     22449  No alignment found (13.14%)
         3  Alignment too short (< 20) (0.00%)
         1  Merged too short (< 100)
         0  Min Q too low (<0) (0.00%)
      2537  Staggered pairs (1.49%) merged & trimmed
     81.03  Mean alignment length
    400.71  Mean merged length
      1.06  Mean fwd expected errors
      1.71  Mean rev expected errors
      0.96  Mean merged expected errors
-----
  ▸ HG091-R3 - Merging Summary
    Merging Rate: 205074 / 242611 (84.5%)
    Median Merged Length: 449

    242611  Pairs (242.6k)
    205074  Merged (205.1k, 84.53%)
     58913  Alignments with zero diffs (24.28%)
     12505  Too many diffs (> 10) (5.15%)
       146  Fwd too short (< 64) after tail trimming (0.06%)
       388  Rev too short (< 64) after tail trimming (0.16%)
     24373  No alignment found (10.05%)
       124  Alignment too short (< 20) (0.05%)
         1  Merged too short (< 100)
         0  Min Q too low (<0) (0.00%)
      1204  Staggered pairs (0.50%) merged & trimmed
     60.11  Mean alignment length
    434.28  Mean merged length
      1.16  Mean fwd expected errors
      1.55  Mean rev expected errors
      0.99  Mean merged expected errors
-----
  ▸ HG141-B1 - Merging Summary
    Merging Rate: 131003 / 136308 (96.1%)
    Median Merged Length: 472

    136308  Pairs (136.3k)
    131003  Merged (131.0k, 96.11%)
     92413  Alignments with zero diffs (67.80%)
       346  Too many diffs (> 10) (0.25%)
        92  Fwd too short (< 64) after tail trimming (0.07%)
       296  Rev too short (< 64) after tail trimming (0.22%)
      4464  No alignment found (3.27%)
        64  Alignment too short (< 20) (0.05%)
        43  Merged too short (< 100)
         0  Min Q too low (<0) (0.00%)
       229  Staggered pairs (0.17%) merged & trimmed
     32.39  Mean alignment length
    466.90  Mean merged length
      0.53  Mean fwd expected errors
      0.74  Mean rev expected errors
      0.81  Mean merged expected errors
-----
```

```
  ‣ HG141-B2 - Merging Summary
    Merging Rate: 247591 / 263016 (94.1%)
    Median Merged Length: 471

    263016  Pairs (263.0k)
    247591  Merged (247.6k, 94.14%)
     91389  Alignments with zero diffs (34.75%)
      4333  Too many diffs (> 10) (1.65%)
         2  Fwd too short (< 64) after tail trimming (0.00%)
        35  Rev too short (< 64) after tail trimming (0.01%)
     11052  No alignment found (4.20%)
         3  Alignment too short (< 20) (0.00%)
         0  Merged too short (< 100)
         0  Min Q too low (<0) (0.00%)
        77  Staggered pairs (0.03%) merged & trimmed
     38.56  Mean alignment length
    462.84  Mean merged length
      1.12  Mean fwd expected errors
      1.37  Mean rev expected errors
      1.19  Mean merged expected errors
-----
  ‣ HG141-B3 - Merging Summary
    Merging Rate: 310455 / 324993 (95.5%)
    Median Merged Length: 472

    324993  Pairs (325.0k)
    310455  Merged (310.5k, 95.53%)
    137382  Alignments with zero diffs (42.27%)
      2405  Too many diffs (> 10) (0.74%)
         0  Fwd too short (< 64) after tail trimming (0.00%)
         4  Rev too short (< 64) after tail trimming (0.00%)
     12120  No alignment found (3.73%)
         9  Alignment too short (< 20) (0.00%)
         0  Merged too short (< 100)
         0  Min Q too low (<0) (0.00%)
        33  Staggered pairs (0.01%) merged & trimmed
     34.03  Mean alignment length
    467.56  Mean merged length
      1.02  Mean fwd expected errors
      1.14  Mean rev expected errors
      1.13  Mean merged expected errors
-----
  ‣ HG141-D1 - Merging Summary
    Merging Rate: 51044 / 53219 (95.9%)
    Median Merged Length: 472

     53219  Pairs (53.2k)
     51044  Merged (51.0k, 95.91%)
     31792  Alignments with zero diffs (59.74%)
       424  Too many diffs (> 10) (0.80%)
        31  Fwd too short (< 64) after tail trimming (0.06%)
       137  Rev too short (< 64) after tail trimming (0.26%)
      1564  No alignment found (2.94%)
         8  Alignment too short (< 20) (0.02%)
```

```
        11  Merged too short (< 100)
         0  Min Q too low (<0) (0.00%)
        66  Staggered pairs (0.12%) merged & trimmed
     36.98  Mean alignment length
    463.15  Mean merged length
      0.57  Mean fwd expected errors
      0.94  Mean rev expected errors
      0.87  Mean merged expected errors
-----
  ▸ HG141-D2 - Merging Summary
    Merging Rate: 373771 / 392148 (95.3%)
    Median Merged Length: 472

    392148  Pairs (392.1k)
    373771  Merged (373.8k, 95.31%)
    157607  Alignments with zero diffs (40.19%)
       942  Too many diffs (> 10) (0.24%)
         2  Fwd too short (< 64) after tail trimming (0.00%)
        28  Rev too short (< 64) after tail trimming (0.01%)
     17399  No alignment found (4.44%)
         6  Alignment too short (< 20) (0.00%)
         0  Merged too short (< 100)
         0  Min Q too low (<0) (0.00%)
        89  Staggered pairs (0.02%) merged & trimmed
     30.81  Mean alignment length
    470.60  Mean merged length
      1.31  Mean fwd expected errors
      1.14  Mean rev expected errors
      1.38  Mean merged expected errors
-----
  ▸ HG141-D3 - Merging Summary
    Merging Rate: 235294 / 250505 (93.9%)
    Median Merged Length: 453

    250505  Pairs (250.5k)
    235294  Merged (235.3k, 93.93%)
     78032  Alignments with zero diffs (31.15%)
      6458  Too many diffs (> 10) (2.58%)
         0  Fwd too short (< 64) after tail trimming (0.00%)
         2  Rev too short (< 64) after tail trimming (0.00%)
      8746  No alignment found (3.49%)
         5  Alignment too short (< 20) (0.00%)
         0  Merged too short (< 100)
         0  Min Q too low (<0) (0.00%)
        32  Staggered pairs (0.01%) merged & trimmed
     43.27  Mean alignment length
    458.31  Mean merged length
      1.05  Mean fwd expected errors
      1.55  Mean rev expected errors
      1.13  Mean merged expected errors
-----
  ▸ HG141-I1 - Merging Summary
    Merging Rate: 124181 / 128784 (96.4%)
    Median Merged Length: 472
```

```
    128784  Pairs (128.8k)
    124181  Merged (124.2k, 96.43%)
     83139  Alignments with zero diffs (64.56%)
       755  Too many diffs (> 10) (0.59%)
        66  Fwd too short (< 64) after tail trimming (0.05%)
       358  Rev too short (< 64) after tail trimming (0.28%)
      3386  No alignment found (2.63%)
        24  Alignment too short (< 20) (0.02%)
        14  Merged too short (< 100)
         0  Min Q too low (<0) (0.00%)
       110  Staggered pairs (0.09%) merged & trimmed
     34.79  Mean alignment length
    465.74  Mean merged length
      0.56  Mean fwd expected errors
      0.78  Mean rev expected errors
      0.80  Mean merged expected errors
-----
  ‣ HG141-I2 - Merging Summary
    Merging Rate: 253459 / 261246 (97.0%)
    Median Merged Length: 472

    261246  Pairs (261.2k)
    253459  Merged (253.5k, 97.02%)
    177729  Alignments with zero diffs (68.03%)
       752  Too many diffs (> 10) (0.29%)
         3  Fwd too short (< 64) after tail trimming (0.00%)
        85  Rev too short (< 64) after tail trimming (0.03%)
      6945  No alignment found (2.66%)
         2  Alignment too short (< 20) (0.00%)
         0  Merged too short (< 100)
         0  Min Q too low (<0) (0.00%)
       187  Staggered pairs (0.07%) merged & trimmed
     32.62  Mean alignment length
    467.90  Mean merged length
      0.51  Mean fwd expected errors
      0.75  Mean rev expected errors
      0.81  Mean merged expected errors
-----
  ‣ HG141-I3 - Merging Summary
    Merging Rate: 163563 / 173242 (94.4%)
    Median Merged Length: 472

    173242  Pairs (173.2k)
    163563  Merged (163.6k, 94.41%)
     73771  Alignments with zero diffs (42.58%)
       899  Too many diffs (> 10) (0.52%)
         0  Fwd too short (< 64) after tail trimming (0.00%)
         5  Rev too short (< 64) after tail trimming (0.00%)
      8771  No alignment found (5.06%)
         4  Alignment too short (< 20) (0.00%)
         0  Merged too short (< 100)
         0  Min Q too low (<0) (0.00%)
        46  Staggered pairs (0.03%) merged & trimmed
```

```
     32.84  Mean alignment length
    468.48  Mean merged length
      1.04  Mean fwd expected errors
      1.13  Mean rev expected errors
      1.18  Mean merged expected errors
-----
  ▸ HG141-J1 - Merging Summary
    Merging Rate: 173717 / 181431 (95.7%)
    Median Merged Length: 472

    181431  Pairs (181.4k)
    173717  Merged (173.7k, 95.75%)
    119542  Alignments with zero diffs (65.89%)
       652  Too many diffs (> 10) (0.36%)
       197  Fwd too short (< 64) after tail trimming (0.11%)
      1115  Rev too short (< 64) after tail trimming (0.61%)
      5724  No alignment found (3.15%)
        11  Alignment too short (< 20) (0.01%)
        15  Merged too short (< 100)
         0  Min Q too low (<0) (0.00%)
       417  Staggered pairs (0.23%) merged & trimmed
     32.76  Mean alignment length
    466.20  Mean merged length
      0.54  Mean fwd expected errors
      0.81  Mean rev expected errors
      0.85  Mean merged expected errors
-----
  ▸ HG141-J2 - Merging Summary
    Merging Rate: 333157 / 357689 (93.1%)
    Median Merged Length: 472

    357689  Pairs (357.7k)
    333157  Merged (333.2k, 93.14%)
    219859  Alignments with zero diffs (61.47%)
      4505  Too many diffs (> 10) (1.26%)
        15  Fwd too short (< 64) after tail trimming (0.00%)
       144  Rev too short (< 64) after tail trimming (0.04%)
     19799  No alignment found (5.54%)
        69  Alignment too short (< 20) (0.02%)
         0  Merged too short (< 100)
         0  Min Q too low (<0) (0.00%)
       569  Staggered pairs (0.16%) merged & trimmed
     42.81  Mean alignment length
    455.38  Mean merged length
      0.58  Mean fwd expected errors
      0.80  Mean rev expected errors
      0.76  Mean merged expected errors
-----
  ▸ HG141-J3 - Merging Summary
    Merging Rate: 251155 / 286374 (87.7%)
    Median Merged Length: 449

    286374  Pairs (286.4k)
    251155  Merged (251.2k, 87.70%)
```

```
    76521  Alignments with zero diffs (26.72%)
    13267  Too many diffs (> 10) (4.63%)
       31  Fwd too short (< 64) after tail trimming (0.01%)
      223  Rev too short (< 64) after tail trimming (0.08%)
    21656  No alignment found (7.56%)
       42  Alignment too short (< 20) (0.01%)
        0  Merged too short (< 100)
        0  Min Q too low (<0) (0.00%)
      569  Staggered pairs (0.20%) merged & trimmed
    58.81  Mean alignment length
   439.58  Mean merged length
     1.08  Mean fwd expected errors
     1.59  Mean rev expected errors
     1.00  Mean merged expected errors
-----
  ‣ HG141-L1 - Merging Summary
    Merging Rate: 241595 / 250069 (96.6%)
    Median Merged Length: 472

   250069  Pairs (250.1k)
   241595  Merged (241.6k, 96.61%)
   163410  Alignments with zero diffs (65.35%)
     1491  Too many diffs (> 10) (0.60%)
      253  Fwd too short (< 64) after tail trimming (0.10%)
      943  Rev too short (< 64) after tail trimming (0.38%)
     5755  No alignment found (2.30%)
        7  Alignment too short (< 20) (0.00%)
       25  Merged too short (< 100)
        0  Min Q too low (<0) (0.00%)
      324  Staggered pairs (0.13%) merged & trimmed
    34.41  Mean alignment length
   465.94  Mean merged length
     0.53  Mean fwd expected errors
     0.77  Mean rev expected errors
     0.79  Mean merged expected errors
-----
  ‣ HG141-L2 - Merging Summary
    Merging Rate: 284689 / 295851 (96.2%)
    Median Merged Length: 472

   295851  Pairs (295.9k)
   284689  Merged (284.7k, 96.23%)
   176321  Alignments with zero diffs (59.60%)
     3015  Too many diffs (> 10) (1.02%)
        2  Fwd too short (< 64) after tail trimming (0.00%)
       55  Rev too short (< 64) after tail trimming (0.02%)
     8088  No alignment found (2.73%)
        2  Alignment too short (< 20) (0.00%)
        0  Merged too short (< 100)
        0  Min Q too low (<0) (0.00%)
      143  Staggered pairs (0.05%) merged & trimmed
    38.66  Mean alignment length
   462.35  Mean merged length
     0.59  Mean fwd expected errors
```

```
      0.89  Mean rev expected errors
      0.83  Mean merged expected errors
-----
  ‣ HG141-L3 - Merging Summary
    Merging Rate: 154380 / 162187 (95.2%)
    Median Merged Length: 472

    162187  Pairs (162.2k)
    154380  Merged (154.4k, 95.19%)
     69493  Alignments with zero diffs (42.85%)
       930  Too many diffs (> 10) (0.57%)
         0  Fwd too short (< 64) after tail trimming (0.00%)
        24  Rev too short (< 64) after tail trimming (0.01%)
      6850  No alignment found (4.22%)
         3  Alignment too short (< 20) (0.00%)
         0  Merged too short (< 100)
         0  Min Q too low (<0) (0.00%)
        55  Staggered pairs (0.03%) merged & trimmed
     32.57  Mean alignment length
    468.61  Mean merged length
      1.12  Mean fwd expected errors
      1.07  Mean rev expected errors
      1.19  Mean merged expected errors
-----
  ‣ HG141-O1 - Merging Summary
    Merging Rate: 625137 / 650898 (96.0%)
    Median Merged Length: 451

    650898  Pairs (650.9k)
    625137  Merged (625.1k, 96.04%)
    347800  Alignments with zero diffs (53.43%)
     10467  Too many diffs (> 10) (1.61%)
        29  Fwd too short (< 64) after tail trimming (0.00%)
       399  Rev too short (< 64) after tail trimming (0.06%)
     14859  No alignment found (2.28%)
         7  Alignment too short (< 20) (0.00%)
         0  Merged too short (< 100)
         0  Min Q too low (<0) (0.00%)
       123  Staggered pairs (0.02%) merged & trimmed
     44.59  Mean alignment length
    456.83  Mean merged length
      0.63  Mean fwd expected errors
      0.95  Mean rev expected errors
      0.78  Mean merged expected errors
-----
  ‣ HG141-O2 - Merging Summary
    Merging Rate: 240269 / 253469 (94.8%)
    Median Merged Length: 472

    253469  Pairs (253.5k)
    240269  Merged (240.3k, 94.79%)
    107086  Alignments with zero diffs (42.25%)
      1634  Too many diffs (> 10) (0.64%)
         6  Fwd too short (< 64) after tail trimming (0.00%)
```

```
        68  Rev too short (< 64) after tail trimming (0.03%)
     11490  No alignment found (4.53%)
         2  Alignment too short (< 20) (0.00%)
         0  Merged too short (< 100)
         0  Min Q too low (<0) (0.00%)
       199  Staggered pairs (0.08%) merged & trimmed
     33.10  Mean alignment length
    467.58  Mean merged length
      1.00  Mean fwd expected errors
      1.18  Mean rev expected errors
      1.19  Mean merged expected errors
-----
  ‣ HG141-O3 - Merging Summary
    Merging Rate: 213670 / 223761 (95.5%)
    Median Merged Length: 472

    223761  Pairs (223.8k)
    213670  Merged (213.7k, 95.49%)
     94193  Alignments with zero diffs (42.10%)
       739  Too many diffs (> 10) (0.33%)
         2  Fwd too short (< 64) after tail trimming (0.00%)
        51  Rev too short (< 64) after tail trimming (0.02%)
      9298  No alignment found (4.16%)
         1  Alignment too short (< 20) (0.00%)
         0  Merged too short (< 100)
         0  Min Q too low (<0) (0.00%)
       122  Staggered pairs (0.05%) merged & trimmed
     31.56  Mean alignment length
    469.42  Mean merged length
      1.18  Mean fwd expected errors
      1.11  Mean rev expected errors
      1.26  Mean merged expected errors
-----
  ‣ HG141-Q1 - Merging Summary
    Merging Rate: 197970 / 205832 (96.2%)
    Median Merged Length: 472

    205832  Pairs (205.8k)
    197970  Merged (198.0k, 96.18%)
    134252  Alignments with zero diffs (65.22%)
      1176  Too many diffs (> 10) (0.57%)
        41  Fwd too short (< 64) after tail trimming (0.02%)
       434  Rev too short (< 64) after tail trimming (0.21%)
      6197  No alignment found (3.01%)
        13  Alignment too short (< 20) (0.01%)
         1  Merged too short (< 100)
         0  Min Q too low (<0) (0.00%)
       253  Staggered pairs (0.12%) merged & trimmed
     35.90  Mean alignment length
    463.67  Mean merged length
      0.55  Mean fwd expected errors
      0.75  Mean rev expected errors
      0.77  Mean merged expected errors
-----
```

```
▸ HG141-Q2 - Merging Summary
  Merging Rate: 137016 / 145171 (94.4%)
  Median Merged Length: 472

  145171  Pairs (145.2k)
  137016  Merged (137.0k, 94.38%)
   52715  Alignments with zero diffs (36.31%)
    1029  Too many diffs (> 10) (0.71%)
       2  Fwd too short (< 64) after tail trimming (0.00%)
      12  Rev too short (< 64) after tail trimming (0.01%)
    7112  No alignment found (4.90%)
       0  Alignment too short (< 20) (0.00%)
       0  Merged too short (< 100)
       0  Min Q too low (<0) (0.00%)
      60  Staggered pairs (0.04%) merged & trimmed
   32.25  Mean alignment length
  469.13  Mean merged length
    1.17  Mean fwd expected errors
    1.48  Mean rev expected errors
    1.47  Mean merged expected errors
-----
▸ HG141-Q3 - Merging Summary
  Merging Rate: 356604 / 380244 (93.8%)
  Median Merged Length: 472

  380244  Pairs (380.2k)
  356604  Merged (356.6k, 93.78%)
  155312  Alignments with zero diffs (40.85%)
    3982  Too many diffs (> 10) (1.05%)
       3  Fwd too short (< 64) after tail trimming (0.00%)
      80  Rev too short (< 64) after tail trimming (0.02%)
   19555  No alignment found (5.14%)
      19  Alignment too short (< 20) (0.00%)
       1  Merged too short (< 100)
       0  Min Q too low (<0) (0.00%)
     436  Staggered pairs (0.11%) merged & trimmed
   36.77  Mean alignment length
  463.44  Mean merged length
    1.03  Mean fwd expected errors
    1.15  Mean rev expected errors
    1.14  Mean merged expected errors
-----
▸ HG141-R1 - Merging Summary
  Merging Rate: 263587 / 274961 (95.9%)
  Median Merged Length: 472

  274961  Pairs (275.0k)
  263587  Merged (263.6k, 95.86%)
  183310  Alignments with zero diffs (66.67%)
    1532  Too many diffs (> 10) (0.56%)
      32  Fwd too short (< 64) after tail trimming (0.01%)
     390  Rev too short (< 64) after tail trimming (0.14%)
    9416  No alignment found (3.42%)
       3  Alignment too short (< 20) (0.00%)
```

```
         1  Merged too short (< 100)
         0  Min Q too low (<0) (0.00%)
       372  Staggered pairs (0.14%) merged & trimmed
     35.22  Mean alignment length
    464.22  Mean merged length
      0.54  Mean fwd expected errors
      0.72  Mean rev expected errors
      0.78  Mean merged expected errors
-----
  ▸ HG141-R2 - Merging Summary
    Merging Rate: 90300 / 113033 (79.9%)
    Median Merged Length: 454

    113033  Pairs (113.0k)
     90300  Merged (90.3k, 79.89%)
     30680  Alignments with zero diffs (27.14%)
      8393  Too many diffs (> 10) (7.43%)
        24  Fwd too short (< 64) after tail trimming (0.02%)
       119  Rev too short (< 64) after tail trimming (0.11%)
     14193  No alignment found (12.56%)
         3  Alignment too short (< 20) (0.00%)
         1  Merged too short (< 100)
         0  Min Q too low (<0) (0.00%)
       557  Staggered pairs (0.49%) merged & trimmed
     69.50  Mean alignment length
    425.42  Mean merged length
      1.08  Mean fwd expected errors
      1.60  Mean rev expected errors
      1.01  Mean merged expected errors
-----
  ▸ HG141-R3 - Merging Summary
    Merging Rate: 233044 / 249193 (93.5%)
    Median Merged Length: 453

    249193  Pairs (249.2k)
    233044  Merged (233.0k, 93.52%)
     83699  Alignments with zero diffs (33.59%)
      5734  Too many diffs (> 10) (2.30%)
         3  Fwd too short (< 64) after tail trimming (0.00%)
        16  Rev too short (< 64) after tail trimming (0.01%)
     10396  No alignment found (4.17%)
         0  Alignment too short (< 20) (0.00%)
         0  Merged too short (< 100)
         0  Min Q too low (<0) (0.00%)
        62  Staggered pairs (0.02%) merged & trimmed
     42.69  Mean alignment length
    458.75  Mean merged length
      1.02  Mean fwd expected errors
      1.41  Mean rev expected errors
      1.09  Mean merged expected errors
-----
  ▸ HG225-B1 - Merging Summary
    Merging Rate: 65077 / 69364 (93.8%)
    Median Merged Length: 472
```

```
     69364  Pairs (69.4k)
     65077  Merged (65.1k, 93.82%)
     45735  Alignments with zero diffs (65.93%)
       385  Too many diffs (> 10) (0.56%)
       168  Fwd too short (< 64) after tail trimming (0.24%)
       425  Rev too short (< 64) after tail trimming (0.61%)
      3206  No alignment found (4.62%)
        57  Alignment too short (< 20) (0.08%)
        46  Merged too short (< 100)
         0  Min Q too low (<0) (0.00%)
       321  Staggered pairs (0.46%) merged & trimmed
     36.53  Mean alignment length
    457.94  Mean merged length
      0.51  Mean fwd expected errors
      0.74  Mean rev expected errors
      0.76  Mean merged expected errors
-----
  ▸ HG225-B2 - Merging Summary
    Merging Rate: 304863 / 324118 (94.1%)
    Median Merged Length: 472

    324118  Pairs (324.1k)
    304863  Merged (304.9k, 94.06%)
    128215  Alignments with zero diffs (39.56%)
      3746  Too many diffs (> 10) (1.16%)
         3  Fwd too short (< 64) after tail trimming (0.00%)
        34  Rev too short (< 64) after tail trimming (0.01%)
     15462  No alignment found (4.77%)
        10  Alignment too short (< 20) (0.00%)
         0  Merged too short (< 100)
         0  Min Q too low (<0) (0.00%)
       441  Staggered pairs (0.14%) merged & trimmed
     36.98  Mean alignment length
    463.09  Mean merged length
      1.10  Mean fwd expected errors
      1.15  Mean rev expected errors
      1.14  Mean merged expected errors
-----
  ▸ HG225-B3 - Merging Summary
    Merging Rate: 239744 / 258166 (92.9%)
    Median Merged Length: 449

    258166  Pairs (258.2k)
    239744  Merged (239.7k, 92.86%)
     67345  Alignments with zero diffs (26.09%)
      9346  Too many diffs (> 10) (3.62%)
         0  Fwd too short (< 64) after tail trimming (0.00%)
         5  Rev too short (< 64) after tail trimming (0.00%)
      9071  No alignment found (3.51%)
         0  Alignment too short (< 20) (0.00%)
         0  Merged too short (< 100)
         0  Min Q too low (<0) (0.00%)
        79  Staggered pairs (0.03%) merged & trimmed
```

```
     48.94  Mean alignment length
    452.44  Mean merged length
      1.12  Mean fwd expected errors
      1.63  Mean rev expected errors
      1.09  Mean merged expected errors
-----
  ▸ HG225-D1 - Merging Summary
    Merging Rate: 58065 / 61429 (94.5%)
    Median Merged Length: 472

     61429  Pairs (61.4k)
     58065  Merged (58.1k, 94.52%)
     40698  Alignments with zero diffs (66.25%)
       306  Too many diffs (> 10) (0.50%)
       173  Fwd too short (< 64) after tail trimming (0.28%)
       535  Rev too short (< 64) after tail trimming (0.87%)
      2314  No alignment found (3.77%)
        17  Alignment too short (< 20) (0.03%)
        19  Merged too short (< 100)
         0  Min Q too low (<0) (0.00%)
       208  Staggered pairs (0.34%) merged & trimmed
     36.07  Mean alignment length
    459.24  Mean merged length
      0.53  Mean fwd expected errors
      0.73  Mean rev expected errors
      0.77  Mean merged expected errors
-----
  ▸ HG225-D2 - Merging Summary
    Merging Rate: 345291 / 368629 (93.7%)
    Median Merged Length: 472

    368629  Pairs (368.6k)
    345291  Merged (345.3k, 93.67%)
    142652  Alignments with zero diffs (38.70%)
      5763  Too many diffs (> 10) (1.56%)
         7  Fwd too short (< 64) after tail trimming (0.00%)
       143  Rev too short (< 64) after tail trimming (0.04%)
     17412  No alignment found (4.72%)
        11  Alignment too short (< 20) (0.00%)
         2  Merged too short (< 100)
         0  Min Q too low (<0) (0.00%)
       338  Staggered pairs (0.09%) merged & trimmed
     37.03  Mean alignment length
    463.31  Mean merged length
      1.12  Mean fwd expected errors
      1.21  Mean rev expected errors
      1.16  Mean merged expected errors
-----
  ▸ HG225-D3 - Merging Summary
    Merging Rate: 193719 / 208008 (93.1%)
    Median Merged Length: 449

    208008  Pairs (208.0k)
    193719  Merged (193.7k, 93.13%)
```

```
     52657  Alignments with zero diffs (25.31%)
      7550  Too many diffs (> 10) (3.63%)
         0  Fwd too short (< 64) after tail trimming (0.00%)
         3  Rev too short (< 64) after tail trimming (0.00%)
      6736  No alignment found (3.24%)
         0  Alignment too short (< 20) (0.00%)
         0  Merged too short (< 100)
         0  Min Q too low (<0) (0.00%)
        23  Staggered pairs (0.01%) merged & trimmed
     51.10  Mean alignment length
    450.53  Mean merged length
      1.09  Mean fwd expected errors
      1.63  Mean rev expected errors
      1.02  Mean merged expected errors
-----
  ‣ HG225-I1 - Merging Summary
    Merging Rate: 66187 / 73519 (90.0%)
    Median Merged Length: 449

     73519  Pairs (73.5k)
     66187  Merged (66.2k, 90.03%)
     32004  Alignments with zero diffs (43.53%)
      2037  Too many diffs (> 10) (2.77%)
       241  Fwd too short (< 64) after tail trimming (0.33%)
      1198  Rev too short (< 64) after tail trimming (1.63%)
      3805  No alignment found (5.18%)
        31  Alignment too short (< 20) (0.04%)
        20  Merged too short (< 100)
         0  Min Q too low (<0) (0.00%)
       412  Staggered pairs (0.56%) merged & trimmed
     58.78  Mean alignment length
    433.66  Mean merged length
      0.64  Mean fwd expected errors
      1.06  Mean rev expected errors
      0.72  Mean merged expected errors
-----
  ‣ HG225-I2 - Merging Summary
    Merging Rate: 223259 / 253431 (88.1%)
    Median Merged Length: 451

    253431  Pairs (253.4k)
    223259  Merged (223.3k, 88.09%)
    114729  Alignments with zero diffs (45.27%)
      7989  Too many diffs (> 10) (3.15%)
        13  Fwd too short (< 64) after tail trimming (0.01%)
       128  Rev too short (< 64) after tail trimming (0.05%)
     22010  No alignment found (8.68%)
        32  Alignment too short (< 20) (0.01%)
         0  Merged too short (< 100)
         0  Min Q too low (<0) (0.00%)
      1135  Staggered pairs (0.45%) merged & trimmed
     60.30  Mean alignment length
    433.44  Mean merged length
      0.59  Mean fwd expected errors
```

```
      1.11  Mean rev expected errors
      0.76  Mean merged expected errors
-----
  ▸ HG225-I3 - Merging Summary
    Merging Rate: 182835 / 196213 (93.2%)
    Median Merged Length: 472

    196213  Pairs (196.2k)
    182835  Merged (182.8k, 93.18%)
     72824  Alignments with zero diffs (37.11%)
      2197  Too many diffs (> 10) (1.12%)
         0  Fwd too short (< 64) after tail trimming (0.00%)
        11  Rev too short (< 64) after tail trimming (0.01%)
     11167  No alignment found (5.69%)
         3  Alignment too short (< 20) (0.00%)
         0  Merged too short (< 100)
         0  Min Q too low (<0) (0.00%)
       101  Staggered pairs (0.05%) merged & trimmed
     35.49  Mean alignment length
    465.59  Mean merged length
      1.08  Mean fwd expected errors
      1.32  Mean rev expected errors
      1.22  Mean merged expected errors
-----
  ▸ HG225-J1 - Merging Summary
    Merging Rate: 30091 / 37684 (79.9%)
    Median Merged Length: 471

     37684  Pairs (37.7k)
     30091  Merged (30.1k, 79.85%)
     17920  Alignments with zero diffs (47.55%)
      1348  Too many diffs (> 10) (3.58%)
       385  Fwd too short (< 64) after tail trimming (1.02%)
      1630  Rev too short (< 64) after tail trimming (4.33%)
      4148  No alignment found (11.01%)
        49  Alignment too short (< 20) (0.13%)
        33  Merged too short (< 100)
         0  Min Q too low (<0) (0.00%)
       495  Staggered pairs (1.31%) merged & trimmed
     65.32  Mean alignment length
    412.75  Mean merged length
      0.55  Mean fwd expected errors
      0.96  Mean rev expected errors
      0.66  Mean merged expected errors
-----
  ▸ HG225-J2 - Merging Summary
    Merging Rate: 352287 / 394987 (89.2%)
    Median Merged Length: 472

    394987  Pairs (395.0k)
    352287  Merged (352.3k, 89.19%)
    236496  Alignments with zero diffs (59.87%)
      6177  Too many diffs (> 10) (1.56%)
         8  Fwd too short (< 64) after tail trimming (0.00%)
```

```
      118  Rev too short (< 64) after tail trimming (0.03%)
    36222  No alignment found (9.17%)
      175  Alignment too short (< 20) (0.04%)
        0  Merged too short (< 100)
        0  Min Q too low (<0) (0.00%)
     1262  Staggered pairs (0.32%) merged & trimmed
    48.46  Mean alignment length
   447.27  Mean merged length
     0.50  Mean fwd expected errors
     0.79  Mean rev expected errors
     0.73  Mean merged expected errors
-----
  ‣ HG225-J3 - Merging Summary
    Merging Rate: 188923 / 221433 (85.3%)
    Median Merged Length: 449

   221433  Pairs (221.4k)
   188923  Merged (188.9k, 85.32%)
    59893  Alignments with zero diffs (27.05%)
    10207  Too many diffs (> 10) (4.61%)
        0  Fwd too short (< 64) after tail trimming (0.00%)
       30  Rev too short (< 64) after tail trimming (0.01%)
    22248  No alignment found (10.05%)
       25  Alignment too short (< 20) (0.01%)
        0  Merged too short (< 100)
        0  Min Q too low (<0) (0.00%)
      713  Staggered pairs (0.32%) merged & trimmed
    56.90  Mean alignment length
   440.28  Mean merged length
     1.07  Mean fwd expected errors
     1.56  Mean rev expected errors
     1.03  Mean merged expected errors
-----
  ‣ HG225-L1 - Merging Summary
    Merging Rate: 335020 / 344679 (97.2%)
    Median Merged Length: 472

   344679  Pairs (344.7k)
   335020  Merged (335.0k, 97.20%)
   237472  Alignments with zero diffs (68.90%)
      509  Too many diffs (> 10) (0.15%)
      213  Fwd too short (< 64) after tail trimming (0.06%)
     1401  Rev too short (< 64) after tail trimming (0.41%)
     7505  No alignment found (2.18%)
       13  Alignment too short (< 20) (0.00%)
       18  Merged too short (< 100)
        0  Min Q too low (<0) (0.00%)
      379  Staggered pairs (0.11%) merged & trimmed
    30.80  Mean alignment length
   469.48  Mean merged length
     0.54  Mean fwd expected errors
     0.74  Mean rev expected errors
     0.83  Mean merged expected errors
-----
```

```
  ‣ HG225-L2 - Merging Summary
    Merging Rate: 311631 / 327885 (95.0%)
    Median Merged Length: 449

    327885  Pairs (327.9k)
    311631  Merged (311.6k, 95.04%)
    137194  Alignments with zero diffs (41.84%)
      8092  Too many diffs (> 10) (2.47%)
         2  Fwd too short (< 64) after tail trimming (0.00%)
        31  Rev too short (< 64) after tail trimming (0.01%)
      8128  No alignment found (2.48%)
         1  Alignment too short (< 20) (0.00%)
         0  Merged too short (< 100)
         0  Min Q too low (<0) (0.00%)
       118  Staggered pairs (0.04%) merged & trimmed
     53.04  Mean alignment length
    448.13  Mean merged length
      0.64  Mean fwd expected errors
      1.20  Mean rev expected errors
      0.80  Mean merged expected errors
-----
  ‣ HG225-L3 - Merging Summary
    Merging Rate: 157772 / 171936 (91.8%)
    Median Merged Length: 472

    171936  Pairs (171.9k)
    157772  Merged (157.8k, 91.76%)
     65309  Alignments with zero diffs (37.98%)
      2500  Too many diffs (> 10) (1.45%)
         4  Fwd too short (< 64) after tail trimming (0.00%)
        24  Rev too short (< 64) after tail trimming (0.01%)
     11628  No alignment found (6.76%)
         8  Alignment too short (< 20) (0.00%)
         0  Merged too short (< 100)
         0  Min Q too low (<0) (0.00%)
       230  Staggered pairs (0.13%) merged & trimmed
     36.96  Mean alignment length
    463.19  Mean merged length
      1.08  Mean fwd expected errors
      1.30  Mean rev expected errors
      1.24  Mean merged expected errors
-----
  ‣ HG225-O1 - Merging Summary
    Merging Rate: 278661 / 287427 (97.0%)
    Median Merged Length: 472

    287427  Pairs (287.4k)
    278661  Merged (278.7k, 96.95%)
    199907  Alignments with zero diffs (69.55%)
       588  Too many diffs (> 10) (0.20%)
        11  Fwd too short (< 64) after tail trimming (0.00%)
       188  Rev too short (< 64) after tail trimming (0.07%)
      7968  No alignment found (2.77%)
         7  Alignment too short (< 20) (0.00%)
```

```
         4  Merged too short (< 100)
         0  Min Q too low (<0) (0.00%)
       178  Staggered pairs (0.06%) merged & trimmed
     31.96  Mean alignment length
    468.68  Mean merged length
      0.54  Mean fwd expected errors
      0.67  Mean rev expected errors
      0.77  Mean merged expected errors
-----
  ‣ HG225-O2 - Merging Summary
    Merging Rate: 69679 / 102381 (68.1%)
    Median Merged Length: 336

    102381  Pairs (102.4k)
     69679  Merged (69.7k, 68.06%)
     25536  Alignments with zero diffs (24.94%)
      9688  Too many diffs (> 10) (9.46%)
        73  Fwd too short (< 64) after tail trimming (0.07%)
       823  Rev too short (< 64) after tail trimming (0.80%)
     22096  No alignment found (21.58%)
        20  Alignment too short (< 20) (0.02%)
         2  Merged too short (< 100)
         0  Min Q too low (<0) (0.00%)
      2269  Staggered pairs (2.22%) merged & trimmed
    106.10  Mean alignment length
    351.61  Mean merged length
      0.82  Mean fwd expected errors
      1.57  Mean rev expected errors
      0.62  Mean merged expected errors
-----
  ‣ HG225-O3 - Merging Summary
    Merging Rate: 147940 / 159271 (92.9%)
    Median Merged Length: 472

    159271  Pairs (159.3k)
    147940  Merged (147.9k, 92.89%)
     54457  Alignments with zero diffs (34.19%)
      2674  Too many diffs (> 10) (1.68%)
         4  Fwd too short (< 64) after tail trimming (0.00%)
        58  Rev too short (< 64) after tail trimming (0.04%)
      8586  No alignment found (5.39%)
         9  Alignment too short (< 20) (0.01%)
         0  Merged too short (< 100)
         0  Min Q too low (<0) (0.00%)
       183  Staggered pairs (0.11%) merged & trimmed
     37.63  Mean alignment length
    462.80  Mean merged length
      1.10  Mean fwd expected errors
      1.47  Mean rev expected errors
      1.29  Mean merged expected errors
-----
  ‣ HG225-Q1 - Merging Summary
    Merging Rate: 218365 / 226039 (96.6%)
    Median Merged Length: 472
```

```
   226039  Pairs (226.0k)
   218365  Merged (218.4k, 96.61%)
   141341  Alignments with zero diffs (62.53%)
     1680  Too many diffs (> 10) (0.74%)
       18  Fwd too short (< 64) after tail trimming (0.01%)
      169  Rev too short (< 64) after tail trimming (0.07%)
     5807  No alignment found (2.57%)
        0  Alignment too short (< 20) (0.00%)
        0  Merged too short (< 100)
        0  Min Q too low (<0) (0.00%)
      109  Staggered pairs (0.05%) merged & trimmed
    35.33  Mean alignment length
   465.12  Mean merged length
     0.61  Mean fwd expected errors
     0.85  Mean rev expected errors
     0.84  Mean merged expected errors
-----
  ‣ HG225-Q2 - Merging Summary
   Merging Rate: 143106 / 186263 (76.8%)
   Median Merged Length: 449

   186263  Pairs (186.3k)
   143106  Merged (143.1k, 76.83%)
    42645  Alignments with zero diffs (22.90%)
    12831  Too many diffs (> 10) (6.89%)
       21  Fwd too short (< 64) after tail trimming (0.01%)
      122  Rev too short (< 64) after tail trimming (0.07%)
    30171  No alignment found (16.20%)
       10  Alignment too short (< 20) (0.01%)
        2  Merged too short (< 100)
        0  Min Q too low (<0) (0.00%)
     1200  Staggered pairs (0.64%) merged & trimmed
    73.15  Mean alignment length
   419.46  Mean merged length
     1.08  Mean fwd expected errors
     1.65  Mean rev expected errors
     0.94  Mean merged expected errors
-----
  ‣ HG225-Q3 - Merging Summary
   Merging Rate: 94593 / 110670 (85.5%)
   Median Merged Length: 449

   110670  Pairs (110.7k)
    94593  Merged (94.6k, 85.47%)
    27565  Alignments with zero diffs (24.91%)
     6091  Too many diffs (> 10) (5.50%)
      389  Fwd too short (< 64) after tail trimming (0.35%)
      559  Rev too short (< 64) after tail trimming (0.51%)
     9017  No alignment found (8.15%)
       18  Alignment too short (< 20) (0.02%)
        3  Merged too short (< 100)
        0  Min Q too low (<0) (0.00%)
      726  Staggered pairs (0.66%) merged & trimmed
```

```
    64.91  Mean alignment length
   427.42  Mean merged length
     0.95  Mean fwd expected errors
     1.67  Mean rev expected errors
     0.87  Mean merged expected errors
-----
  ‣ HG225-R1 - Merging Summary
    Merging Rate: 192002 / 200820 (95.6%)
    Median Merged Length: 449

   200820  Pairs (200.8k)
   192002  Merged (192.0k, 95.61%)
    99121  Alignments with zero diffs (49.36%)
     3998  Too many diffs (> 10) (1.99%)
        1  Fwd too short (< 64) after tail trimming (0.00%)
       10  Rev too short (< 64) after tail trimming (0.00%)
     4809  No alignment found (2.39%)
        0  Alignment too short (< 20) (0.00%)
        0  Merged too short (< 100)
        0  Min Q too low (<0) (0.00%)
       33  Staggered pairs (0.02%) merged & trimmed
    49.31  Mean alignment length
   452.05  Mean merged length
     0.63  Mean fwd expected errors
     0.98  Mean rev expected errors
     0.75  Mean merged expected errors
-----
  ‣ HG225-R2 - Merging Summary
    Merging Rate: 278075 / 307229 (90.5%)
    Median Merged Length: 451

   307229  Pairs (307.2k)
   278075  Merged (278.1k, 90.51%)
    90335  Alignments with zero diffs (29.40%)
    11332  Too many diffs (> 10) (3.69%)
        1  Fwd too short (< 64) after tail trimming (0.00%)
       30  Rev too short (< 64) after tail trimming (0.01%)
    17779  No alignment found (5.79%)
       12  Alignment too short (< 20) (0.00%)
        0  Merged too short (< 100)
        0  Min Q too low (<0) (0.00%)
      259  Staggered pairs (0.08%) merged & trimmed
    46.19  Mean alignment length
   454.49  Mean merged length
     1.21  Mean fwd expected errors
     1.49  Mean rev expected errors
     1.18  Mean merged expected errors
-----
  ‣ HG225-R3 - Merging Summary
    Merging Rate: 27932 / 35570 (78.5%)
    Median Merged Length: 470

    35570  Pairs (35.6k)
    27932  Merged (27.9k, 78.53%)
```

```
     11388  Alignments with zero diffs (32.02%)
      1675  Too many diffs (> 10) (4.71%)
       315  Fwd too short (< 64) after tail trimming (0.89%)
       429  Rev too short (< 64) after tail trimming (1.21%)
      5184  No alignment found (14.57%)
        30  Alignment too short (< 20) (0.08%)
         5  Merged too short (< 100)
         0  Min Q too low (<0) (0.00%)
       459  Staggered pairs (1.29%) merged & trimmed
     64.91  Mean alignment length
    416.68  Mean merged length
      0.93  Mean fwd expected errors
      1.34  Mean rev expected errors
      0.91  Mean merged expected errors
-----
  ▸ HG319-B1 - Merging Summary
    Merging Rate: 125252 / 130294 (96.1%)
    Median Merged Length: 452

    130294  Pairs (130.3k)
    125252  Merged (125.3k, 96.13%)
     66309  Alignments with zero diffs (50.89%)
      1729  Too many diffs (> 10) (1.33%)
        70  Fwd too short (< 64) after tail trimming (0.05%)
       353  Rev too short (< 64) after tail trimming (0.27%)
      2852  No alignment found (2.19%)
        21  Alignment too short (< 20) (0.02%)
        17  Merged too short (< 100)
         0  Min Q too low (<0) (0.00%)
       174  Staggered pairs (0.13%) merged & trimmed
     46.88  Mean alignment length
    452.19  Mean merged length
      0.56  Mean fwd expected errors
      1.03  Mean rev expected errors
      0.76  Mean merged expected errors
-----
  ▸ HG319-B2 - Merging Summary
    Merging Rate: 203163 / 216773 (93.7%)
    Median Merged Length: 472

    216773  Pairs (216.8k)
    203163  Merged (203.2k, 93.72%)
     93547  Alignments with zero diffs (43.15%)
      1496  Too many diffs (> 10) (0.69%)
        12  Fwd too short (< 64) after tail trimming (0.01%)
       183  Rev too short (< 64) after tail trimming (0.08%)
     11907  No alignment found (5.49%)
        12  Alignment too short (< 20) (0.01%)
         0  Merged too short (< 100)
         0  Min Q too low (<0) (0.00%)
       456  Staggered pairs (0.21%) merged & trimmed
     34.69  Mean alignment length
    463.70  Mean merged length
      1.04  Mean fwd expected errors
```

```
      1.16  Mean rev expected errors
      1.18  Mean merged expected errors
-----
  ‣ HG319-B3 - Merging Summary
    Merging Rate: 313818 / 331297 (94.7%)
    Median Merged Length: 472

    331297  Pairs (331.3k)
    313818  Merged (313.8k, 94.72%)
    131419  Alignments with zero diffs (39.67%)
      3887  Too many diffs (> 10) (1.17%)
         0  Fwd too short (< 64) after tail trimming (0.00%)
         5  Rev too short (< 64) after tail trimming (0.00%)
     13584  No alignment found (4.10%)
         3  Alignment too short (< 20) (0.00%)
         0  Merged too short (< 100)
         0  Min Q too low (<0) (0.00%)
        45  Staggered pairs (0.01%) merged & trimmed
     35.57  Mean alignment length
    465.93  Mean merged length
      1.06  Mean fwd expected errors
      1.22  Mean rev expected errors
      1.16  Mean merged expected errors
-----
  ‣ HG319-D1 - Merging Summary
    Merging Rate: 92700 / 95790 (96.8%)
    Median Merged Length: 472

     95790  Pairs (95.8k)
     92700  Merged (92.7k, 96.77%)
     62709  Alignments with zero diffs (65.47%)
       325  Too many diffs (> 10) (0.34%)
        48  Fwd too short (< 64) after tail trimming (0.05%)
       228  Rev too short (< 64) after tail trimming (0.24%)
      2448  No alignment found (2.56%)
        23  Alignment too short (< 20) (0.02%)
        18  Merged too short (< 100)
         0  Min Q too low (<0) (0.00%)
       156  Staggered pairs (0.16%) merged & trimmed
     33.03  Mean alignment length
    466.10  Mean merged length
      0.55  Mean fwd expected errors
      0.86  Mean rev expected errors
      0.87  Mean merged expected errors
-----
  ‣ HG319-D2 - Merging Summary
    Merging Rate: 306162 / 330611 (92.6%)
    Median Merged Length: 472

    330611  Pairs (330.6k)
    306162  Merged (306.2k, 92.60%)
    140527  Alignments with zero diffs (42.51%)
      3393  Too many diffs (> 10) (1.03%)
        49  Fwd too short (< 64) after tail trimming (0.01%)
```

```
      588  Rev too short (< 64) after tail trimming (0.18%)
    20408  No alignment found (6.17%)
        5  Alignment too short (< 20) (0.00%)
        6  Merged too short (< 100)
        0  Min Q too low (<0) (0.00%)
     1092  Staggered pairs (0.33%) merged & trimmed
    37.86  Mean alignment length
   457.82  Mean merged length
     1.08  Mean fwd expected errors
     1.17  Mean rev expected errors
     1.15  Mean merged expected errors
-----
  ‣ HG319-D3 - Merging Summary
    Merging Rate: 214332 / 224269 (95.6%)
    Median Merged Length: 472

   224269  Pairs (224.3k)
   214332  Merged (214.3k, 95.57%)
    96579  Alignments with zero diffs (43.06%)
      685  Too many diffs (> 10) (0.31%)
        0  Fwd too short (< 64) after tail trimming (0.00%)
       17  Rev too short (< 64) after tail trimming (0.01%)
     9235  No alignment found (4.12%)
        0  Alignment too short (< 20) (0.00%)
        0  Merged too short (< 100)
        0  Min Q too low (<0) (0.00%)
       39  Staggered pairs (0.02%) merged & trimmed
    31.22  Mean alignment length
   470.31  Mean merged length
     1.05  Mean fwd expected errors
     1.15  Mean rev expected errors
     1.21  Mean merged expected errors
-----
  ‣ HG319-I1 - Merging Summary
    Merging Rate: 299144 / 308212 (97.1%)
    Median Merged Length: 472

   308212  Pairs (308.2k)
   299144  Merged (299.1k, 97.06%)
   207989  Alignments with zero diffs (67.48%)
      546  Too many diffs (> 10) (0.18%)
        1  Fwd too short (< 64) after tail trimming (0.00%)
       50  Rev too short (< 64) after tail trimming (0.02%)
     8456  No alignment found (2.74%)
       14  Alignment too short (< 20) (0.00%)
        1  Merged too short (< 100)
        0  Min Q too low (<0) (0.00%)
      144  Staggered pairs (0.05%) merged & trimmed
    31.93  Mean alignment length
   469.07  Mean merged length
     0.52  Mean fwd expected errors
     0.78  Mean rev expected errors
     0.84  Mean merged expected errors
-----
```

```
  ‣ HG319-I2 - Merging Summary
    Merging Rate: 229255 / 246196 (93.1%)
    Median Merged Length: 470

    246196  Pairs (246.2k)
    229255  Merged (229.3k, 93.12%)
    132536  Alignments with zero diffs (53.83%)
      4639  Too many diffs (> 10) (1.88%)
         4  Fwd too short (< 64) after tail trimming (0.00%)
        67  Rev too short (< 64) after tail trimming (0.03%)
     12217  No alignment found (4.96%)
        13  Alignment too short (< 20) (0.01%)
         1  Merged too short (< 100)
         0  Min Q too low (<0) (0.00%)
       422  Staggered pairs (0.17%) merged & trimmed
     49.25  Mean alignment length
    449.41  Mean merged length
      0.62  Mean fwd expected errors
      0.93  Mean rev expected errors
      0.78  Mean merged expected errors
-----
  ‣ HG319-I3 - Merging Summary
    Merging Rate: 249122 / 261574 (95.2%)
    Median Merged Length: 472

    261574  Pairs (261.6k)
    249122  Merged (249.1k, 95.24%)
    109565  Alignments with zero diffs (41.89%)
       514  Too many diffs (> 10) (0.20%)
         1  Fwd too short (< 64) after tail trimming (0.00%)
         4  Rev too short (< 64) after tail trimming (0.00%)
     11930  No alignment found (4.56%)
         3  Alignment too short (< 20) (0.00%)
         0  Merged too short (< 100)
         0  Min Q too low (<0) (0.00%)
        54  Staggered pairs (0.02%) merged & trimmed
     30.80  Mean alignment length
    470.70  Mean merged length
      1.07  Mean fwd expected errors
      1.16  Mean rev expected errors
      1.24  Mean merged expected errors
-----
  ‣ HG319-J1 - Merging Summary
    Merging Rate: 230353 / 246876 (93.3%)
    Median Merged Length: 470

    246876  Pairs (246.9k)
    230353  Merged (230.4k, 93.31%)
    153444  Alignments with zero diffs (62.15%)
      2397  Too many diffs (> 10) (0.97%)
         0  Fwd too short (< 64) after tail trimming (0.00%)
        86  Rev too short (< 64) after tail trimming (0.03%)
     14037  No alignment found (5.69%)
         3  Alignment too short (< 20) (0.00%)
```

```
         0  Merged too short (< 100)
         0  Min Q too low (<0) (0.00%)
       434  Staggered pairs (0.18%) merged & trimmed
     44.24  Mean alignment length
    453.40  Mean merged length
      0.69  Mean fwd expected errors
      0.70  Mean rev expected errors
      0.81  Mean merged expected errors
-----
  ‣ HG319-J2 - Merging Summary
    Merging Rate: 383627 / 398225 (96.3%)
    Median Merged Length: 472

    398225  Pairs (398.2k)
    383627  Merged (383.6k, 96.33%)
    264870  Alignments with zero diffs (66.51%)
      1751  Too many diffs (> 10) (0.44%)
         7  Fwd too short (< 64) after tail trimming (0.00%)
       215  Rev too short (< 64) after tail trimming (0.05%)
     12622  No alignment found (3.17%)
         3  Alignment too short (< 20) (0.00%)
         0  Merged too short (< 100)
         0  Min Q too low (<0) (0.00%)
       495  Staggered pairs (0.12%) merged & trimmed
     34.48  Mean alignment length
    464.73  Mean merged length
      0.53  Mean fwd expected errors
      0.81  Mean rev expected errors
      0.84  Mean merged expected errors
-----
  ‣ HG319-J3 - Merging Summary
    Merging Rate: 149612 / 163430 (91.5%)
    Median Merged Length: 472

    163430  Pairs (163.4k)
    149612  Merged (149.6k, 91.55%)
     65859  Alignments with zero diffs (40.30%)
      2230  Too many diffs (> 10) (1.36%)
         5  Fwd too short (< 64) after tail trimming (0.00%)
        67  Rev too short (< 64) after tail trimming (0.04%)
     11507  No alignment found (7.04%)
         9  Alignment too short (< 20) (0.01%)
         0  Merged too short (< 100)
         0  Min Q too low (<0) (0.00%)
       504  Staggered pairs (0.31%) merged & trimmed
     40.54  Mean alignment length
    457.35  Mean merged length
      1.11  Mean fwd expected errors
      1.17  Mean rev expected errors
      1.19  Mean merged expected errors
-----
  ‣ HG319-L1 - Merging Summary
    Merging Rate: 346019 / 356603 (97.0%)
    Median Merged Length: 472
```

```
    356603  Pairs (356.6k)
    346019  Merged (346.0k, 97.03%)
    237292  Alignments with zero diffs (66.54%)
       753  Too many diffs (> 10) (0.21%)
         0  Fwd too short (< 64) after tail trimming (0.00%)
        41  Rev too short (< 64) after tail trimming (0.01%)
      9784  No alignment found (2.74%)
         6  Alignment too short (< 20) (0.00%)
         0  Merged too short (< 100)
         0  Min Q too low (<0) (0.00%)
       129  Staggered pairs (0.04%) merged & trimmed
     31.84  Mean alignment length
    469.34  Mean merged length
      0.59  Mean fwd expected errors
      0.77  Mean rev expected errors
      0.87  Mean merged expected errors
-----
  ‣ HG319-L2 - Merging Summary
    Merging Rate: 457671 / 472320 (96.9%)
    Median Merged Length: 472

    472320  Pairs (472.3k)
    457671  Merged (457.7k, 96.90%)
    312656  Alignments with zero diffs (66.20%)
      1395  Too many diffs (> 10) (0.30%)
         1  Fwd too short (< 64) after tail trimming (0.00%)
        70  Rev too short (< 64) after tail trimming (0.01%)
     13179  No alignment found (2.79%)
         4  Alignment too short (< 20) (0.00%)
         0  Merged too short (< 100)
         0  Min Q too low (<0) (0.00%)
       354  Staggered pairs (0.07%) merged & trimmed
     32.75  Mean alignment length
    467.71  Mean merged length
      0.58  Mean fwd expected errors
      0.79  Mean rev expected errors
      0.87  Mean merged expected errors
-----
  ‣ HG319-L3 - Merging Summary
    Merging Rate: 201400 / 211510 (95.2%)
    Median Merged Length: 472

    211510  Pairs (211.5k)
    201400  Merged (201.4k, 95.22%)
     92223  Alignments with zero diffs (43.60%)
       876  Too many diffs (> 10) (0.41%)
         1  Fwd too short (< 64) after tail trimming (0.00%)
        26  Rev too short (< 64) after tail trimming (0.01%)
      9203  No alignment found (4.35%)
         4  Alignment too short (< 20) (0.00%)
         0  Merged too short (< 100)
         0  Min Q too low (<0) (0.00%)
       134  Staggered pairs (0.06%) merged & trimmed
```

```
    32.94  Mean alignment length
   467.60  Mean merged length
     1.06  Mean fwd expected errors
     1.10  Mean rev expected errors
     1.19  Mean merged expected errors
-----
  ‣ HG319-01 - Merging Summary
   Merging Rate: 273644 / 284243 (96.3%)
   Median Merged Length: 472

   284243  Pairs (284.2k)
   273644  Merged (273.6k, 96.27%)
   192600  Alignments with zero diffs (67.76%)
      853  Too many diffs (> 10) (0.30%)
       64  Fwd too short (< 64) after tail trimming (0.02%)
      754  Rev too short (< 64) after tail trimming (0.27%)
     8921  No alignment found (3.14%)
        6  Alignment too short (< 20) (0.00%)
        1  Merged too short (< 100)
        0  Min Q too low (<0) (0.00%)
      468  Staggered pairs (0.16%) merged & trimmed
    32.52  Mean alignment length
   465.39  Mean merged length
     0.58  Mean fwd expected errors
     0.77  Mean rev expected errors
     0.83  Mean merged expected errors
-----
  ‣ HG319-02 - Merging Summary
   Merging Rate: 154482 / 170966 (90.4%)
   Median Merged Length: 472

   170966  Pairs (171.0k)
   154482  Merged (154.5k, 90.36%)
    72602  Alignments with zero diffs (42.47%)
     2868  Too many diffs (> 10) (1.68%)
      130  Fwd too short (< 64) after tail trimming (0.08%)
      934  Rev too short (< 64) after tail trimming (0.55%)
    12532  No alignment found (7.33%)
        9  Alignment too short (< 20) (0.01%)
       11  Merged too short (< 100)
        0  Min Q too low (<0) (0.00%)
     1061  Staggered pairs (0.62%) merged & trimmed
    44.70  Mean alignment length
   446.69  Mean merged length
     0.98  Mean fwd expected errors
     1.06  Mean rev expected errors
     1.02  Mean merged expected errors
-----
  ‣ HG319-03 - Merging Summary
   Merging Rate: 137121 / 145483 (94.3%)
   Median Merged Length: 472

   145483  Pairs (145.5k)
   137121  Merged (137.1k, 94.25%)
```

```
     62587  Alignments with zero diffs (43.02%)
      1303  Too many diffs (> 10) (0.90%)
         0  Fwd too short (< 64) after tail trimming (0.00%)
        20  Rev too short (< 64) after tail trimming (0.01%)
      7034  No alignment found (4.83%)
         5  Alignment too short (< 20) (0.00%)
         0  Merged too short (< 100)
         0  Min Q too low (<0) (0.00%)
       156  Staggered pairs (0.11%) merged & trimmed
     36.17  Mean alignment length
    464.07  Mean merged length
      1.05  Mean fwd expected errors
      1.12  Mean rev expected errors
      1.13  Mean merged expected errors
-----
  ‣ HG319-Q2 - Merging Summary
    Merging Rate: 159473 / 169219 (94.2%)
    Median Merged Length: 472

    169219  Pairs (169.2k)
    159473  Merged (159.5k, 94.24%)
     70122  Alignments with zero diffs (41.44%)
      1399  Too many diffs (> 10) (0.83%)
         3  Fwd too short (< 64) after tail trimming (0.00%)
        35  Rev too short (< 64) after tail trimming (0.02%)
      8302  No alignment found (4.91%)
         6  Alignment too short (< 20) (0.00%)
         1  Merged too short (< 100)
         0  Min Q too low (<0) (0.00%)
       219  Staggered pairs (0.13%) merged & trimmed
     34.73  Mean alignment length
    465.63  Mean merged length
      1.11  Mean fwd expected errors
      1.14  Mean rev expected errors
      1.22  Mean merged expected errors
-----
  ‣ HG319-Q3 - Merging Summary
    Merging Rate: 81343 / 90475 (89.9%)
    Median Merged Length: 472

     90475  Pairs (90.5k)
     81343  Merged (81.3k, 89.91%)
     34610  Alignments with zero diffs (38.25%)
      1493  Too many diffs (> 10) (1.65%)
        50  Fwd too short (< 64) after tail trimming (0.06%)
       527  Rev too short (< 64) after tail trimming (0.58%)
      7040  No alignment found (7.78%)
        14  Alignment too short (< 20) (0.02%)
         8  Merged too short (< 100)
         0  Min Q too low (<0) (0.00%)
       653  Staggered pairs (0.72%) merged & trimmed
     43.61  Mean alignment length
    448.59  Mean merged length
      0.97  Mean fwd expected errors
```

```
      1.27  Mean rev expected errors
      1.13  Mean merged expected errors
-----
  ‣ HG319-R1 - Merging Summary
    Merging Rate: 230426 / 257156 (89.6%)
    Median Merged Length: 472

    257156  Pairs (257.2k)
    230426  Merged (230.4k, 89.61%)
    142215  Alignments with zero diffs (55.30%)
      7669  Too many diffs (> 10) (2.98%)
        13  Fwd too short (< 64) after tail trimming (0.01%)
       171  Rev too short (< 64) after tail trimming (0.07%)
     18860  No alignment found (7.33%)
        17  Alignment too short (< 20) (0.01%)
         0  Merged too short (< 100)
         0  Min Q too low (<0) (0.00%)
       773  Staggered pairs (0.30%) merged & trimmed
     57.42  Mean alignment length
    438.15  Mean merged length
      0.61  Mean fwd expected errors
      0.86  Mean rev expected errors
      0.73  Mean merged expected errors
-----
  ‣ HG319-R2 - Merging Summary
    Merging Rate: 193521 / 203672 (95.0%)
    Median Merged Length: 472

    203672  Pairs (203.7k)
    193521  Merged (193.5k, 95.02%)
     82242  Alignments with zero diffs (40.38%)
      1518  Too many diffs (> 10) (0.75%)
         0  Fwd too short (< 64) after tail trimming (0.00%)
         1  Rev too short (< 64) after tail trimming (0.00%)
      8627  No alignment found (4.24%)
         5  Alignment too short (< 20) (0.00%)
         0  Merged too short (< 100)
         0  Min Q too low (<0) (0.00%)
        66  Staggered pairs (0.03%) merged & trimmed
     32.87  Mean alignment length
    468.56  Mean merged length
      1.16  Mean fwd expected errors
      1.18  Mean rev expected errors
      1.26  Mean merged expected errors
-----
  ‣ HG319-R3 - Merging Summary
    Merging Rate: 48392 / 62365 (77.6%)
    Median Merged Length: 468

     62365  Pairs (62.4k)
     48392  Merged (48.4k, 77.59%)
     18433  Alignments with zero diffs (29.56%)
      3892  Too many diffs (> 10) (6.24%)
       158  Fwd too short (< 64) after tail trimming (0.25%)
```

```
       810  Rev too short (< 64) after tail trimming (1.30%)
      9086  No alignment found (14.57%)
        22  Alignment too short (< 20) (0.04%)
         5  Merged too short (< 100)
         0  Min Q too low (<0) (0.00%)
      1197  Staggered pairs (1.92%) merged & trimmed
     78.95  Mean alignment length
    398.01  Mean merged length
      0.89  Mean fwd expected errors
      1.49  Mean rev expected errors
      0.87  Mean merged expected errors
-----
  ‣ HGnegA - Merging Summary
    Merging Rate: 9702 / 11010 (88.1%)
    Median Merged Length: 464

     11010  Pairs (11.0k)
      9702  Merged (9702, 88.12%)
      3714  Alignments with zero diffs (33.73%)
       232  Too many diffs (> 10) (2.11%)
         2  Fwd too short (< 64) after tail trimming (0.02%)
         4  Rev too short (< 64) after tail trimming (0.04%)
      1059  No alignment found (9.62%)
         6  Alignment too short (< 20) (0.05%)
         5  Merged too short (< 100)
         0  Min Q too low (<0) (0.00%)
        23  Staggered pairs (0.21%) merged & trimmed
     40.47  Mean alignment length
    461.04  Mean merged length
      1.07  Mean fwd expected errors
      1.57  Mean rev expected errors
      1.38  Mean merged expected errors
-----
 End_B6_Trim_and_Merge: 17:05:26 07/01/2021
....................................................................
............
END_Workflow_StepB: 17:05:26 07/01/2021
====================================================================
============
Step C | Trim Primer Sites
====================================================================
============
          Application: usearch v11.0.667_i86linux64
              Command: search_pcr
  Amplicon Size Range: 100-600
       Forward Primer: U341F 5'-CCTACGGG[D]GGC[W]GCA-3'
       Reverse Primer: U806R 5'-GGACTAC[H][V]GGGT[M]TCTAATC-3'
Number of Mis-Matches: 2
             Coverage: full-length (no end gaps)
    Wildcards enabled: IUPAC codes
 Additional Filtering:
    -Exclude PCR-hits with mismatches at the primer end.
    -Exclude amplicons in the wrong (reverse) orientation.
    -Remove sequences with more than 1 amplicon.
```

```
--------------------------------------------------------------------
------------
START_Workflow_StepC: 17:05:57 07/01/2021
....................................................................
............
 HG015-B1: N(allPCRHits)= 226980 (-13997)
 HG015-B1: N(SingleHits)= 226980 (-13997)
 HG015-B1: N(CleanHits) = 226093 (-14884)
 ◇ Amplicon Orientation:
    226980 HG015-B1-PF HG015-B1-PR
 ◇ Mis-Matches (top3):
   PrimerF   2078 C...............
   PrimerF   3334 ...A............
   PrimerF 214244 ................
   PrimerR   1252 ..A..................
   PrimerR   1632 .................A...
   PrimerR 216485 .....................
 - - -
 HG015-B3: N(allPCRHits)= 74278 (-65083)
 HG015-B3: N(SingleHits)= 74278 (-65083)
 HG015-B3: N(CleanHits) = 67058 (-72303)
 ◇ Amplicon Orientation:
     74278 HG015-B3-PF HG015-B3-PR
 ◇ Mis-Matches (top3):
   PrimerF    649 ............W...
   PrimerF   6686 ...............A
   PrimerF  63346 ................
   PrimerR    665 ..................A.C
   PrimerR   5570 .................AA..
   PrimerR  64297 .....................
 - - -
 HG015-D1: N(allPCRHits)= 184454 (-20496)
 HG015-D1: N(SingleHits)= 184454 (-20496)
 HG015-D1: N(CleanHits) = 182219 (-22731)
 ◇ Amplicon Orientation:
    184453 HG015-D1-PF HG015-D1-PR
         1 HG015-D1-PR HG015-D1-PF
 ◇ Mis-Matches (top3):
   PrimerF   1759 ...............A
   PrimerF   2758 ...A............
   PrimerF 172218 ................
   PrimerR   1152 ..A..................
   PrimerR   1350 .................AA..
   PrimerR 174461 .....................
 - - -
 HG015-D2: N(allPCRHits)= 278853 (-15924)
 HG015-D2: N(SingleHits)= 278853 (-15924)
 HG015-D2: N(CleanHits) = 276064 (-18713)
 ◇ Amplicon Orientation:
    278853 HG015-D2-PF HG015-D2-PR
 ◇ Mis-Matches (top3):
   PrimerF   2630 C...............
   PrimerF   3982 ...A............
   PrimerF 263159 ................
```

```
  PrimerR   1715 .................A...
  PrimerR   1725 ..A..................
  PrimerR 264402 .....................
– – –
HG015–D3: N(allPCRHits)= 128254 (–22645)
HG015–D3: N(SingleHits)= 128254 (–22645)
HG015–D3: N(CleanHits) = 126108 (–24791)
◇ Amplicon Orientation:
   128254 HG015–D3–PF HG015–D3–PR
◇ Mis–Matches (top3):
  PrimerF   1355 ............W...
  PrimerF   1908 ...............A
  PrimerF 119529 ................
  PrimerR    751 ..................A..
  PrimerR   1558 .................AA..
  PrimerR 120941 .....................
– – –
HG015–I1: N(allPCRHits)= 64794 (–35054)
HG015–I1: N(SingleHits)= 64794 (–35054)
HG015–I1: N(CleanHits) = 52777 (–47071)
◇ Amplicon Orientation:
    64794 HG015–I1–PF HG015–I1–PR
◇ Mis–Matches (top3):
  PrimerF    719 ...A............
  PrimerF  11249 ...............A
  PrimerF  50223 ................
  PrimerR    410 .................A...
  PrimerR  10919 .................AA..
  PrimerR  50608 .....................
– – –
HG015–I2: N(allPCRHits)= 172256 (–10685)
HG015–I2: N(SingleHits)= 172256 (–10685)
HG015–I2: N(CleanHits) = 171242 (–11699)
◇ Amplicon Orientation:
   172256 HG015–I2–PF HG015–I2–PR
◇ Mis–Matches (top3):
  PrimerF   1640 C...............
  PrimerF   2517 ...A............
  PrimerF 162526 ................
  PrimerR   1074 ..A..................
  PrimerR   1316 .................A...
  PrimerR 163413 .....................
– – –
HG015–I3: N(allPCRHits)= 138334 (–7048)
HG015–I3: N(SingleHits)= 138334 (–7048)
HG015–I3: N(CleanHits) = 137833 (–7549)
◇ Amplicon Orientation:
   138334 HG015–I3–PF HG015–I3–PR
◇ Mis–Matches (top3):
  PrimerF   1142 ............W...
  PrimerF   1236 C...............
  PrimerF 131244 ................
  PrimerR    818 ..................A..
  PrimerR    949 ..A..................
```

```
  PrimerR 131812 ....................
- - -
HG015-J1: N(allPCRHits)= 89154 (-13659)
HG015-J1: N(SingleHits)= 89154 (-13659)
HG015-J1: N(CleanHits) = 84847 (-17966)
◇ Amplicon Orientation:
    89154 HG015-J1-PF HG015-J1-PR
◇ Mis-Matches (top3):
  PrimerF   1166 ...A...........
  PrimerF   3994 ...............A
  PrimerF  80493 ................
  PrimerR    681 .................A...
  PrimerR   4010 .................AA..
  PrimerR  80826 .....................
- - -
HG015-J2: N(allPCRHits)= 232577 (-23260)
HG015-J2: N(SingleHits)= 232577 (-23260)
HG015-J2: N(CleanHits) = 230087 (-25750)
◇ Amplicon Orientation:
   232577 HG015-J2-PF HG015-J2-PR
◇ Mis-Matches (top3):
  PrimerF   2297 C...............
  PrimerF   3281 ...A............
  PrimerF 217887 ................
  PrimerR   1267 .................A...
  PrimerR   1722 .................AA..
  PrimerR 220897 .....................
- - -
HG015-J3: N(allPCRHits)= 236745 (-13047)
HG015-J3: N(SingleHits)= 236745 (-13047)
HG015-J3: N(CleanHits) = 235299 (-14493)
◇ Amplicon Orientation:
   236745 HG015-J3-PF HG015-J3-PR
◇ Mis-Matches (top3):
  PrimerF   1221 ...............A
  PrimerF   2141 C...............
  PrimerF 225007 ................
  PrimerR   1096 ..................A..
  PrimerR   1385 .................A...
  PrimerR 226122 .....................
- - -
HG015-L1: N(allPCRHits)= 381911 (-27481)
HG015-L1: N(SingleHits)= 381911 (-27481)
HG015-L1: N(CleanHits) = 379957 (-29435)
◇ Amplicon Orientation:
   381911 HG015-L1-PF HG015-L1-PR
◇ Mis-Matches (top3):
  PrimerF   3672 C...............
  PrimerF   5660 ...A............
  PrimerF 359990 ................
  PrimerR   2782 .................A...
  PrimerR   2862 ..A..................
  PrimerR 362097 .....................
- - -
```

```
HG015-L2: N(allPCRHits)= 236551 (-29970)
HG015-L2: N(SingleHits)= 236551 (-29970)
HG015-L2: N(CleanHits) = 233337 (-33184)
◇ Amplicon Orientation:
   236551 HG015-L2-PF HG015-L2-PR
◇ Mis-Matches (top3):
  PrimerF   2802 ...............A
  PrimerF   3334 ...A............
  PrimerF 220970 ................
  PrimerR   1640 .................A...
  PrimerR   2358 .................AA..
  PrimerR 222741 .....................
- - -
HG015-L3: N(allPCRHits)= 161392 (-10996)
HG015-L3: N(SingleHits)= 161392 (-10996)
HG015-L3: N(CleanHits) = 159884 (-12504)
◇ Amplicon Orientation:
   161392 HG015-L3-PF HG015-L3-PR
◇ Mis-Matches (top3):
  PrimerF   1277 ...............A
  PrimerF   1431 C...............
  PrimerF 152710 ................
  PrimerR    720 ..................A..
  PrimerR   1066 .................AA..
  PrimerR 154192 .....................
- - -
HG015-O1: N(allPCRHits)= 207642 (-19045)
HG015-O1: N(SingleHits)= 207642 (-19045)
HG015-O1: N(CleanHits) = 204377 (-22310)
◇ Amplicon Orientation:
   207642 HG015-O1-PF HG015-O1-PR
◇ Mis-Matches (top3):
  PrimerF   2877 ...............A
  PrimerF   3029 ...A............
  PrimerF 193762 ................
  PrimerR   2005 .................A...
  PrimerR   2510 .................AA..
  PrimerR 194016 .....................
- - -
HG015-O2: N(allPCRHits)= 94331 (-43135)
HG015-O2: N(SingleHits)= 94331 (-43135)
HG015-O2: N(CleanHits) = 82001 (-55465)
◇ Amplicon Orientation:
    94331 HG015-O2-PF HG015-O2-PR
◇ Mis-Matches (top3):
  PrimerF   1170 ...A............
  PrimerF  11348 ...............A
  PrimerF  77733 ................
  PrimerR    548 .................A...
  PrimerR  11236 .................AA..
  PrimerR  78461 .....................
- - -
HG015-O3: N(allPCRHits)= 197010 (-16432)
HG015-O3: N(SingleHits)= 197010 (-16432)
```

```
HG015-O3: N(CleanHits) = 195419 (-18023)
◇ Amplicon Orientation:
   197009 HG015-O3-PF HG015-O3-PR
        1 HG015-O3-PR HG015-O3-PF
◇ Mis-Matches (top3):
  PrimerF   1771 C...............
  PrimerF   2007 ............W...
  PrimerF 184833 ................
  PrimerR   1254 ..................A..
  PrimerR   1629 ..A..................
  PrimerR 186484 .....................
- - -
HG015-Q1: N(allPCRHits)= 229914 (-12145)
HG015-Q1: N(SingleHits)= 229914 (-12145)
HG015-Q1: N(CleanHits) = 229222 (-12837)
◇ Amplicon Orientation:
   229914 HG015-Q1-PF HG015-Q1-PR
◇ Mis-Matches (top3):
  PrimerF   2226 C...............
  PrimerF   3078 ...A............
  PrimerF 218228 ................
  PrimerR   1387 ..................A..
  PrimerR   1979 .................A...
  PrimerR 218344 .....................
- - -
HG015-Q2: N(allPCRHits)= 137822 (-9209)
HG015-Q2: N(SingleHits)= 137822 (-9209)
HG015-Q2: N(CleanHits) = 136740 (-10291)
◇ Amplicon Orientation:
   137822 HG015-Q2-PF HG015-Q2-PR
◇ Mis-Matches (top3):
  PrimerF   1046 ............W...
  PrimerF   1301 C...............
  PrimerF 130775 ................
  PrimerR    614 ..................A..
  PrimerR    754 .................AA..
  PrimerR 131863 .....................
- - -
HG015-Q3: N(allPCRHits)= 287741 (-13482)
HG015-Q3: N(SingleHits)= 287741 (-13482)
HG015-Q3: N(CleanHits) = 286749 (-14474)
◇ Amplicon Orientation:
   287741 HG015-Q3-PF HG015-Q3-PR
◇ Mis-Matches (top3):
  PrimerF   1409 ............W...
  PrimerF   2814 C...............
  PrimerF 274657 ................
  PrimerR   1187 ..A..................
  PrimerR   1248 ..................A..
  PrimerR 276450 .....................
- - -
HG015-R1: N(allPCRHits)= 355546 (-20562)
HG015-R1: N(SingleHits)= 355546 (-20562)
HG015-R1: N(CleanHits) = 354030 (-22078)
```

```
◇ Amplicon Orientation:
   355546 HG015-R1-PF HG015-R1-PR
◇ Mis-Matches (top3):
  PrimerF   3403 C...............
  PrimerF   5292 ...A............
  PrimerF 335943 ................
  PrimerR   2449 ..A..................
  PrimerR   2788 .................A...
  PrimerR 337601 .....................
- - -
HG015-R2: N(allPCRHits)= 14939 (-11830)
HG015-R2: N(SingleHits)= 14939 (-11830)
HG015-R2: N(CleanHits) = 12433 (-14336)
◇ Amplicon Orientation:
    14939 HG015-R2-PF HG015-R2-PR
◇ Mis-Matches (top3):
  PrimerF    152 C...............
  PrimerF   2344 ...............A
  PrimerF  11817 ................
  PrimerR    167 ..................A..
  PrimerR   2182 .................AA..
  PrimerR  11665 .....................
- - -
HG015-R3: N(allPCRHits)= 168056 (-10138)
HG015-R3: N(SingleHits)= 168056 (-10138)
HG015-R3: N(CleanHits) = 166412 (-11782)
◇ Amplicon Orientation:
   168056 HG015-R3-PF HG015-R3-PR
◇ Mis-Matches (top3):
  PrimerF   1392 ...............A
  PrimerF   1636 C...............
  PrimerF 158368 ................
  PrimerR   1050 ..................A..
  PrimerR   1105 .................AA..
  PrimerR 159174 .....................
- - -
HG033-B1: N(allPCRHits)= 259162 (-20392)
HG033-B1: N(SingleHits)= 259162 (-20392)
HG033-B1: N(CleanHits) = 258163 (-21391)
◇ Amplicon Orientation:
   259161 HG033-B1-PF HG033-B1-PR
        1 HG033-B1-PR HG033-B1-PF
◇ Mis-Matches (top3):
  PrimerF   2404 C...............
  PrimerF   3781 ...A............
  PrimerF 244337 ................
  PrimerR   1684 .................A...
  PrimerR   1970 ..................A..
  PrimerR 246642 .....................
- - -
HG033-B2: N(allPCRHits)= 309174 (-21573)
HG033-B2: N(SingleHits)= 309174 (-21573)
HG033-B2: N(CleanHits) = 307782 (-22965)
◇ Amplicon Orientation:
```

```
   309174 HG033-B2-PF HG033-B2-PR
◇ Mis-Matches (top3):
  PrimerF   2963 C...............
  PrimerF   4706 ...A............
  PrimerF 291437 ................
  PrimerR   2035 .................A...
  PrimerR   2092 ..A..................
  PrimerR 294445 .....................
- - -
HG033-B3: N(allPCRHits)= 139924 (-9729)
HG033-B3: N(SingleHits)= 139924 (-9729)
HG033-B3: N(CleanHits) = 139486 (-10167)
◇ Amplicon Orientation:
   139923 HG033-B3-PF HG033-B3-PR
        1 HG033-B3-PR HG033-B3-PF
◇ Mis-Matches (top3):
  PrimerF   1174 ............W...
  PrimerF   1252 C...............
  PrimerF 132189 ................
  PrimerR    604 ..................A..
  PrimerR    619 ..A..................
  PrimerR 134263 .....................
- - -
HG033-D1: N(allPCRHits)= 193547 (-12300)
HG033-D1: N(SingleHits)= 193547 (-12300)
HG033-D1: N(CleanHits) = 192423 (-13424)
◇ Amplicon Orientation:
   193547 HG033-D1-PF HG033-D1-PR
◇ Mis-Matches (top3):
  PrimerF   1813 C...............
  PrimerF   2715 ...A............
  PrimerF 182498 ................
  PrimerR    980 ..................A..
  PrimerR   1400 .................A...
  PrimerR 184002 .....................
- - -
HG033-D2: N(allPCRHits)= 279731 (-17663)
HG033-D2: N(SingleHits)= 279731 (-17663)
HG033-D2: N(CleanHits) = 277802 (-19592)
◇ Amplicon Orientation:
   279731 HG033-D2-PF HG033-D2-PR
◇ Mis-Matches (top3):
  PrimerF   2617 C...............
  PrimerF   4339 ...A............
  PrimerF 263928 ................
  PrimerR   1581 .................A...
  PrimerR   1741 ..A..................
  PrimerR 266681 .....................
- - -
HG033-D3: N(allPCRHits)= 198192 (-37579)
HG033-D3: N(SingleHits)= 198192 (-37579)
HG033-D3: N(CleanHits) = 191490 (-44281)
◇ Amplicon Orientation:
   198192 HG033-D3-PF HG033-D3-PR
```

```
◇ Mis-Matches (top3):
  PrimerF   1933 C...............
  PrimerF   6237 ...............A
  PrimerF 182468 ................
  PrimerR   1020 ..................A..
  PrimerR   5285 .................AA..
  PrimerR 183923 .....................
- - -
HG033-I1: N(allPCRHits)= 147390 (-53984)
HG033-I1: N(SingleHits)= 147390 (-53984)
HG033-I1: N(CleanHits) = 116626 (-84748)
◇ Amplicon Orientation:
   147390 HG033-I1-PF HG033-I1-PR
◇ Mis-Matches (top3):
  PrimerF   1623 ...A............
  PrimerF  28492 ...............A
  PrimerF 110766 ................
  PrimerR   1434 ..................A..
  PrimerR  26885 .................AA..
  PrimerR 111533 .....................
- - -
HG033-I2: N(allPCRHits)= 216396 (-14745)
HG033-I2: N(SingleHits)= 216396 (-14745)
HG033-I2: N(CleanHits) = 214549 (-16592)
◇ Amplicon Orientation:
   216396 HG033-I2-PF HG033-I2-PR
◇ Mis-Matches (top3):
  PrimerF   2365 C...............
  PrimerF   3153 ...A............
  PrimerF 202899 ................
  PrimerR   1499 ..A..................
  PrimerR   1915 .................A...
  PrimerR 204342 .....................
- - -
HG033-I3: N(allPCRHits)= 571939 (-94005)
HG033-I3: N(SingleHits)= 571939 (-94005)
HG033-I3: N(CleanHits) = 560461 (-105483)
◇ Amplicon Orientation:
   571939 HG033-I3-PF HG033-I3-PR
◇ Mis-Matches (top3):
  PrimerF   5590 C...............
  PrimerF  10199 ...............A
  PrimerF 532652 ................
  PrimerR   2871 ..................A..
  PrimerR   8471 .................AA..
  PrimerR 537035 .....................
- - -
HG033-J1: N(allPCRHits)= 346805 (-18233)
HG033-J1: N(SingleHits)= 346803 (-18235)
HG033-J1: N(CleanHits) = 345815 (-19223)
◇ Amplicon Orientation:
   346805 HG033-J1-PF HG033-J1-PR
◇ Mis-Matches (top3):
  PrimerF   3291 C...............
```

```
  PrimerF   4970 ...A...........
  PrimerF 328093 ...............
  PrimerR   2327 ..A.................
  PrimerR   2452 .................A...
  PrimerR 329929 .....................
- - -
HG033-J2: N(allPCRHits)= 170748 (-23910)
HG033-J2: N(SingleHits)= 170748 (-23910)
HG033-J2: N(CleanHits) = 165390 (-29268)
◇ Amplicon Orientation:
   170748 HG033-J2-PF HG033-J2-PR
◇ Mis-Matches (top3):
  PrimerF   2350 ...A...........
  PrimerF   4768 ..............A
  PrimerF 156854 ...............
  PrimerR   1328 .................A...
  PrimerR   4225 .................AA..
  PrimerR 157891 .....................
- - -
HG033-J3: N(allPCRHits)= 172097 (-15190)
HG033-J3: N(SingleHits)= 172097 (-15190)
HG033-J3: N(CleanHits) = 170859 (-16428)
◇ Amplicon Orientation:
   172097 HG033-J3-PF HG033-J3-PR
◇ Mis-Matches (top3):
  PrimerF   1502 ............W...
  PrimerF   1575 C..............
  PrimerF 162096 ...............
  PrimerR    743 .................A...
  PrimerR    933 ..................A..
  PrimerR 164020 .....................
- - -
HG033-L1: N(allPCRHits)= 242025 (-11929)
HG033-L1: N(SingleHits)= 242025 (-11929)
HG033-L1: N(CleanHits) = 241224 (-12730)
◇ Amplicon Orientation:
   242025 HG033-L1-PF HG033-L1-PR
◇ Mis-Matches (top3):
  PrimerF   2291 C..............
  PrimerF   3152 ...A...........
  PrimerF 229562 ...............
  PrimerR   1489 ..A.................
  PrimerR   2021 .................A...
  PrimerR 229985 .....................
- - -
HG033-L2: N(allPCRHits)= 76541 (-28548)
HG033-L2: N(SingleHits)= 76541 (-28548)
HG033-L2: N(CleanHits) = 67363 (-37726)
◇ Amplicon Orientation:
    76540 HG033-L2-PF HG033-L2-PR
        1 HG033-L2-PR HG033-L2-PF
◇ Mis-Matches (top3):
  PrimerF    959 ...A...........
  PrimerF   8582 ..............A
```

```
  PrimerF  64014 ................
  PrimerR    678 .................A.T.
  PrimerR   8182 .................AA..
  PrimerR  63749 ....................
- - -
HG033-L3: N(allPCRHits)= 1320547 (-103662)
HG033-L3: N(SingleHits)= 1320547 (-103662)
HG033-L3: N(CleanHits) = 1310796 (-113413)
◇ Amplicon Orientation:
  1320547 HG033-L3-PF HG033-L3-PR
◇ Mis-Matches (top3):
  PrimerF  12775 C...............
  PrimerF  12802 ............W...
  PrimerF1241464 ................
  PrimerR   6027 .................A...
  PrimerR   6493 ..................A..
  PrimerR1258253 ....................
- - -
HG033-O1: N(allPCRHits)= 358630 (-24932)
HG033-O1: N(SingleHits)= 358630 (-24932)
HG033-O1: N(CleanHits) = 354718 (-28844)
◇ Amplicon Orientation:
   358630 HG033-O1-PF HG033-O1-PR
◇ Mis-Matches (top3):
  PrimerF   3410 C...............
  PrimerF   5100 ...A............
  PrimerF 336476 ................
  PrimerR   2472 .................A...
  PrimerR   2911 .................AA..
  PrimerR 339025 ....................
- - -
HG033-O2: N(allPCRHits)= 278880 (-15994)
HG033-O2: N(SingleHits)= 278870 (-16004)
HG033-O2: N(CleanHits) = 277671 (-17203)
◇ Amplicon Orientation:
   278879 HG033-O2-PF HG033-O2-PR
        1 HG033-O2-PR HG033-O2-PF
◇ Mis-Matches (top3):
  PrimerF   2658 C...............
  PrimerF   3879 ...A............
  PrimerF 264141 ................
  PrimerR   1492 ..A.................
  PrimerR   1802 .................A...
  PrimerR 266388 ....................
- - -
HG033-O3: N(allPCRHits)= 145130 (-11230)
HG033-O3: N(SingleHits)= 145128 (-11232)
HG033-O3: N(CleanHits) = 143887 (-12473)
◇ Amplicon Orientation:
   145130 HG033-O3-PF HG033-O3-PR
◇ Mis-Matches (top3):
  PrimerF   1134 ............W...
  PrimerF   1245 C...............
  PrimerF 136936 ................
```

```
  PrimerR    868 ..A..................
  PrimerR    914 .................AA..
  PrimerR 137770 .....................
- - -
HG033-Q1: N(allPCRHits)= 172217 (-35106)
HG033-Q1: N(SingleHits)= 172217 (-35106)
HG033-Q1: N(CleanHits) = 166285 (-41038)
◇ Amplicon Orientation:
   172217 HG033-Q1-PF HG033-Q1-PR
◇ Mis-Matches (top3):
  PrimerF   2466 ...A............
  PrimerF   5097 ...............A
  PrimerF 157513 ................
  PrimerR   1162 ..A..................
  PrimerR   4825 .................AA..
  PrimerR 158893 .....................
- - -
HG033-Q2: N(allPCRHits)= 151238 (-15559)
HG033-Q2: N(SingleHits)= 151238 (-15559)
HG033-Q2: N(CleanHits) = 148840 (-17957)
◇ Amplicon Orientation:
   151237 HG033-Q2-PF HG033-Q2-PR
        1 HG033-Q2-PR HG033-Q2-PF
◇ Mis-Matches (top3):
  PrimerF   1372 C...............
  PrimerF   2116 ...............A
  PrimerF 141859 ................
  PrimerR    870 .................A...
  PrimerR   2025 .................AA..
  PrimerR 142650 .....................
- - -
HG033-Q3: N(allPCRHits)= 143462 (-11223)
HG033-Q3: N(SingleHits)= 143462 (-11223)
HG033-Q3: N(CleanHits) = 140865 (-13820)
◇ Amplicon Orientation:
   143462 HG033-Q3-PF HG033-Q3-PR
◇ Mis-Matches (top3):
  PrimerF   1382 C...............
  PrimerF   2347 ...............A
  PrimerF 134516 ................
  PrimerR    800 ..................A..
  PrimerR   2240 .................AA..
  PrimerR 135085 .....................
- - -
HG033-R1: N(allPCRHits)= 215832 (-54114)
HG033-R1: N(SingleHits)= 215832 (-54114)
HG033-R1: N(CleanHits) = 204756 (-65190)
◇ Amplicon Orientation:
   215832 HG033-R1-PF HG033-R1-PR
◇ Mis-Matches (top3):
  PrimerF   2970 ...A............
  PrimerF   9777 ...............A
  PrimerF 193854 ................
  PrimerR   1543 .................A...
```

```
  PrimerR   9054 .................AA..
  PrimerR 195185 .....................
- - -
HG033-R2: N(allPCRHits)= 119176 (-10612)
HG033-R2: N(SingleHits)= 119176 (-10612)
HG033-R2: N(CleanHits) = 118460 (-11328)
◇ Amplicon Orientation:
   119176 HG033-R2-PF HG033-R2-PR
◇ Mis-Matches (top3):
  PrimerF   1194 ............W...
  PrimerF   1372 C...............
  PrimerF 112083 ................
  PrimerR   6224 .................A...
  PrimerR   8166 ..................A..
  PrimerR  84552 .....................
- - -
HG033-R3: N(allPCRHits)= 99795 (-30962)
HG033-R3: N(SingleHits)= 99795 (-30962)
HG033-R3: N(CleanHits) = 87031 (-43726)
◇ Amplicon Orientation:
    99795 HG033-R3-PF HG033-R3-PR
◇ Mis-Matches (top3):
  PrimerF    882 C...............
  PrimerF  11905 ...............A
  PrimerF  82930 ................
  PrimerR    529 .................A.T.
  PrimerR  12047 .................AA..
  PrimerR  82769 .....................
- - -
HG059-B1: N(allPCRHits)= 279370 (-20188)
HG059-B1: N(SingleHits)= 279370 (-20188)
HG059-B1: N(CleanHits) = 278338 (-21220)
◇ Amplicon Orientation:
   279370 HG059-B1-PF HG059-B1-PR
◇ Mis-Matches (top3):
  PrimerF   2679 C...............
  PrimerF   4057 ...A............
  PrimerF 263089 ................
  PrimerR   1536 ..A..................
  PrimerR   2136 .................A...
  PrimerR 266172 .....................
- - -
HG059-B2: N(allPCRHits)= 269111 (-16313)
HG059-B2: N(SingleHits)= 269111 (-16313)
HG059-B2: N(CleanHits) = 268218 (-17206)
◇ Amplicon Orientation:
   269110 HG059-B2-PF HG059-B2-PR
        1 HG059-B2-PR HG059-B2-PF
◇ Mis-Matches (top3):
  PrimerF   2474 C...............
  PrimerF   4015 ...A............
  PrimerF 254166 ................
  PrimerR   1706 ..A..................
  PrimerR   1738 .................A...
```

```
  PrimerR 257092 ....................
– – –
HG059–B3: N(allPCRHits)= 158802 (–7987)
HG059–B3: N(SingleHits)= 158802 (–7987)
HG059–B3: N(CleanHits) = 158350 (–8439)
◇ Amplicon Orientation:
   158802 HG059–B3–PF HG059–B3–PR
◇ Mis–Matches (top3):
  PrimerF   1025 ............W...
  PrimerF   1519 C...............
  PrimerF 150835 ................
  PrimerR    855 ..................A..
  PrimerR   3293 .................A...
  PrimerR 149697 ....................
– – –
HG059–D1: N(allPCRHits)= 336998 (–28563)
HG059–D1: N(SingleHits)= 336998 (–28563)
HG059–D1: N(CleanHits) = 335466 (–30095)
◇ Amplicon Orientation:
   336998 HG059–D1–PF HG059–D1–PR
◇ Mis–Matches (top3):
  PrimerF   3124 C...............
  PrimerF   4934 ...A............
  PrimerF 317437 ................
  PrimerR   2047 ..A.................
  PrimerR   2262 .................A...
  PrimerR 321106 ....................
– – –
HG059–D2: N(allPCRHits)= 391503 (–22499)
HG059–D2: N(SingleHits)= 391503 (–22499)
HG059–D2: N(CleanHits) = 389449 (–24553)
◇ Amplicon Orientation:
   391502 HG059–D2–PF HG059–D2–PR
        1 HG059–D2–PR HG059–D2–PF
◇ Mis–Matches (top3):
  PrimerF   3672 C...............
  PrimerF   5366 ...A............
  PrimerF 369790 ................
  PrimerR   2238 ..A.................
  PrimerR   2477 .................A...
  PrimerR 373231 ....................
– – –
HG059–D3: N(allPCRHits)= 320334 (–20545)
HG059–D3: N(SingleHits)= 320334 (–20545)
HG059–D3: N(CleanHits) = 318814 (–22065)
◇ Amplicon Orientation:
   320334 HG059–D3–PF HG059–D3–PR
◇ Mis–Matches (top3):
  PrimerF   2808 ............W...
  PrimerF   2878 C...............
  PrimerF 302617 ................
  PrimerR   1715 ..A.................
  PrimerR   2058 ..................A..
  PrimerR 305060 ....................
```

```
- - -
HG059-I1: N(allPCRHits)= 543782 (-26798)
HG059-I1: N(SingleHits)= 543782 (-26798)
HG059-I1: N(CleanHits) = 542419 (-28161)
◇ Amplicon Orientation:
   543782 HG059-I1-PF HG059-I1-PR
◇ Mis-Matches (top3):
  PrimerF   5149 C...............
  PrimerF   7337 ...A............
  PrimerF 516141 ................
  PrimerR   2868 ..A..................
  PrimerR   4375 .................A...
  PrimerR 518715 .....................
- - -
HG059-I2: N(allPCRHits)= 159147 (-11953)
HG059-I2: N(SingleHits)= 159147 (-11953)
HG059-I2: N(CleanHits) = 158446 (-12654)
◇ Amplicon Orientation:
   159147 HG059-I2-PF HG059-I2-PR
◇ Mis-Matches (top3):
  PrimerF   1449 C...............
  PrimerF   2333 ...A............
  PrimerF 150034 ................
  PrimerR   1234 ..A..................
  PrimerR   1254 .................A...
  PrimerR 150895 .....................
- - -
HG059-I3: N(allPCRHits)= 190471 (-9689)
HG059-I3: N(SingleHits)= 190471 (-9689)
HG059-I3: N(CleanHits) = 189974 (-10186)
◇ Amplicon Orientation:
   190471 HG059-I3-PF HG059-I3-PR
◇ Mis-Matches (top3):
  PrimerF   1396 ............W...
  PrimerF   2012 C...............
  PrimerF 180481 ................
  PrimerR    828 .................A...
  PrimerR    929 ..................A..
  PrimerR 182701 .....................
- - -
HG059-J1: N(allPCRHits)= 249328 (-15504)
HG059-J1: N(SingleHits)= 249328 (-15504)
HG059-J1: N(CleanHits) = 246967 (-17865)
◇ Amplicon Orientation:
   249328 HG059-J1-PF HG059-J1-PR
◇ Mis-Matches (top3):
  PrimerF   2446 C...............
  PrimerF   3671 ...A............
  PrimerF 234535 ................
  PrimerR   1739 .................AA..
  PrimerR   1795 .................A...
  PrimerR 236015 .....................
- - -
HG059-J2: N(allPCRHits)= 241113 (-38180)
```

```
HG059-J2: N(SingleHits)= 241113 (-38180)
HG059-J2: N(CleanHits) = 235383 (-43910)
◇ Amplicon Orientation:
   241112 HG059-J2-PF HG059-J2-PR
        1 HG059-J2-PR HG059-J2-PF
◇ Mis-Matches (top3):
  PrimerF   3593 ...A...........
  PrimerF   5081 ...............A
  PrimerF 222929 ................
  PrimerR   1681 .................A...
  PrimerR   4405 .................AA..
  PrimerR 224831 .....................
- - -
HG059-J3: N(allPCRHits)= 923054 (-56527)
HG059-J3: N(SingleHits)= 923050 (-56531)
HG059-J3: N(CleanHits) = 918105 (-61476)
◇ Amplicon Orientation:
   923054 HG059-J3-PF HG059-J3-PR
◇ Mis-Matches (top3):
  PrimerF   7313 ............W...
  PrimerF   8334 C...............
  PrimerF 874065 ................
  PrimerR   4051 .................A...
  PrimerR   4250 ..................A..
  PrimerR 882129 .....................
- - -
HG059-L1: N(allPCRHits)= 161608 (-8258)
HG059-L1: N(SingleHits)= 161608 (-8258)
HG059-L1: N(CleanHits) = 161112 (-8754)
◇ Amplicon Orientation:
   161608 HG059-L1-PF HG059-L1-PR
◇ Mis-Matches (top3):
  PrimerF   1425 C...............
  PrimerF   2499 ...A............
  PrimerF 152999 ................
  PrimerR   1151 ..A..................
  PrimerR   1247 .................A...
  PrimerR 154034 .....................
- - -
HG059-L2: N(allPCRHits)= 264218 (-28652)
HG059-L2: N(SingleHits)= 264218 (-28652)
HG059-L2: N(CleanHits) = 261295 (-31575)
◇ Amplicon Orientation:
   264218 HG059-L2-PF HG059-L2-PR
◇ Mis-Matches (top3):
  PrimerF   2493 C...............
  PrimerF   3981 ...A............
  PrimerF 247920 ................
  PrimerR   1525 .................A...
  PrimerR   1584 ..A..................
  PrimerR 250825 .....................
- - -
HG059-L3: N(allPCRHits)= 212972 (-13432)
HG059-L3: N(SingleHits)= 212972 (-13432)
```

```
HG059-L3: N(CleanHits) = 212134 (-14270)
◇ Amplicon Orientation:
   212972 HG059-L3-PF HG059-L3-PR
◇ Mis-Matches (top3):
  PrimerF   1894 C...............
  PrimerF   2255 ............W...
  PrimerF 200742 ................
  PrimerR   1025 ..................A..
  PrimerR   1118 ..A..................
  PrimerR 203696 .....................
- - -
HG059-O1: N(allPCRHits)= 359046 (-21812)
HG059-O1: N(SingleHits)= 359046 (-21812)
HG059-O1: N(CleanHits) = 356604 (-24254)
◇ Amplicon Orientation:
   359046 HG059-O1-PF HG059-O1-PR
◇ Mis-Matches (top3):
  PrimerF   3409 C...............
  PrimerF   5507 ...A............
  PrimerF 337944 ................
  PrimerR   2012 ..A..................
  PrimerR   2655 .................A...
  PrimerR 340650 .....................
- - -
HG059-O2: N(allPCRHits)= 294216 (-13959)
HG059-O2: N(SingleHits)= 294216 (-13959)
HG059-O2: N(CleanHits) = 293051 (-15124)
◇ Amplicon Orientation:
   294216 HG059-O2-PF HG059-O2-PR
◇ Mis-Matches (top3):
  PrimerF   2982 C...............
  PrimerF   4195 ...A............
  PrimerF 278163 ................
  PrimerR   1666 .................A...
  PrimerR   1690 ..A..................
  PrimerR 281478 .....................
- - -
HG059-O3: N(allPCRHits)= 128031 (-5958)
HG059-O3: N(SingleHits)= 128031 (-5958)
HG059-O3: N(CleanHits) = 127774 (-6215)
◇ Amplicon Orientation:
   128031 HG059-O3-PF HG059-O3-PR
◇ Mis-Matches (top3):
  PrimerF    819 ............W...
  PrimerF   1260 C...............
  PrimerF 121822 ................
  PrimerR    627 ..A..................
  PrimerR    664 ..................A..
  PrimerR 122643 .....................
- - -
HG059-Q1: N(allPCRHits)= 566144 (-27976)
HG059-Q1: N(SingleHits)= 566144 (-27976)
HG059-Q1: N(CleanHits) = 564469 (-29651)
◇ Amplicon Orientation:
```

```
   566140 HG059-Q1-PF HG059-Q1-PR
        4 HG059-Q1-PR HG059-Q1-PF
◇ Mis-Matches (top3):
  PrimerF   4944 C...............
  PrimerF   8069 ...A............
  PrimerF 536538 ................
  PrimerR   4276 .................A...
  PrimerR   4373 ..A..................
  PrimerR 537805 .....................
- - -
HG059-Q2: N(allPCRHits)= 125725 (-10636)
HG059-Q2: N(SingleHits)= 125725 (-10636)
HG059-Q2: N(CleanHits) = 123734 (-12627)
◇ Amplicon Orientation:
   125725 HG059-Q2-PF HG059-Q2-PR
◇ Mis-Matches (top3):
  PrimerF   1289 C...............
  PrimerF   1682 ...............A
  PrimerF 117818 ................
  PrimerR    673 ..................A..
  PrimerR   1542 .................AA..
  PrimerR 118835 .....................
- - -
HG059-Q3: N(allPCRHits)= 162206 (-7514)
HG059-Q3: N(SingleHits)= 162206 (-7514)
HG059-Q3: N(CleanHits) = 161872 (-7848)
◇ Amplicon Orientation:
   162206 HG059-Q3-PF HG059-Q3-PR
◇ Mis-Matches (top3):
  PrimerF    931 ............W...
  PrimerF   1807 C...............
  PrimerF 154412 ................
  PrimerR    914 ..A..................
  PrimerR    931 ..................A..
  PrimerR 155110 .....................
- - -
HG059-R1: N(allPCRHits)= 319364 (-16958)
HG059-R1: N(SingleHits)= 319364 (-16958)
HG059-R1: N(CleanHits) = 318285 (-18037)
◇ Amplicon Orientation:
   319364 HG059-R1-PF HG059-R1-PR
◇ Mis-Matches (top3):
  PrimerF   3079 C...............
  PrimerF   4808 ...A............
  PrimerF 301541 ................
  PrimerR   2266 .................A...
  PrimerR   2284 ..A..................
  PrimerR 303899 .....................
- - -
HG059-R2: N(allPCRHits)= 191979 (-10051)
HG059-R2: N(SingleHits)= 191979 (-10051)
HG059-R2: N(CleanHits) = 191490 (-10540)
◇ Amplicon Orientation:
   191979 HG059-R2-PF HG059-R2-PR
```

```
◇ Mis-Matches (top3):
  PrimerF   1700 ............W...
  PrimerF   1750 C...............
  PrimerF 182054 ................
  PrimerR    962 ..................A..
  PrimerR    979 ..A..................
  PrimerR 183740 .....................
- - -
HG059-R3: N(allPCRHits)= 239235 (-12380)
HG059-R3: N(SingleHits)= 239235 (-12380)
HG059-R3: N(CleanHits) = 238091 (-13524)
◇ Amplicon Orientation:
   239235 HG059-R3-PF HG059-R3-PR
◇ Mis-Matches (top3):
  PrimerF   1216 ............W...
  PrimerF   2234 C...............
  PrimerF 227850 ................
  PrimerR   1083 .................A...
  PrimerR   1217 ..A..................
  PrimerR 228979 .....................
- - -
HG082-B1: N(allPCRHits)= 220239 (-12917)
HG082-B1: N(SingleHits)= 220239 (-12917)
HG082-B1: N(CleanHits) = 219448 (-13708)
◇ Amplicon Orientation:
   220239 HG082-B1-PF HG082-B1-PR
◇ Mis-Matches (top3):
  PrimerF   1918 C...............
  PrimerF   3197 ...A............
  PrimerF 208001 ................
  PrimerR   1234 ..A..................
  PrimerR   1402 .................A...
  PrimerR 210236 .....................
- - -
HG082-B2: N(allPCRHits)= 386768 (-20970)
HG082-B2: N(SingleHits)= 386768 (-20970)
HG082-B2: N(CleanHits) = 385793 (-21945)
◇ Amplicon Orientation:
   386767 HG082-B2-PF HG082-B2-PR
        1 HG082-B2-PR HG082-B2-PF
◇ Mis-Matches (top3):
  PrimerF   2079 ...A............
  PrimerF   3547 C...............
  PrimerF 367994 ................
  PrimerR   1964 .................A...
  PrimerR   2104 ..A..................
  PrimerR 370923 .....................
- - -
HG082-B3: N(allPCRHits)= 169880 (-9910)
HG082-B3: N(SingleHits)= 169878 (-9912)
HG082-B3: N(CleanHits) = 169473 (-10317)
◇ Amplicon Orientation:
   169880 HG082-B3-PF HG082-B3-PR
◇ Mis-Matches (top3):
```

```
  PrimerF   1578 ............W...
  PrimerF   1771 C...............
  PrimerF 160298 ................
  PrimerR    873 ..................A..
  PrimerR    875 ..A..................
  PrimerR 162740 .....................
- - -
HG082-D1: N(allPCRHits)= 179950 (-16358)
HG082-D1: N(SingleHits)= 179950 (-16358)
HG082-D1: N(CleanHits) = 178059 (-18249)
◇ Amplicon Orientation:
   179950 HG082-D1-PF HG082-D1-PR
◇ Mis-Matches (top3):
  PrimerF   1657 C...............
  PrimerF   2446 ...A............
  PrimerF 168722 ................
  PrimerR   1164 .................AA..
  PrimerR   1332 .................A...
  PrimerR 170137 .....................
- - -
HG082-D2: N(allPCRHits)= 223890 (-12079)
HG082-D2: N(SingleHits)= 223890 (-12079)
HG082-D2: N(CleanHits) = 223392 (-12577)
◇ Amplicon Orientation:
   223890 HG082-D2-PF HG082-D2-PR
◇ Mis-Matches (top3):
  PrimerF   1805 ............W...
  PrimerF   2002 C...............
  PrimerF 212417 ................
  PrimerR   1111 ..................A..
  PrimerR   1122 ..A..................
  PrimerR 214891 .....................
- - -
HG082-D3: N(allPCRHits)= 171331 (-10347)
HG082-D3: N(SingleHits)= 171329 (-10349)
HG082-D3: N(CleanHits) = 170600 (-11078)
◇ Amplicon Orientation:
   171331 HG082-D3-PF HG082-D3-PR
◇ Mis-Matches (top3):
  PrimerF   1389 ............W...
  PrimerF   1596 C...............
  PrimerF 162364 ................
  PrimerR    779 .................A...
  PrimerR    880 ..................A..
  PrimerR 163922 .....................
- - -
HG082-I1: N(allPCRHits)= 104676 (-27484)
HG082-I1: N(SingleHits)= 104676 (-27484)
HG082-I1: N(CleanHits) = 91403 (-40757)
◇ Amplicon Orientation:
   104676 HG082-I1-PF HG082-I1-PR
◇ Mis-Matches (top3):
  PrimerF   1207 ...A............
  PrimerF  12386 ...............A
```

```
  PrimerF  86970 ................
  PrimerR   1418 .................A.T.
  PrimerR  11736 .................AA..
  PrimerR  86447 ....................
- - -
HG082-I2: N(allPCRHits)= 246585 (-11665)
HG082-I2: N(SingleHits)= 246585 (-11665)
HG082-I2: N(CleanHits) = 245759 (-12491)
◇ Amplicon Orientation:
   246585 HG082-I2-PF HG082-I2-PR
◇ Mis-Matches (top3):
  PrimerF   2339 C...............
  PrimerF   3509 ...A............
  PrimerF 234298 ................
  PrimerR   1207 ..A.................
  PrimerR   1713 .................A...
  PrimerR 235553 ....................
- - -
HG082-I3: N(allPCRHits)= 270485 (-15494)
HG082-I3: N(SingleHits)= 270483 (-15496)
HG082-I3: N(CleanHits) = 269305 (-16674)
◇ Amplicon Orientation:
   270485 HG082-I3-PF HG082-I3-PR
◇ Mis-Matches (top3):
  PrimerF   1909 ............W...
  PrimerF   2488 C...............
  PrimerF 256550 ................
  PrimerR   1487 ..................A..
  PrimerR   1952 ..A.................
  PrimerR 258188 ....................
- - -
HG082-J1: N(allPCRHits)= 242386 (-11735)
HG082-J1: N(SingleHits)= 242386 (-11735)
HG082-J1: N(CleanHits) = 241735 (-12386)
◇ Amplicon Orientation:
   242386 HG082-J1-PF HG082-J1-PR
◇ Mis-Matches (top3):
  PrimerF   2390 C...............
  PrimerF   3542 ...A............
  PrimerF 230208 ................
  PrimerR   1236 ..A.................
  PrimerR   1630 .................A...
  PrimerR 231994 ....................
- - -
HG082-J2: N(allPCRHits)= 213606 (-10725)
HG082-J2: N(SingleHits)= 213606 (-10725)
HG082-J2: N(CleanHits) = 213048 (-11283)
◇ Amplicon Orientation:
   213606 HG082-J2-PF HG082-J2-PR
◇ Mis-Matches (top3):
  PrimerF   2297 C...............
  PrimerF   2597 ...A............
  PrimerF 202248 ................
  PrimerR    834 ..................A..
```

```
  PrimerR   1463 .................A...
  PrimerR 205004 .....................
- - -
HG082-J3: N(allPCRHits)= 145538 (-8549)
HG082-J3: N(SingleHits)= 145538 (-8549)
HG082-J3: N(CleanHits) = 145248 (-8839)
◇ Amplicon Orientation:
   145538 HG082-J3-PF HG082-J3-PR
◇ Mis-Matches (top3):
  PrimerF   1120 ............W...
  PrimerF   1425 C...............
  PrimerF 137883 ................
  PrimerR    640 ..................A..
  PrimerR    914 ..A..................
  PrimerR 139367 .....................
- - -
HG082-L1: N(allPCRHits)= 260950 (-12970)
HG082-L1: N(SingleHits)= 260950 (-12970)
HG082-L1: N(CleanHits) = 260088 (-13832)
◇ Amplicon Orientation:
   260949 HG082-L1-PF HG082-L1-PR
        1 HG082-L1-PR HG082-L1-PF
◇ Mis-Matches (top3):
  PrimerF   2490 C...............
  PrimerF   3945 ...A............
  PrimerF 247191 ................
  PrimerR   1758 ..A..................
  PrimerR   1862 .................A...
  PrimerR 248636 .....................
- - -
HG082-L2: N(allPCRHits)= 570171 (-78928)
HG082-L2: N(SingleHits)= 570171 (-78928)
HG082-L2: N(CleanHits) = 564421 (-84678)
◇ Amplicon Orientation:
   570171 HG082-L2-PF HG082-L2-PR
◇ Mis-Matches (top3):
  PrimerF   5492 C...............
  PrimerF   8130 ...A............
  PrimerF 535532 ................
  PrimerR   3006 ..A..................
  PrimerR   3904 .................A...
  PrimerR 540717 .....................
- - -
HG082-L3: N(allPCRHits)= 124070 (-8267)
HG082-L3: N(SingleHits)= 124070 (-8267)
HG082-L3: N(CleanHits) = 123728 (-8609)
◇ Amplicon Orientation:
   124070 HG082-L3-PF HG082-L3-PR
◇ Mis-Matches (top3):
  PrimerF   1160 ............W...
  PrimerF   1202 C...............
  PrimerF 117141 ................
  PrimerR    792 ..................A..
  PrimerR   1000 ..A..................
```

```
  PrimerR 118075 ....................
- - -
HG082-O1: N(allPCRHits)= 177134 (-16773)
HG082-O1: N(SingleHits)= 177134 (-16773)
HG082-O1: N(CleanHits) = 171235 (-22672)
◇ Amplicon Orientation:
   177134 HG082-O1-PF HG082-O1-PR
◇ Mis-Matches (top3):
  PrimerF   2466 ...A...........
  PrimerF   5377 ...............A
  PrimerF 162681 ................
  PrimerR   1024 .................A...
  PrimerR   5421 .................AA..
  PrimerR 163749 ....................
- - -
HG082-O2: N(allPCRHits)= 60362 (-53535)
HG082-O2: N(SingleHits)= 60362 (-53535)
HG082-O2: N(CleanHits) = 29447 (-84450)
◇ Amplicon Orientation:
    60362 HG082-O2-PF HG082-O2-PR
◇ Mis-Matches (top3):
  PrimerF   1146 ..............CA
  PrimerF  28025 ................
  PrimerF  28583 ...............A
  PrimerR    793 ..................A.C
  PrimerR  27388 ....................
  PrimerR  27540 .................AA..
- - -
HG082-O3: N(allPCRHits)= 268191 (-17240)
HG082-O3: N(SingleHits)= 268191 (-17240)
HG082-O3: N(CleanHits) = 265524 (-19907)
◇ Amplicon Orientation:
   268191 HG082-O3-PF HG082-O3-PR
◇ Mis-Matches (top3):
  PrimerF   2257 ...............A
  PrimerF   2626 C...............
  PrimerF 253101 ................
  PrimerR   1700 ..................A..
  PrimerR   2001 .................AA..
  PrimerR 254107 ....................
- - -
HG082-Q1: N(allPCRHits)= 162645 (-33591)
HG082-Q1: N(SingleHits)= 162645 (-33591)
HG082-Q1: N(CleanHits) = 152738 (-43498)
◇ Amplicon Orientation:
   162645 HG082-Q1-PF HG082-Q1-PR
◇ Mis-Matches (top3):
  PrimerF   2187 ...A...........
  PrimerF   8916 ...............A
  PrimerF 145185 ................
  PrimerR    987 ..A.................
  PrimerR   8063 .................AA..
  PrimerR 146325 ....................
- - -
```

```
HG082-Q2: N(allPCRHits)= 143137 (-8430)
HG082-Q2: N(SingleHits)= 143137 (-8430)
HG082-Q2: N(CleanHits) = 141907 (-9660)
◇ Amplicon Orientation:
   143137 HG082-Q2-PF HG082-Q2-PR
◇ Mis-Matches (top3):
  PrimerF    996 ...............A
  PrimerF   1387 C...............
  PrimerF 135689 ................
  PrimerR    774 ..................A..
  PrimerR    827 .................AA..
  PrimerR 136202 .....................
- - -
HG082-Q3: N(allPCRHits)= 246466 (-18035)
HG082-Q3: N(SingleHits)= 246466 (-18035)
HG082-Q3: N(CleanHits) = 244948 (-19553)
◇ Amplicon Orientation:
   246466 HG082-Q3-PF HG082-Q3-PR
◇ Mis-Matches (top3):
  PrimerF   2115 ............W...
  PrimerF   2430 C...............
  PrimerF 232265 ................
  PrimerR   1345 ..A..................
  PrimerR   1658 ..................A..
  PrimerR 234301 .....................
- - -
HG082-R1: N(allPCRHits)= 309911 (-16776)
HG082-R1: N(SingleHits)= 309911 (-16776)
HG082-R1: N(CleanHits) = 307867 (-18820)
◇ Amplicon Orientation:
   309911 HG082-R1-PF HG082-R1-PR
◇ Mis-Matches (top3):
  PrimerF   2995 C...............
  PrimerF   4308 ...A............
  PrimerF 292837 ................
  PrimerR   2045 ..A..................
  PrimerR   2087 .................A...
  PrimerR 294669 .....................
- - -
HG082-R2: N(allPCRHits)= 167639 (-8763)
HG082-R2: N(SingleHits)= 167639 (-8763)
HG082-R2: N(CleanHits) = 166787 (-9615)
◇ Amplicon Orientation:
   167639 HG082-R2-PF HG082-R2-PR
◇ Mis-Matches (top3):
  PrimerF   1102 ...A............
  PrimerF   1450 C...............
  PrimerF 159638 ................
  PrimerR    743 ..................A..
  PrimerR    985 ..A..................
  PrimerR 160476 .....................
- - -
HG082-R3: N(allPCRHits)= 340935 (-36650)
HG082-R3: N(SingleHits)= 340935 (-36650)
```

```
HG082-R3: N(CleanHits) = 334785 (-42800)
◇ Amplicon Orientation:
   340935 HG082-R3-PF HG082-R3-PR
◇ Mis-Matches (top3):
  PrimerF   3224 C...............
  PrimerF   5204 ...............A
  PrimerF 318523 ................
  PrimerR   1997 ..................A..
  PrimerR   4829 .................AA..
  PrimerR 320760 .....................
- - -
HG091-B1: N(allPCRHits)= 21487 (-4299)
HG091-B1: N(SingleHits)= 21487 (-4299)
HG091-B1: N(CleanHits) = 21047 (-4739)
◇ Amplicon Orientation:
    21487 HG091-B1-PF HG091-B1-PR
◇ Mis-Matches (top3):
  PrimerF    342 ...A............
  PrimerF    372 ...............A
  PrimerF  19912 ................
  PrimerR    170 ..A..................
  PrimerR    270 .................AA..
  PrimerR  20053 .....................
- - -
HG091-B2: N(allPCRHits)= 129113 (-22441)
HG091-B2: N(SingleHits)= 129113 (-22441)
HG091-B2: N(CleanHits) = 127672 (-23882)
◇ Amplicon Orientation:
   129112 HG091-B2-PF HG091-B2-PR
        1 HG091-B2-PR HG091-B2-PF
◇ Mis-Matches (top3):
  PrimerF   1222 ...............A
  PrimerF   1238 C...............
  PrimerF 121342 ................
  PrimerR    650 ..A..................
  PrimerR   1132 .................AA..
  PrimerR 122657 .....................
- - -
HG091-B3: N(allPCRHits)= 269252 (-17551)
HG091-B3: N(SingleHits)= 269252 (-17551)
HG091-B3: N(CleanHits) = 268425 (-18378)
◇ Amplicon Orientation:
   269252 HG091-B3-PF HG091-B3-PR
◇ Mis-Matches (top3):
  PrimerF   2515 C...............
  PrimerF   2597 ............W...
  PrimerF 254365 ................
  PrimerR   1264 ..A..................
  PrimerR   1362 ..................A..
  PrimerR 257560 .....................
- - -
HG091-D1: N(allPCRHits)= 41006 (-6670)
HG091-D1: N(SingleHits)= 41006 (-6670)
HG091-D1: N(CleanHits) = 40105 (-7571)
```

```
◇ Amplicon Orientation:
    41005 HG091-D1-PF HG091-D1-PR
        1 HG091-D1-PR HG091-D1-PF
◇ Mis-Matches (top3):
  PrimerF    643 ...A...........
  PrimerF    777 ...............A
  PrimerF  37955 ...............
  PrimerR    466 ..A..................
  PrimerR    634 .................AA..
  PrimerR  37739 .....................
- - -
HG091-D2: N(allPCRHits)= 161722 (-26196)
HG091-D2: N(SingleHits)= 161722 (-26196)
HG091-D2: N(CleanHits) = 159497 (-28421)
◇ Amplicon Orientation:
   161722 HG091-D2-PF HG091-D2-PR
◇ Mis-Matches (top3):
  PrimerF   1429 C...............
  PrimerF   1915 ...............A
  PrimerF 151354 ...............
  PrimerR    898 ..A..................
  PrimerR   1621 .................AA..
  PrimerR 152757 .....................
- - -
HG091-D3: N(allPCRHits)= 38332 (-30311)
HG091-D3: N(SingleHits)= 38332 (-30311)
HG091-D3: N(CleanHits) = 34155 (-34488)
◇ Amplicon Orientation:
    38332 HG091-D3-PF HG091-D3-PR
◇ Mis-Matches (top3):
  PrimerF    307 C...............
  PrimerF   3876 ...............A
  PrimerF  32512 ...............
  PrimerR    214 ..................A..
  PrimerR   3759 .................AA..
  PrimerR  32759 .....................
- - -
HG091-I1: N(allPCRHits)= 235458 (-14286)
HG091-I1: N(SingleHits)= 235458 (-14286)
HG091-I1: N(CleanHits) = 232615 (-17129)
◇ Amplicon Orientation:
   235458 HG091-I1-PF HG091-I1-PR
◇ Mis-Matches (top3):
  PrimerF   2532 ...............A
  PrimerF   3231 ...A...........
  PrimerF 221899 ...............
  PrimerR   1450 .................A...
  PrimerR   2222 .................AA..
  PrimerR 223301 .....................
- - -
HG091-I2: N(allPCRHits)= 256147 (-28290)
HG091-I2: N(SingleHits)= 256147 (-28290)
HG091-I2: N(CleanHits) = 248907 (-35530)
◇ Amplicon Orientation:
```

```
   256147 HG091-I2-PF HG091-I2-PR
◇ Mis-Matches (top3):
  PrimerF   3583 ...A............
  PrimerF   6463 ...............A
  PrimerF 236101 ................
  PrimerR   1609 .................A...
  PrimerR   5952 .................AA..
  PrimerR 238455 .....................
- - -
HG091-I3: N(allPCRHits)= 139533 (-10315)
HG091-I3: N(SingleHits)= 139533 (-10315)
HG091-I3: N(CleanHits) = 138665 (-11183)
◇ Amplicon Orientation:
   139533 HG091-I3-PF HG091-I3-PR
◇ Mis-Matches (top3):
  PrimerF    798 ............W...
  PrimerF   1339 C...............
  PrimerF 132117 ................
  PrimerR    839 ..................A..
  PrimerR    928 ..A..................
  PrimerR 132397 .....................
- - -
HG091-J1: N(allPCRHits)= 4515 (-3387)
HG091-J1: N(SingleHits)= 4515 (-3387)
HG091-J1: N(CleanHits) = 2995 (-4907)
◇ Amplicon Orientation:
     4515 HG091-J1-PF HG091-J1-PR
◇ Mis-Matches (top3):
  PrimerF     82 ..............CA
  PrimerF   1338 ...............A
  PrimerF   2884 ................
  PrimerR     36 .................A...
  PrimerR   1371 .................AA..
  PrimerR   2825 .....................
- - -
HG091-J2: N(allPCRHits)= 204190 (-34108)
HG091-J2: N(SingleHits)= 204190 (-34108)
HG091-J2: N(CleanHits) = 197309 (-40989)
◇ Amplicon Orientation:
   204190 HG091-J2-PF HG091-J2-PR
◇ Mis-Matches (top3):
  PrimerF   2635 ...A............
  PrimerF   6279 ...............A
  PrimerF 187239 ................
  PrimerR   1089 .................A...
  PrimerR   5446 .................AA..
  PrimerR 189701 .....................
- - -
HG091-J3: N(allPCRHits)= 77960 (-38216)
HG091-J3: N(SingleHits)= 77960 (-38216)
HG091-J3: N(CleanHits) = 71325 (-44851)
◇ Amplicon Orientation:
    77960 HG091-J3-PF HG091-J3-PR
◇ Mis-Matches (top3):
```

```
  PrimerF    725 C...............
  PrimerF   6153 ...............A
  PrimerF  67756 ................
  PrimerR    524 ..................A..
  PrimerR   5768 .................AA..
  PrimerR  68194 .....................
- - -
HG091-L1: N(allPCRHits)= 7403 (-5685)
HG091-L1: N(SingleHits)= 7403 (-5685)
HG091-L1: N(CleanHits) = 5574 (-7514)
◇ Amplicon Orientation:
     7403 HG091-L1-PF HG091-L1-PR
◇ Mis-Matches (top3):
  PrimerF     80 ...A............
  PrimerF   1632 ...............A
  PrimerF   5322 ................
  PrimerR     39 .................A...
  PrimerR   1651 .................AA..
  PrimerR   5320 .....................
- - -
HG091-L2: N(allPCRHits)= 252836 (-20429)
HG091-L2: N(SingleHits)= 252836 (-20429)
HG091-L2: N(CleanHits) = 248668 (-24597)
◇ Amplicon Orientation:
   252836 HG091-L2-PF HG091-L2-PR
◇ Mis-Matches (top3):
  PrimerF   3479 ...A............
  PrimerF   3725 ...............A
  PrimerF 237042 ................
  PrimerR   1625 .................A...
  PrimerR   3158 .................AA..
  PrimerR 238887 .....................
- - -
HG091-L3: N(allPCRHits)= 107280 (-12330)
HG091-L3: N(SingleHits)= 107280 (-12330)
HG091-L3: N(CleanHits) = 106115 (-13495)
◇ Amplicon Orientation:
   107279 HG091-L3-PF HG091-L3-PR
        1 HG091-L3-PR HG091-L3-PF
◇ Mis-Matches (top3):
  PrimerF   1054 C...............
  PrimerF   1170 ............W...
  PrimerF 100062 ................
  PrimerR    868 .................AA..
  PrimerR    976 ..A..................
  PrimerR 100889 .....................
- - -
HG091-O1: N(allPCRHits)= 362445 (-29571)
HG091-O1: N(SingleHits)= 362445 (-29571)
HG091-O1: N(CleanHits) = 354354 (-37662)
◇ Amplicon Orientation:
   362445 HG091-O1-PF HG091-O1-PR
◇ Mis-Matches (top3):
  PrimerF   5204 ...A............
```

```
  PrimerF   7251 ...............A
  PrimerF 336819 ................
  PrimerR   2484 .................A...
  PrimerR   6693 .................AA..
  PrimerR 338706 .....................
- - -
HG091-O2: N(allPCRHits)= 78268 (-16937)
HG091-O2: N(SingleHits)= 78268 (-16937)
HG091-O2: N(CleanHits) = 75839 (-19366)
◇ Amplicon Orientation:
    78268 HG091-O2-PF HG091-O2-PR
◇ Mis-Matches (top3):
  PrimerF    780 C...............
  PrimerF   2226 ...............A
  PrimerF  72201 ................
  PrimerR    554 ..................A..
  PrimerR   2010 .................AA..
  PrimerR  72503 .....................
- - -
HG091-O3: N(allPCRHits)= 149490 (-13511)
HG091-O3: N(SingleHits)= 149490 (-13511)
HG091-O3: N(CleanHits) = 146224 (-16777)
◇ Amplicon Orientation:
   149490 HG091-O3-PF HG091-O3-PR
◇ Mis-Matches (top3):
  PrimerF   1489 C...............
  PrimerF   2907 ...............A
  PrimerF 139326 ................
  PrimerR    702 ..................A..
  PrimerR   2632 .................AA..
  PrimerR 140634 .....................
- - -
HG091-Q1: N(allPCRHits)= 209225 (-29032)
HG091-Q1: N(SingleHits)= 209225 (-29032)
HG091-Q1: N(CleanHits) = 198160 (-40097)
◇ Amplicon Orientation:
   209225 HG091-Q1-PF HG091-Q1-PR
◇ Mis-Matches (top3):
  PrimerF   2712 ...A............
  PrimerF  10154 ...............A
  PrimerF 188685 ................
  PrimerR   1318 .................A...
  PrimerR   9893 .................AA..
  PrimerR 189658 .....................
- - -
HG091-Q2: N(allPCRHits)= 152469 (-8620)
HG091-Q2: N(SingleHits)= 152469 (-8620)
HG091-Q2: N(CleanHits) = 151370 (-9719)
◇ Amplicon Orientation:
   152469 HG091-Q2-PF HG091-Q2-PR
◇ Mis-Matches (top3):
  PrimerF   1013 ............W...
  PrimerF   1424 C...............
  PrimerF 144648 ................
```

```
  PrimerR    692 .................A..
  PrimerR    832 ................AA..
  PrimerR 146034 ....................
- - -
HG091-Q3: N(allPCRHits)= 347491 (-31596)
HG091-Q3: N(SingleHits)= 347487 (-31600)
HG091-Q3: N(CleanHits) = 342721 (-36366)
◇ Amplicon Orientation:
   347491 HG091-Q3-PF HG091-Q3-PR
◇ Mis-Matches (top3):
  PrimerF   3380 C...............
  PrimerF   4102 ...............A
  PrimerF 326592 ................
  PrimerR   1908 .................A..
  PrimerR   3610 ................AA..
  PrimerR 329023 ....................
- - -
HG091-R1: N(allPCRHits)= 87569 (-66348)
HG091-R1: N(SingleHits)= 87569 (-66348)
HG091-R1: N(CleanHits) = 56123 (-97794)
◇ Amplicon Orientation:
    87569 HG091-R1-PF HG091-R1-PR
◇ Mis-Matches (top3):
  PrimerF    750 ...A............
  PrimerF  29269 ...............A
  PrimerF  53158 ................
  PrimerR   1443 .................AT.
  PrimerR  27526 ................AA..
  PrimerR  52960 ....................
- - -
HG091-R2: N(allPCRHits)= 89386 (-42594)
HG091-R2: N(SingleHits)= 89386 (-42594)
HG091-R2: N(CleanHits) = 76973 (-55007)
◇ Amplicon Orientation:
    89386 HG091-R2-PF HG091-R2-PR
◇ Mis-Matches (top3):
  PrimerF    751 C...............
  PrimerF  11446 ...............A
  PrimerF  73424 ................
  PrimerR    524 .................AT.
  PrimerR  10342 ................AA..
  PrimerR  74428 ....................
- - -
HG091-R3: N(allPCRHits)= 176802 (-28272)
HG091-R3: N(SingleHits)= 176800 (-28274)
HG091-R3: N(CleanHits) = 172437 (-32637)
◇ Amplicon Orientation:
   176801 HG091-R3-PF HG091-R3-PR
        1 HG091-R3-PR HG091-R3-PF
◇ Mis-Matches (top3):
  PrimerF   1848 C...............
  PrimerF   3780 ...............A
  PrimerF 163524 ................
  PrimerR    893 ..A.................
```

```
  PrimerR   3533 .................AA..
  PrimerR 165908 .....................
- - -
HG141-B1: N(allPCRHits)= 121864 (-9139)
HG141-B1: N(SingleHits)= 121864 (-9139)
HG141-B1: N(CleanHits) = 121331 (-9672)
◇ Amplicon Orientation:
   121863 HG141-B1-PF HG141-B1-PR
        1 HG141-B1-PR HG141-B1-PF
◇ Mis-Matches (top3):
  PrimerF   1020 C...............
  PrimerF   1832 ...A............
  PrimerF 114721 ................
  PrimerR    938 .................A...
  PrimerR   1260 ..A..................
  PrimerR 115012 .....................
- - -
HG141-B2: N(allPCRHits)= 235328 (-12263)
HG141-B2: N(SingleHits)= 235328 (-12263)
HG141-B2: N(CleanHits) = 234688 (-12903)
◇ Amplicon Orientation:
   235328 HG141-B2-PF HG141-B2-PR
◇ Mis-Matches (top3):
  PrimerF   1957 ............W...
  PrimerF   2206 C...............
  PrimerF 222373 ................
  PrimerR   1447 ..................A..
  PrimerR   1552 ..A..................
  PrimerR 224784 .....................
- - -
HG141-B3: N(allPCRHits)= 296327 (-14128)
HG141-B3: N(SingleHits)= 296327 (-14128)
HG141-B3: N(CleanHits) = 295625 (-14830)
◇ Amplicon Orientation:
   296327 HG141-B3-PF HG141-B3-PR
◇ Mis-Matches (top3):
  PrimerF   1905 ............W...
  PrimerF   2740 C...............
  PrimerF 282270 ................
  PrimerR   1661 ..................A..
  PrimerR   2654 .................A...
  PrimerR 282432 .....................
- - -
HG141-D1: N(allPCRHits)= 47864 (-3180)
HG141-D1: N(SingleHits)= 47864 (-3180)
HG141-D1: N(CleanHits) = 47630 (-3414)
◇ Amplicon Orientation:
    47864 HG141-D1-PF HG141-D1-PR
◇ Mis-Matches (top3):
  PrimerF    428 C...............
  PrimerF    659 ...A............
  PrimerF  45214 ................
  PrimerR    345 .................A...
  PrimerR    448 ..A..................
```

```
  PrimerR  45119 ....................
- - -
HG141-D2: N(allPCRHits)= 355510 (-18261)
HG141-D2: N(SingleHits)= 355510 (-18261)
HG141-D2: N(CleanHits) = 354649 (-19122)
◇ Amplicon Orientation:
   355509 HG141-D2-PF HG141-D2-PR
        1 HG141-D2-PR HG141-D2-PF
◇ Mis-Matches (top3):
  PrimerF   3278 ............W...
  PrimerF   3821 C...............
  PrimerF 334866 ................
  PrimerR   2249 .................A..
  PrimerR   2407 ..A.................
  PrimerR 339321 ....................
- - -
HG141-D3: N(allPCRHits)= 224541 (-10753)
HG141-D3: N(SingleHits)= 224541 (-10753)
HG141-D3: N(CleanHits) = 223889 (-11405)
◇ Amplicon Orientation:
   224541 HG141-D3-PF HG141-D3-PR
◇ Mis-Matches (top3):
  PrimerF   1177 ...A............
  PrimerF   2187 C...............
  PrimerF 213952 ................
  PrimerR   1063 ..A.................
  PrimerR   1329 .................A..
  PrimerR 214996 ....................
- - -
HG141-I1: N(allPCRHits)= 117138 (-7043)
HG141-I1: N(SingleHits)= 117138 (-7043)
HG141-I1: N(CleanHits) = 116669 (-7512)
◇ Amplicon Orientation:
   117138 HG141-I1-PF HG141-I1-PR
◇ Mis-Matches (top3):
  PrimerF   1083 C...............
  PrimerF   1793 ...A............
  PrimerF 110487 ................
  PrimerR    881 ..A.................
  PrimerR    934 ................A...
  PrimerR 111131 ....................
- - -
HG141-I2: N(allPCRHits)= 237760 (-15699)
HG141-I2: N(SingleHits)= 237760 (-15699)
HG141-I2: N(CleanHits) = 236640 (-16819)
◇ Amplicon Orientation:
   237760 HG141-I2-PF HG141-I2-PR
◇ Mis-Matches (top3):
  PrimerF   2266 C...............
  PrimerF   3829 ...A............
  PrimerF 224402 ................
  PrimerR   1878 ..A.................
  PrimerR   2238 ................A...
  PrimerR 224487 ....................
```

```
- - -
HG141-I3: N(allPCRHits)= 153447 (-10116)
HG141-I3: N(SingleHits)= 153445 (-10118)
HG141-I3: N(CleanHits) = 153086 (-10477)
◇ Amplicon Orientation:
   153447 HG141-I3-PF HG141-I3-PR
◇ Mis-Matches (top3):
  PrimerF   1341 C...............
  PrimerF   1702 ............W...
  PrimerF 144643 ................
  PrimerR    912 ..................A..
  PrimerR   1227 ..A..................
  PrimerR 146030 .....................
- - -
HG141-J1: N(allPCRHits)= 162207 (-11510)
HG141-J1: N(SingleHits)= 162207 (-11510)
HG141-J1: N(CleanHits) = 161213 (-12504)
◇ Amplicon Orientation:
   162207 HG141-J1-PF HG141-J1-PR
◇ Mis-Matches (top3):
  PrimerF   1546 C...............
  PrimerF   2435 ...A............
  PrimerF 152662 ................
  PrimerR   1151 ..A..................
  PrimerR   1409 .................A...
  PrimerR 153110 .....................
- - -
HG141-J2: N(allPCRHits)= 296687 (-36470)
HG141-J2: N(SingleHits)= 296687 (-36470)
HG141-J2: N(CleanHits) = 291059 (-42098)
◇ Amplicon Orientation:
   296687 HG141-J2-PF HG141-J2-PR
◇ Mis-Matches (top3):
  PrimerF   4581 ...A............
  PrimerF   4795 ...............A
  PrimerF 275034 ................
  PrimerR   2069 ..A..................
  PrimerR   4087 .................AA..
  PrimerR 278177 .....................
- - -
HG141-J3: N(allPCRHits)= 220041 (-31114)
HG141-J3: N(SingleHits)= 220041 (-31114)
HG141-J3: N(CleanHits) = 216638 (-34517)
◇ Amplicon Orientation:
   220041 HG141-J3-PF HG141-J3-PR
◇ Mis-Matches (top3):
  PrimerF   1987 C...............
  PrimerF   3104 ...............A
  PrimerF 205650 ................
  PrimerR   1776 ..A..................
  PrimerR   2709 .................AA..
  PrimerR 207262 .....................
- - -
HG141-L1: N(allPCRHits)= 228405 (-13190)
```

```
HG141-L1: N(SingleHits)= 228405 (-13190)
HG141-L1: N(CleanHits) = 227198 (-14397)
◇ Amplicon Orientation:
   228405 HG141-L1-PF HG141-L1-PR
◇ Mis-Matches (top3):
  PrimerF   2196 C...............
  PrimerF   3361 ...A............
  PrimerF 215774 ................
  PrimerR   1554 ..A.................
  PrimerR   1799 .................A...
  PrimerR 216570 .....................
- - -
HG141-L2: N(allPCRHits)= 269222 (-15467)
HG141-L2: N(SingleHits)= 269220 (-15469)
HG141-L2: N(CleanHits) = 267921 (-16768)
◇ Amplicon Orientation:
   269222 HG141-L2-PF HG141-L2-PR
◇ Mis-Matches (top3):
  PrimerF   2620 C...............
  PrimerF   3986 ...A............
  PrimerF 254521 ................
  PrimerR   1735 ..A.................
  PrimerR   2058 .................A...
  PrimerR 255485 .....................
- - -
HG141-L3: N(allPCRHits)= 145641 (-8739)
HG141-L3: N(SingleHits)= 145641 (-8739)
HG141-L3: N(CleanHits) = 144842 (-9538)
◇ Amplicon Orientation:
   145641 HG141-L3-PF HG141-L3-PR
◇ Mis-Matches (top3):
  PrimerF   1205 ............W...
  PrimerF   1338 C...............
  PrimerF 137641 ................
  PrimerR    828 ..................A..
  PrimerR    955 ..A..................
  PrimerR 138672 .....................
- - -
HG141-O1: N(allPCRHits)= 597355 (-27782)
HG141-O1: N(SingleHits)= 597355 (-27782)
HG141-O1: N(CleanHits) = 595108 (-30029)
◇ Amplicon Orientation:
   597353 HG141-O1-PF HG141-O1-PR
        2 HG141-O1-PR HG141-O1-PF
◇ Mis-Matches (top3):
  PrimerF   5734 C...............
  PrimerF   8202 ...A............
  PrimerF 568480 ................
  PrimerR   2713 ..................A..
  PrimerR   3807 .................A...
  PrimerR 572038 .....................
- - -
HG141-O2: N(allPCRHits)= 225415 (-14854)
HG141-O2: N(SingleHits)= 225415 (-14854)
```

```
HG141-O2: N(CleanHits) = 224267 (-16002)
◇ Amplicon Orientation:
   225415 HG141-O2-PF HG141-O2-PR
◇ Mis-Matches (top3):
  PrimerF   1920 C...............
  PrimerF   2450 ............W...
  PrimerF 212588 ................
  PrimerR   1385 ..................A..
  PrimerR   1569 ..A..................
  PrimerR 214213 .....................
- - -
HG141-O3: N(allPCRHits)= 202265 (-11405)
HG141-O3: N(SingleHits)= 202265 (-11405)
HG141-O3: N(CleanHits) = 201732 (-11938)
◇ Amplicon Orientation:
   202265 HG141-O3-PF HG141-O3-PR
◇ Mis-Matches (top3):
  PrimerF   1718 ............W...
  PrimerF   1922 C...............
  PrimerF 191812 ................
  PrimerR    959 ..A..................
  PrimerR   1154 ..................A..
  PrimerR 193646 .....................
- - -
HG141-Q1: N(allPCRHits)= 184641 (-13329)
HG141-Q1: N(SingleHits)= 184641 (-13329)
HG141-Q1: N(CleanHits) = 183337 (-14633)
◇ Amplicon Orientation:
   184641 HG141-Q1-PF HG141-Q1-PR
◇ Mis-Matches (top3):
  PrimerF   1692 C...............
  PrimerF   2654 ...A............
  PrimerF 173864 ................
  PrimerR   1240 .................A...
  PrimerR   1432 ..A..................
  PrimerR 175011 .....................
- - -
HG141-Q2: N(allPCRHits)= 129929 (-7087)
HG141-Q2: N(SingleHits)= 129929 (-7087)
HG141-Q2: N(CleanHits) = 129461 (-7555)
◇ Amplicon Orientation:
   129929 HG141-Q2-PF HG141-Q2-PR
◇ Mis-Matches (top3):
  PrimerF   1104 ............W...
  PrimerF   1285 C...............
  PrimerF 123076 ................
  PrimerR    858 .................A...
  PrimerR   1223 ..................A..
  PrimerR 122972 .....................
- - -
HG141-Q3: N(allPCRHits)= 329661 (-26943)
HG141-Q3: N(SingleHits)= 329661 (-26943)
HG141-Q3: N(CleanHits) = 328682 (-27922)
◇ Amplicon Orientation:
```

```
   329661 HG141-Q3-PF HG141-Q3-PR
◇ Mis-Matches (top3):
  PrimerF   2862 C...............
  PrimerF   3557 ............W...
  PrimerF 310720 ................
  PrimerR   1834 ..................A..
  PrimerR   2039 ..A..................
  PrimerR 314490 .....................
- - -
HG141-R1: N(allPCRHits)= 244043 (-19544)
HG141-R1: N(SingleHits)= 244043 (-19544)
HG141-R1: N(CleanHits) = 241749 (-21838)
◇ Amplicon Orientation:
   244043 HG141-R1-PF HG141-R1-PR
◇ Mis-Matches (top3):
  PrimerF   2152 C...............
  PrimerF   4008 ...A............
  PrimerF 228866 ................
  PrimerR   1836 .................A...
  PrimerR   2369 ..A..................
  PrimerR 230025 .....................
- - -
HG141-R2: N(allPCRHits)= 70787 (-19513)
HG141-R2: N(SingleHits)= 70787 (-19513)
HG141-R2: N(CleanHits) = 64578 (-25722)
◇ Amplicon Orientation:
    70787 HG141-R2-PF HG141-R2-PR
◇ Mis-Matches (top3):
  PrimerF    609 C...............
  PrimerF   5824 ...............A
  PrimerF  61685 ................
  PrimerR    389 ..................A..
  PrimerR   5371 .................AA..
  PrimerR  62236 .....................
- - -
HG141-R3: N(allPCRHits)= 220491 (-12553)
HG141-R3: N(SingleHits)= 220485 (-12559)
HG141-R3: N(CleanHits) = 219823 (-13221)
◇ Amplicon Orientation:
   220491 HG141-R3-PF HG141-R3-PR
◇ Mis-Matches (top3):
  PrimerF   2053 C...............
  PrimerF   2061 ............W...
  PrimerF 208225 ................
  PrimerR   1053 ..................A..
  PrimerR   1538 ..A..................
  PrimerR 210669 .....................
- - -
HG225-B1: N(allPCRHits)= 58360 (-6717)
HG225-B1: N(SingleHits)= 58360 (-6717)
HG225-B1: N(CleanHits) = 57862 (-7215)
◇ Amplicon Orientation:
    58360 HG225-B1-PF HG225-B1-PR
◇ Mis-Matches (top3):
```

```
  PrimerF    460 C...............
  PrimerF    892 ...A............
  PrimerF  54755 ................
  PrimerR    421 .................A...
  PrimerR    616 ..A..................
  PrimerR  54869 .....................
- - -
HG225-B2: N(allPCRHits)= 283725 (-21138)
HG225-B2: N(SingleHits)= 283725 (-21138)
HG225-B2: N(CleanHits) = 282876 (-21987)
◇ Amplicon Orientation:
   283725 HG225-B2-PF HG225-B2-PR
◇ Mis-Matches (top3):
  PrimerF   2516 C...............
  PrimerF   2536 ............W...
  PrimerF 268043 ................
  PrimerR   1472 ..................A..
  PrimerR   1723 ..A..................
  PrimerR 271264 .....................
- - -
HG225-B3: N(allPCRHits)= 227943 (-11801)
HG225-B3: N(SingleHits)= 227941 (-11803)
HG225-B3: N(CleanHits) = 227318 (-12426)
◇ Amplicon Orientation:
   227943 HG225-B3-PF HG225-B3-PR
◇ Mis-Matches (top3):
  PrimerF   1137 ...A............
  PrimerF   1999 C...............
  PrimerF 217167 ................
  PrimerR   1096 ..A..................
  PrimerR   1282 ..................A..
  PrimerR 218156 .....................
- - -
HG225-D1: N(allPCRHits)= 52541 (-5524)
HG225-D1: N(SingleHits)= 52541 (-5524)
HG225-D1: N(CleanHits) = 52012 (-6053)
◇ Amplicon Orientation:
    52541 HG225-D1-PF HG225-D1-PR
◇ Mis-Matches (top3):
  PrimerF    440 ...............A
  PrimerF    763 ...A............
  PrimerF  49427 ................
  PrimerR    331 .................A...
  PrimerR    566 ..A..................
  PrimerR  49362 .....................
- - -
HG225-D2: N(allPCRHits)= 324946 (-20345)
HG225-D2: N(SingleHits)= 324946 (-20345)
HG225-D2: N(CleanHits) = 323874 (-21417)
◇ Amplicon Orientation:
   324946 HG225-D2-PF HG225-D2-PR
◇ Mis-Matches (top3):
  PrimerF   2585 ............W...
  PrimerF   2912 C...............
```

```
  PrimerF 307766 ................
  PrimerR   1743 ..................A..
  PrimerR   1947 ..A..................
  PrimerR 310313 .....................
- - -
HG225-D3: N(allPCRHits)= 185331 (-8388)
HG225-D3: N(SingleHits)= 185331 (-8388)
HG225-D3: N(CleanHits) = 184869 (-8850)
◇ Amplicon Orientation:
   185330 HG225-D3-PF HG225-D3-PR
        1 HG225-D3-PR HG225-D3-PF
◇ Mis-Matches (top3):
  PrimerF   1091 ...A............
  PrimerF   1946 C...............
  PrimerF 176585 ................
  PrimerR    917 ..................A..
  PrimerR   1194 .................A...
  PrimerR 177631 .....................
- - -
HG225-I1: N(allPCRHits)= 57901 (-8286)
HG225-I1: N(SingleHits)= 57901 (-8286)
HG225-I1: N(CleanHits) = 56787 (-9400)
◇ Amplicon Orientation:
    57901 HG225-I1-PF HG225-I1-PR
◇ Mis-Matches (top3):
  PrimerF    870 ...A............
  PrimerF    987 ...............A
  PrimerF  53810 ................
  PrimerR    363 ..A..................
  PrimerR    841 .................AA..
  PrimerR  54385 .....................
- - -
HG225-I2: N(allPCRHits)= 183641 (-39618)
HG225-I2: N(SingleHits)= 183641 (-39618)
HG225-I2: N(CleanHits) = 178384 (-44875)
◇ Amplicon Orientation:
   183640 HG225-I2-PF HG225-I2-PR
        1 HG225-I2-PR HG225-I2-PF
◇ Mis-Matches (top3):
  PrimerF   2475 ...A............
  PrimerF   4557 ...............A
  PrimerF 169018 ................
  PrimerR   1352 .................A...
  PrimerR   4216 .................AA..
  PrimerR 169520 .....................
- - -
HG225-I3: N(allPCRHits)= 171870 (-10965)
HG225-I3: N(SingleHits)= 171870 (-10965)
HG225-I3: N(CleanHits) = 170963 (-11872)
◇ Amplicon Orientation:
   171870 HG225-I3-PF HG225-I3-PR
◇ Mis-Matches (top3):
  PrimerF   1316 ............W...
  PrimerF   1547 C...............
```

```
  PrimerF 162642 ................
  PrimerR   1186 ..A.................
  PrimerR   1191 ..................A..
  PrimerR 163347 .....................
- - -
HG225-J1: N(allPCRHits)= 22712 (-7379)
HG225-J1: N(SingleHits)= 22712 (-7379)
HG225-J1: N(CleanHits) = 20642 (-9449)
◇ Amplicon Orientation:
    22712 HG225-J1-PF HG225-J1-PR
◇ Mis-Matches (top3):
  PrimerF    300 ...A............
  PrimerF   1862 ...............A
  PrimerF  19605 ................
  PrimerR    177 ..A..................
  PrimerR   1815 .................AA..
  PrimerR  19575 .....................
- - -
HG225-J2: N(allPCRHits)= 294924 (-57363)
HG225-J2: N(SingleHits)= 294924 (-57363)
HG225-J2: N(CleanHits) = 288981 (-63306)
◇ Amplicon Orientation:
   294924 HG225-J2-PF HG225-J2-PR
◇ Mis-Matches (top3):
  PrimerF   4406 ...A............
  PrimerF   5110 ...............A
  PrimerF 273294 ................
  PrimerR   2396 .................A...
  PrimerR   4585 .................AA..
  PrimerR 274351 .....................
- - -
HG225-J3: N(allPCRHits)= 162660 (-26263)
HG225-J3: N(SingleHits)= 162660 (-26263)
HG225-J3: N(CleanHits) = 159811 (-29112)
◇ Amplicon Orientation:
   162660 HG225-J3-PF HG225-J3-PR
◇ Mis-Matches (top3):
  PrimerF   1711 C...............
  PrimerF   2517 ...............A
  PrimerF 151293 ................
  PrimerR   1011 ..A..................
  PrimerR   2120 .................AA..
  PrimerR 153292 .....................
- - -
HG225-L1: N(allPCRHits)= 317830 (-17190)
HG225-L1: N(SingleHits)= 317830 (-17190)
HG225-L1: N(CleanHits) = 316257 (-18763)
◇ Amplicon Orientation:
   317830 HG225-L1-PF HG225-L1-PR
◇ Mis-Matches (top3):
  PrimerF   2860 C...............
  PrimerF   4588 ...A............
  PrimerF 300975 ................
  PrimerR   2146 ..A..................
```

```
  PrimerR   2509 .................A...
  PrimerR 301515 .....................
- - -
HG225-L2: N(allPCRHits)= 295192 (-16439)
HG225-L2: N(SingleHits)= 295192 (-16439)
HG225-L2: N(CleanHits) = 293447 (-18184)
◇ Amplicon Orientation:
   295192 HG225-L2-PF HG225-L2-PR
◇ Mis-Matches (top3):
  PrimerF   2817 C...............
  PrimerF   4030 ...A............
  PrimerF 279089 ................
  PrimerR   1886 .................A...
  PrimerR   1899 ..A..................
  PrimerR 281100 .....................
- - -
HG225-L3: N(allPCRHits)= 146080 (-11692)
HG225-L3: N(SingleHits)= 146080 (-11692)
HG225-L3: N(CleanHits) = 143691 (-14081)
◇ Amplicon Orientation:
   146080 HG225-L3-PF HG225-L3-PR
◇ Mis-Matches (top3):
  PrimerF   1346 C...............
  PrimerF   1976 ...............A
  PrimerF 136707 ................
  PrimerR   1012 ..................A..
  PrimerR   1645 .................AA..
  PrimerR 137488 .....................
- - -
HG225-O1: N(allPCRHits)= 261865 (-16796)
HG225-O1: N(SingleHits)= 261865 (-16796)
HG225-O1: N(CleanHits) = 260980 (-17681)
◇ Amplicon Orientation:
   261863 HG225-O1-PF HG225-O1-PR
        2 HG225-O1-PR HG225-O1-PF
◇ Mis-Matches (top3):
  PrimerF   2428 C...............
  PrimerF   3963 ...A............
  PrimerF 246689 ................
  PrimerR   1925 .................A...
  PrimerR   1973 ..A..................
  PrimerR 249124 .....................
- - -
HG225-O2: N(allPCRHits)= 34650 (-35029)
HG225-O2: N(SingleHits)= 34650 (-35029)
HG225-O2: N(CleanHits) = 26354 (-43325)
◇ Amplicon Orientation:
    34650 HG225-O2-PF HG225-O2-PR
◇ Mis-Matches (top3):
  PrimerF    282 ............W...
  PrimerF   7671 ...............A
  PrimerF  25027 ................
  PrimerR   1057 ...................TC
  PrimerR   6520 .................AA..
```

```
  PrimerR  24642 ......................
– – –
HG225-O3: N(allPCRHits)= 139060 (-8880)
HG225-O3: N(SingleHits)= 139058 (-8882)
HG225-O3: N(CleanHits) = 138186 (-9754)
◇ Amplicon Orientation:
   139060 HG225-O3-PF HG225-O3-PR
◇ Mis-Matches (top3):
  PrimerF   1204 C...............
  PrimerF   1270 ............W...
  PrimerF 131307 ................
  PrimerR    782 ..A...................
  PrimerR    945 ..................A..
  PrimerR 131937 .....................
– – –
HG225-Q1: N(allPCRHits)= 206973 (-11392)
HG225-Q1: N(SingleHits)= 206973 (-11392)
HG225-Q1: N(CleanHits) = 205306 (-13059)
◇ Amplicon Orientation:
   206973 HG225-Q1-PF HG225-Q1-PR
◇ Mis-Matches (top3):
  PrimerF   1818 C...............
  PrimerF   3080 ...A............
  PrimerF 194985 ................
  PrimerR   1396 .................A...
  PrimerR   1678 ..A..................
  PrimerR 195920 .....................
– – –
HG225-Q2: N(allPCRHits)= 109450 (-33656)
HG225-Q2: N(SingleHits)= 109450 (-33656)
HG225-Q2: N(CleanHits) = 103079 (-40027)
◇ Amplicon Orientation:
   109450 HG225-Q2-PF HG225-Q2-PR
◇ Mis-Matches (top3):
  PrimerF    996 C...............
  PrimerF   5660 ...............A
  PrimerF  97971 ................
  PrimerR    608 .................A...
  PrimerR   5421 .................AA..
  PrimerR  98400 .....................
– – –
HG225-Q3: N(allPCRHits)= 80390 (-14203)
HG225-Q3: N(SingleHits)= 80390 (-14203)
HG225-Q3: N(CleanHits) = 78389 (-16204)
◇ Amplicon Orientation:
    80390 HG225-Q3-PF HG225-Q3-PR
◇ Mis-Matches (top3):
  PrimerF    739 C...............
  PrimerF   1783 ...............A
  PrimerF  74399 ................
  PrimerR    452 ..A..................
  PrimerR   1702 .................AA..
  PrimerR  75378 .....................
– – –
```

```
HG225-R1: N(allPCRHits)= 182926 (-9076)
HG225-R1: N(SingleHits)= 182924 (-9078)
HG225-R1: N(CleanHits) = 182056 (-9946)
◇ Amplicon Orientation:
   182926 HG225-R1-PF HG225-R1-PR
◇ Mis-Matches (top3):
  PrimerF   1712 C...............
  PrimerF   2663 ...A............
  PrimerF 172958 ................
  PrimerR    891 .................A...
  PrimerR   1045 ..A..................
  PrimerR 175195 .....................
- - -
HG225-R2: N(allPCRHits)= 259175 (-18900)
HG225-R2: N(SingleHits)= 259173 (-18902)
HG225-R2: N(CleanHits) = 257456 (-20619)
◇ Amplicon Orientation:
   259175 HG225-R2-PF HG225-R2-PR
◇ Mis-Matches (top3):
  PrimerF   2043 ............W...
  PrimerF   2436 C...............
  PrimerF 244307 ................
  PrimerR   1484 ..A..................
  PrimerR   1532 ..................A..
  PrimerR 246059 .....................
- - -
HG225-R3: N(allPCRHits)= 20504 (-7428)
HG225-R3: N(SingleHits)= 20504 (-7428)
HG225-R3: N(CleanHits) = 19417 (-8515)
◇ Amplicon Orientation:
    20504 HG225-R3-PF HG225-R3-PR
◇ Mis-Matches (top3):
  PrimerF    186 ............W...
  PrimerF    974 ...............A
  PrimerF  18350 ................
  PrimerR    106 ..A..................
  PrimerR    867 .................AA..
  PrimerR  18581 .....................
- - -
HG319-B1: N(allPCRHits)= 116928 (-8324)
HG319-B1: N(SingleHits)= 116928 (-8324)
HG319-B1: N(CleanHits) = 116405 (-8847)
◇ Amplicon Orientation:
   116927 HG319-B1-PF HG319-B1-PR
        1 HG319-B1-PR HG319-B1-PF
◇ Mis-Matches (top3):
  PrimerF    945 C...............
  PrimerF   1767 ...A............
  PrimerF 110385 ................
  PrimerR    885 .................A...
  PrimerR   1301 ..A..................
  PrimerR 110716 .....................
- - -
HG319-B2: N(allPCRHits)= 185890 (-17273)
```

```
HG319-B2: N(SingleHits)= 185890 (-17273)
HG319-B2: N(CleanHits) = 185333 (-17830)
◇ Amplicon Orientation:
   185890 HG319-B2-PF HG319-B2-PR
◇ Mis-Matches (top3):
  PrimerF   1439 ............W...
  PrimerF   1627 C...............
  PrimerF 176450 ................
  PrimerR    764 ..A.................
  PrimerR   1120 ..................A..
  PrimerR 177856 ....................
- - -
HG319-B3: N(allPCRHits)= 298401 (-15417)
HG319-B3: N(SingleHits)= 298401 (-15417)
HG319-B3: N(CleanHits) = 297508 (-16310)
◇ Amplicon Orientation:
   298401 HG319-B3-PF HG319-B3-PR
◇ Mis-Matches (top3):
  PrimerF   1754 ............W...
  PrimerF   2660 C...............
  PrimerF 284304 ................
  PrimerR   1417 .................A...
  PrimerR   1720 ..................A..
  PrimerR 285383 ....................
- - -
HG319-D1: N(allPCRHits)= 86474 (-6226)
HG319-D1: N(SingleHits)= 86474 (-6226)
HG319-D1: N(CleanHits) = 86124 (-6576)
◇ Amplicon Orientation:
    86474 HG319-D1-PF HG319-D1-PR
◇ Mis-Matches (top3):
  PrimerF    703 C...............
  PrimerF   1343 ...A............
  PrimerF  81650 ................
  PrimerR    647 .................A...
  PrimerR    855 ..A.................
  PrimerR  81643 ....................
- - -
HG319-D2: N(allPCRHits)= 273756 (-32406)
HG319-D2: N(SingleHits)= 273756 (-32406)
HG319-D2: N(CleanHits) = 272970 (-33192)
◇ Amplicon Orientation:
   273756 HG319-D2-PF HG319-D2-PR
◇ Mis-Matches (top3):
  PrimerF   2221 ............W...
  PrimerF   2574 C...............
  PrimerF 259682 ................
  PrimerR   1064 ..A.................
  PrimerR   1436 ..................A..
  PrimerR 262373 ....................
- - -
HG319-D3: N(allPCRHits)= 204161 (-10171)
HG319-D3: N(SingleHits)= 204159 (-10173)
HG319-D3: N(CleanHits) = 203520 (-10812)
```

```
◇ Amplicon Orientation:
   204161 HG319-D3-PF HG319-D3-PR
◇ Mis-Matches (top3):
  PrimerF    858 ............W...
  PrimerF   1960 C...............
  PrimerF 195179 ................
  PrimerR   1113 .................A...
  PrimerR   1198 ..................A..
  PrimerR 194951 .....................
- - -
HG319-I1: N(allPCRHits)= 281371 (-17773)
HG319-I1: N(SingleHits)= 281371 (-17773)
HG319-I1: N(CleanHits) = 280496 (-18648)
◇ Amplicon Orientation:
   281371 HG319-I1-PF HG319-I1-PR
◇ Mis-Matches (top3):
  PrimerF   2723 C...............
  PrimerF   4261 ...A............
  PrimerF 266080 ................
  PrimerR   1710 ..................A..
  PrimerR   2388 .................A...
  PrimerR 267154 .....................
- - -
HG319-I2: N(allPCRHits)= 202243 (-27012)
HG319-I2: N(SingleHits)= 202243 (-27012)
HG319-I2: N(CleanHits) = 201432 (-27823)
◇ Amplicon Orientation:
   202242 HG319-I2-PF HG319-I2-PR
        1 HG319-I2-PR HG319-I2-PF
◇ Mis-Matches (top3):
  PrimerF   2050 C...............
  PrimerF   3064 ...A............
  PrimerF 190306 ................
  PrimerR   1498 .................A...
  PrimerR   1591 ..A..................
  PrimerR 191847 .....................
- - -
HG319-I3: N(allPCRHits)= 236087 (-13035)
HG319-I3: N(SingleHits)= 236087 (-13035)
HG319-I3: N(CleanHits) = 235527 (-13595)
◇ Amplicon Orientation:
   236087 HG319-I3-PF HG319-I3-PR
◇ Mis-Matches (top3):
  PrimerF   1880 ............W...
  PrimerF   2116 C...............
  PrimerF 223670 ................
  PrimerR   1467 ..A..................
  PrimerR   1548 ..................A..
  PrimerR 224997 .....................
- - -
HG319-J1: N(allPCRHits)= 201902 (-28451)
HG319-J1: N(SingleHits)= 201902 (-28451)
HG319-J1: N(CleanHits) = 199187 (-31166)
◇ Amplicon Orientation:
```

```
   201902 HG319-J1-PF HG319-J1-PR
◇ Mis-Matches (top3):
  PrimerF   2361 ...............A
  PrimerF   3059 ...A............
  PrimerF 188341 ................
  PrimerR   1349 .................A...
  PrimerR   2140 .................AA..
  PrimerR 190230 .....................
- - -
HG319-J2: N(allPCRHits)= 354644 (-28983)
HG319-J2: N(SingleHits)= 354644 (-28983)
HG319-J2: N(CleanHits) = 351847 (-31780)
◇ Amplicon Orientation:
   354644 HG319-J2-PF HG319-J2-PR
◇ Mis-Matches (top3):
  PrimerF   3216 C...............
  PrimerF   5371 ...A............
  PrimerF 334556 ................
  PrimerR   2985 ..A..................
  PrimerR   3032 .................A...
  PrimerR 333548 .....................
- - -
HG319-J3: N(allPCRHits)= 131559 (-18053)
HG319-J3: N(SingleHits)= 131559 (-18053)
HG319-J3: N(CleanHits) = 130569 (-19043)
◇ Amplicon Orientation:
   131559 HG319-J3-PF HG319-J3-PR
◇ Mis-Matches (top3):
  PrimerF   1163 ............W...
  PrimerF   1348 C...............
  PrimerF 123601 ................
  PrimerR    754 ..................A..
  PrimerR    875 ..A..................
  PrimerR 124908 .....................
- - -
HG319-L1: N(allPCRHits)= 326953 (-19066)
HG319-L1: N(SingleHits)= 326953 (-19066)
HG319-L1: N(CleanHits) = 325806 (-20213)
◇ Amplicon Orientation:
   326953 HG319-L1-PF HG319-L1-PR
◇ Mis-Matches (top3):
  PrimerF   3208 C...............
  PrimerF   4847 ...A............
  PrimerF 309175 ................
  PrimerR   1951 ..................A..
  PrimerR   2526 .................A...
  PrimerR 310771 .....................
- - -
HG319-L2: N(allPCRHits)= 429362 (-28309)
HG319-L2: N(SingleHits)= 429362 (-28309)
HG319-L2: N(CleanHits) = 427728 (-29943)
◇ Amplicon Orientation:
   429362 HG319-L2-PF HG319-L2-PR
◇ Mis-Matches (top3):
```

```
  PrimerF   4213 C...............
  PrimerF   6452 ...A............
  PrimerF 406154 ................
  PrimerR   2891 ..A.................
  PrimerR   3599 .................A...
  PrimerR 407558 .....................
- - -
HG319-L3: N(allPCRHits)= 188133 (-13267)
HG319-L3: N(SingleHits)= 188133 (-13267)
HG319-L3: N(CleanHits) = 187391 (-14009)
◇ Amplicon Orientation:
   188133 HG319-L3-PF HG319-L3-PR
◇ Mis-Matches (top3):
  PrimerF   1364 ............W...
  PrimerF   1665 C...............
  PrimerF 178787 ................
  PrimerR   1103 ..................A..
  PrimerR   1136 ..A..................
  PrimerR 179339 .....................
- - -
HG319-O1: N(allPCRHits)= 253234 (-20410)
HG319-O1: N(SingleHits)= 253234 (-20410)
HG319-O1: N(CleanHits) = 252305 (-21339)
◇ Amplicon Orientation:
   253234 HG319-O1-PF HG319-O1-PR
◇ Mis-Matches (top3):
  PrimerF   2460 C...............
  PrimerF   3671 ...A............
  PrimerF 239353 ................
  PrimerR   1328 ..A..................
  PrimerR   1646 .................A...
  PrimerR 241607 .....................
- - -
HG319-O2: N(allPCRHits)= 130658 (-23824)
HG319-O2: N(SingleHits)= 130658 (-23824)
HG319-O2: N(CleanHits) = 129015 (-25467)
◇ Amplicon Orientation:
   130658 HG319-O2-PF HG319-O2-PR
◇ Mis-Matches (top3):
  PrimerF   1204 ............W...
  PrimerF   1241 ...............A
  PrimerF 122806 ................
  PrimerR    727 ..................A..
  PrimerR   1056 .................AA..
  PrimerR 123582 .....................
- - -
HG319-O3: N(allPCRHits)= 126327 (-10794)
HG319-O3: N(SingleHits)= 126327 (-10794)
HG319-O3: N(CleanHits) = 125493 (-11628)
◇ Amplicon Orientation:
   126327 HG319-O3-PF HG319-O3-PR
◇ Mis-Matches (top3):
  PrimerF    918 ............W...
  PrimerF   1115 C...............
```

```
  PrimerF 119552 ...............
  PrimerR    673 ..A.................
  PrimerR    731 .................A..
  PrimerR 120261 ....................
– – –
HG319-Q2: N(allPCRHits)= 148321 (-11152)
HG319-Q2: N(SingleHits)= 148321 (-11152)
HG319-Q2: N(CleanHits) = 147754 (-11719)
◇ Amplicon Orientation:
   148321 HG319-Q2-PF HG319-Q2-PR
◇ Mis-Matches (top3):
  PrimerF   1410 ...........W...
  PrimerF   1435 C..............
  PrimerF 139810 ...............
  PrimerR    891 ..A.................
  PrimerR    902 .................A..
  PrimerR 141261 ....................
– – –
HG319-Q3: N(allPCRHits)= 69451 (-11892)
HG319-Q3: N(SingleHits)= 69451 (-11892)
HG319-Q3: N(CleanHits) = 69156 (-12187)
◇ Amplicon Orientation:
    69451 HG319-Q3-PF HG319-Q3-PR
◇ Mis-Matches (top3):
  PrimerF    643 ...........W...
  PrimerF    663 C..............
  PrimerF  65468 ...............
  PrimerR    382 ..A.................
  PrimerR    470 .................A..
  PrimerR  65967 ....................
– – –
HG319-R1: N(allPCRHits)= 191052 (-39374)
HG319-R1: N(SingleHits)= 191052 (-39374)
HG319-R1: N(CleanHits) = 178773 (-51653)
◇ Amplicon Orientation:
   191052 HG319-R1-PF HG319-R1-PR
◇ Mis-Matches (top3):
  PrimerF   2703 ...A...........
  PrimerF  11377 ..............A
  PrimerF 169821 ...............
  PrimerR   1189 ................A...
  PrimerR  10466 ................AA..
  PrimerR 171198 ....................
– – –
HG319-R2: N(allPCRHits)= 183572 (-9949)
HG319-R2: N(SingleHits)= 183572 (-9949)
HG319-R2: N(CleanHits) = 182983 (-10538)
◇ Amplicon Orientation:
   183572 HG319-R2-PF HG319-R2-PR
◇ Mis-Matches (top3):
  PrimerF   1423 ...........W...
  PrimerF   1728 C..............
  PrimerF 173896 ...............
  PrimerR   1094 ..A.................
```

```
    PrimerR   1154 ..................A..
    PrimerR 174785 .....................
  - - -
  HG319-R3: N(allPCRHits)= 30800 (-17592)
  HG319-R3: N(SingleHits)= 30800 (-17592)
  HG319-R3: N(CleanHits) = 29191 (-19201)
  ◇ Amplicon Orientation:
      30800 HG319-R3-PF HG319-R3-PR
  ◇ Mis-Matches (top3):
    PrimerF    290 ............W...
    PrimerF   1318 ...............A
    PrimerF  27686 ................
    PrimerR    201 .................A...
    PrimerR   1143 .................AA..
    PrimerR  27666 .....................
  - - -
  HGnegA: N(allPCRHits)= 9239 (-463)
  HGnegA: N(SingleHits)= 9239 (-463)
  HGnegA: N(CleanHits) = 9202 (-500)
  ◇ Amplicon Orientation:
       9239 HGnegA-PF HGnegA-PR
  ◇ Mis-Matches (top3):
    PrimerF    103 ...A............
    PrimerF    154 ..........G.....
    PrimerF   8569 ................
    PrimerR     58 ..A..................
    PrimerR    124 ...................T.
    PrimerR   8698 .....................
  - - -
....................................................................
............
END_Workflow_StepC: 18:17:54 07/01/2021
====================================================================
============
Step D | Size Range Selection and (Quality) Filtering
--------------------------------------------------------------------
------------
PRINSEQ-lite 0.20.4
Size Range    : 300-500
GC Range      : 30-70
Min Q Mean    : 20
Number of Ns  : 0
Low Complexity: dust / 30
--------------------------------------------------------------------
------------
START_Worklfow_StepD: 18:18:19 07/01/2021
....................................................................
............
 Start_QF: 18:18:19 07/01/2021
  ➸ HG015-B1: N(filtered)= 223072 (-3021)
  ➸ HG015-B3: N(filtered)= 65747 (-1311)
  ➸ HG015-D1: N(filtered)= 179665 (-2554)
  ➸ HG015-D2: N(filtered)= 272280 (-3784)
  ➸ HG015-D3: N(filtered)= 124341 (-1767)
```

➸ HG015-I1: N(filtered)= 51695 (-1082)
➸ HG015-I2: N(filtered)= 168843 (-2399)
➸ HG015-I3: N(filtered)= 135936 (-1897)
➸ HG015-J1: N(filtered)= 83547 (-1300)
➸ HG015-J2: N(filtered)= 226656 (-3431)
➸ HG015-J3: N(filtered)= 232158 (-3141)
➸ HG015-L1: N(filtered)= 374896 (-5061)
➸ HG015-L2: N(filtered)= 229995 (-3342)
➸ HG015-L3: N(filtered)= 157727 (-2157)
➸ HG015-O1: N(filtered)= 201557 (-2820)
➸ HG015-O2: N(filtered)= 80641 (-1360)
➸ HG015-O3: N(filtered)= 192743 (-2676)
➸ HG015-Q1: N(filtered)= 226025 (-3197)
➸ HG015-Q2: N(filtered)= 134900 (-1840)
➸ HG015-Q3: N(filtered)= 282626 (-4123)
➸ HG015-R1: N(filtered)= 349176 (-4854)
➸ HG015-R2: N(filtered)= 12152 (-281)
➸ HG015-R3: N(filtered)= 164048 (-2364)
➸ HG033-B1: N(filtered)= 254525 (-3638)
➸ HG033-B2: N(filtered)= 303362 (-4420)
➸ HG033-B3: N(filtered)= 137634 (-1852)
➸ HG033-D1: N(filtered)= 189835 (-2588)
➸ HG033-D2: N(filtered)= 273954 (-3848)
➸ HG033-D3: N(filtered)= 188433 (-3057)
➸ HG033-I1: N(filtered)= 113931 (-2695)
➸ HG033-I2: N(filtered)= 211535 (-3014)
➸ HG033-I3: N(filtered)= 551909 (-8552)
➸ HG033-J1: N(filtered)= 341447 (-4368)
➸ HG033-J2: N(filtered)= 162932 (-2458)
➸ HG033-J3: N(filtered)= 168447 (-2412)
➸ HG033-L1: N(filtered)= 237936 (-3288)
➸ HG033-L2: N(filtered)= 65554 (-1809)
➸ HG033-L3: N(filtered)= 1292111 (-18685)
➸ HG033-O1: N(filtered)= 350095 (-4623)
➸ HG033-O2: N(filtered)= 273861 (-3810)
➸ HG033-O3: N(filtered)= 141843 (-2044)
➸ HG033-Q1: N(filtered)= 163586 (-2699)
➸ HG033-Q2: N(filtered)= 146680 (-2160)
➸ HG033-Q3: N(filtered)= 139016 (-1849)
➸ HG033-R1: N(filtered)= 201140 (-3616)
➸ HG033-R2: N(filtered)= 117072 (-1388)
➸ HG033-R3: N(filtered)= 85113 (-1918)
➸ HG059-B1: N(filtered)= 274511 (-3827)
➸ HG059-B2: N(filtered)= 264655 (-3563)
➸ HG059-B3: N(filtered)= 156209 (-2141)
➸ HG059-D1: N(filtered)= 330745 (-4721)
➸ HG059-D2: N(filtered)= 384310 (-5139)
➸ HG059-D3: N(filtered)= 314349 (-4465)
➸ HG059-I1: N(filtered)= 535034 (-7385)
➸ HG059-I2: N(filtered)= 156359 (-2087)
➸ HG059-I3: N(filtered)= 187475 (-2499)
➸ HG059-J1: N(filtered)= 243570 (-3397)
➸ HG059-J2: N(filtered)= 232006 (-3377)
➸ HG059-J3: N(filtered)= 905314 (-12791)

➼ HG059-L1: N(filtered)= 158979 (-2133)
➼ HG059-L2: N(filtered)= 257282 (-4013)
➼ HG059-L3: N(filtered)= 209196 (-2938)
➼ HG059-O1: N(filtered)= 351767 (-4837)
➼ HG059-O2: N(filtered)= 289004 (-4047)
➼ HG059-O3: N(filtered)= 125992 (-1782)
➼ HG059-Q1: N(filtered)= 556611 (-7858)
➼ HG059-Q2: N(filtered)= 121936 (-1798)
➼ HG059-Q3: N(filtered)= 159711 (-2161)
➼ HG059-R1: N(filtered)= 314061 (-4224)
➼ HG059-R2: N(filtered)= 188938 (-2552)
➼ HG059-R3: N(filtered)= 234844 (-3247)
➼ HG082-B1: N(filtered)= 216367 (-3081)
➼ HG082-B2: N(filtered)= 380522 (-5271)
➼ HG082-B3: N(filtered)= 167199 (-2274)
➼ HG082-D1: N(filtered)= 175492 (-2567)
➼ HG082-D2: N(filtered)= 220334 (-3058)
➼ HG082-D3: N(filtered)= 168244 (-2356)
➼ HG082-I1: N(filtered)= 88599 (-2804)
➼ HG082-I2: N(filtered)= 242383 (-3376)
➼ HG082-I3: N(filtered)= 265674 (-3631)
➼ HG082-J1: N(filtered)= 238461 (-3274)
➼ HG082-J2: N(filtered)= 210063 (-2985)
➼ HG082-J3: N(filtered)= 143239 (-2009)
➼ HG082-L1: N(filtered)= 256457 (-3631)
➼ HG082-L2: N(filtered)= 555807 (-8614)
➼ HG082-L3: N(filtered)= 122127 (-1601)
➼ HG082-O1: N(filtered)= 168773 (-2462)
➼ HG082-O2: N(filtered)= 27749 (-1698)
➼ HG082-O3: N(filtered)= 261635 (-3889)
➼ HG082-Q1: N(filtered)= 150217 (-2521)
➼ HG082-Q2: N(filtered)= 139499 (-2408)
➼ HG082-Q3: N(filtered)= 241582 (-3366)
➼ HG082-R1: N(filtered)= 303771 (-4096)
➼ HG082-R2: N(filtered)= 164545 (-2242)
➼ HG082-R3: N(filtered)= 330012 (-4773)
➼ HG091-B1: N(filtered)= 20710 (-337)
➼ HG091-B2: N(filtered)= 125455 (-2217)
➼ HG091-B3: N(filtered)= 264610 (-3815)
➼ HG091-D1: N(filtered)= 39486 (-619)
➼ HG091-D2: N(filtered)= 157077 (-2420)
➼ HG091-D3: N(filtered)= 33630 (-525)
➼ HG091-I1: N(filtered)= 229382 (-3233)
➼ HG091-I2: N(filtered)= 245233 (-3674)
➼ HG091-I3: N(filtered)= 136671 (-1994)
➼ HG091-J1: N(filtered)= 2833 (-162)
➼ HG091-J2: N(filtered)= 194345 (-2964)
➼ HG091-J3: N(filtered)= 69988 (-1337)
➼ HG091-L1: N(filtered)= 5395 (-179)
➼ HG091-L2: N(filtered)= 245180 (-3488)
➼ HG091-L3: N(filtered)= 104465 (-1650)
➼ HG091-O1: N(filtered)= 349515 (-4839)
➼ HG091-O2: N(filtered)= 74489 (-1350)
➼ HG091-O3: N(filtered)= 144119 (-2105)

- ➽ HG091-Q1: N(filtered)= 194978 (-3182)
- ➽ HG091-Q2: N(filtered)= 149261 (-2109)
- ➽ HG091-Q3: N(filtered)= 337850 (-4871)
- ➽ HG091-R1: N(filtered)= 52556 (-3567)
- ➽ HG091-R2: N(filtered)= 74769 (-2204)
- ➽ HG091-R3: N(filtered)= 169845 (-2592)
- ➽ HG141-B1: N(filtered)= 119720 (-1611)
- ➽ HG141-B2: N(filtered)= 231445 (-3243)
- ➽ HG141-B3: N(filtered)= 291514 (-4111)
- ➽ HG141-D1: N(filtered)= 46949 (-681)
- ➽ HG141-D2: N(filtered)= 349806 (-4843)
- ➽ HG141-D3: N(filtered)= 220940 (-2949)
- ➽ HG141-I1: N(filtered)= 115070 (-1599)
- ➽ HG141-I2: N(filtered)= 233157 (-3483)
- ➽ HG141-I3: N(filtered)= 151124 (-1962)
- ➽ HG141-J1: N(filtered)= 158947 (-2266)
- ➽ HG141-J2: N(filtered)= 286986 (-4073)
- ➽ HG141-J3: N(filtered)= 213532 (-3106)
- ➽ HG141-L1: N(filtered)= 223998 (-3200)
- ➽ HG141-L2: N(filtered)= 264165 (-3756)
- ➽ HG141-L3: N(filtered)= 142866 (-1976)
- ➽ HG141-O1: N(filtered)= 587268 (-7840)
- ➽ HG141-O2: N(filtered)= 221265 (-3002)
- ➽ HG141-O3: N(filtered)= 198946 (-2786)
- ➽ HG141-Q1: N(filtered)= 180836 (-2501)
- ➽ HG141-Q2: N(filtered)= 127553 (-1908)
- ➽ HG141-Q3: N(filtered)= 323913 (-4769)
- ➽ HG141-R1: N(filtered)= 238347 (-3402)
- ➽ HG141-R2: N(filtered)= 63620 (-958)
- ➽ HG141-R3: N(filtered)= 216814 (-3009)
- ➽ HG225-B1: N(filtered)= 57020 (-842)
- ➽ HG225-B2: N(filtered)= 279038 (-3838)
- ➽ HG225-B3: N(filtered)= 224167 (-3151)
- ➽ HG225-D1: N(filtered)= 51312 (-700)
- ➽ HG225-D2: N(filtered)= 319267 (-4607)
- ➽ HG225-D3: N(filtered)= 182376 (-2493)
- ➽ HG225-I1: N(filtered)= 55967 (-820)
- ➽ HG225-I2: N(filtered)= 175342 (-3042)
- ➽ HG225-I3: N(filtered)= 168519 (-2444)
- ➽ HG225-J1: N(filtered)= 20269 (-373)
- ➽ HG225-J2: N(filtered)= 284523 (-4458)
- ➽ HG225-J3: N(filtered)= 157550 (-2261)
- ➽ HG225-L1: N(filtered)= 311916 (-4341)
- ➽ HG225-L2: N(filtered)= 289552 (-3895)
- ➽ HG225-L3: N(filtered)= 141658 (-2033)
- ➽ HG225-O1: N(filtered)= 257265 (-3715)
- ➽ HG225-O2: N(filtered)= 25670 (-684)
- ➽ HG225-O3: N(filtered)= 136257 (-1929)
- ➽ HG225-Q1: N(filtered)= 202474 (-2832)
- ➽ HG225-Q2: N(filtered)= 101484 (-1595)
- ➽ HG225-Q3: N(filtered)= 77138 (-1251)
- ➽ HG225-R1: N(filtered)= 179636 (-2420)
- ➽ HG225-R2: N(filtered)= 253957 (-3499)
- ➽ HG225-R3: N(filtered)= 19012 (-405)

```
  ➸ HG319-B1: N(filtered)= 114842 (-1563)
  ➸ HG319-B2: N(filtered)= 182741 (-2592)
  ➸ HG319-B3: N(filtered)= 293667 (-3841)
  ➸ HG319-D1: N(filtered)= 84967 (-1157)
  ➸ HG319-D2: N(filtered)= 268826 (-4144)
  ➸ HG319-D3: N(filtered)= 200648 (-2872)
  ➸ HG319-I1: N(filtered)= 276698 (-3798)
  ➸ HG319-I2: N(filtered)= 198742 (-2690)
  ➸ HG319-I3: N(filtered)= 232261 (-3266)
  ➸ HG319-J1: N(filtered)= 195971 (-3216)
  ➸ HG319-J2: N(filtered)= 346700 (-5147)
  ➸ HG319-J3: N(filtered)= 128741 (-1828)
  ➸ HG319-L1: N(filtered)= 321294 (-4512)
  ➸ HG319-L2: N(filtered)= 421857 (-5871)
  ➸ HG319-L3: N(filtered)= 184407 (-2984)
  ➸ HG319-O1: N(filtered)= 248784 (-3521)
  ➸ HG319-O2: N(filtered)= 126890 (-2125)
  ➸ HG319-O3: N(filtered)= 123606 (-1887)
  ➸ HG319-Q2: N(filtered)= 145457 (-2297)
  ➸ HG319-Q3: N(filtered)= 68088 (-1068)
  ➸ HG319-R1: N(filtered)= 176075 (-2698)
  ➸ HG319-R2: N(filtered)= 180490 (-2493)
  ➸ HG319-R3: N(filtered)= 28550 (-641)
  ➸ HGnegA: N(filtered)= 9069 (-133)
 End_QF: 23:05:23 07/01/2021
....................................................................
............
 Start_CombineSequences: 23:05:23 07/01/2021
  ➸ N(all): 39635349 (== 39635349) ✔pass
 End_CombineSequences: 23:10:54 07/01/2021
....................................................................
............
 Start_ReadReport: 23:10:54 07/01/2021
  Total Number of Amplicons: 39635349 ✔pass
 End_ReadReport: 23:32:21 07/01/2021
....................................................................
............
END_Workflow_StepD: 23:32:21 07/01/2021
====================================================================
============
Data Loss Statistic for steps B-D
  Summary:
    Average total data loss       : 18.136%
    Average data loss Raw-Filter  :  0.004%
    Average data loss Filter-Merge:  7.808%
    Average data loss Merge-Primer:  9.904%
    Average data loss Primer-Clean:  1.437%
  Per Sample:
    Sample;Raw;B1-Filter;B2-Merged;C-Primer;D-Clean;MeanLength
    HG015-B1;252219;252217;240977;226093;223072;428.717
    HG015-B3;198950;198881;139361;67058;65747;425.115
    HG015-D1;221531;221524;204950;182219;179665;428.271
    HG015-D2;307848;307840;294777;276064;272280;421.478
    HG015-D3;174077;174075;150899;126108;124341;428.823
```

HG015-I1;120265;120254;99848;52777;51695;423.564
HG015-I2;190266;190259;182941;171242;168843;428.152
HG015-I3;152885;152872;145382;137833;135936;428.093
HG015-J1;113678;113674;102813;84847;83547;428.77
HG015-J2;274016;274010;255837;230087;226656;428.741
HG015-J3;273726;273721;249792;235299;232158;406.334
HG015-L1;425482;425471;409392;379957;374896;428.254
HG015-L2;288808;288798;266521;233337;229995;428.644
HG015-L3;190511;190507;172388;159884;157727;415.553
HG015-O1;240195;240184;226687;204377;201557;428.576
HG015-O2;180647;180641;137466;82001;80641;414.448
HG015-O3;232928;232907;213442;195419;192743;426.047
HG015-Q1;249194;249188;242059;229222;226025;428.884
HG015-Q2;156770;156765;147031;136740;134900;428.553
HG015-Q3;322564;322559;301223;286749;282626;414.78
HG015-R1;388444;388432;376108;354030;349176;427.344
HG015-R2;47819;47815;26769;12433;12152;416.762
HG015-R3;188644;188638;178194;166412;164048;428.09
HG033-B1;296631;296622;279554;258163;254525;418.182
HG033-B2;343225;343220;330747;307782;303362;428.97
HG033-B3;160754;160750;149653;139486;137634;419.28
HG033-D1;212823;212814;205847;192423;189835;428.791
HG033-D2;307079;307077;297394;277802;273954;428.948
HG033-D3;279073;279065;235771;191490;188433;411.844
HG033-I1;258136;258128;201374;116626;113931;422.218
HG033-I2;239668;239659;231141;214549;211535;428.957
HG033-I3;765004;764976;665944;560461;551909;427.181
HG033-J1;375326;375315;365038;345815;341447;428.347
HG033-J2;214918;214912;194658;165390;162932;427.003
HG033-J3;205545;205540;187287;170859;168447;428.479
HG033-L1;261063;261055;253954;241224;237936;427.851
HG033-L2;129673;129671;105089;67363;65554;427.756
HG033-L3;1545420;1545390;1424209;1310796;1292111;425.351
HG033-O1;407874;407862;383562;354718;350095;417.061
HG033-O2;312173;312165;294874;277671;273861;413.986
HG033-O3;168128;168126;156360;143887;141843;423.827
HG033-Q1;235327;235322;207323;166285;163586;428.916
HG033-Q2;187428;187425;166797;148840;146680;412.445
HG033-Q3;170533;170531;154685;140865;139016;415.992
HG033-R1;313021;313004;269946;204756;201140;428.819
HG033-R2;181957;181946;129788;118460;117072;428.981
HG033-R3;175437;175434;130757;87031;85113;428.008
HG059-B1;312125;312116;299558;278338;274511;428.576
HG059-B2;293478;293473;285424;268218;264655;428.354
HG059-B3;178002;178001;166789;158350;156209;411.861
HG059-D1;381378;381369;365561;335466;330745;427.468
HG059-D2;427506;427496;414002;389449;384310;427.371
HG059-D3;362246;362243;340879;318814;314349;426.457
HG059-I1;583626;583611;570580;542419;535034;428.1
HG059-I2;179489;179484;171100;158446;156359;425.965
HG059-I3;211438;211435;200160;189974;187475;428.565
HG059-J1;276314;276309;264832;246967;243570;423.912
HG059-J2;309576;309567;279293;235383;232006;428.241
HG059-J3;1035315;1035294;979581;918105;905314;428.299

HG059-L1;175586;175580;169866;161112;158979;419.024
HG059-L2;315013;315005;292870;261295;257282;428.374
HG059-L3;245677;245625;226404;212134;209196;416.119
HG059-O1;394282;394279;380858;356604;351767;428.006
HG059-O2;324092;324085;308175;293051;289004;409.084
HG059-O3;139656;139654;133989;127774;125992;428.451
HG059-Q1;613227;613205;594120;564469;556611;423.508
HG059-Q2;148924;148920;136361;123734;121936;423.3
HG059-Q3;177415;177412;169720;161872;159711;428.508
HG059-R1;345375;345365;336322;318285;314061;428.288
HG059-R2;211006;211002;202030;191490;188938;428.825
HG059-R3;268162;268159;251615;238091;234844;416.829
HG082-B1;240030;240026;233156;219448;216367;428.242
HG082-B2;437367;437360;407738;385793;380522;412.532
HG082-B3;189900;189896;179790;169473;167199;426.367
HG082-D1;206700;206698;196308;178059;175492;426.589
HG082-D2;249289;249285;235969;223392;220334;424.414
HG082-D3;191619;191613;181678;170600;168244;426.958
HG082-I1;152334;152331;132160;91403;88599;424.136
HG082-I2;265048;265045;258250;245759;242383;427.441
HG082-I3;303794;303790;285979;269305;265674;421.638
HG082-J1;261048;261045;254121;241735;238461;424.444
HG082-J2;237305;237293;224331;213048;210063;409.113
HG082-J3;161566;161564;154087;145248;143239;424.708
HG082-L1;280484;280477;273920;260088;256457;428.721
HG082-L2;703778;703755;649099;564421;555807;426.937
HG082-L3;140479;140477;132337;123728;122127;428.058
HG082-O1;208389;208384;193907;171235;168773;428.845
HG082-O2;175384;175377;113897;29447;27749;421.041
HG082-O3;308668;308661;285431;265524;261635;425.731
HG082-Q1;219119;219109;196236;152738;150217;426.838
HG082-Q2;161804;161784;151567;141907;139499;420.633
HG082-Q3;282246;282241;264501;244948;241582;428.318
HG082-R1;336142;336139;326687;307867;303771;428.467
HG082-R2;185459;185457;176402;166787;164545;426.823
HG082-R3;414351;414345;377585;334785;330012;428.799
HG091-B1;36492;36476;25786;21047;20710;422.325
HG091-B2;174741;174737;151554;127672;125455;425.144
HG091-B3;305849;305832;286803;268425;264610;424.7
HG091-D1;73392;73347;47676;40105;39486;408.077
HG091-D2;212247;212235;187918;159497;157077;428.539
HG091-D3;96930;96928;68643;34155;33630;428.527
HG091-I1;262690;262684;249744;232615;229382;429.817
HG091-I2;306020;306011;284437;248907;245233;427.193
HG091-I3;166514;166510;149848;138665;136671;410.212
HG091-J1;17769;17763;7902;2995;2833;421.571
HG091-J2;269290;269285;238298;197309;194345;412.251
HG091-J3;162562;162559;116176;71325;69988;427.066
HG091-L1;30531;30504;13088;5574;5395;426.463
HG091-L2;289629;289620;273265;248668;245180;414.014
HG091-L3;134830;134829;119610;106115;104465;428.994
HG091-O1;417379;417370;392016;354354;349515;414.473
HG091-O2;116789;116783;95205;75839;74489;421.627
HG091-O3;182873;182870;163001;146224;144119;413.882

HG091-Q1;259634;259622;238257;198160;194978;428.832
HG091-Q2;172329;172329;161089;151370;149261;429.457
HG091-Q3;432498;432484;379087;342721;337850;409.654
HG091-R1;214759;214747;153917;56123;52556;427.489
HG091-R2;172215;172206;131980;76973;74769;426.976
HG091-R3;244829;244822;205074;172437;169845;410.695
HG141-B1;154562;154521;131003;121331;119720;428.397
HG141-B2;263817;263815;247591;234688;231445;420.376
HG141-B3;326048;326045;310455;295625;291514;424.831
HG141-D1;56606;56601;51044;47630;46949;422.399
HG141-D2;393462;393449;373771;354649;349806;428.32
HG141-D3;251107;251107;235294;223889;220940;415.451
HG141-I1;134157;134140;124181;116669;115070;425.141
HG141-I2;261793;261786;253459;236640;233157;428.312
HG141-I3;173771;173765;163563;153086;151124;427.856
HG141-J1;184852;184838;173717;161213;158947;428.4
HG141-J2;358964;358961;333157;291059;286986;426.063
HG141-J3;289207;289182;251155;216638;213532;411.069
HG141-L1;254056;254047;241595;227198;223998;425.169
HG141-L2;296483;296478;284689;267921;264165;421.411
HG141-L3;162736;162736;154380;144842;142866;427.967
HG141-O1;652857;652841;625137;595108;587268;414.425
HG141-O2;254317;254313;240269;224267;221265;427.965
HG141-O3;224308;224305;213670;201732;198946;428.239
HG141-Q1;206392;206390;197970;183337;180836;425.631
HG141-Q2;145671;145669;137016;129461;127553;427.269
HG141-Q3;381514;381505;356604;328682;323913;425.663
HG141-R1;275734;275726;263587;241749;238347;427.473
HG141-R2;114632;114628;90300;64578;63620;420.834
HG141-R3;250233;250212;233044;219823;216814;417.036
HG225-B1;91036;91002;65077;57862;57020;427.715
HG225-B2;325072;325066;304863;282876;279038;424.223
HG225-B3;258916;258914;239744;227318;224167;410.17
HG225-D1;68591;68573;58065;52012;51312;428.316
HG225-D2;370072;370042;345291;323874;319267;423.128
HG225-D3;208823;208821;193719;184869;182376;407.319
HG225-I1;80746;80742;66187;56787;55967;408.918
HG225-I2;254700;254693;223259;178384;175342;416.967
HG225-I3;196797;196791;182835;170963;168519;425.222
HG225-J1;50051;50025;30091;20642;20269;423.916
HG225-J2;396791;396779;352287;288981;284523;428.097
HG225-J3;222747;222743;188923;159811;157550;413.975
HG225-L1;348550;348537;335020;316257;311916;428.661
HG225-L2;328680;328671;311631;293447;289552;406.875
HG225-L3;172497;172491;157772;143691;141658;427.41
HG225-O1;289511;289499;278661;260980;257265;428.327
HG225-O2;105009;104994;69679;26354;25670;420.113
HG225-O3;159977;159952;147940;138186;136257;423.365
HG225-Q1;226639;226636;218365;205306;202474;424.22
HG225-Q2;188127;188118;143106;103079;101484;414.148
HG225-Q3;114555;114550;94593;78389;77138;406.806
HG225-R1;201435;201433;192002;182056;179636;409.834
HG225-R2;308518;308514;278075;257456;253957;415.403
HG225-R3;41326;41318;27932;19417;19012;424.253

```
    HG319-B1;138783;138771;125252;116405;114842;412.398
    HG319-B2;217644;217641;203163;185333;182741;428.462
    HG319-B3;332209;332202;313818;297508;293667;423.714
    HG319-D1;102797;102774;92700;86124;84967;426.964
    HG319-D2;331926;331918;306162;272970;268826;427.359
    HG319-D3;224990;224961;214332;203520;200648;428.086
    HG319-I1;308835;308834;299144;280496;276698;428.781
    HG319-I2;246946;246931;229255;201432;198742;418.65
    HG319-I3;262152;262147;249122;235527;232261;428.744
    HG319-J1;247684;247677;230353;199187;195971;426.329
    HG319-J2;398868;398859;383627;351847;346700;428.88
    HG319-J3;164020;164013;149612;130569;128741;428.825
    HG319-L1;357278;357268;346019;325806;321294;428.422
    HG319-L2;473572;473565;457671;427728;421857;428.131
    HG319-L3;211980;211978;201400;187391;184407;428.933
    HG319-O1;285900;285886;273644;252305;248784;428.903
    HG319-O2;173321;173316;154482;129015;126890;428.716
    HG319-O3;145925;145922;137121;125493;123606;427.846
    HG319-Q2;169823;169820;159473;147754;145457;427.561
    HG319-Q3;92853;92848;81343;69156;68088;427.168
    HG319-R1;258565;258557;230426;178773;176075;426.216
    HG319-R2;204158;204155;193521;182983;180490;426.654
    HG319-R3;64989;64983;48392;29191;28550;424.13
    HGnegA;12502;12497;9702;9202;9069;418.633
====================================================================
============
Step E1 | UPARSE & UNOISE + 99%-, 98%-, and 97%-Identity Clustering
--------------------------------------------------------------------
------------
UPARSE : usearch v11.0.667_i86linux64
Min Abundance Size: 2
....................................................................
............
UNOISE3: usearch v11.0.667_i86linux64
Min Abundance Size: 10
--------------------------------------------------------------------
------------
START_E1: 10:10:45 08/01/2021
--------------------------------------------------------------------
------------
 ▶ Deduplicate Amplicons
....................................................................
............
  De-replicate amplicons to obtain unique amplicons.
  Determine error rates of amplicon reads > *error.report
  ⇨ Number of Unique Amplicons: 7526015 (19%)
--------------------------------------------------------------------
------------
▶ UPARSE - Cluster OTU (97%)
....................................................................
............
  Clusters OTU at 97% using the UPARSE-OTU algorithm.
  ⇨ Number of OTUs: 772
--------------------------------------------------------------------
```

```
------------
▶ UNOISE3 - Amplicon Sequence Variants
.....................................................................
............
  Uses the UNOISE algorithm to perform denoising (error-correction)
of amplicon sequence variants (zero OTUs).
  ⇨ Number of ZOTUs: 997
---------------------------------------------------------------------
------------
▶ Additional Clustering
.....................................................................
............
  Clusters ZOTUs at different identity levels (i.e. 97%,98% and
99%).
  • Sort ZOTUs by lengt
  • Cluster ZOTU with 99% id ✔
  • Cluster ZOTU with 98% id ✔
  • Cluster ZOTU with 98% id ✔
  ⇨ Number of ZOTUs 99%: 708
  ⇨ Number of ZOTUs 98%: 574
  ⇨ Number of ZOTUs 97%: 443
---------------------------------------------------------------------
------------
END_E1: 10:35:56 08/01/2021
=====================================================================
============
Step E2 - Back-Mapping Amplicons to OTU
---------------------------------------------------------------------
------------
USEARCHusearch v11.0.667_i86linux64
Identity threshold for mapping: 0.97
---------------------------------------------------------------------
------------
START_E2: 11:16:03 08/01/2021
---------------------------------------------------------------------
------------
Counts per OTU:
13373246 OTU1
8752816 OTU2
4949096 OTU3
2961951 OTU9
1148437 OTU4
 990692 OTU5
 856399 OTU6
 839760 OTU8
 800969 OTU7
 667374 OTU41
 576479 OTU12
 575662 OTU22
 370736 OTU11
 360410 OTU346
 210963 OTU10
 131706 OTU59
 125103 OTU13
```

114870	OTU16
107432	OTU15
100127	OTU69
96700	OTU14
66180	OTU17
53184	OTU104
47466	OTU74
40715	OTU42
30033	OTU18
19710	OTU396
11976	OTU19
6577	OTU20
6571	OTU21
6420	OTU24
6020	OTU324
5716	OTU26
4615	OTU25
4466	OTU23
4198	OTU28
4090	OTU38
3753	OTU235
3537	OTU27
3377	OTU34
3015	OTU30
2914	OTU129
2667	OTU33
2382	OTU37
2153	OTU29
2132	OTU455
2131	OTU157
2064	OTU46
2037	OTU35
1937	OTU31
1884	OTU32
1876	OTU45
1635	OTU64
1426	OTU47
1390	OTU124
1370	OTU39
1227	OTU560
1200	OTU40
1129	OTU479
1108	OTU44
1104	OTU135
1100	OTU36
1075	OTU291
1008	OTU76
965	OTU546
957	OTU79
930	OTU91
913	OTU43
875	OTU116
869	OTU127
826	OTU306

821 OTU622
817 OTU160
810 OTU51
805 OTU50
799 OTU78
795 OTU48
791 OTU106
783 OTU118
758 OTU133
754 OTU53
750 OTU57
718 OTU52
652 OTU56
650 OTU49
626 OTU66
625 OTU712
588 OTU55
584 OTU582
575 OTU54
566 OTU767
556 OTU108
548 OTU168
521 OTU109
520 OTU72
504 OTU105
476 OTU63
471 OTU441
465 OTU61
457 OTU70
452 OTU58
447 OTU125
443 OTU83
438 OTU62
437 OTU77
428 OTU65
427 OTU60
393 OTU68
391 OTU71
391 OTU93
383 OTU67
380 OTU94
376 OTU180
355 OTU431
340 OTU73
318 OTU80
318 OTU81
317 OTU379
315 OTU82
314 OTU113
310 OTU144
310 OTU89
308 OTU121
307 OTU103
306 OTU75

288 OTU682
285 OTU90
279 OTU718
270 OTU102
270 OTU87
269 OTU96
264 OTU126
262 OTU84
262 OTU88
257 OTU422
256 OTU86
255 OTU85
245 OTU130
242 OTU183
242 OTU468
228 OTU92
216 OTU97
214 OTU107
213 OTU101
207 OTU289
201 OTU117
201 OTU493
201 OTU98
198 OTU232
194 OTU750
194 OTU99
191 OTU132
188 OTU123
187 OTU114
184 OTU128
182 OTU131
182 OTU246
182 OTU724
176 OTU700
175 OTU95
171 OTU110
169 OTU138
168 OTU112
160 OTU166
157 OTU100
157 OTU111
151 OTU751
149 OTU122
148 OTU149
146 OTU147
145 OTU707
144 OTU115
144 OTU161
141 OTU120
141 OTU151
140 OTU141
139 OTU254
137 OTU276
136 OTU206

```
133 OTU119
133 OTU368
132 OTU709
131 OTU139
130 OTU142
129 OTU146
129 OTU163
129 OTU165
129 OTU167
128 OTU198
127 OTU502
126 OTU172
122 OTU134
122 OTU332
121 OTU676
117 OTU137
117 OTU154
117 OTU339
116 OTU191
116 OTU772
115 OTU761
111 OTU181
106 OTU185
103 OTU145
102 OTU248
100 OTU176
 99 OTU153
 99 OTU207
 99 OTU519
 99 OTU633
 97 OTU136
 97 OTU156
 97 OTU158
 97 OTU171
 95 OTU164
 93 OTU219
 91 OTU184
 91 OTU227
 90 OTU192
 90 OTU297
 88 OTU143
 88 OTU308
 87 OTU318
 85 OTU152
 84 OTU140
 84 OTU148
 84 OTU162
 84 OTU234
 82 OTU237
 82 OTU265
 82 OTU409
 81 OTU261
 81 OTU458
 80 OTU159
```

80 OTU228
80 OTU270
80 OTU301
79 OTU169
76 OTU150
76 OTU309
75 OTU195
74 OTU174
74 OTU210
73 OTU155
73 OTU649
72 OTU218
72 OTU335
70 OTU173
68 OTU213
68 OTU216
68 OTU244
68 OTU517
67 OTU189
66 OTU203
66 OTU354
65 OTU175
65 OTU187
65 OTU252
65 OTU500
64 OTU201
64 OTU277
64 OTU471
63 OTU186
63 OTU317
62 OTU170
62 OTU177
62 OTU190
61 OTU224
61 OTU383
60 OTU188
60 OTU412
60 OTU444
59 OTU230
58 OTU410
58 OTU637
57 OTU300
56 OTU197
56 OTU204
56 OTU236
56 OTU290
56 OTU376
55 OTU194
55 OTU209
55 OTU223
55 OTU225
55 OTU233
55 OTU748
54 OTU178

54 OTU196
54 OTU222
54 OTU229
54 OTU255
54 OTU304
54 OTU352
53 OTU193
53 OTU212
53 OTU296
52 OTU466
52 OTU713
51 OTU179
51 OTU217
51 OTU266
50 OTU182
50 OTU208
50 OTU215
50 OTU231
49 OTU202
49 OTU221
49 OTU258
49 OTU720
48 OTU241
48 OTU274
48 OTU320
47 OTU247
46 OTU243
46 OTU283
46 OTU285
46 OTU316
46 OTU456
46 OTU487
46 OTU659
45 OTU200
45 OTU286
45 OTU435
45 OTU626
45 OTU627
44 OTU211
44 OTU238
44 OTU245
43 OTU220
43 OTU345
43 OTU363
43 OTU675
42 OTU199
42 OTU205
42 OTU282
42 OTU305
41 OTU262
41 OTU279
40 OTU257
40 OTU293
40 OTU351

40 OTU430
39 OTU226
39 OTU543
38 OTU249
38 OTU260
38 OTU264
38 OTU292
38 OTU330
38 OTU710
37 OTU239
37 OTU321
37 OTU464
36 OTU214
36 OTU267
36 OTU271
36 OTU373
35 OTU250
34 OTU259
34 OTU263
34 OTU278
34 OTU327
34 OTU384
34 OTU571
33 OTU256
33 OTU269
33 OTU287
33 OTU424
33 OTU514
33 OTU574
32 OTU275
32 OTU311
32 OTU336
32 OTU448
32 OTU554
31 OTU253
31 OTU268
31 OTU272
31 OTU273
31 OTU302
31 OTU307
31 OTU314
31 OTU393
30 OTU242
30 OTU624
29 OTU251
29 OTU323
28 OTU295
28 OTU415
28 OTU494
28 OTU522
27 OTU240
27 OTU294
27 OTU392
27 OTU404

26 OTU284
26 OTU319
26 OTU328
26 OTU344
26 OTU386
26 OTU389
26 OTU526
25 OTU281
25 OTU427
25 OTU538
24 OTU288
24 OTU329
24 OTU334
24 OTU337
24 OTU353
24 OTU447
24 OTU461
23 OTU299
23 OTU303
23 OTU331
23 OTU348
23 OTU397
22 OTU326
22 OTU349
22 OTU387
22 OTU391
22 OTU402
22 OTU403
22 OTU413
22 OTU420
21 OTU298
21 OTU312
21 OTU322
21 OTU338
21 OTU356
21 OTU533
21 OTU638
21 OTU654
21 OTU731
20 OTU364
20 OTU375
20 OTU395
20 OTU414
20 OTU509
20 OTU722
19 OTU280
19 OTU343
19 OTU362
19 OTU366
19 OTU371
19 OTU400
19 OTU405
19 OTU411
19 OTU598

19 OTU617
18 OTU313
18 OTU340
18 OTU357
18 OTU358
18 OTU367
18 OTU385
18 OTU426
18 OTU506
17 OTU315
17 OTU325
17 OTU406
17 OTU417
17 OTU470
17 OTU661
17 OTU672
17 OTU771
16 OTU310
16 OTU347
16 OTU370
16 OTU390
16 OTU432
16 OTU433
16 OTU465
16 OTU501
16 OTU503
16 OTU625
16 OTU706
16 OTU737
15 OTU333
15 OTU361
15 OTU365
15 OTU401
15 OTU428
15 OTU439
15 OTU453
15 OTU508
15 OTU577
15 OTU602
15 OTU621
15 OTU645
14 OTU341
14 OTU342
14 OTU350
14 OTU359
14 OTU380
14 OTU381
14 OTU394
14 OTU407
14 OTU467
14 OTU498
14 OTU537
14 OTU562
14 OTU584

13 OTU360
13 OTU374
13 OTU399
13 OTU442
13 OTU476
13 OTU486
13 OTU530
13 OTU549
13 OTU590
13 OTU667
12 OTU355
12 OTU372
12 OTU388
12 OTU398
12 OTU469
12 OTU473
12 OTU485
12 OTU518
12 OTU534
12 OTU609
12 OTU629
12 OTU636
11 OTU369
11 OTU378
11 OTU382
11 OTU419
11 OTU423
11 OTU443
11 OTU446
11 OTU450
11 OTU475
11 OTU484
11 OTU488
11 OTU490
11 OTU492
11 OTU524
11 OTU542
11 OTU569
11 OTU573
11 OTU613
11 OTU646
11 OTU655
11 OTU660
11 OTU696
11 OTU747
11 OTU765
10 OTU416
10 OTU418
10 OTU440
10 OTU445
10 OTU449
10 OTU452
10 OTU457
10 OTU472

10	OTU474
10	OTU478
10	OTU521
10	OTU527
10	OTU528
10	OTU529
10	OTU531
10	OTU535
10	OTU548
10	OTU557
10	OTU639
10	OTU664
10	OTU756
10	OTU757
9	OTU377
9	OTU408
9	OTU421
9	OTU434
9	OTU438
9	OTU483
9	OTU497
9	OTU515
9	OTU536
9	OTU563
9	OTU572
9	OTU575
9	OTU587
9	OTU605
9	OTU640
9	OTU680
9	OTU766
9	OTU768
8	OTU429
8	OTU460
8	OTU462
8	OTU463
8	OTU482
8	OTU491
8	OTU499
8	OTU544
8	OTU552
8	OTU564
8	OTU588
8	OTU593
8	OTU596
8	OTU685
8	OTU687
8	OTU690
8	OTU693
8	OTU714
8	OTU739
8	OTU745
8	OTU763
7	OTU425

7 OTU480
7 OTU495
7 OTU507
7 OTU539
7 OTU545
7 OTU565
7 OTU592
7 OTU608
7 OTU614
7 OTU616
7 OTU688
7 OTU723
7 OTU743
7 OTU744
7 OTU752
6 OTU436
6 OTU437
6 OTU454
6 OTU459
6 OTU477
6 OTU481
6 OTU512
6 OTU523
6 OTU532
6 OTU561
6 OTU578
6 OTU583
6 OTU594
6 OTU595
6 OTU599
6 OTU603
6 OTU604
6 OTU631
6 OTU677
6 OTU681
6 OTU686
6 OTU692
6 OTU716
6 OTU717
6 OTU734
6 OTU755
5 OTU451
5 OTU489
5 OTU510
5 OTU511
5 OTU513
5 OTU516
5 OTU520
5 OTU525
5 OTU547
5 OTU550
5 OTU551
5 OTU556
5 OTU559

5 OTU566
5 OTU568
5 OTU581
5 OTU586
5 OTU597
5 OTU601
5 OTU607
5 OTU611
5 OTU612
5 OTU618
5 OTU623
5 OTU628
5 OTU630
5 OTU635
5 OTU651
5 OTU653
5 OTU689
5 OTU698
5 OTU708
5 OTU711
5 OTU729
5 OTU733
5 OTU736
5 OTU759
5 OTU764
5 OTU769
4 OTU496
4 OTU504
4 OTU505
4 OTU541
4 OTU567
4 OTU576
4 OTU579
4 OTU580
4 OTU585
4 OTU591
4 OTU606
4 OTU610
4 OTU619
4 OTU644
4 OTU650
4 OTU671
4 OTU695
4 OTU704
4 OTU728
4 OTU730
4 OTU732
4 OTU740
4 OTU741
4 OTU742
4 OTU746
4 OTU758
4 OTU762
3 OTU540

```
3 OTU553
3 OTU555
3 OTU558
3 OTU570
3 OTU589
3 OTU600
3 OTU615
3 OTU620
3 OTU632
3 OTU634
3 OTU641
3 OTU643
3 OTU647
3 OTU656
3 OTU657
3 OTU658
3 OTU666
3 OTU670
3 OTU674
3 OTU679
3 OTU683
3 OTU684
3 OTU691
3 OTU701
3 OTU702
3 OTU705
3 OTU719
3 OTU726
3 OTU749
3 OTU760
3 OTU770
2 OTU642
2 OTU648
2 OTU652
2 OTU662
2 OTU663
2 OTU665
2 OTU668
2 OTU669
2 OTU673
2 OTU678
2 OTU694
2 OTU697
2 OTU699
2 OTU703
2 OTU715
2 OTU721
2 OTU725
2 OTU727
2 OTU735
2 OTU738
2 OTU753
2 OTU754
..................................................................
```

```
............
End_E2: 14:09:54 08/01/2021
=========================================================================
Step E2 - Back-Mapping Amplicons to ZOTU
-------------------------------------------------------------------------
USEARCHusearch v11.0.667_i86linux64
Identity threshold for mapping: 0.97
-------------------------------------------------------------------------
START_E2: 11:16:09 08/01/2021
-------------------------------------------------------------------------
Counts per ZOTU:
13372386 ZOTU1
6335980 ZOTU2
4948964 ZOTU3
2196734 ZOTU4
1147693 ZOTU5
 990718 ZOTU6
 856256 ZOTU7
 839754 ZOTU9
 800972 ZOTU8
 750659 ZOTU10
 690170 ZOTU11
 615935 ZOTU12
 577302 ZOTU13
 511427 ZOTU14
 382404 ZOTU15
 317836 ZOTU16
 262021 ZOTU17
 210963 ZOTU18
 173837 ZOTU20
 157728 ZOTU19
 145469 ZOTU21
 138038 ZOTU24
 130050 ZOTU22
 125103 ZOTU26
 122667 ZOTU23
 113608 ZOTU25
 110731 ZOTU28
 102400 ZOTU29
  96700 ZOTU27
  88341 ZOTU30
  78025 ZOTU32
  77223 ZOTU31
  67283 ZOTU39
  66022 ZOTU35
  63849 ZOTU34
  63497 ZOTU40
  61666 ZOTU38
  59369 ZOTU33
  50071 ZOTU36
```

```
49542 ZOTU37
48985 ZOTU42
45418 ZOTU43
45394 ZOTU41
41960 ZOTU44
40447 ZOTU45
33589 ZOTU46
29115 ZOTU47
28921 ZOTU48
27810 ZOTU52
27028 ZOTU51
26626 ZOTU53
23992 ZOTU50
23277 ZOTU49
22484 ZOTU57
22337 ZOTU55
21242 ZOTU54
20016 ZOTU56
17855 ZOTU58
15611 ZOTU59
15346 ZOTU60
14591 ZOTU61
11671 ZOTU62
 7113 ZOTU63
 6577 ZOTU65
 6571 ZOTU67
 5859 ZOTU64
 5109 ZOTU66
 5077 ZOTU68
 4750 ZOTU70
 4615 ZOTU73
 4466 ZOTU69
 4404 ZOTU74
 3403 ZOTU71
 3160 ZOTU76
 3018 ZOTU72
 2902 ZOTU79
 2885 ZOTU77
 2776 ZOTU75
 2454 ZOTU85
 2424 ZOTU83
 2153 ZOTU78
 2096 ZOTU87
 2041 ZOTU81
 1937 ZOTU80
 1884 ZOTU84
 1862 ZOTU82
 1726 ZOTU92
 1715 ZOTU86
 1596 ZOTU88
 1501 ZOTU166
 1403 ZOTU90
 1370 ZOTU94
 1322 ZOTU97
```

1314	ZOTU100
1221	ZOTU91
1195	ZOTU95
1176	ZOTU98
1174	ZOTU96
1138	ZOTU110
1122	ZOTU99
1101	ZOTU93
1100	ZOTU89
1071	ZOTU104
1068	ZOTU116
1028	ZOTU103
971	ZOTU106
956	ZOTU111
948	ZOTU101
926	ZOTU108
913	ZOTU102
864	ZOTU105
861	ZOTU109
837	ZOTU885
821	ZOTU114
810	ZOTU119
805	ZOTU118
795	ZOTU112
793	ZOTU115
787	ZOTU107
780	ZOTU121
773	ZOTU125
760	ZOTU158
751	ZOTU133
731	ZOTU145
717	ZOTU120
706	ZOTU122
673	ZOTU117
665	ZOTU124
652	ZOTU135
650	ZOTU113
648	ZOTU131
637	ZOTU151
632	ZOTU153
627	ZOTU148
618	ZOTU230
608	ZOTU130
605	ZOTU134
588	ZOTU132
575	ZOTU127
574	ZOTU138
564	ZOTU123
562	ZOTU129
553	ZOTU694
551	ZOTU136
546	ZOTU126
533	ZOTU140
533	ZOTU172

525 ZOTU150
524 ZOTU128
522 ZOTU160
480 ZOTU137
476 ZOTU144
474 ZOTU146
474 ZOTU191
471 ZOTU147
465 ZOTU142
452 ZOTU139
450 ZOTU152
438 ZOTU143
427 ZOTU141
426 ZOTU149
420 ZOTU201
413 ZOTU163
410 ZOTU161
405 ZOTU159
402 ZOTU154
399 ZOTU171
396 ZOTU952
391 ZOTU173
383 ZOTU164
381 ZOTU156
371 ZOTU157
366 ZOTU185
364 ZOTU170
363 ZOTU174
362 ZOTU186
361 ZOTU189
356 ZOTU167
354 ZOTU178
343 ZOTU190
341 ZOTU175
341 ZOTU362
339 ZOTU184
336 ZOTU188
329 ZOTU187
326 ZOTU193
322 ZOTU176
322 ZOTU182
320 ZOTU179
318 ZOTU199
315 ZOTU162
315 ZOTU183
315 ZOTU202
315 ZOTU232
314 ZOTU180
309 ZOTU165
308 ZOTU311
306 ZOTU169
306 ZOTU181
304 ZOTU155
304 ZOTU204

302 ZOTU195
297 ZOTU200
295 ZOTU168
295 ZOTU177
292 ZOTU325
290 ZOTU194
285 ZOTU223
280 ZOTU198
279 ZOTU192
275 ZOTU203
272 ZOTU318
266 ZOTU205
264 ZOTU219
262 ZOTU196
262 ZOTU216
262 ZOTU217
259 ZOTU197
259 ZOTU381
256 ZOTU208
255 ZOTU207
255 ZOTU213
255 ZOTU221
255 ZOTU226
253 ZOTU209
248 ZOTU211
248 ZOTU212
248 ZOTU218
239 ZOTU222
239 ZOTU266
237 ZOTU224
232 ZOTU210
229 ZOTU206
228 ZOTU225
228 ZOTU229
226 ZOTU234
226 ZOTU264
224 ZOTU246
223 ZOTU220
223 ZOTU980
222 ZOTU214
222 ZOTU231
216 ZOTU215
216 ZOTU227
216 ZOTU239
212 ZOTU233
212 ZOTU238
210 ZOTU249
210 ZOTU269
209 ZOTU256
205 ZOTU273
204 ZOTU228
202 ZOTU241
202 ZOTU245
202 ZOTU262

201 ZOTU242
201 ZOTU283
198 ZOTU236
194 ZOTU244
194 ZOTU251
193 ZOTU243
193 ZOTU257
191 ZOTU333
189 ZOTU254
188 ZOTU240
187 ZOTU294
184 ZOTU388
183 ZOTU274
182 ZOTU267
177 ZOTU275
176 ZOTU258
175 ZOTU237
175 ZOTU271
174 ZOTU235
172 ZOTU255
172 ZOTU261
172 ZOTU263
171 ZOTU277
169 ZOTU247
169 ZOTU248
169 ZOTU252
169 ZOTU361
169 ZOTU468
168 ZOTU250
168 ZOTU279
167 ZOTU338
167 ZOTU368
166 ZOTU265
166 ZOTU278
165 ZOTU291
163 ZOTU260
163 ZOTU949
161 ZOTU387
160 ZOTU307
159 ZOTU259
159 ZOTU286
158 ZOTU270
157 ZOTU253
155 ZOTU302
153 ZOTU272
153 ZOTU284
152 ZOTU268
152 ZOTU280
152 ZOTU303
152 ZOTU315
150 ZOTU308
149 ZOTU312
149 ZOTU401
148 ZOTU287

147 ZOTU276
147 ZOTU297
147 ZOTU317
146 ZOTU304
144 ZOTU282
142 ZOTU285
142 ZOTU298
141 ZOTU306
140 ZOTU288
140 ZOTU328
140 ZOTU332
140 ZOTU437
140 ZOTU961
138 ZOTU316
138 ZOTU331
137 ZOTU301
135 ZOTU300
134 ZOTU299
134 ZOTU314
133 ZOTU296
133 ZOTU305
133 ZOTU345
133 ZOTU347
132 ZOTU281
132 ZOTU292
132 ZOTU321
132 ZOTU322
132 ZOTU449
131 ZOTU330
131 ZOTU390
131 ZOTU393
131 ZOTU476
131 ZOTU532
130 ZOTU313
130 ZOTU383
129 ZOTU344
128 ZOTU290
128 ZOTU396
127 ZOTU320
127 ZOTU341
126 ZOTU295
126 ZOTU323
126 ZOTU412
126 ZOTU725
125 ZOTU293
125 ZOTU398
124 ZOTU326
124 ZOTU359
124 ZOTU682
123 ZOTU319
122 ZOTU289
122 ZOTU327
122 ZOTU329
122 ZOTU343

122	ZOTU352
122	ZOTU357
121	ZOTU310
121	ZOTU334
120	ZOTU309
120	ZOTU365
120	ZOTU373
120	ZOTU413
117	ZOTU360
117	ZOTU427
117	ZOTU428
116	ZOTU367
116	ZOTU514
115	ZOTU340
114	ZOTU451
113	ZOTU335
113	ZOTU336
113	ZOTU355
113	ZOTU452
112	ZOTU418
112	ZOTU445
111	ZOTU324
110	ZOTU339
108	ZOTU376
108	ZOTU378
108	ZOTU419
108	ZOTU478
108	ZOTU489
108	ZOTU806
107	ZOTU342
107	ZOTU346
107	ZOTU421
107	ZOTU471
106	ZOTU366
106	ZOTU403
106	ZOTU434
106	ZOTU441
106	ZOTU461
106	ZOTU577
105	ZOTU408
103	ZOTU337
103	ZOTU371
103	ZOTU391
103	ZOTU493
102	ZOTU351
102	ZOTU380
102	ZOTU422
101	ZOTU363
101	ZOTU415
101	ZOTU417
100	ZOTU349
100	ZOTU377
100	ZOTU379
100	ZOTU394

100	ZOTU406
100	ZOTU414
100	ZOTU423
99	ZOTU356
99	ZOTU369
99	ZOTU426
98	ZOTU486
97	ZOTU354
97	ZOTU436
97	ZOTU438
96	ZOTU348
96	ZOTU430
95	ZOTU364
95	ZOTU375
95	ZOTU459
94	ZOTU460
93	ZOTU372
93	ZOTU453
92	ZOTU384
92	ZOTU385
92	ZOTU389
92	ZOTU433
92	ZOTU448
91	ZOTU350
91	ZOTU395
91	ZOTU497
91	ZOTU518
90	ZOTU353
90	ZOTU462
90	ZOTU540
89	ZOTU889
88	ZOTU382
88	ZOTU386
88	ZOTU464
88	ZOTU469
88	ZOTU732
87	ZOTU480
87	ZOTU915
86	ZOTU507
85	ZOTU397
85	ZOTU400
85	ZOTU402
85	ZOTU407
85	ZOTU420
85	ZOTU481
85	ZOTU547
84	ZOTU374
84	ZOTU404
84	ZOTU450
84	ZOTU662
84	ZOTU724
83	ZOTU424
83	ZOTU442
83	ZOTU533

82 ZOTU399
82 ZOTU432
82 ZOTU479
81 ZOTU392
81 ZOTU521
80 ZOTU411
80 ZOTU435
80 ZOTU439
80 ZOTU516
80 ZOTU640
80 ZOTU861
79 ZOTU358
79 ZOTU440
79 ZOTU470
79 ZOTU496
79 ZOTU508
79 ZOTU614
78 ZOTU447
78 ZOTU519
77 ZOTU405
77 ZOTU504
77 ZOTU585
77 ZOTU670
77 ZOTU717
76 ZOTU410
76 ZOTU425
76 ZOTU559
76 ZOTU637
75 ZOTU370
75 ZOTU454
75 ZOTU472
75 ZOTU513
75 ZOTU546
75 ZOTU618
74 ZOTU416
74 ZOTU483
74 ZOTU539
74 ZOTU548
74 ZOTU556
74 ZOTU590
74 ZOTU605
73 ZOTU431
73 ZOTU446
73 ZOTU455
73 ZOTU503
73 ZOTU622
72 ZOTU443
72 ZOTU477
72 ZOTU554
72 ZOTU955
71 ZOTU457
71 ZOTU498
71 ZOTU530
70 ZOTU429

70 ZOTU463
70 ZOTU466
70 ZOTU482
70 ZOTU484
70 ZOTU492
69 ZOTU409
69 ZOTU465
69 ZOTU588
68 ZOTU509
68 ZOTU560
68 ZOTU580
68 ZOTU596
68 ZOTU607
68 ZOTU731
67 ZOTU467
67 ZOTU495
67 ZOTU512
67 ZOTU536
67 ZOTU563
67 ZOTU704
66 ZOTU456
66 ZOTU568
66 ZOTU575
66 ZOTU582
66 ZOTU629
65 ZOTU474
65 ZOTU485
65 ZOTU490
65 ZOTU534
65 ZOTU656
65 ZOTU715
65 ZOTU815
64 ZOTU488
64 ZOTU502
64 ZOTU551
64 ZOTU566
64 ZOTU659
64 ZOTU910
63 ZOTU458
63 ZOTU500
63 ZOTU505
63 ZOTU515
63 ZOTU527
63 ZOTU583
62 ZOTU475
62 ZOTU494
62 ZOTU537
62 ZOTU558
62 ZOTU617
61 ZOTU517
61 ZOTU633
61 ZOTU652
61 ZOTU660
61 ZOTU738

61 ZOTU785
60 ZOTU487
60 ZOTU522
60 ZOTU528
60 ZOTU535
60 ZOTU561
60 ZOTU601
59 ZOTU531
59 ZOTU538
59 ZOTU572
59 ZOTU643
59 ZOTU855
58 ZOTU523
58 ZOTU524
58 ZOTU581
58 ZOTU589
58 ZOTU606
58 ZOTU709
57 ZOTU545
57 ZOTU615
57 ZOTU627
57 ZOTU848
57 ZOTU858
57 ZOTU917
56 ZOTU444
56 ZOTU491
56 ZOTU506
56 ZOTU555
56 ZOTU570
56 ZOTU611
56 ZOTU623
56 ZOTU668
56 ZOTU739
56 ZOTU826
56 ZOTU901
55 ZOTU544
55 ZOTU553
55 ZOTU586
55 ZOTU595
55 ZOTU632
55 ZOTU634
55 ZOTU646
55 ZOTU661
54 ZOTU550
54 ZOTU576
54 ZOTU631
54 ZOTU641
54 ZOTU707
54 ZOTU818
54 ZOTU880
53 ZOTU543
53 ZOTU567
53 ZOTU593
53 ZOTU612

53 ZOTU854
52 ZOTU473
52 ZOTU542
52 ZOTU552
52 ZOTU648
52 ZOTU684
51 ZOTU501
51 ZOTU511
51 ZOTU529
51 ZOTU609
51 ZOTU628
51 ZOTU644
51 ZOTU748
50 ZOTU510
50 ZOTU541
50 ZOTU565
50 ZOTU573
50 ZOTU578
50 ZOTU579
50 ZOTU599
50 ZOTU608
50 ZOTU649
50 ZOTU654
50 ZOTU926
49 ZOTU520
49 ZOTU525
49 ZOTU598
49 ZOTU626
49 ZOTU669
49 ZOTU726
49 ZOTU981
48 ZOTU549
48 ZOTU569
48 ZOTU651
48 ZOTU658
48 ZOTU693
48 ZOTU701
48 ZOTU711
48 ZOTU761
48 ZOTU773
48 ZOTU819
48 ZOTU896
48 ZOTU921
47 ZOTU499
47 ZOTU557
47 ZOTU602
47 ZOTU639
47 ZOTU667
47 ZOTU699
47 ZOTU769
47 ZOTU892
46 ZOTU526
46 ZOTU679
46 ZOTU681

46 ZOTU692
46 ZOTU803
46 ZOTU807
45 ZOTU564
45 ZOTU600
45 ZOTU671
45 ZOTU809
45 ZOTU822
45 ZOTU911
45 ZOTU983
44 ZOTU571
44 ZOTU587
44 ZOTU591
44 ZOTU616
44 ZOTU620
44 ZOTU625
44 ZOTU638
44 ZOTU647
44 ZOTU653
44 ZOTU696
44 ZOTU740
44 ZOTU743
44 ZOTU850
43 ZOTU592
43 ZOTU594
43 ZOTU604
43 ZOTU619
43 ZOTU621
43 ZOTU624
43 ZOTU630
43 ZOTU687
43 ZOTU755
42 ZOTU562
42 ZOTU574
42 ZOTU610
42 ZOTU613
42 ZOTU645
42 ZOTU675
42 ZOTU678
42 ZOTU700
42 ZOTU834
42 ZOTU884
41 ZOTU657
41 ZOTU664
41 ZOTU673
41 ZOTU676
41 ZOTU680
41 ZOTU729
41 ZOTU730
41 ZOTU735
41 ZOTU794
41 ZOTU872
41 ZOTU978
40 ZOTU655

40 ZOTU677
40 ZOTU722
40 ZOTU768
40 ZOTU835
40 ZOTU976
39 ZOTU603
39 ZOTU636
39 ZOTU737
39 ZOTU784
39 ZOTU791
39 ZOTU837
38 ZOTU650
38 ZOTU666
38 ZOTU708
38 ZOTU728
38 ZOTU734
38 ZOTU736
38 ZOTU762
38 ZOTU833
38 ZOTU942
37 ZOTU584
37 ZOTU672
37 ZOTU683
37 ZOTU698
37 ZOTU702
37 ZOTU721
37 ZOTU751
37 ZOTU922
36 ZOTU597
36 ZOTU674
36 ZOTU695
36 ZOTU750
36 ZOTU752
36 ZOTU765
36 ZOTU821
36 ZOTU840
35 ZOTU663
35 ZOTU697
35 ZOTU710
35 ZOTU745
35 ZOTU753
35 ZOTU757
35 ZOTU774
35 ZOTU796
35 ZOTU890
34 ZOTU635
34 ZOTU686
34 ZOTU691
34 ZOTU703
34 ZOTU705
34 ZOTU712
34 ZOTU714
34 ZOTU727
34 ZOTU733

34 ZOTU741
34 ZOTU759
34 ZOTU789
34 ZOTU792
34 ZOTU804
34 ZOTU811
34 ZOTU816
34 ZOTU843
34 ZOTU859
34 ZOTU879
34 ZOTU931
33 ZOTU690
33 ZOTU719
33 ZOTU720
33 ZOTU772
33 ZOTU777
33 ZOTU787
33 ZOTU810
33 ZOTU918
33 ZOTU957
32 ZOTU642
32 ZOTU718
32 ZOTU779
32 ZOTU786
32 ZOTU831
32 ZOTU844
32 ZOTU876
32 ZOTU878
32 ZOTU900
32 ZOTU945
32 ZOTU962
32 ZOTU964
31 ZOTU688
31 ZOTU716
31 ZOTU742
31 ZOTU744
31 ZOTU754
31 ZOTU756
31 ZOTU766
31 ZOTU771
31 ZOTU782
31 ZOTU790
31 ZOTU866
31 ZOTU875
31 ZOTU882
31 ZOTU891
31 ZOTU906
31 ZOTU909
31 ZOTU972
31 ZOTU977
30 ZOTU665
30 ZOTU689
30 ZOTU706
30 ZOTU746

30 ZOTU758
30 ZOTU763
30 ZOTU770
30 ZOTU934
30 ZOTU953
30 ZOTU974
29 ZOTU713
29 ZOTU749
29 ZOTU760
29 ZOTU764
29 ZOTU797
29 ZOTU828
29 ZOTU838
29 ZOTU871
29 ZOTU925
29 ZOTU946
28 ZOTU788
28 ZOTU801
28 ZOTU802
28 ZOTU813
28 ZOTU825
28 ZOTU827
28 ZOTU845
28 ZOTU846
28 ZOTU877
28 ZOTU988
27 ZOTU685
27 ZOTU747
27 ZOTU823
27 ZOTU829
27 ZOTU836
27 ZOTU841
27 ZOTU847
27 ZOTU849
27 ZOTU996
26 ZOTU805
26 ZOTU812
26 ZOTU874
26 ZOTU897
26 ZOTU912
26 ZOTU919
26 ZOTU937
26 ZOTU994
25 ZOTU776
25 ZOTU783
25 ZOTU793
25 ZOTU795
25 ZOTU800
25 ZOTU820
25 ZOTU867
25 ZOTU898
25 ZOTU920
25 ZOTU992
24 ZOTU767

24 ZOTU781
24 ZOTU814
24 ZOTU817
24 ZOTU824
24 ZOTU842
24 ZOTU851
24 ZOTU852
24 ZOTU863
24 ZOTU938
24 ZOTU950
24 ZOTU963
24 ZOTU969
23 ZOTU799
23 ZOTU808
23 ZOTU839
23 ZOTU857
23 ZOTU860
23 ZOTU868
23 ZOTU869
23 ZOTU870
23 ZOTU881
23 ZOTU893
23 ZOTU914
23 ZOTU940
23 ZOTU944
23 ZOTU958
23 ZOTU960
23 ZOTU986
23 ZOTU991
22 ZOTU723
22 ZOTU780
22 ZOTU887
22 ZOTU894
22 ZOTU908
22 ZOTU927
22 ZOTU929
22 ZOTU943
22 ZOTU995
21 ZOTU775
21 ZOTU832
21 ZOTU856
21 ZOTU873
21 ZOTU904
21 ZOTU916
21 ZOTU923
21 ZOTU939
21 ZOTU965
20 ZOTU830
20 ZOTU853
20 ZOTU864
20 ZOTU886
20 ZOTU899
20 ZOTU913
20 ZOTU947

```
     20 ZOTU984
     20 ZOTU990
     19 ZOTU778
     19 ZOTU798
     19 ZOTU865
     19 ZOTU903
     19 ZOTU954
     19 ZOTU973
     18 ZOTU888
     18 ZOTU902
     18 ZOTU905
     18 ZOTU932
     18 ZOTU941
     18 ZOTU967
     18 ZOTU993
     17 ZOTU883
     17 ZOTU907
     17 ZOTU924
     17 ZOTU928
     17 ZOTU930
     17 ZOTU951
     17 ZOTU956
     17 ZOTU985
     17 ZOTU989
     17 ZOTU997
     16 ZOTU895
     16 ZOTU936
     16 ZOTU959
     16 ZOTU970
     16 ZOTU975
     16 ZOTU979
     15 ZOTU948
     14 ZOTU862
     14 ZOTU933
     14 ZOTU968
     14 ZOTU971
     14 ZOTU987
     13 ZOTU935
     13 ZOTU966
     13 ZOTU982
....................................................................
............
End_E2: 13:56:57 08/01/2021
====================================================================
============
Step E2 - Back-Mapping Amplicons to ZOTU_c99
--------------------------------------------------------------------
------------
USEARCHusearch v11.0.667_i86linux64
Identity threshold for mapping: 0.97
--------------------------------------------------------------------
------------
START_E2: 11:16:05 08/01/2021
--------------------------------------------------------------------
```

```
------------
Counts per ZOTU_c99:
13372167 ZOTU1
8525187 ZOTU2
4948965 ZOTU3
1147703 ZOTU5
 990718 ZOTU6
 856284 ZOTU7
 839754 ZOTU9
 800972 ZOTU8
 750662 ZOTU10
 710086 ZOTU48
 615004 ZOTU12
 577302 ZOTU13
 511237 ZOTU14
 405473 ZOTU40
 381824 ZOTU15
 317745 ZOTU16
 262642 ZOTU17
 238944 ZOTU45
 210963 ZOTU18
 187349 ZOTU37
 145009 ZOTU21
 138055 ZOTU24
 135967 ZOTU39
 130224 ZOTU22
 125103 ZOTU26
 122712 ZOTU23
 119051 ZOTU25
 114542 ZOTU61
 110728 ZOTU28
 104585 ZOTU104
  96700 ZOTU27
  77418 ZOTU31
  66022 ZOTU35
  63849 ZOTU34
  61607 ZOTU38
  48509 ZOTU46
  45397 ZOTU41
  42029 ZOTU44
  32569 ZOTU64
  29323 ZOTU63
  29116 ZOTU47
  24010 ZOTU50
  20016 ZOTU56
  15628 ZOTU59
  11684 ZOTU62
   6577 ZOTU65
   6571 ZOTU67
   6419 ZOTU72
   5563 ZOTU639
   4615 ZOTU73
   4466 ZOTU69
   4190 ZOTU97
```

3537	ZOTU75
2902	ZOTU79
2820	ZOTU85
2424	ZOTU83
2219	ZOTU197
2153	ZOTU78
2109	ZOTU149
2096	ZOTU87
2077	ZOTU793
2062	ZOTU81
2037	ZOTU88
1937	ZOTU80
1884	ZOTU84
1862	ZOTU82
1807	ZOTU673
1758	ZOTU148
1597	ZOTU107
1501	ZOTU166
1423	ZOTU146
1403	ZOTU90
1383	ZOTU674
1370	ZOTU94
1325	ZOTU138
1318	ZOTU100
1302	ZOTU124
1221	ZOTU91
1204	ZOTU666
1199	ZOTU651
1195	ZOTU95
1152	ZOTU134
1122	ZOTU99
1108	ZOTU105
1108	ZOTU616
1103	ZOTU93
1100	ZOTU89
1030	ZOTU103
1021	ZOTU106
1003	ZOTU547
999	ZOTU675
983	ZOTU401
967	ZOTU255
948	ZOTU101
926	ZOTU108
913	ZOTU102
909	ZOTU645
879	ZOTU885
861	ZOTU109
823	ZOTU309
810	ZOTU119
808	ZOTU511
805	ZOTU118
802	ZOTU990
800	ZOTU230
795	ZOTU112

773 ZOTU125
760 ZOTU158
731 ZOTU145
717 ZOTU120
701 ZOTU425
682 ZOTU147
680 ZOTU694
671 ZOTU117
664 ZOTU200
656 ZOTU802
652 ZOTU135
650 ZOTU113
637 ZOTU151
620 ZOTU778
599 ZOTU167
598 ZOTU595
588 ZOTU132
575 ZOTU127
567 ZOTU129
565 ZOTU163
564 ZOTU123
563 ZOTU291
552 ZOTU608
551 ZOTU530
548 ZOTU126
533 ZOTU140
524 ZOTU128
513 ZOTU252
513 ZOTU844
480 ZOTU137
479 ZOTU191
476 ZOTU144
469 ZOTU310
466 ZOTU264
465 ZOTU142
458 ZOTU491
452 ZOTU139
445 ZOTU186
438 ZOTU143
437 ZOTU189
427 ZOTU141
412 ZOTU188
402 ZOTU952
400 ZOTU194
400 ZOTU218
400 ZOTU684
399 ZOTU171
391 ZOTU173
389 ZOTU613
383 ZOTU155
383 ZOTU164
382 ZOTU176
373 ZOTU157
371 ZOTU433

366 ZOTU185
363 ZOTU672
341 ZOTU175
330 ZOTU187
320 ZOTU179
320 ZOTU677
318 ZOTU199
317 ZOTU761
316 ZOTU162
315 ZOTU183
315 ZOTU202
314 ZOTU180
309 ZOTU165
308 ZOTU311
306 ZOTU169
306 ZOTU181
301 ZOTU737
300 ZOTU839
295 ZOTU168
295 ZOTU177
295 ZOTU269
292 ZOTU325
287 ZOTU654
285 ZOTU223
283 ZOTU467
275 ZOTU203
275 ZOTU646
272 ZOTU318
269 ZOTU698
269 ZOTU859
268 ZOTU589
264 ZOTU930
262 ZOTU196
262 ZOTU216
261 ZOTU787
259 ZOTU381
257 ZOTU623
256 ZOTU208
256 ZOTU403
255 ZOTU207
255 ZOTU221
252 ZOTU571
250 ZOTU559
248 ZOTU212
247 ZOTU659
245 ZOTU656
238 ZOTU406
235 ZOTU682
229 ZOTU344
228 ZOTU225
228 ZOTU229
224 ZOTU246
223 ZOTU220
223 ZOTU308

223 ZOTU980
216 ZOTU215
216 ZOTU239
216 ZOTU273
214 ZOTU653
213 ZOTU254
212 ZOTU233
211 ZOTU270
211 ZOTU508
211 ZOTU849
209 ZOTU516
208 ZOTU614
208 ZOTU648
204 ZOTU228
203 ZOTU642
202 ZOTU241
201 ZOTU242
201 ZOTU300
200 ZOTU667
194 ZOTU244
193 ZOTU243
193 ZOTU882
191 ZOTU333
187 ZOTU294
182 ZOTU267
182 ZOTU331
181 ZOTU512
178 ZOTU580
176 ZOTU592
175 ZOTU237
174 ZOTU235
172 ZOTU263
172 ZOTU594
171 ZOTU277
171 ZOTU505
169 ZOTU247
169 ZOTU361
169 ZOTU468
168 ZOTU250
168 ZOTU279
168 ZOTU679
167 ZOTU400
166 ZOTU265
163 ZOTU812
163 ZOTU949
161 ZOTU387
160 ZOTU757
159 ZOTU286
157 ZOTU253
156 ZOTU735
154 ZOTU523
154 ZOTU775
154 ZOTU989
153 ZOTU272

153 ZOTU284
150 ZOTU313
149 ZOTU312
147 ZOTU496
146 ZOTU304
146 ZOTU399
143 ZOTU437
143 ZOTU585
142 ZOTU298
142 ZOTU961
141 ZOTU285
141 ZOTU306
141 ZOTU985
140 ZOTU319
140 ZOTU332
140 ZOTU600
140 ZOTU811
139 ZOTU549
134 ZOTU314
133 ZOTU296
133 ZOTU305
133 ZOTU345
133 ZOTU347
132 ZOTU281
132 ZOTU321
132 ZOTU393
132 ZOTU449
131 ZOTU532
131 ZOTU763
130 ZOTU383
129 ZOTU460
129 ZOTU611
129 ZOTU664
129 ZOTU997
128 ZOTU290
128 ZOTU396
128 ZOTU707
127 ZOTU341
126 ZOTU293
126 ZOTU323
126 ZOTU421
126 ZOTU553
126 ZOTU725
126 ZOTU886
124 ZOTU326
124 ZOTU732
122 ZOTU343
122 ZOTU352
122 ZOTU357
121 ZOTU604
120 ZOTU373
120 ZOTU413
120 ZOTU650
117 ZOTU360

117 ZOTU365
117 ZOTU427
117 ZOTU428
116 ZOTU606
114 ZOTU451
112 ZOTU418
112 ZOTU663
112 ZOTU783
111 ZOTU324
111 ZOTU507
108 ZOTU489
108 ZOTU806
106 ZOTU434
106 ZOTU461
106 ZOTU602
106 ZOTU827
103 ZOTU337
103 ZOTU391
103 ZOTU493
103 ZOTU521
103 ZOTU902
103 ZOTU940
101 ZOTU415
101 ZOTU417
100 ZOTU349
100 ZOTU423
100 ZOTU545
99 ZOTU369
99 ZOTU426
99 ZOTU615
97 ZOTU354
97 ZOTU436
97 ZOTU438
97 ZOTU776
96 ZOTU348
95 ZOTU459
93 ZOTU372
93 ZOTU435
92 ZOTU385
92 ZOTU389
92 ZOTU448
92 ZOTU533
91 ZOTU350
91 ZOTU518
90 ZOTU540
89 ZOTU936
88 ZOTU382
88 ZOTU386
88 ZOTU469
88 ZOTU892
87 ZOTU481
87 ZOTU915
86 ZOTU442
85 ZOTU420

85 ZOTU529
84 ZOTU374
84 ZOTU404
84 ZOTU450
84 ZOTU522
84 ZOTU662
84 ZOTU724
82 ZOTU479
82 ZOTU504
82 ZOTU918
81 ZOTU392
81 ZOTU500
81 ZOTU768
81 ZOTU890
81 ZOTU914
80 ZOTU439
80 ZOTU541
80 ZOTU640
80 ZOTU861
79 ZOTU440
79 ZOTU470
79 ZOTU917
78 ZOTU620
77 ZOTU665
76 ZOTU410
76 ZOTU637
75 ZOTU513
75 ZOTU546
75 ZOTU618
75 ZOTU740
74 ZOTU483
74 ZOTU548
74 ZOTU590
74 ZOTU605
73 ZOTU431
73 ZOTU622
73 ZOTU647
73 ZOTU717
72 ZOTU458
72 ZOTU539
72 ZOTU554
72 ZOTU658
72 ZOTU955
71 ZOTU457
70 ZOTU429
70 ZOTU463
70 ZOTU482
70 ZOTU492
70 ZOTU959
70 ZOTU994
69 ZOTU447
69 ZOTU465
68 ZOTU509
68 ZOTU560

68 ZOTU596
68 ZOTU607
67 ZOTU495
67 ZOTU536
67 ZOTU563
67 ZOTU704
66 ZOTU502
66 ZOTU568
66 ZOTU575
66 ZOTU582
65 ZOTU474
65 ZOTU485
65 ZOTU490
65 ZOTU534
65 ZOTU715
65 ZOTU815
65 ZOTU865
64 ZOTU566
64 ZOTU910
63 ZOTU527
63 ZOTU583
63 ZOTU644
63 ZOTU828
62 ZOTU475
62 ZOTU494
62 ZOTU537
62 ZOTU558
62 ZOTU617
61 ZOTU517
61 ZOTU633
61 ZOTU652
61 ZOTU660
61 ZOTU738
61 ZOTU785
61 ZOTU901
60 ZOTU487
60 ZOTU535
60 ZOTU561
60 ZOTU601
60 ZOTU780
59 ZOTU531
59 ZOTU538
59 ZOTU572
59 ZOTU643
59 ZOTU855
58 ZOTU524
58 ZOTU709
58 ZOTU826
57 ZOTU627
57 ZOTU848
57 ZOTU858
56 ZOTU506
56 ZOTU555
56 ZOTU570

56 ZOTU668
56 ZOTU739
55 ZOTU544
55 ZOTU586
55 ZOTU632
55 ZOTU634
55 ZOTU661
54 ZOTU550
54 ZOTU576
54 ZOTU631
54 ZOTU641
54 ZOTU818
54 ZOTU832
54 ZOTU880
53 ZOTU543
53 ZOTU567
53 ZOTU593
53 ZOTU612
53 ZOTU854
52 ZOTU542
52 ZOTU552
51 ZOTU501
51 ZOTU609
51 ZOTU628
51 ZOTU711
51 ZOTU748
51 ZOTU881
50 ZOTU510
50 ZOTU565
50 ZOTU578
50 ZOTU579
50 ZOTU599
50 ZOTU649
50 ZOTU926
49 ZOTU525
49 ZOTU598
49 ZOTU626
49 ZOTU726
49 ZOTU981
48 ZOTU569
48 ZOTU681
48 ZOTU701
48 ZOTU773
48 ZOTU774
48 ZOTU921
47 ZOTU557
47 ZOTU699
47 ZOTU769
46 ZOTU526
46 ZOTU692
46 ZOTU803
46 ZOTU807
46 ZOTU840
45 ZOTU564

45 ZOTU671
45 ZOTU809
45 ZOTU822
45 ZOTU911
45 ZOTU983
44 ZOTU591
44 ZOTU638
44 ZOTU696
44 ZOTU850
43 ZOTU619
43 ZOTU624
43 ZOTU630
43 ZOTU687
43 ZOTU755
42 ZOTU562
42 ZOTU574
42 ZOTU678
42 ZOTU870
42 ZOTU884
41 ZOTU657
41 ZOTU676
41 ZOTU680
41 ZOTU706
41 ZOTU730
41 ZOTU794
41 ZOTU872
40 ZOTU655
40 ZOTU722
40 ZOTU835
40 ZOTU847
39 ZOTU603
39 ZOTU636
39 ZOTU784
39 ZOTU791
38 ZOTU708
38 ZOTU728
38 ZOTU736
38 ZOTU762
38 ZOTU833
38 ZOTU942
37 ZOTU584
37 ZOTU683
37 ZOTU751
37 ZOTU922
36 ZOTU597
36 ZOTU695
36 ZOTU750
36 ZOTU752
36 ZOTU765
36 ZOTU821
35 ZOTU697
35 ZOTU710
35 ZOTU745
35 ZOTU753

34 ZOTU635
34 ZOTU686
34 ZOTU691
34 ZOTU705
34 ZOTU727
34 ZOTU733
34 ZOTU741
34 ZOTU759
34 ZOTU792
34 ZOTU804
34 ZOTU843
34 ZOTU879
34 ZOTU931
33 ZOTU690
33 ZOTU720
33 ZOTU772
33 ZOTU810
33 ZOTU957
32 ZOTU779
32 ZOTU786
32 ZOTU831
32 ZOTU876
32 ZOTU878
32 ZOTU900
32 ZOTU916
32 ZOTU945
32 ZOTU962
32 ZOTU964
31 ZOTU688
31 ZOTU716
31 ZOTU754
31 ZOTU756
31 ZOTU766
31 ZOTU771
31 ZOTU782
31 ZOTU866
31 ZOTU875
31 ZOTU891
31 ZOTU906
31 ZOTU909
31 ZOTU972
30 ZOTU689
30 ZOTU770
30 ZOTU934
29 ZOTU713
29 ZOTU749
29 ZOTU764
29 ZOTU871
29 ZOTU925
29 ZOTU927
29 ZOTU946
28 ZOTU788
28 ZOTU801
28 ZOTU813

28 ZOTU845
28 ZOTU877
27 ZOTU685
27 ZOTU747
27 ZOTU823
27 ZOTU829
27 ZOTU836
27 ZOTU841
27 ZOTU893
27 ZOTU996
26 ZOTU805
26 ZOTU874
26 ZOTU897
26 ZOTU919
26 ZOTU937
25 ZOTU800
25 ZOTU851
25 ZOTU969
25 ZOTU992
24 ZOTU817
24 ZOTU852
24 ZOTU938
24 ZOTU950
24 ZOTU963
23 ZOTU808
23 ZOTU857
23 ZOTU860
23 ZOTU868
23 ZOTU869
23 ZOTU944
23 ZOTU986
23 ZOTU991
22 ZOTU908
22 ZOTU929
22 ZOTU943
22 ZOTU984
22 ZOTU995
21 ZOTU856
21 ZOTU904
21 ZOTU923
21 ZOTU939
21 ZOTU965
20 ZOTU913
19 ZOTU798
19 ZOTU973
18 ZOTU888
18 ZOTU905
18 ZOTU941
18 ZOTU967
18 ZOTU993
17 ZOTU907
17 ZOTU924
17 ZOTU928
17 ZOTU956

```
     16 ZOTU895
     16 ZOTU975
     16 ZOTU979
     15 ZOTU948
     14 ZOTU968
     14 ZOTU971
     14 ZOTU987
     13 ZOTU966
....................................................................
............
End_E2: 11:42:53 08/01/2021
====================================================================
============
Step E2 - Back-Mapping Amplicons to ZOTU_c98
--------------------------------------------------------------------
------------
USEARCHusearch v11.0.667_i86linux64
Identity threshold for mapping: 0.97
--------------------------------------------------------------------
------------
START_E2: 11:20:50 08/01/2021
--------------------------------------------------------------------
------------
Counts per ZOTU_c98:
12780472 ZOTU106
8697065 ZOTU4
4948792 ZOTU3
3316241 ZOTU59
1070015 ZOTU984
 990718 ZOTU6
 856282 ZOTU7
 839756 ZOTU9
 800972 ZOTU8
 693495 ZOTU166
 577410 ZOTU13
 548572 ZOTU40
 239427 ZOTU45
 210963 ZOTU18
 125103 ZOTU26
 125090 ZOTU168
 118859 ZOTU241
 114542 ZOTU61
 104585 ZOTU104
 100929 ZOTU530
  96700 ZOTU27
  63849 ZOTU34
  62020 ZOTU337
  45976 ZOTU124
  45962 ZOTU179
  43033 ZOTU83
  29325 ZOTU63
  27981 ZOTU163
  21797 ZOTU56
  14594 ZOTU706
```

11307 ZOTU646
9075 ZOTU806
8470 ZOTU85
6577 ZOTU65
6571 ZOTU67
6448 ZOTU373
6419 ZOTU72
6393 ZOTU151
5563 ZOTU639
5296 ZOTU167
4615 ZOTU73
4466 ZOTU69
4216 ZOTU357
4190 ZOTU97
4167 ZOTU802
4111 ZOTU547
3925 ZOTU82
3775 ZOTU711
3669 ZOTU324
3605 ZOTU381
3537 ZOTU75
3251 ZOTU839
3143 ZOTU140
3081 ZOTU393
3032 ZOTU296
2961 ZOTU815
2873 ZOTU93
2859 ZOTU413
2855 ZOTU673
2662 ZOTU186
2590 ZOTU382
2586 ZOTU145
2537 ZOTU149
2153 ZOTU78
2088 ZOTU793
2059 ZOTU148
2037 ZOTU88
1937 ZOTU80
1884 ZOTU84
1862 ZOTU888
1671 ZOTU674
1619 ZOTU158
1578 ZOTU908
1565 ZOTU658
1514 ZOTU523
1444 ZOTU886
1423 ZOTU146
1370 ZOTU94
1347 ZOTU348
1326 ZOTU100
1325 ZOTU138
1266 ZOTU694
1264 ZOTU572
1229 ZOTU493

```
1208 ZOTU666
1204 ZOTU618
1201 ZOTU651
1196 ZOTU95
1184 ZOTU160
1175 ZOTU512
1109 ZOTU616
1108 ZOTU105
1108 ZOTU114
1104 ZOTU885
1100 ZOTU89
1096 ZOTU109
1072 ZOTU675
1064 ZOTU108
1045 ZOTU682
 993 ZOTU595
 985 ZOTU401
 948 ZOTU101
 913 ZOTU102
 911 ZOTU511
 907 ZOTU958
 858 ZOTU125
 858 ZOTU265
 836 ZOTU281
 823 ZOTU309
 822 ZOTU584
 810 ZOTU119
 808 ZOTU601
 805 ZOTU118
 802 ZOTU990
 799 ZOTU310
 795 ZOTU112
 780 ZOTU107
 771 ZOTU406
 718 ZOTU120
 712 ZOTU268
 709 ZOTU647
 682 ZOTU318
 672 ZOTU117
 664 ZOTU123
 653 ZOTU956
 652 ZOTU135
 650 ZOTU113
 631 ZOTU491
 630 ZOTU290
 630 ZOTU751
 628 ZOTU344
 620 ZOTU778
 588 ZOTU132
 584 ZOTU485
 575 ZOTU127
 563 ZOTU291
 558 ZOTU128
 557 ZOTU325
```

554 ZOTU608
549 ZOTU606
548 ZOTU126
511 ZOTU545
507 ZOTU264
494 ZOTU594
482 ZOTU191
481 ZOTU660
480 ZOTU137
476 ZOTU144
469 ZOTU147
467 ZOTU243
465 ZOTU142
462 ZOTU585
460 ZOTU952
452 ZOTU139
439 ZOTU877
438 ZOTU143
437 ZOTU189
434 ZOTU505
428 ZOTU155
427 ZOTU141
427 ZOTU926
414 ZOTU654
409 ZOTU389
405 ZOTU236
399 ZOTU171
391 ZOTU173
389 ZOTU613
383 ZOTU164
373 ZOTU157
371 ZOTU433
366 ZOTU185
364 ZOTU959
363 ZOTU672
353 ZOTU870
341 ZOTU175
332 ZOTU517
320 ZOTU677
318 ZOTU199
318 ZOTU761
315 ZOTU202
315 ZOTU508
310 ZOTU221
308 ZOTU311
306 ZOTU181
305 ZOTU844
303 ZOTU737
302 ZOTU385
297 ZOTU980
295 ZOTU177
291 ZOTU513
285 ZOTU223
283 ZOTU914

272 ZOTU859
269 ZOTU698
268 ZOTU589
266 ZOTU571
264 ZOTU930
262 ZOTU216
261 ZOTU787
260 ZOTU936
259 ZOTU208
257 ZOTU623
255 ZOTU207
252 ZOTU657
248 ZOTU212
245 ZOTU656
241 ZOTU592
238 ZOTU214
230 ZOTU648
228 ZOTU229
228 ZOTU308
226 ZOTU220
226 ZOTU558
224 ZOTU246
216 ZOTU215
216 ZOTU239
216 ZOTU273
215 ZOTU653
213 ZOTU254
211 ZOTU270
209 ZOTU516
208 ZOTU614
204 ZOTU442
201 ZOTU242
201 ZOTU300
200 ZOTU667
198 ZOTU652
196 ZOTU724
195 ZOTU435
194 ZOTU244
193 ZOTU882
191 ZOTU333
188 ZOTU313
188 ZOTU725
187 ZOTU294
182 ZOTU247
182 ZOTU267
182 ZOTU331
178 ZOTU580
174 ZOTU235
172 ZOTU263
171 ZOTU277
169 ZOTU252
169 ZOTU361
169 ZOTU468
167 ZOTU665

165 ZOTU532
163 ZOTU735
161 ZOTU355
160 ZOTU757
159 ZOTU286
157 ZOTU253
156 ZOTU272
155 ZOTU284
155 ZOTU949
155 ZOTU989
149 ZOTU312
147 ZOTU496
146 ZOTU304
146 ZOTU399
142 ZOTU298
142 ZOTU811
142 ZOTU961
141 ZOTU306
141 ZOTU985
140 ZOTU319
140 ZOTU600
138 ZOTU784
134 ZOTU314
134 ZOTU345
134 ZOTU347
133 ZOTU305
132 ZOTU321
131 ZOTU763
130 ZOTU383
130 ZOTU605
129 ZOTU460
129 ZOTU611
129 ZOTU664
129 ZOTU788
128 ZOTU396
128 ZOTU707
127 ZOTU326
127 ZOTU448
126 ZOTU323
126 ZOTU569
126 ZOTU783
125 ZOTU349
125 ZOTU598
122 ZOTU289
122 ZOTU352
122 ZOTU526
121 ZOTU604
121 ZOTU650
118 ZOTU861
117 ZOTU335
117 ZOTU360
117 ZOTU427
113 ZOTU681
112 ZOTU663

111	ZOTU507
106	ZOTU434
106	ZOTU525
106	ZOTU827
104	ZOTU655
103	ZOTU391
103	ZOTU394
103	ZOTU902
99	ZOTU369
99	ZOTU426
99	ZOTU469
99	ZOTU578
99	ZOTU615
99	ZOTU688
97	ZOTU354
97	ZOTU436
97	ZOTU438
97	ZOTU776
96	ZOTU619
95	ZOTU459
95	ZOTU529
93	ZOTU617
92	ZOTU901
91	ZOTU518
90	ZOTU540
90	ZOTU855
88	ZOTU386
88	ZOTU892
87	ZOTU915
86	ZOTU994
85	ZOTU420
84	ZOTU374
84	ZOTU404
84	ZOTU450
84	ZOTU522
84	ZOTU662
83	ZOTU533
82	ZOTU479
82	ZOTU504
82	ZOTU620
82	ZOTU890
82	ZOTU918
81	ZOTU738
81	ZOTU768
80	ZOTU439
80	ZOTU500
80	ZOTU541
80	ZOTU640
80	ZOTU874
79	ZOTU470
79	ZOTU917
78	ZOTU447
76	ZOTU410
76	ZOTU637

75 ZOTU546
75 ZOTU740
74 ZOTU483
74 ZOTU590
73 ZOTU431
73 ZOTU554
73 ZOTU717
73 ZOTU897
72 ZOTU955
71 ZOTU457
70 ZOTU429
70 ZOTU463
70 ZOTU482
69 ZOTU465
68 ZOTU560
68 ZOTU596
68 ZOTU607
67 ZOTU495
67 ZOTU536
67 ZOTU563
67 ZOTU704
67 ZOTU741
66 ZOTU568
66 ZOTU575
65 ZOTU474
65 ZOTU490
65 ZOTU534
65 ZOTU715
64 ZOTU566
64 ZOTU910
63 ZOTU527
63 ZOTU583
63 ZOTU644
63 ZOTU828
62 ZOTU475
62 ZOTU494
62 ZOTU537
62 ZOTU992
61 ZOTU633
61 ZOTU755
61 ZOTU785
61 ZOTU843
60 ZOTU535
60 ZOTU561
60 ZOTU818
59 ZOTU531
59 ZOTU538
59 ZOTU643
58 ZOTU557
58 ZOTU627
58 ZOTU709
58 ZOTU826
57 ZOTU848
57 ZOTU858

56 ZOTU555
56 ZOTU570
56 ZOTU668
55 ZOTU544
55 ZOTU586
55 ZOTU632
55 ZOTU634
55 ZOTU661
54 ZOTU550
54 ZOTU576
54 ZOTU631
54 ZOTU641
54 ZOTU832
54 ZOTU880
53 ZOTU543
53 ZOTU567
53 ZOTU593
53 ZOTU612
53 ZOTU854
52 ZOTU542
52 ZOTU565
51 ZOTU501
51 ZOTU609
51 ZOTU628
51 ZOTU748
50 ZOTU510
50 ZOTU579
50 ZOTU599
50 ZOTU649
50 ZOTU981
49 ZOTU626
49 ZOTU726
48 ZOTU701
48 ZOTU773
48 ZOTU774
48 ZOTU921
47 ZOTU699
47 ZOTU934
46 ZOTU679
46 ZOTU692
46 ZOTU803
46 ZOTU807
46 ZOTU840
45 ZOTU564
45 ZOTU671
45 ZOTU809
45 ZOTU911
45 ZOTU983
44 ZOTU591
44 ZOTU638
44 ZOTU696
44 ZOTU850
44 ZOTU851
43 ZOTU624

43 ZOTU630
43 ZOTU687
42 ZOTU562
42 ZOTU574
42 ZOTU678
42 ZOTU884
41 ZOTU676
41 ZOTU730
41 ZOTU794
41 ZOTU821
40 ZOTU722
40 ZOTU835
39 ZOTU603
39 ZOTU636
38 ZOTU708
38 ZOTU728
38 ZOTU736
38 ZOTU747
38 ZOTU762
38 ZOTU833
38 ZOTU942
37 ZOTU683
37 ZOTU922
36 ZOTU597
36 ZOTU695
36 ZOTU752
36 ZOTU765
35 ZOTU697
35 ZOTU710
35 ZOTU745
34 ZOTU635
34 ZOTU727
34 ZOTU733
34 ZOTU759
34 ZOTU792
34 ZOTU804
34 ZOTU879
34 ZOTU931
33 ZOTU690
33 ZOTU720
33 ZOTU810
32 ZOTU779
32 ZOTU786
32 ZOTU831
32 ZOTU876
32 ZOTU878
32 ZOTU900
32 ZOTU916
32 ZOTU945
32 ZOTU962
32 ZOTU964
31 ZOTU716
31 ZOTU754
31 ZOTU756

31 ZOTU764
31 ZOTU766
31 ZOTU771
31 ZOTU808
31 ZOTU866
31 ZOTU891
31 ZOTU906
30 ZOTU689
29 ZOTU713
29 ZOTU871
29 ZOTU925
29 ZOTU927
28 ZOTU801
28 ZOTU845
27 ZOTU685
27 ZOTU823
27 ZOTU836
27 ZOTU841
27 ZOTU893
27 ZOTU996
26 ZOTU805
26 ZOTU919
26 ZOTU937
25 ZOTU800
25 ZOTU969
24 ZOTU817
24 ZOTU869
24 ZOTU938
24 ZOTU950
24 ZOTU963
23 ZOTU857
23 ZOTU860
23 ZOTU868
23 ZOTU944
23 ZOTU986
23 ZOTU991
22 ZOTU929
22 ZOTU943
22 ZOTU995
22 ZOTU997
21 ZOTU856
21 ZOTU904
21 ZOTU923
21 ZOTU965
20 ZOTU913
19 ZOTU798
19 ZOTU973
18 ZOTU905
18 ZOTU967
18 ZOTU993
17 ZOTU907
17 ZOTU928
16 ZOTU895
16 ZOTU975

```
     16 ZOTU979
     15 ZOTU948
     14 ZOTU968
     14 ZOTU971
..................................................................
............
End_E2: 11:47:44 08/01/2021
==================================================================
============
Step E2 - Back-Mapping Amplicons to ZOTU_c97
------------------------------------------------------------------
------------
USEARCHusearch v11.0.667_i86linux64
Identity threshold for mapping: 0.97
------------------------------------------------------------------
------------
START_E2: 11:20:50 08/01/2021
------------------------------------------------------------------
------------
Counts per ZOTU_c97:
12780472 ZOTU106
8628063 ZOTU124
4201581 ZOTU949
1071722 ZOTU984
 990718 ZOTU6
 916283 ZOTU660
 839758 ZOTU9
 800972 ZOTU8
 676211 ZOTU167
 630056 ZOTU532
 626294 ZOTU572
 601395 ZOTU13
 544155 ZOTU523
 288121 ZOTU821
 242634 ZOTU149
 210963 ZOTU18
 185801 ZOTU606
 158824 ZOTU468
 131012 ZOTU658
 126992 ZOTU385
 125294 ZOTU208
 125103 ZOTU26
 114542 ZOTU61
 100929 ZOTU530
  98531 ZOTU138
  96700 ZOTU27
  63849 ZOTU34
  62257 ZOTU815
  62020 ZOTU337
  49153 ZOTU179
  48471 ZOTU745
  47723 ZOTU761
  30639 ZOTU653
  28738 ZOTU517
```

22280	ZOTU580
21949	ZOTU56
21776	ZOTU657
17344	ZOTU952
16069	ZOTU706
11307	ZOTU646
10803	ZOTU619
10198	ZOTU675
9076	ZOTU806
8776	ZOTU325
7267	ZOTU263
6935	ZOTU674
6844	ZOTU665
6577	ZOTU65
6571	ZOTU67
6419	ZOTU72
6402	ZOTU571
6106	ZOTU247
5974	ZOTU511
5563	ZOTU639
5174	ZOTU648
4761	ZOTU558
4692	ZOTU215
4615	ZOTU73
4466	ZOTU69
4289	ZOTU97
4208	ZOTU505
4111	ZOTU547
3775	ZOTU711
3537	ZOTU75
3511	ZOTU983
3284	ZOTU161
3099	ZOTU808
3016	ZOTU608
2994	ZOTU673
2942	ZOTU326
2859	ZOTU413
2753	ZOTU323
2710	ZOTU997
2699	ZOTU145
2662	ZOTU186
2628	ZOTU959
2513	ZOTU990
2485	ZOTU994
2344	ZOTU651
2256	ZOTU557
2228	ZOTU448
2170	ZOTU843
2153	ZOTU78
2075	ZOTU947
2059	ZOTU148
1937	ZOTU80
1884	ZOTU84
1876	ZOTU107

1731	ZOTU100
1731	ZOTU303
1635	ZOTU147
1431	ZOTU985
1423	ZOTU146
1370	ZOTU94
1265	ZOTU694
1250	ZOTU545
1219	ZOTU666
1200	ZOTU95
1174	ZOTU916
1136	ZOTU620
1110	ZOTU616
1108	ZOTU105
1104	ZOTU885
1100	ZOTU89
1040	ZOTU828
948	ZOTU101
945	ZOTU584
933	ZOTU598
915	ZOTU310
914	ZOTU102
905	ZOTU491
891	ZOTU309
885	ZOTU228
883	ZOTU902
870	ZOTU318
858	ZOTU125
810	ZOTU119
805	ZOTU118
795	ZOTU112
761	ZOTU844
738	ZOTU268
718	ZOTU120
696	ZOTU956
682	ZOTU936
652	ZOTU135
650	ZOTU113
642	ZOTU433
642	ZOTU693
630	ZOTU751
620	ZOTU778
588	ZOTU132
575	ZOTU127
570	ZOTU654
563	ZOTU291
545	ZOTU663
543	ZOTU594
530	ZOTU246
507	ZOTU264
476	ZOTU144
468	ZOTU652
465	ZOTU142
457	ZOTU171

452 ZOTU139
439 ZOTU877
438 ZOTU143
437 ZOTU189
428 ZOTU155
427 ZOTU141
416 ZOTU870
415 ZOTU676
409 ZOTU389
394 ZOTU583
391 ZOTU173
389 ZOTU613
383 ZOTU164
366 ZOTU185
345 ZOTU202
320 ZOTU677
318 ZOTU199
315 ZOTU508
310 ZOTU221
309 ZOTU531
309 ZOTU882
308 ZOTU311
306 ZOTU181
288 ZOTU442
285 ZOTU223
281 ZOTU655
276 ZOTU659
273 ZOTU589
270 ZOTU541
269 ZOTU345
269 ZOTU698
264 ZOTU930
262 ZOTU216
261 ZOTU787
259 ZOTU777
255 ZOTU207
255 ZOTU592
245 ZOTU347
245 ZOTU656
239 ZOTU650
237 ZOTU702
237 ZOTU735
236 ZOTU220
228 ZOTU229
223 ZOTU811
216 ZOTU239
213 ZOTU254
209 ZOTU516
209 ZOTU603
208 ZOTU614
201 ZOTU242
201 ZOTU300
196 ZOTU529
194 ZOTU244

```
191 ZOTU333
188 ZOTU989
187 ZOTU294
187 ZOTU864
184 ZOTU879
176 ZOTU628
175 ZOTU235
175 ZOTU272
174 ZOTU587
172 ZOTU434
171 ZOTU277
169 ZOTU361
168 ZOTU679
160 ZOTU757
159 ZOTU286
157 ZOTU253
155 ZOTU961
149 ZOTU312
147 ZOTU496
146 ZOTU304
146 ZOTU399
146 ZOTU429
144 ZOTU447
143 ZOTU298
141 ZOTU306
140 ZOTU600
138 ZOTU784
137 ZOTU788
136 ZOTU575
133 ZOTU305
131 ZOTU763
130 ZOTU383
129 ZOTU392
129 ZOTU460
129 ZOTU554
129 ZOTU611
129 ZOTU664
128 ZOTU707
126 ZOTU783
123 ZOTU826
122 ZOTU352
118 ZOTU861
117 ZOTU360
117 ZOTU427
113 ZOTU681
111 ZOTU507
106 ZOTU827
103 ZOTU391
102 ZOTU704
101 ZOTU688
 99 ZOTU369
 99 ZOTU426
 99 ZOTU578
 99 ZOTU615
```

97 ZOTU354
97 ZOTU436
97 ZOTU438
97 ZOTU776
95 ZOTU459
93 ZOTU617
92 ZOTU499
92 ZOTU901
91 ZOTU518
91 ZOTU637
90 ZOTU540
90 ZOTU855
88 ZOTU386
88 ZOTU892
87 ZOTU915
85 ZOTU420
85 ZOTU563
84 ZOTU374
84 ZOTU404
84 ZOTU450
84 ZOTU662
84 ZOTU920
82 ZOTU595
82 ZOTU918
81 ZOTU768
80 ZOTU439
80 ZOTU640
80 ZOTU874
79 ZOTU470
79 ZOTU917
76 ZOTU410
75 ZOTU546
75 ZOTU981
74 ZOTU483
74 ZOTU590
73 ZOTU431
72 ZOTU612
72 ZOTU955
71 ZOTU457
70 ZOTU482
70 ZOTU644
68 ZOTU596
68 ZOTU607
67 ZOTU536
67 ZOTU576
67 ZOTU741
67 ZOTU759
67 ZOTU770
66 ZOTU568
66 ZOTU692
65 ZOTU490
65 ZOTU534
65 ZOTU715
64 ZOTU566

64 ZOTU910
63 ZOTU527
62 ZOTU475
62 ZOTU494
62 ZOTU537
62 ZOTU633
62 ZOTU992
62 ZOTU993
61 ZOTU561
61 ZOTU785
60 ZOTU535
59 ZOTU538
59 ZOTU643
58 ZOTU709
57 ZOTU848
57 ZOTU858
56 ZOTU555
56 ZOTU570
56 ZOTU668
55 ZOTU544
55 ZOTU586
55 ZOTU632
55 ZOTU634
55 ZOTU661
54 ZOTU550
54 ZOTU631
54 ZOTU641
54 ZOTU832
54 ZOTU880
53 ZOTU543
53 ZOTU567
53 ZOTU593
53 ZOTU854
52 ZOTU542
51 ZOTU501
51 ZOTU609
51 ZOTU748
50 ZOTU510
50 ZOTU579
50 ZOTU599
50 ZOTU649
49 ZOTU626
49 ZOTU726
48 ZOTU687
48 ZOTU773
48 ZOTU921
47 ZOTU699
46 ZOTU803
46 ZOTU807
46 ZOTU840
46 ZOTU975
45 ZOTU564
45 ZOTU671
45 ZOTU809

44 ZOTU591
44 ZOTU638
44 ZOTU696
44 ZOTU850
44 ZOTU871
43 ZOTU624
43 ZOTU630
43 ZOTU941
42 ZOTU562
42 ZOTU574
42 ZOTU884
41 ZOTU730
41 ZOTU794
40 ZOTU722
40 ZOTU835
39 ZOTU636
38 ZOTU708
38 ZOTU728
38 ZOTU736
38 ZOTU833
38 ZOTU942
37 ZOTU683
37 ZOTU922
36 ZOTU597
36 ZOTU635
36 ZOTU695
36 ZOTU752
36 ZOTU765
35 ZOTU710
35 ZOTU869
34 ZOTU727
34 ZOTU733
34 ZOTU792
34 ZOTU931
33 ZOTU720
33 ZOTU810
33 ZOTU831
32 ZOTU779
32 ZOTU878
32 ZOTU900
32 ZOTU962
31 ZOTU716
31 ZOTU754
31 ZOTU764
31 ZOTU766
31 ZOTU771
31 ZOTU866
31 ZOTU891
31 ZOTU906
30 ZOTU689
29 ZOTU713
29 ZOTU925
28 ZOTU845
27 ZOTU685

```
     27 ZOTU805
     27 ZOTU836
     27 ZOTU841
     26 ZOTU919
     26 ZOTU937
     25 ZOTU800
     24 ZOTU817
     24 ZOTU938
     24 ZOTU950
     24 ZOTU963
     23 ZOTU857
     23 ZOTU868
     23 ZOTU944
     23 ZOTU986
     22 ZOTU929
     22 ZOTU995
     21 ZOTU856
     21 ZOTU904
     21 ZOTU923
     21 ZOTU965
     19 ZOTU798
     19 ZOTU973
     18 ZOTU905
     18 ZOTU967
     17 ZOTU907
     17 ZOTU928
     16 ZOTU895
     16 ZOTU979
     15 ZOTU948
     14 ZOTU968
     14 ZOTU971
....................................................................
............
End_E2: 12:00:55 08/01/2021
====================================================================
============
Step E3 | Table Reports / MSA / Trees / Stats
--------------------------------------------------------------------
------------
USEARCH: usearch v11.0.667_i86linux64
--------------------------------------------------------------------
------------
START_E3: 14:11:21 08/01/2021
--------------------------------------------------------------------
------------
▶ ZOTU Table Report
....................................................................
............
  Create summary-reports for the (Z)OTU tables > *_Count.summary
  • Summary for OTU ✔
  • Summary for ZOTU ✔
  • Summary for ZOTU_c99 ✔
  • Summary for ZOTU_c98 ✔
  • Summary for ZOTU_c97 ✔
```

```
--------------------------------------------------------------------
------------
▶ Distance matrices (for Octave plots)
....................................................................
............
  Pairwise distances between (Z)OTU sequences.
  • DM for OTU ✔
  • DM for ZOTU ✔
  • DM for ZOTU_c99 ✔
  • DM for ZOTU_c98 ✔
  • DM for ZOTU_c97 ✔
--------------------------------------------------------------------
------------
▶ Octave plots
....................................................................
............
  Octave plots with low-abundance (Z)OTUs and cross-talk
information.
  • Octave plot for OTU ✔
  • Octave plot for ZOTU ✔
  • Octave plot for ZOTU_c99 ✔
  • Octave plot for ZOTU_c98 ✔
  • Octave plot for ZOTU_c97 ✔
--------------------------------------------------------------------
------------
▶ Approximate Phylogenetic Trees
....................................................................
............
  Create tree files.
  The trees will be very approximate in both cases.
  ◦ Multiple Sequence Alignments (MSA)
  MUSCLE v3.8.1551 by Robert C. Edgar
   • MSA tree for OTU ✔
   • MSA tree for ZOTU ✔
   • MSA tree for ZOTU_c99 ✔
   • MSA tree for ZOTU_c98 ✔
   • MSA tree for ZOTU_c97 ✔
  ◦ Cluster Trees (CLU)
  usearch v11.0.667_i86linux64
   • CLU tree for OTU ✔
   • CLU tree for ZOTU ✔
   • CLU tree for ZOTU_c99 ✔
   • CLU tree for ZOTU_c98 ✔
   • CLU tree for ZOTU_c97 ✔
--------------------------------------------------------------------
------------
▶ Uncross
....................................................................
............
  Detects and filters cross-talk (sample mis-assignment) in a OTU
table using the UNCROSS algorithm.
  • Uncross for for OTU ✔
  • Uncross for for ZOTU ✔
  • Uncross for for ZOTU_c99 ✔
```

```
  • Uncross for for ZOTU_c98 ✔
  • Uncross for for ZOTU_c97 ✔
--------------------------------------------------------------------
------------
▶ Back-Mapping Efficiency
....................................................................
............
N(Amplicons)      = 39635349
N(Map-2-OTU)      = 38543837 (.972)
N(Map-2-ZOTU)     = 38780506 (.978)
N(Map-2-ZOTU_c99) = 38757668 (.977)
N(Map-2-ZOTU_c98) = 37995942 (.958)
N(Map-2-ZOTU_c97) = 36106582 (.910)
--------------------------------------------------------------------
------------
END_E3: 14:13:32 08/01/2021
====================================================================
============
Step F | Taxonomic Assignment-Predictions with SINTAX
--------------------------------------------------------------------
------------
Application   : SINTAX (v11.0.667_i86linux64)
Reference     : SILVA v128
Tax Filter    : 0.85
====================================================================
============
Tax-Prediction Workflow Summary:
 (F1) Prepare Reference
 (F2) Assign Taxa with SINTAX
 (F3) Reformat Tax-Information
 (F4) Combine Count-Table and Taxa for Phyloseq Import
 (F5) Tax-Assignment Report for Phylum Level
 (F6) Additional Chimera Check (Not Needed)
-----------------------------------------------------
START_Tax-Prediction: 14:13:51 08/01/2021
 Start_F1_Restrict_Reference: 14:13:51 08/01/2021
  ‣Number of Reference Sequences:  341153
  ‣OTU related References:         322033
  ‣ZOTU related References:        321810
 End_F1_Restrict_Reference: 14:48:47 08/01/2021
-----------------------------------------------------
 Start_F1_Unique_Records: 14:48:47 08/01/2021
  ‣OTU: N(seq)= 322033 / N(uniques)= 271885 / N(singletons)= 256648
(94.4%)
  ‣OTU: Min size 1 / median 1 / max 515 / avg 1.18
  ‣ZOTU: N(seq)= 321810 / N(uniques)= 271653 / N(singletons)= 256414
(94.4%)
  ‣ZOTU: Min size 1 / median 1 / max 515 / avg 1.18
 End_F1_Unique_Records: 14:48:53 08/01/2021
-----------------------------------------------------
 Start_F1_Build_UPD_Reference: 14:48:53 08/01/2021
 End_F1_Build_UPD_Reference: 14:49:14 08/01/2021
-----------------------------------------------------
 Start_F2_Tax-Assignment: 14:49:14 08/01/2021
```

```
 End_F2_Tax-Assignment: 14:57:18 08/01/2021
--------------------------------------------------
 Start_F3_Re-Format: 14:57:18 08/01/2021
 End_F3_Re-Format: 14:57:18 08/01/2021
--------------------------------------------------
 Start_F4_Combine_CountTable_TaxLabel: 14:57:18 08/01/2021
 End_F4_Combine_CountTable_TaxLabel: 14:57:19 08/01/2021
--------------------------------------------------
 Start_F5_Tax-Summary: 14:57:19 08/01/2021
  Tax Summary for OTU:
   Cyanobacteria 326     42.2    42.2
   Proteobacteria        181     23.4    65.7
   Planctomycetes        59      7.6     73.3
   Firmicutes    56      7.3     80.6
   (Unassigned)  40      5.2     85.8
   Actinobacteria        39      5.1     90.8
   Bacteroidetes 19      2.5     93.3
   Verrucomicrobia       14      1.8     95.1
   Acidobacteria 11      1.4     96.5
   Chloroflexi   9       1.2     97.7
   Parcubacteria 6       0.8     98.4
   Saccharibacteria      4       0.5     99.0
   Deinococcus-Thermus   2       0.3     99.2
   Gemmatimonadetes      2       0.3     99.5
   TM6_Dependentiae      1       0.1     99.6
   FBP   1       0.1     99.7
   Latescibacteria       1       0.1     99.9
   Nitrospirae   1       0.1     100.0
  - - -
  Tax Summary for ZOTU:
   Cyanobacteria 667     66.9    66.9
   Proteobacteria        190     19.1    86.0
   Firmicutes    44      4.4     90.4
   Planctomycetes        31      3.1     93.5
   Actinobacteria        22      2.2     95.7
   (Unassigned)  14      1.4     97.1
   Bacteroidetes 10      1.0     98.1
   Verrucomicrobia       7       0.7     98.8
   Chloroflexi   3       0.3     99.1
   Gemmatimonadetes      3       0.3     99.4
   TM6_Dependentiae      1       0.1     99.5
   Saccharibacteria      1       0.1     99.6
   Acidobacteria 1       0.1     99.7
   Deinococcus-Thermus   1       0.1     99.8
   Parcubacteria 1       0.1     99.9
   Nitrospirae   1       0.1     100.0
 End_F5_Tax-Summary: 14:57:19 08/01/2021
--------------------------------------------------
 Start_F6_Chimera-Check: 14:57:19 08/01/2021
  Additional Chimera-Check
  Note: Although the high_confidence mode is used for chimera
detection,
        this is an extra step with a high risk of false positives.
        There is already a sufficient built-in de novo chimera
```

```
filtering
        as part of the UPARSE or UNOISE workflow. This extra step
might
        be useful if applied with caution.
  For details see -> e_OTU/p257_runMIX_16S_HG_(Z)OTU_chimera.txt
..................................................
  Chimera-Check for OTU: 6
  Chimera-Check for ZOTU: 8
  Chimera-Check for ZOTU_c99: 8
  Chimera-Check for ZOTU_c98: 7
  Chimera-Check for ZOTU_c97: 6
 End_F6_Chimera-Check: 14:58:09 08/01/2021
--------------------------------------------------
END_Tax-Prediction: 14:58:10 08/01/2021
--------------------------------------------------------------------
------------
END_Workflow: 14:58:17 08/01/2021
====================================================================
============
```
