# Supplementary material for: Genetic variation and microbiota in bumble bees cross-infected by different strains of C. bombi
Source: PLoS One. 2022 Nov 28;17(11):e0277041. doi: 10.1371/journal.pone.0277041 (PMC9704641; doi:10.1371/journal.pone.0277041)
Supplement: S11 File — This file contains a list of all samples from which the bacterial metagenome was typed and deposited with ENA (accession PRJEB52013). We used universal primers for the V3/V4 region of 16S RNA. A key to sample numbering is given. (PDF) [file pone.0277041.s011.pdf]

File D1. List of samples deposited with ENA.
Accession: PRJEB52013
Secondary accession, Name: ERP136687
Secondary accession, files: ERR100855298 … ERR100855160

Coding for samples is as follows:
- 'HG': lab-internal name of experiment.
- first three digits: colony number (e.g. '015')
- first digit after dash: parasite strain (e.g. 'B')
- last number: replicate within this host x parasite combination (e.g. '1' = first replicate)

Note that the two replicates 'HG015-B2' and 'HG319-Q1' were excluded due to technical problems as mentioned in the text.

a

sample	study	instrument_model	library_source	library_selection	library_strategy	library_layout	forward_file_name	reverse_file_name
**HG015-B1**	PRJEB52013	Illumina MiSeq	METAGENOMIC	PCR	AMPLICON	PAIRED	HG015-B1_R1.fq.gz	HG015-B1_R2.fq.gz
**HG015-B3**	PRJEB52013	Illumina MiSeq	METAGENOMIC	PCR	AMPLICON	PAIRED	HG015-B3_R1.fq.gz	HG015-B3_R2.fq.gz
**HG015-D1**	PRJEB52013	Illumina MiSeq	METAGENOMIC	PCR	AMPLICON	PAIRED	HG015-D1_R1.fq.gz	HG015-D1_R2.fq.gz
**HG015-D2**	PRJEB52013	Illumina MiSeq	METAGENOMIC	PCR	AMPLICON	PAIRED	HG015-D2_R1.fq.gz	HG015-D2_R2.fq.gz
**HG015-D3**	PRJEB52013	Illumina MiSeq	METAGENOMIC	PCR	AMPLICON	PAIRED	HG015-D3_R1.fq.gz	HG015-D3_R2.fq.gz
**HG015-I1**	PRJEB52013	Illumina MiSeq	METAGENOMIC	PCR	AMPLICON	PAIRED	HG015-I1_R1.fq.gz	HG015-I1_R2.fq.gz
**HG015-I2**	PRJEB52013	Illumina MiSeq	METAGENOMIC	PCR	AMPLICON	PAIRED	HG015-I2_R1.fq.gz	HG015-I2_R2.fq.gz
**HG015-I3**	PRJEB52013	Illumina MiSeq	METAGENOMIC	PCR	AMPLICON	PAIRED	HG015-I3_R1.fq.gz	HG015-I3_R2.fq.gz
**HG015-J1**	PRJEB52013	Illumina MiSeq	METAGENOMIC	PCR	AMPLICON	PAIRED	HG015-J1_R1.fq.gz	HG015-J1_R2.fq.gz
**HG015-J2**	PRJEB52013	Illumina MiSeq	METAGENOMIC	PCR	AMPLICON	PAIRED	HG015-J2_R1.fq.gz	HG015-J2_R2.fq.gz
**HG015-J3**	PRJEB52013	Illumina MiSeq	METAGENOMIC	PCR	AMPLICON	PAIRED	HG015-J3_R1.fq.gz	HG015-J3_R2.fq.gz
**HG015-L1**	PRJEB52013	Illumina MiSeq	METAGENOMIC	PCR	AMPLICON	PAIRED	HG015-L1_R1.fq.gz	HG015-L1_R2.fq.gz
**HG015-L2**	PRJEB52013	Illumina MiSeq	METAGENOMIC	PCR	AMPLICON	PAIRED	HG015-L2_R1.fq.gz	HG015-L2_R2.fq.gz
**HG015-L3**	PRJEB52013	Illumina MiSeq	METAGENOMIC	PCR	AMPLICON	PAIRED	HG015-L3_R1.fq.gz	HG015-L3_R2.fq.gz
**HG015-O1**	PRJEB52013	Illumina MiSeq	METAGENOMIC	PCR	AMPLICON	PAIRED	HG015-O1_R1.fq.gz	HG015-O1_R2.fq.gz
**HG015-O2**	PRJEB52013	Illumina MiSeq	METAGENOMIC	PCR	AMPLICON	PAIRED	HG015-O2_R1.fq.gz	HG015-O2_R2.fq.gz
**HG015-O3**	PRJEB52013	Illumina MiSeq	METAGENOMIC	PCR	AMPLICON	PAIRED	HG015-O3_R1.fq.gz	HG015-O3_R2.fq.gz
**HG015-Q1**	PRJEB52013	Illumina MiSeq	METAGENOMIC	PCR	AMPLICON	PAIRED	HG015-Q1_R1.fq.gz	HG015-Q1_R2.fq.gz
**HG015-Q2**	PRJEB52013	Illumina MiSeq	METAGENOMIC	PCR	AMPLICON	PAIRED	HG015-Q2_R1.fq.gz	HG015-Q2_R2.fq.gz
**HG015-Q3**	PRJEB52013	Illumina MiSeq	METAGENOMIC	PCR	AMPLICON	PAIRED	HG015-Q3_R1.fq.gz	HG015-Q3_R2.fq.gz
**HG015-R1**	PRJEB52013	Illumina MiSeq	METAGENOMIC	PCR	AMPLICON	PAIRED	HG015-R1_R1.fq.gz	HG015-R1_R2.fq.gz
**HG015-R2**	PRJEB52013	Illumina MiSeq	METAGENOMIC	PCR	AMPLICON	PAIRED	HG015-R2_R1.fq.gz	HG015-R2_R2.fq.gz
**HG015-R3**	PRJEB52013	Illumina MiSeq	METAGENOMIC	PCR	AMPLICON	PAIRED	HG015-R3_R1.fq.gz	HG015-R3_R2.fq.gz
**HG033-B1**	PRJEB52013	Illumina MiSeq	METAGENOMIC	PCR	AMPLICON	PAIRED	HG033-B1_R1.fq.gz	HG033-B1_R2.fq.gz
**HG033-B2**	PRJEB52013	Illumina MiSeq	METAGENOMIC	PCR	AMPLICON	PAIRED	HG033-B2_R1.fq.gz	HG033-B2_R2.fq.gz
**HG033-B3**	PRJEB52013	Illumina MiSeq	METAGENOMIC	PCR	AMPLICON	PAIRED	HG033-B3_R1.fq.gz	HG033-B3_R2.fq.gz
**HG033-D1**	PRJEB52013	Illumina MiSeq	METAGENOMIC	PCR	AMPLICON	PAIRED	HG033-D1_R1.fq.gz	HG033-D1_R2.fq.gz
**HG033-D2**	PRJEB52013	Illumina MiSeq	METAGENOMIC	PCR	AMPLICON	PAIRED	HG033-D2_R1.fq.gz	HG033-D2_R2.fq.gz
**HG033-D3**	PRJEB52013	Illumina MiSeq	METAGENOMIC	PCR	AMPLICON	PAIRED	HG033-D3_R1.fq.gz	HG033-D3_R2.fq.gz
**HG033-I1**	PRJEB52013	Illumina MiSeq	METAGENOMIC	PCR	AMPLICON	PAIRED	HG033-I1_R1.fq.gz	HG033-I1_R2.fq.gz
**HG033-I2**	PRJEB52013	Illumina MiSeq	METAGENOMIC	PCR	AMPLICON	PAIRED	HG033-I2_R1.fq.gz	HG033-I2_R2.fq.gz
**HG033-I3**	PRJEB52013	Illumina MiSeq	METAGENOMIC	PCR	AMPLICON	PAIRED	HG033-I3_R1.fq.gz	HG033-I3_R2.fq.gz
**HG033-J1**	PRJEB52013	Illumina MiSeq	METAGENOMIC	PCR	AMPLICON	PAIRED	HG033-J1_R1.fq.gz	HG033-J1_R2.fq.gz
**HG033-J2**	PRJEB52013	Illumina MiSeq	METAGENOMIC	PCR	AMPLICON	PAIRED	HG033-J2_R1.fq.gz	HG033-J2_R2.fq.gz
**HG033-J3**	PRJEB52013	Illumina MiSeq	METAGENOMIC	PCR	AMPLICON	PAIRED	HG033-J3_R1.fq.gz	HG033-J3_R2.fq.gz
**HG033-L1**	PRJEB52013	Illumina MiSeq	METAGENOMIC	PCR	AMPLICON	PAIRED	HG033-L1_R1.fq.gz	HG033-L1_R2.fq.gz
**HG033-L2**	PRJEB52013	Illumina MiSeq	METAGENOMIC	PCR	AMPLICON	PAIRED	HG033-L2_R1.fq.gz	HG033-L2_R2.fq.gz
**HG033-L3**	PRJEB52013	Illumina MiSeq	METAGENOMIC	PCR	AMPLICON	PAIRED	HG033-L3_R1.fq.gz	HG033-L3_R2.fq.gz
**HG033-O1**	PRJEB52013	Illumina MiSeq	METAGENOMIC	PCR	AMPLICON	PAIRED	HG033-O1_R1.fq.gz	HG033-O1_R2.fq.gz
**HG033-O2**	PRJEB52013	Illumina MiSeq	METAGENOMIC	PCR	AMPLICON	PAIRED	HG033-O2_R1.fq.gz	HG033-O2_R2.fq.gz
**HG033-O3**	PRJEB52013	Illumina MiSeq	METAGENOMIC	PCR	AMPLICON	PAIRED	HG033-O3_R1.fq.gz	HG033-O3_R2.fq.gz
**HG033-Q1**	PRJEB52013	Illumina MiSeq	METAGENOMIC	PCR	AMPLICON	PAIRED	HG033-Q1_R1.fq.gz	HG033-Q1_R2.fq.gz
**HG033-Q2**	PRJEB52013	Illumina MiSeq	METAGENOMIC	PCR	AMPLICON	PAIRED	HG033-Q2_R1.fq.gz	HG033-Q2_R2.fq.gz
**HG033-Q3**	PRJEB52013	Illumina MiSeq	METAGENOMIC	PCR	AMPLICON	PAIRED	HG033-Q3_R1.fq.gz	HG033-Q3_R2.fq.gz
**HG033-R1**	PRJEB52013	Illumina MiSeq	METAGENOMIC	PCR	AMPLICON	PAIRED	HG033-R1_R1.fq.gz	HG033-R1_R2.fq.gz
**HG033-R2**	PRJEB52013	Illumina MiSeq	METAGENOMIC	PCR	AMPLICON	PAIRED	HG033-R2_R1.fq.gz	HG033-R2_R2.fq.gz
**HG033-R3**	PRJEB52013	Illumina MiSeq	METAGENOMIC	PCR	AMPLICON	PAIRED	HG033-R3_R1.fq.gz	HG033-R3_R2.fq.gz
**HG059-B1**	PRJEB52013	Illumina MiSeq	METAGENOMIC	PCR	AMPLICON	PAIRED	HG059-B1_R1.fq.gz	HG059-B1_R2.fq.gz
**HG059-B2**	PRJEB52013	Illumina MiSeq	METAGENOMIC	PCR	AMPLICON	PAIRED	HG059-B2_R1.fq.gz	HG059-B2_R2.fq.gz
**HG059-B3**	PRJEB52013	Illumina MiSeq	METAGENOMIC	PCR	AMPLICON	PAIRED	HG059-B3_R1.fq.gz	HG059-B3_R2.fq.gz
**HG059-D1**	PRJEB52013	Illumina MiSeq	METAGENOMIC	PCR	AMPLICON	PAIRED	HG059-D1_R1.fq.gz	HG059-D1_R2.fq.gz
**HG059-D2**	PRJEB52013	Illumina MiSeq	METAGENOMIC	PCR	AMPLICON	PAIRED	HG059-D2_R1.fq.gz	HG059-D2_R2.fq.gz
**HG059-D3**	PRJEB52013	Illumina MiSeq	METAGENOMIC	PCR	AMPLICON	PAIRED	HG059-D3_R1.fq.gz	HG059-D3_R2.fq.gz
**HG059-I1**	PRJEB52013	Illumina MiSeq	METAGENOMIC	PCR	AMPLICON	PAIRED	HG059-I1_R1.fq.gz	HG059-I1_R2.fq.gz
**HG059-I2**	PRJEB52013	Illumina MiSeq	METAGENOMIC	PCR	AMPLICON	PAIRED	HG059-I2_R1.fq.gz	HG059-I2_R2.fq.gz
**HG059-I3**	PRJEB52013	Illumina MiSeq	METAGENOMIC	PCR	AMPLICON	PAIRED	HG059-I3_R1.fq.gz	HG059-I3_R2.fq.gz
**HG059-J1**	PRJEB52013	Illumina MiSeq	METAGENOMIC	PCR	AMPLICON	PAIRED	HG059-J1_R1.fq.gz	HG059-J1_R2.fq.gz

**HG059-J2**	PRJEB52013	Illumina MiSeq	METAGENOMIC	PCR	AMPLICON	PAIRED	HG059-J2_R1.fq.gz	HG059-J2_R2.fq.gz
**HG059-J3**	PRJEB52013	Illumina MiSeq	METAGENOMIC	PCR	AMPLICON	PAIRED	HG059-J3_R1.fq.gz	HG059-J3_R2.fq.gz
**HG059-L1**	PRJEB52013	Illumina MiSeq	METAGENOMIC	PCR	AMPLICON	PAIRED	HG059-L1_R1.fq.gz	HG059-L1_R2.fq.gz
**HG059-L2**	PRJEB52013	Illumina MiSeq	METAGENOMIC	PCR	AMPLICON	PAIRED	HG059-L2_R1.fq.gz	HG059-L2_R2.fq.gz
**HG059-L3**	PRJEB52013	Illumina MiSeq	METAGENOMIC	PCR	AMPLICON	PAIRED	HG059-L3_R1.fq.gz	HG059-L3_R2.fq.gz
**HG059-O1**	PRJEB52013	Illumina MiSeq	METAGENOMIC	PCR	AMPLICON	PAIRED	HG059-O1_R1.fq.gz	HG059-O1_R2.fq.gz
**HG059-O2**	PRJEB52013	Illumina MiSeq	METAGENOMIC	PCR	AMPLICON	PAIRED	HG059-O2_R1.fq.gz	HG059-O2_R2.fq.gz
**HG059-O3**	PRJEB52013	Illumina MiSeq	METAGENOMIC	PCR	AMPLICON	PAIRED	HG059-O3_R1.fq.gz	HG059-O3_R2.fq.gz
**HG059-Q1**	PRJEB52013	Illumina MiSeq	METAGENOMIC	PCR	AMPLICON	PAIRED	HG059-Q1_R1.fq.gz	HG059-Q1_R2.fq.gz
**HG059-Q2**	PRJEB52013	Illumina MiSeq	METAGENOMIC	PCR	AMPLICON	PAIRED	HG059-Q2_R1.fq.gz	HG059-Q2_R2.fq.gz
**HG059-Q3**	PRJEB52013	Illumina MiSeq	METAGENOMIC	PCR	AMPLICON	PAIRED	HG059-Q3_R1.fq.gz	HG059-Q3_R2.fq.gz
**HG059-R1**	PRJEB52013	Illumina MiSeq	METAGENOMIC	PCR	AMPLICON	PAIRED	HG059-R1_R1.fq.gz	HG059-R1_R2.fq.gz
**HG059-R2**	PRJEB52013	Illumina MiSeq	METAGENOMIC	PCR	AMPLICON	PAIRED	HG059-R2_R1.fq.gz	HG059-R2_R2.fq.gz
**HG059-R3**	PRJEB52013	Illumina MiSeq	METAGENOMIC	PCR	AMPLICON	PAIRED	HG059-R3_R1.fq.gz	HG059-R3_R2.fq.gz
**HG082-B1**	PRJEB52013	Illumina MiSeq	METAGENOMIC	PCR	AMPLICON	PAIRED	HG082-B1_R1.fq.gz	HG082-B1_R2.fq.gz
**HG082-B2**	PRJEB52013	Illumina MiSeq	METAGENOMIC	PCR	AMPLICON	PAIRED	HG082-B2_R1.fq.gz	HG082-B2_R2.fq.gz
**HG082-B3**	PRJEB52013	Illumina MiSeq	METAGENOMIC	PCR	AMPLICON	PAIRED	HG082-B3_R1.fq.gz	HG082-B3_R2.fq.gz
**HG082-D1**	PRJEB52013	Illumina MiSeq	METAGENOMIC	PCR	AMPLICON	PAIRED	HG082-D1_R1.fq.gz	HG082-D1_R2.fq.gz
**HG082-D2**	PRJEB52013	Illumina MiSeq	METAGENOMIC	PCR	AMPLICON	PAIRED	HG082-D2_R1.fq.gz	HG082-D2_R2.fq.gz
**HG082-D3**	PRJEB52013	Illumina MiSeq	METAGENOMIC	PCR	AMPLICON	PAIRED	HG082-D3_R1.fq.gz	HG082-D3_R2.fq.gz
**HG082-I1**	PRJEB52013	Illumina MiSeq	METAGENOMIC	PCR	AMPLICON	PAIRED	HG082-I1_R1.fq.gz	HG082-I1_R2.fq.gz
**HG082-I2**	PRJEB52013	Illumina MiSeq	METAGENOMIC	PCR	AMPLICON	PAIRED	HG082-I2_R1.fq.gz	HG082-I2_R2.fq.gz
**HG082-I3**	PRJEB52013	Illumina MiSeq	METAGENOMIC	PCR	AMPLICON	PAIRED	HG082-I3_R1.fq.gz	HG082-I3_R2.fq.gz
**HG082-J1**	PRJEB52013	Illumina MiSeq	METAGENOMIC	PCR	AMPLICON	PAIRED	HG082-J1_R1.fq.gz	HG082-J1_R2.fq.gz
**HG082-J2**	PRJEB52013	Illumina MiSeq	METAGENOMIC	PCR	AMPLICON	PAIRED	HG082-J2_R1.fq.gz	HG082-J2_R2.fq.gz
**HG082-J3**	PRJEB52013	Illumina MiSeq	METAGENOMIC	PCR	AMPLICON	PAIRED	HG082-J3_R1.fq.gz	HG082-J3_R2.fq.gz
**HG082-L1**	PRJEB52013	Illumina MiSeq	METAGENOMIC	PCR	AMPLICON	PAIRED	HG082-L1_R1.fq.gz	HG082-L1_R2.fq.gz
**HG082-L2**	PRJEB52013	Illumina MiSeq	METAGENOMIC	PCR	AMPLICON	PAIRED	HG082-L2_R1.fq.gz	HG082-L2_R2.fq.gz
**HG082-L3**	PRJEB52013	Illumina MiSeq	METAGENOMIC	PCR	AMPLICON	PAIRED	HG082-L3_R1.fq.gz	HG082-L3_R2.fq.gz
**HG082-O1**	PRJEB52013	Illumina MiSeq	METAGENOMIC	PCR	AMPLICON	PAIRED	HG082-O1_R1.fq.gz	HG082-O1_R2.fq.gz
**HG082-O2**	PRJEB52013	Illumina MiSeq	METAGENOMIC	PCR	AMPLICON	PAIRED	HG082-O2_R1.fq.gz	HG082-O2_R2.fq.gz
**HG082-O3**	PRJEB52013	Illumina MiSeq	METAGENOMIC	PCR	AMPLICON	PAIRED	HG082-O3_R1.fq.gz	HG082-O3_R2.fq.gz
**HG082-Q1**	PRJEB52013	Illumina MiSeq	METAGENOMIC	PCR	AMPLICON	PAIRED	HG082-Q1_R1.fq.gz	HG082-Q1_R2.fq.gz
**HG082-Q2**	PRJEB52013	Illumina MiSeq	METAGENOMIC	PCR	AMPLICON	PAIRED	HG082-Q2_R1.fq.gz	HG082-Q2_R2.fq.gz
**HG082-Q3**	PRJEB52013	Illumina MiSeq	METAGENOMIC	PCR	AMPLICON	PAIRED	HG082-Q3_R1.fq.gz	HG082-Q3_R2.fq.gz
**HG082-R1**	PRJEB52013	Illumina MiSeq	METAGENOMIC	PCR	AMPLICON	PAIRED	HG082-R1_R1.fq.gz	HG082-R1_R2.fq.gz
**HG082-R2**	PRJEB52013	Illumina MiSeq	METAGENOMIC	PCR	AMPLICON	PAIRED	HG082-R2_R1.fq.gz	HG082-R2_R2.fq.gz
**HG082-R3**	PRJEB52013	Illumina MiSeq	METAGENOMIC	PCR	AMPLICON	PAIRED	HG082-R3_R1.fq.gz	HG082-R3_R2.fq.gz
**HG091-B1**	PRJEB52013	Illumina MiSeq	METAGENOMIC	PCR	AMPLICON	PAIRED	HG091-B1_R1.fq.gz	HG091-B1_R2.fq.gz
**HG091-B2**	PRJEB52013	Illumina MiSeq	METAGENOMIC	PCR	AMPLICON	PAIRED	HG091-B2_R1.fq.gz	HG091-B2_R2.fq.gz
**HG091-B3**	PRJEB52013	Illumina MiSeq	METAGENOMIC	PCR	AMPLICON	PAIRED	HG091-B3_R1.fq.gz	HG091-B3_R2.fq.gz
**HG091-D1**	PRJEB52013	Illumina MiSeq	METAGENOMIC	PCR	AMPLICON	PAIRED	HG091-D1_R1.fq.gz	HG091-D1_R2.fq.gz
**HG091-D2**	PRJEB52013	Illumina MiSeq	METAGENOMIC	PCR	AMPLICON	PAIRED	HG091-D2_R1.fq.gz	HG091-D2_R2.fq.gz
**HG091-D3**	PRJEB52013	Illumina MiSeq	METAGENOMIC	PCR	AMPLICON	PAIRED	HG091-D3_R1.fq.gz	HG091-D3_R2.fq.gz
**HG091-I1**	PRJEB52013	Illumina MiSeq	METAGENOMIC	PCR	AMPLICON	PAIRED	HG091-I1_R1.fq.gz	HG091-I1_R2.fq.gz
**HG091-I2**	PRJEB52013	Illumina MiSeq	METAGENOMIC	PCR	AMPLICON	PAIRED	HG091-I2_R1.fq.gz	HG091-I2_R2.fq.gz
**HG091-I3**	PRJEB52013	Illumina MiSeq	METAGENOMIC	PCR	AMPLICON	PAIRED	HG091-I3_R1.fq.gz	HG091-I3_R2.fq.gz
**HG091-J1**	PRJEB52013	Illumina MiSeq	METAGENOMIC	PCR	AMPLICON	PAIRED	HG091-J1_R1.fq.gz	HG091-J1_R2.fq.gz
**HG091-J2**	PRJEB52013	Illumina MiSeq	METAGENOMIC	PCR	AMPLICON	PAIRED	HG091-J2_R1.fq.gz	HG091-J2_R2.fq.gz
**HG091-J3**	PRJEB52013	Illumina MiSeq	METAGENOMIC	PCR	AMPLICON	PAIRED	HG091-J3_R1.fq.gz	HG091-J3_R2.fq.gz
**HG091-L1**	PRJEB52013	Illumina MiSeq	METAGENOMIC	PCR	AMPLICON	PAIRED	HG091-L1_R1.fq.gz	HG091-L1_R2.fq.gz
**HG091-L2**	PRJEB52013	Illumina MiSeq	METAGENOMIC	PCR	AMPLICON	PAIRED	HG091-L2_R1.fq.gz	HG091-L2_R2.fq.gz
**HG091-L3**	PRJEB52013	Illumina MiSeq	METAGENOMIC	PCR	AMPLICON	PAIRED	HG091-L3_R1.fq.gz	HG091-L3_R2.fq.gz
**HG091-O1**	PRJEB52013	Illumina MiSeq	METAGENOMIC	PCR	AMPLICON	PAIRED	HG091-O1_R1.fq.gz	HG091-O1_R2.fq.gz
**HG091-O2**	PRJEB52013	Illumina MiSeq	METAGENOMIC	PCR	AMPLICON	PAIRED	HG091-O2_R1.fq.gz	HG091-O2_R2.fq.gz
**HG091-O3**	PRJEB52013	Illumina MiSeq	METAGENOMIC	PCR	AMPLICON	PAIRED	HG091-O3_R1.fq.gz	HG091-O3_R2.fq.gz
**HG091-Q1**	PRJEB52013	Illumina MiSeq	METAGENOMIC	PCR	AMPLICON	PAIRED	HG091-Q1_R1.fq.gz	HG091-Q1_R2.fq.gz
**HG091-Q2**	PRJEB52013	Illumina MiSeq	METAGENOMIC	PCR	AMPLICON	PAIRED	HG091-Q2_R1.fq.gz	HG091-Q2_R2.fq.gz
**HG091-Q3**	PRJEB52013	Illumina MiSeq	METAGENOMIC	PCR	AMPLICON	PAIRED	HG091-Q3_R1.fq.gz	HG091-Q3_R2.fq.gz

**HG091-R1**	PRJEB52013	Illumina MiSeq	METAGENOMIC	PCR	AMPLICON	PAIRED	HG091-R1_R1.fq.gz	HG091-R1_R2.fq.gz
**HG091-R2**	PRJEB52013	Illumina MiSeq	METAGENOMIC	PCR	AMPLICON	PAIRED	HG091-R2_R1.fq.gz	HG091-R2_R2.fq.gz
**HG091-R3**	PRJEB52013	Illumina MiSeq	METAGENOMIC	PCR	AMPLICON	PAIRED	HG091-R3_R1.fq.gz	HG091-R3_R2.fq.gz
**HG141-B1**	PRJEB52013	Illumina MiSeq	METAGENOMIC	PCR	AMPLICON	PAIRED	HG141-B1_R1.fq.gz	HG141-B1_R2.fq.gz
**HG141-B2**	PRJEB52013	Illumina MiSeq	METAGENOMIC	PCR	AMPLICON	PAIRED	HG141-B2_R1.fq.gz	HG141-B2_R2.fq.gz
**HG141-B3**	PRJEB52013	Illumina MiSeq	METAGENOMIC	PCR	AMPLICON	PAIRED	HG141-B3_R1.fq.gz	HG141-B3_R2.fq.gz
**HG141-D1**	PRJEB52013	Illumina MiSeq	METAGENOMIC	PCR	AMPLICON	PAIRED	HG141-D1_R1.fq.gz	HG141-D1_R2.fq.gz
**HG141-D2**	PRJEB52013	Illumina MiSeq	METAGENOMIC	PCR	AMPLICON	PAIRED	HG141-D2_R1.fq.gz	HG141-D2_R2.fq.gz
**HG141-D3**	PRJEB52013	Illumina MiSeq	METAGENOMIC	PCR	AMPLICON	PAIRED	HG141-D3_R1.fq.gz	HG141-D3_R2.fq.gz
**HG141-I1**	PRJEB52013	Illumina MiSeq	METAGENOMIC	PCR	AMPLICON	PAIRED	HG141-I1_R1.fq.gz	HG141-I1_R2.fq.gz
**HG141-I2**	PRJEB52013	Illumina MiSeq	METAGENOMIC	PCR	AMPLICON	PAIRED	HG141-I2_R1.fq.gz	HG141-I2_R2.fq.gz
**HG141-I3**	PRJEB52013	Illumina MiSeq	METAGENOMIC	PCR	AMPLICON	PAIRED	HG141-I3_R1.fq.gz	HG141-I3_R2.fq.gz
**HG141-J1**	PRJEB52013	Illumina MiSeq	METAGENOMIC	PCR	AMPLICON	PAIRED	HG141-J1_R1.fq.gz	HG141-J1_R2.fq.gz
**HG141-J2**	PRJEB52013	Illumina MiSeq	METAGENOMIC	PCR	AMPLICON	PAIRED	HG141-J2_R1.fq.gz	HG141-J2_R2.fq.gz
**HG141-J3**	PRJEB52013	Illumina MiSeq	METAGENOMIC	PCR	AMPLICON	PAIRED	HG141-J3_R1.fq.gz	HG141-J3_R2.fq.gz
**HG141-L1**	PRJEB52013	Illumina MiSeq	METAGENOMIC	PCR	AMPLICON	PAIRED	HG141-L1_R1.fq.gz	HG141-L1_R2.fq.gz
**HG141-L2**	PRJEB52013	Illumina MiSeq	METAGENOMIC	PCR	AMPLICON	PAIRED	HG141-L2_R1.fq.gz	HG141-L2_R2.fq.gz
**HG141-L3**	PRJEB52013	Illumina MiSeq	METAGENOMIC	PCR	AMPLICON	PAIRED	HG141-L3_R1.fq.gz	HG141-L3_R2.fq.gz
**HG141-O1**	PRJEB52013	Illumina MiSeq	METAGENOMIC	PCR	AMPLICON	PAIRED	HG141-O1_R1.fq.gz	HG141-O1_R2.fq.gz
**HG141-O2**	PRJEB52013	Illumina MiSeq	METAGENOMIC	PCR	AMPLICON	PAIRED	HG141-O2_R1.fq.gz	HG141-O2_R2.fq.gz
**HG141-O3**	PRJEB52013	Illumina MiSeq	METAGENOMIC	PCR	AMPLICON	PAIRED	HG141-O3_R1.fq.gz	HG141-O3_R2.fq.gz
**HG141-Q1**	PRJEB52013	Illumina MiSeq	METAGENOMIC	PCR	AMPLICON	PAIRED	HG141-Q1_R1.fq.gz	HG141-Q1_R2.fq.gz
**HG141-Q2**	PRJEB52013	Illumina MiSeq	METAGENOMIC	PCR	AMPLICON	PAIRED	HG141-Q2_R1.fq.gz	HG141-Q2_R2.fq.gz
**HG141-Q3**	PRJEB52013	Illumina MiSeq	METAGENOMIC	PCR	AMPLICON	PAIRED	HG141-Q3_R1.fq.gz	HG141-Q3_R2.fq.gz
**HG141-R1**	PRJEB52013	Illumina MiSeq	METAGENOMIC	PCR	AMPLICON	PAIRED	HG141-R1_R1.fq.gz	HG141-R1_R2.fq.gz
**HG141-R2**	PRJEB52013	Illumina MiSeq	METAGENOMIC	PCR	AMPLICON	PAIRED	HG141-R2_R1.fq.gz	HG141-R2_R2.fq.gz
**HG141-R3**	PRJEB52013	Illumina MiSeq	METAGENOMIC	PCR	AMPLICON	PAIRED	HG141-R3_R1.fq.gz	HG141-R3_R2.fq.gz
**HG225-B1**	PRJEB52013	Illumina MiSeq	METAGENOMIC	PCR	AMPLICON	PAIRED	HG225-B1_R1.fq.gz	HG225-B1_R2.fq.gz
**HG225-B2**	PRJEB52013	Illumina MiSeq	METAGENOMIC	PCR	AMPLICON	PAIRED	HG225-B2_R1.fq.gz	HG225-B2_R2.fq.gz
**HG225-B3**	PRJEB52013	Illumina MiSeq	METAGENOMIC	PCR	AMPLICON	PAIRED	HG225-B3_R1.fq.gz	HG225-B3_R2.fq.gz
**HG225-D1**	PRJEB52013	Illumina MiSeq	METAGENOMIC	PCR	AMPLICON	PAIRED	HG225-D1_R1.fq.gz	HG225-D1_R2.fq.gz
**HG225-D2**	PRJEB52013	Illumina MiSeq	METAGENOMIC	PCR	AMPLICON	PAIRED	HG225-D2_R1.fq.gz	HG225-D2_R2.fq.gz
**HG225-D3**	PRJEB52013	Illumina MiSeq	METAGENOMIC	PCR	AMPLICON	PAIRED	HG225-D3_R1.fq.gz	HG225-D3_R2.fq.gz
**HG225-I1**	PRJEB52013	Illumina MiSeq	METAGENOMIC	PCR	AMPLICON	PAIRED	HG225-I1_R1.fq.gz	HG225-I1_R2.fq.gz
**HG225-I2**	PRJEB52013	Illumina MiSeq	METAGENOMIC	PCR	AMPLICON	PAIRED	HG225-I2_R1.fq.gz	HG225-I2_R2.fq.gz
**HG225-I3**	PRJEB52013	Illumina MiSeq	METAGENOMIC	PCR	AMPLICON	PAIRED	HG225-I3_R1.fq.gz	HG225-I3_R2.fq.gz
**HG225-J1**	PRJEB52013	Illumina MiSeq	METAGENOMIC	PCR	AMPLICON	PAIRED	HG225-J1_R1.fq.gz	HG225-J1_R2.fq.gz
**HG225-J2**	PRJEB52013	Illumina MiSeq	METAGENOMIC	PCR	AMPLICON	PAIRED	HG225-J2_R1.fq.gz	HG225-J2_R2.fq.gz
**HG225-J3**	PRJEB52013	Illumina MiSeq	METAGENOMIC	PCR	AMPLICON	PAIRED	HG225-J3_R1.fq.gz	HG225-J3_R2.fq.gz
**HG225-L1**	PRJEB52013	Illumina MiSeq	METAGENOMIC	PCR	AMPLICON	PAIRED	HG225-L1_R1.fq.gz	HG225-L1_R2.fq.gz
**HG225-L2**	PRJEB52013	Illumina MiSeq	METAGENOMIC	PCR	AMPLICON	PAIRED	HG225-L2_R1.fq.gz	HG225-L2_R2.fq.gz
**HG225-L3**	PRJEB52013	Illumina MiSeq	METAGENOMIC	PCR	AMPLICON	PAIRED	HG225-L3_R1.fq.gz	HG225-L3_R2.fq.gz
**HG225-O1**	PRJEB52013	Illumina MiSeq	METAGENOMIC	PCR	AMPLICON	PAIRED	HG225-O1_R1.fq.gz	HG225-O1_R2.fq.gz
**HG225-O2**	PRJEB52013	Illumina MiSeq	METAGENOMIC	PCR	AMPLICON	PAIRED	HG225-O2_R1.fq.gz	HG225-O2_R2.fq.gz
**HG225-O3**	PRJEB52013	Illumina MiSeq	METAGENOMIC	PCR	AMPLICON	PAIRED	HG225-O3_R1.fq.gz	HG225-O3_R2.fq.gz
**HG225-Q1**	PRJEB52013	Illumina MiSeq	METAGENOMIC	PCR	AMPLICON	PAIRED	HG225-Q1_R1.fq.gz	HG225-Q1_R2.fq.gz
**HG225-Q2**	PRJEB52013	Illumina MiSeq	METAGENOMIC	PCR	AMPLICON	PAIRED	HG225-Q2_R1.fq.gz	HG225-Q2_R2.fq.gz
**HG225-Q3**	PRJEB52013	Illumina MiSeq	METAGENOMIC	PCR	AMPLICON	PAIRED	HG225-Q3_R1.fq.gz	HG225-Q3_R2.fq.gz
**HG225-R1**	PRJEB52013	Illumina MiSeq	METAGENOMIC	PCR	AMPLICON	PAIRED	HG225-R1_R1.fq.gz	HG225-R1_R2.fq.gz
**HG225-R2**	PRJEB52013	Illumina MiSeq	METAGENOMIC	PCR	AMPLICON	PAIRED	HG225-R2_R1.fq.gz	HG225-R2_R2.fq.gz
**HG225-R3**	PRJEB52013	Illumina MiSeq	METAGENOMIC	PCR	AMPLICON	PAIRED	HG225-R3_R1.fq.gz	HG225-R3_R2.fq.gz
**HG319-B1**	PRJEB52013	Illumina MiSeq	METAGENOMIC	PCR	AMPLICON	PAIRED	HG319-B1_R1.fq.gz	HG319-B1_R2.fq.gz
**HG319-B2**	PRJEB52013	Illumina MiSeq	METAGENOMIC	PCR	AMPLICON	PAIRED	HG319-B2_R1.fq.gz	HG319-B2_R2.fq.gz
**HG319-B3**	PRJEB52013	Illumina MiSeq	METAGENOMIC	PCR	AMPLICON	PAIRED	HG319-B3_R1.fq.gz	HG319-B3_R2.fq.gz
**HG319-D1**	PRJEB52013	Illumina MiSeq	METAGENOMIC	PCR	AMPLICON	PAIRED	HG319-D1_R1.fq.gz	HG319-D1_R2.fq.gz
**HG319-D2**	PRJEB52013	Illumina MiSeq	METAGENOMIC	PCR	AMPLICON	PAIRED	HG319-D2_R1.fq.gz	HG319-D2_R2.fq.gz
**HG319-D3**	PRJEB52013	Illumina MiSeq	METAGENOMIC	PCR	AMPLICON	PAIRED	HG319-D3_R1.fq.gz	HG319-D3_R2.fq.gz
**HG319-I1**	PRJEB52013	Illumina MiSeq	METAGENOMIC	PCR	AMPLICON	PAIRED	HG319-I1_R1.fq.gz	HG319-I1_R2.fq.gz
**HG319-I2**	PRJEB52013	Illumina MiSeq	METAGENOMIC	PCR	AMPLICON	PAIRED	HG319-I2_R1.fq.gz	HG319-I2_R2.fq.gz

**HG319-I3**	PRJEB52013	Illumina MiSeq	METAGENOMIC	PCR	AMPLICON	PAIRED	HG319-I3_R1.fq.gz	HG319-I3_R2.fq.gz
**HG319-J1**	PRJEB52013	Illumina MiSeq	METAGENOMIC	PCR	AMPLICON	PAIRED	HG319-J1_R1.fq.gz	HG319-J1_R2.fq.gz
**HG319-J2**	PRJEB52013	Illumina MiSeq	METAGENOMIC	PCR	AMPLICON	PAIRED	HG319-J2_R1.fq.gz	HG319-J2_R2.fq.gz
**HG319-J3**	PRJEB52013	Illumina MiSeq	METAGENOMIC	PCR	AMPLICON	PAIRED	HG319-J3_R1.fq.gz	HG319-J3_R2.fq.gz
**HG319-L1**	PRJEB52013	Illumina MiSeq	METAGENOMIC	PCR	AMPLICON	PAIRED	HG319-L1_R1.fq.gz	HG319-L1_R2.fq.gz
**HG319-L2**	PRJEB52013	Illumina MiSeq	METAGENOMIC	PCR	AMPLICON	PAIRED	HG319-L2_R1.fq.gz	HG319-L2_R2.fq.gz
**HG319-L3**	PRJEB52013	Illumina MiSeq	METAGENOMIC	PCR	AMPLICON	PAIRED	HG319-L3_R1.fq.gz	HG319-L3_R2.fq.gz
**HG319-O1**	PRJEB52013	Illumina MiSeq	METAGENOMIC	PCR	AMPLICON	PAIRED	HG319-O1_R1.fq.gz	HG319-O1_R2.fq.gz
**HG319-O2**	PRJEB52013	Illumina MiSeq	METAGENOMIC	PCR	AMPLICON	PAIRED	HG319-O2_R1.fq.gz	HG319-O2_R2.fq.gz
**HG319-O3**	PRJEB52013	Illumina MiSeq	METAGENOMIC	PCR	AMPLICON	PAIRED	HG319-O3_R1.fq.gz	HG319-O3_R2.fq.gz
**HG319-Q2**	PRJEB52013	Illumina MiSeq	METAGENOMIC	PCR	AMPLICON	PAIRED	HG319-Q2_R1.fq.gz	HG319-Q2_R2.fq.gz
**HG319-Q3**	PRJEB52013	Illumina MiSeq	METAGENOMIC	PCR	AMPLICON	PAIRED	HG319-Q3_R1.fq.gz	HG319-Q3_R2.fq.gz
**HG319-R1**	PRJEB52013	Illumina MiSeq	METAGENOMIC	PCR	AMPLICON	PAIRED	HG319-R1_R1.fq.gz	HG319-R1_R2.fq.gz
**HG319-R2**	PRJEB52013	Illumina MiSeq	METAGENOMIC	PCR	AMPLICON	PAIRED	HG319-R2_R1.fq.gz	HG319-R2_R2.fq.gz
**HG319-R3**	PRJEB52013	Illumina MiSeq	METAGENOMIC	PCR	AMPLICON	PAIRED	HG319-R3_R1.fq.gz	HG319-R3_R2.fq.gz
**HGnegA**	PRJEB52013	Illumina MiSeq	METAGENOMIC	PCR	AMPLICON	PAIRED	HGnegA_R1.fq.gz	HGnegA_R2.fq.gz
