# Supplementary material for: Genetic variation and microbiota in bumble bees cross-infected by different strains of C. bombi
Source: PLoS One. 2022 Nov 28;17(11):e0277041. doi: 10.1371/journal.pone.0277041 (PMC9704641; doi:10.1371/journal.pone.0277041)
Supplement: S12 File — File contains several sections. Methods: assessing infection intensity, selecting the 8x8 sub-matrix. Basic data: explanations, lists of colonies and parasite strains. Variation of infection intensity across colonies and strains. Explanations and supporting results for data in Appendices A to E. (PDF) [file pone.0277041.s012.pdf]

Supporting Information
Explanations

Overview

Methods

Selecting the 8 x 8matrix

Quantitative qPCR

Basic data

Appendix A: Documentation and statistics for the 'low-coverage' study.
This refers to using the full data set, based on the 20 x 20 matrix (Colonies x Strains). 'Low coverage' means that every individual (genome) was sequenced to a coverage of around 2-3x.

Appendix B: Documentation and statistics for the 'high-coverage' study.
This refers to using the restricted data set, based on the 8 x 8 matrix (Colonies x Strains) subset, chosen from the full matrix above.
'High coverage' means that every individual (genome) was sequenced to a coverage of around 20-30x.

Appendix C: Documentation and statistics for the analyses of the microbiota. This was done for the 8 x 8 sub-matrix used in the high-coverage study (Appendix B). All bees involved in this set were analysed for their bacterial metagenome.

Appendix D: Documentation and statistics for the analyses of the microbiota separated by groups. This was done for the 8 x 8 sub-matrix used in the high-coverage study (Appendix B). All bees involved in this set were analysed for their bacterial metagenome, ordered by MDS for their taxonomic composition, and separated by the Axis.1-value into 'resistant' (Axis.1 ≥ 0) and 'permissive' (Axis.1 < 0) .

Appendix E: Documentation and statistics for the analyses of different sizes of the interaction matrix(from 2 x 2 to 20 x 20). The analyses refer to the infection phenotype, i.e. infection intensity per bee, 7 d post-exposure.

Methods

Standards for assessing infection intensity (using qPCR)

Lab-IDs of *C. bombi* clones: 08. 068, 08.075, 08.076, 10.175, 10.208, 10.218, 10.374, 10.361, 10.441

Of each clone, 100 µl were taken and mixed for a reference sample. Counting was with an automated cell counter and resulted in 1.61 x10^7 cells/ml of solution.

We extracted 10^6 cells (equivalent to 62.1 µl) for tests with the Lysis Reagent Method. For this purpose, cells were centrifuged at 2,500 rpm for 10 min and the supernatant discarded. Then 50 µl of 1x PBS-solution was added, and again centrifuged at 2'500 rpm for 10 min with the supernatant discarded.

For the measurement of infection intensities in the reference samples, Eppendorf tubes were prepared as in the following table:

MM	1x	2x
DirectPCR Lysis Reagent (Cell)	0.1 ml	50 ul
ddH2O	0.9 ml	450 ul
Proteinase K (20mg/ml)	0.01 ml (10 ul)	5 ul

We then added 150 µl of *C. bombi* mixture (equivalent to 10^6 cells) to each of the Eppendorf tubes. These were incubated at 55˚C, centrifuged at 300 rpm for 45 min, followed by incubation at 85˚C for 40 min.

The mixture was diluted several times (at a ratio of 1:3 in each step) to generate the following reference standards. Starting from 'Standard 1', at each step, 6 µl of solution from the previous level was diluted with 12 µl of distilled H_20 to yield the next standard:

Reference	Estimated number of cells
Standard 1	6,666.66
Standard 2	2,222.22
Standard 3	740.74
Standard 4	246.91
Standard 5	82.304
Standard 6	27.434
Standard 7	9.1449

These standards were then subject to qPCR analyses to establish the relationship of a qPCR-signal with the underlying cell concentration in the probe. The mixture for the qPCR was as follows, with 8µl Mixture and 2 µl cDNA per well:

MM	1x [ul]	106 [ul]
EvaGreen	2	212.0
Primer Cri RT f [10uM]	0.2	21.2
Primer Cri RT r [10uM]	0.2	21.2
H2O	5.6	593.6

Selecting the 8 x 8 (sub-) matrix

There are a total of 125,970 possible combinations of colonies and parasite strains when selecting a 8 x 8 matrix out of n = 20 colonies and strains (the 20 x 20 matrix). Of those, a random sample of 100,000 (sub-) matrices were statistically tested for the significance of the main (colony, strain) and interaction term (colony x strain), using transformed values, $x' = log(1 + x)$, for infection intensity (our phenotype) to normalize variances. The interaction term was of special interest, as it was the initial aim of the study to focus on the genetic bases of this interaction. Each such combination of colony and strain represents an 'envelope'.

We considered further those envelopes containing at least 191 bees and having a significance level for the colony x strain interaction term of $P_{crit} = 10^{-6}$ or less, which reduced the available set to 22 envelopes. Among those, we visually inspected the interaction plots. The final choice was defined by the plots having small error bars, large differences among blocks (i.e., colonies), but some variation within blocks, and a minimum of zero infection cases. Envelope no. 29133 was the best fit for these criteria and therefore chose; the interaction plot is shown in Fig 1 of main text.

Basic data

File: '*S1-BasicData*' (.xlsx)

The file contains data from all of the N = 1,200 bees that were tested.

Variables are:

- *Sample_Name*: Lab-code for the sample (the bee), composed from colony no. (015, ..., 319), infecting strain (A, ..., T) and replicate number (1, ..., 3) (three bees were tested for each combination).
- *Colony*: Lab-code for colony identity (as a number, see Table S1).
- *Strain_ID*: Lab-code for strain identity (as a letter, see Table S1).
- *strain:* The standard code for the strain. For matching with *Strain_ID*, see Table S2.
- *Quantitative_Mean*: Infection intensity (cells/bee) as mean of replicated measures per bee.
- *Quantitative_SD*: Standard deviation of infection intensity from replicated measures per bee.
- *infection_date*: Date (day, month) of exposure to the infective dose (year 2014), see also Table S1.
- *Wing_unit*: Size of radial cell in right forewing, as seen in scale of microscope ocular.
- *Wing_mm*: Absolute size of radial cell in right forewing (mm), adjusted for magnification.

Table S1. List of colonies[1].

Code used in this paper	Lab-code[2] (Queen code)	Exposure dates 2014 (Replicate 1, 2, 3)
15	14.015	May 16, 19, 23
20	14.020	May 25, 27, 29
25	14.025	May 21, 27, 29
33	14.033	May 16, 19, 23
39	14.039	May 16, 23, 27
47	14.047	May 29; June 2, 4
59	14.059	May 29; June 2, 4
81	14.081	May 29; June2, 2
82	14.082	May 16, 23, 27
90	14.090	June 4, 4, 6
91	14.091	May 19, 19, 23
124	14.124	May 16, 23 27
141	14.141	June 4, 6, 6
152	14.152	May 29; June 2, 2
183	14.183	June 4, 4, 6
194	14.194	May 21, 23, 27
225	14.225	May 19, 21, 23
269	14.269	May 21, 23, 27
319	14.319	May 29; June 2, 4

[1] All colonies are derived from wild-caught queens, sampled in spring 2014, and raised in the lab. Absence of *Crithidia*-infections was tested with a general 16S rRNA-primer.
[2] Standard-code refers to the internal reference in our long-term studies and is consistent among different studies.

Table S2. List of strains[1].

Code used in this paper	Lab-code[2]
A	08.068
B	08.075
C	08.076
D	08.091
E	08.161
F	10.027
G	10.132
H	10.175
I	10.290
J	10.486
K	12.246
L	12.248
M	12.444
N	12.448
O	12.450
P	14.065
Q	14.149
R	14.172
S	14.255
T	14.338

1 All strains from lab-cultured clones, initially extracted from wild bees.
2 Lab-code refers to the internal reference in our long-term studies and is consistent among different publications.

Overview infection intensities

Our phenotype was infection intensity, measured as number of parasite cells per bee. Figure S1 shows the variation in infection intensity over all colonies or parasite strains respectively.

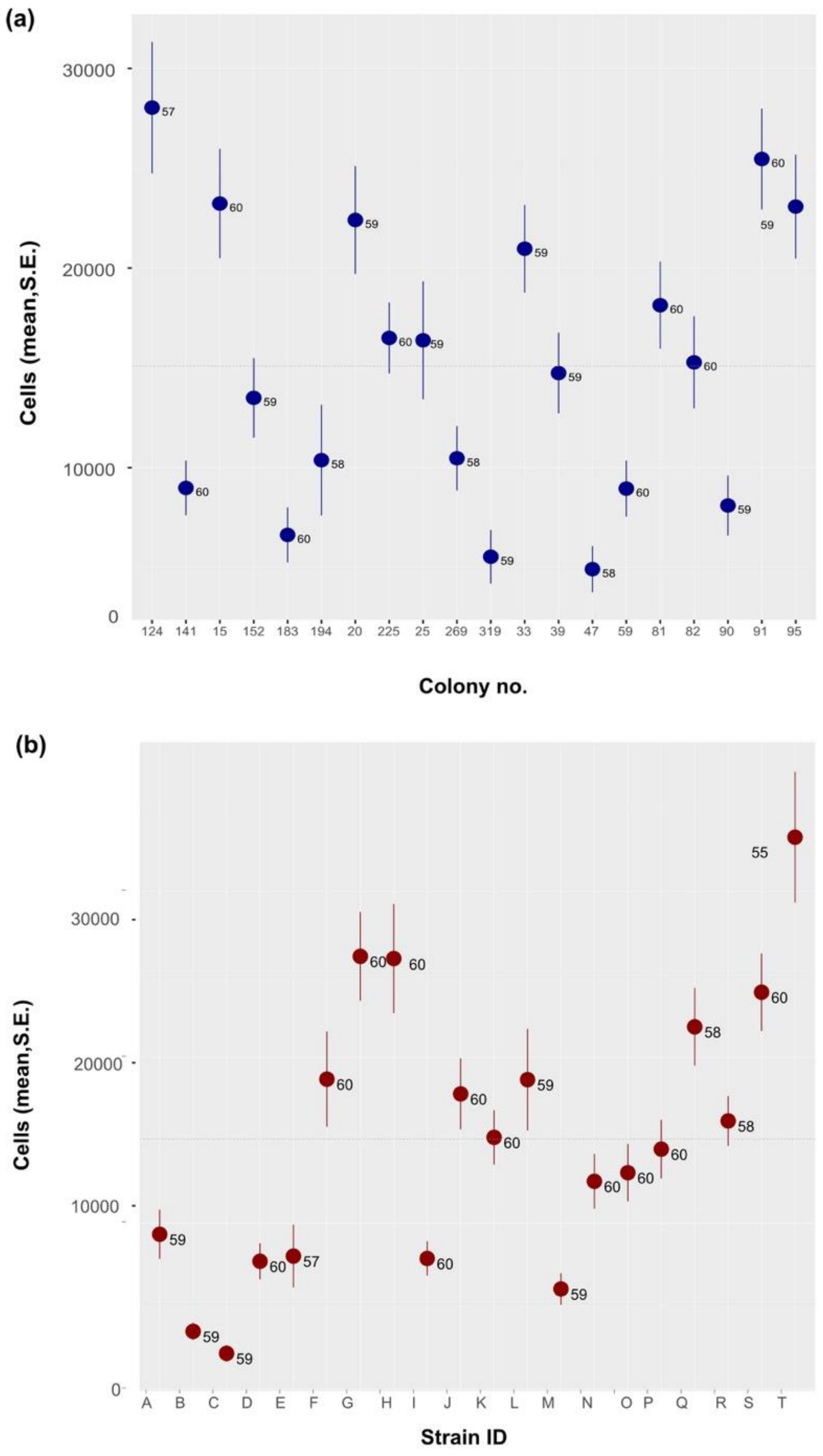

Fig S1. Infection intensity (cells per bee; mean, S.E.) per bee (a) over all colonies and **(b)** over all strains. Small figures refer to sample sizes.

Appendix A:

Documentation and statistics for the low coverage study (20 x 20 matrix)

Data files:

File: '*S2-TopSNP_LowCoverage*' (.xlsx) File of: 14 Dec 2021

The file contains the top 20 SNPs associated with infection intensity in the 20 x 20 matrix.

Variables are:

- *Location:* SNP-code taken from Ensemble database.
- *Major, Minor:* major and minor allele in this SNP.
- *Frequency:* The minor allele frequency
- *N*: Number of individuals.
- *LRT*: Likelihood ratio statistics.
- *high WT/HE/HO*: Number of sites with a WT/HE/HO genotype posterior probability above 0.9.
- *id*: Internal index.
- *P*: P-value-
- *X1:* Chromosome of *B. terrestris* (B01, ..., B18).
- *X2:* Genomic position within the assembled chromosome.
- *X5:* Genomic position where annotated gene starts.
- *X6:* Genomic position where annotated gene ends.
- *X7:* Gene number in data base.
- *X10:* Algorithm used for annotation.
- *X11:* Characterisation of the region.
- *X13:* Hit in data base.

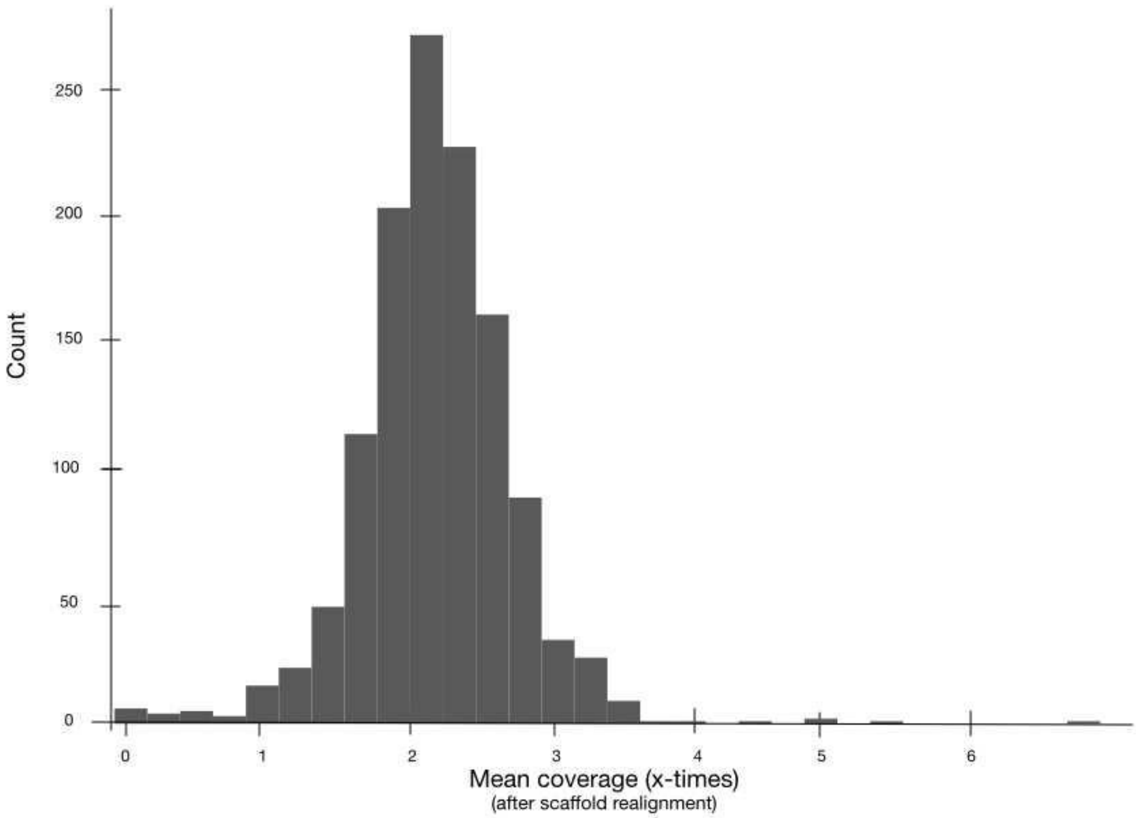

Fig A1. Mean coverage in low-coverage study (20 x 20 matrix). Estimates are for the realigned scaffolds. Mean coverage is c. 2.1 x.

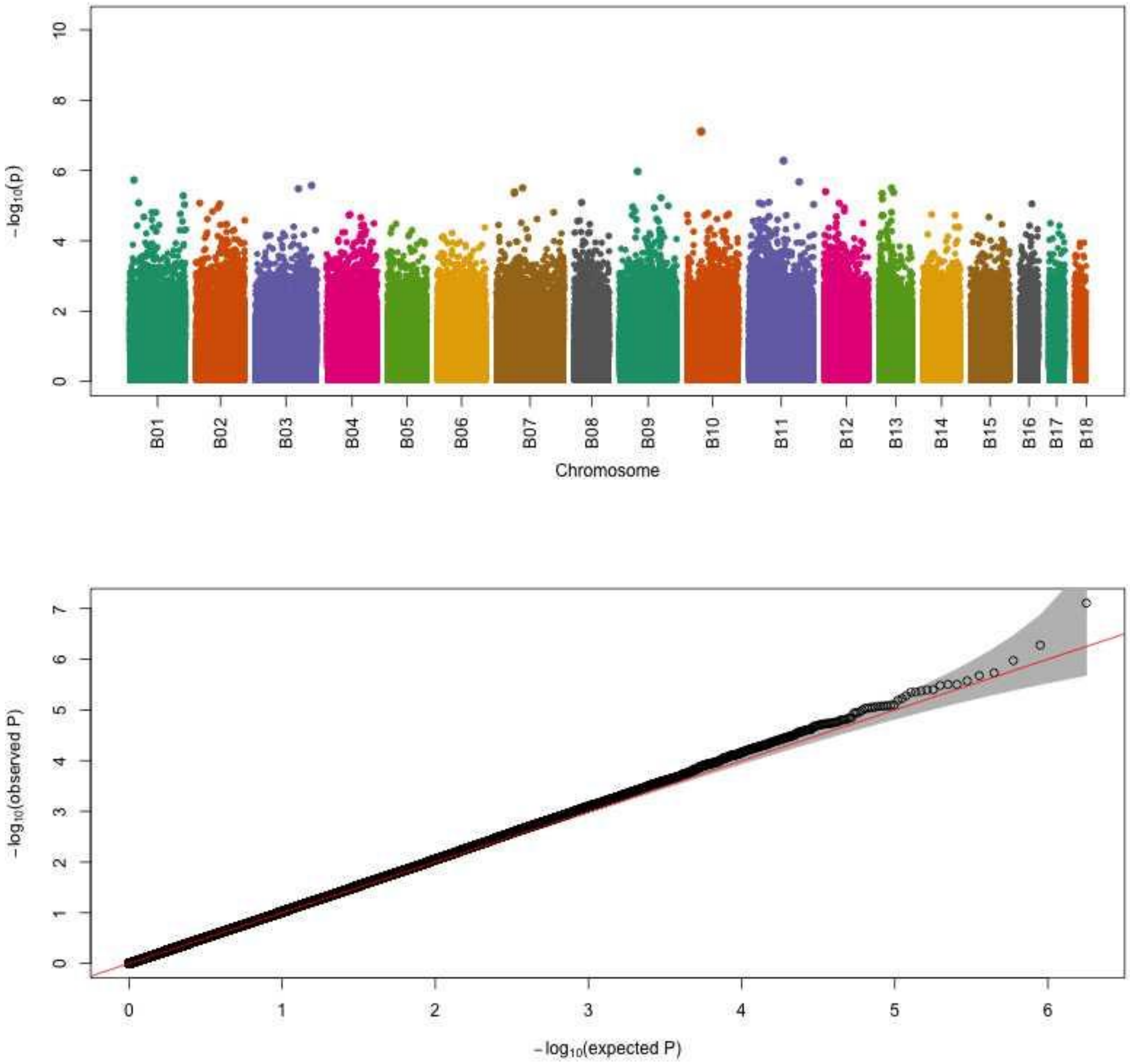

Fig A2. **Low-coverage study (20 x 20 matrix)**. Data as described in text. *Top panel:* Probability (*p*) of association with infection intensity for SNPs along the genome (with chromosome B01 to B18 in different colours). No SNP reached the genome-wide threshold (*P*= 0.1, after Bonferroni correction). *Bottom pane*l: comparing expected to observed probabilities. The calculations used sqrt-transformed infection intensities, using 19 covariates.

Appendix B:

Documentation and statistics for the high coverage study (8 x 8 matrix)

Data files

File: '*S3-TopSNP_HighCoverage*' (.xlsx) File of: 13 Dec 2021

The file contains the top 20 SNPs associated with infection intensity in the 8 x 8 matrix, using sqrt-transformed phenotype data, corrected for the strain effect, and pruned from linkage disequilibria *LD* > 0.75.

Variables are:
- *Location:* SNP-code taken from Ensemble database.
- *ps:* Genomic position of SNP.
-*n_miss:*number of missing alleles.
- *allele1:* Majority allele at this SNP.
- *allele0:* Minority allele at this SNP.
- *af:* Allele frequency.
- *beta:* Association coefficient.
- *se:* S.E. for estimate of *beta.*
- *log1_H1:* value for alternative hypothesis.
- *l_remle:* Likelihood of association using remle.
- *l_mle:* Likelihood of association using mle.
- *p_wald:* Genome-wide significance (probability) using Wald-statistic.
- *p_lrt:* Genome-wide significance based on likelihood.
- *p_score:* Score test statistics.
- *LG:* Chromosome (linkage group).
- *Annotation:* Annotation in Ensemble (assembly 'Bter_1.0').

Note that the *p*-values listed in these files are not corrected for multiple testing. The corrected value with the Bonferroni method is $P = p/n$, where *n* is the number of multiple tests, i.e. the number of sites (SNPs) in the analysis. In this study, the corresponding genome-wide threshold is set to $P_{crit} = 0.1$. We have used *p_lrt* for the plots.

Appendix C:

Microbiota data (8 x 8 matrix)

File: '*S4-DataPrep_Workflow_Report*' (pdf)
The file contains the detailed report on the Amplicon sequencing procedure.

File:'*S5-TopSNP_Mbiota_Ax1*' (.xlsx)
The file contains data from the top 20 SNPs associated with Axis.1 of the Multi-Dimensional Scaling (MDS) of zOTUs. Values for Axis.1 were LD-pruned with LD > 0.75.

Original label for this file:

TotalSNPs_Q20_mac3_maf0.05_minDP3_meanDP10_NA10.50_reduced_NA20.2_QUAL_maxDP30_NA30.95_SNP3_reduced_LD200_50_0.75_lmm_res_wuf2.Axis.1.assoc

Variables are:
- Rank: rank from 1to 20 within the top twenty candidate SNPs.
- remainder: see legend for file *3-TopSNP_HighCoverage* above.

File:'*S6-TopSNP_ MBiota_Ax2*'
The file contains data from the top 20 SNPs associated with Axis.2 of MDS-ordination of OTUs. Values for Axis.2 were LD-pruned with LD > 0.75.

Original label for this file:
TotalSNPs_Q20_mac3_maf0.05_minDP3_meanDP10_NA10.50_reduced_NA20.2_QUAL_maxDP30_NA30.95_SNP3_reduced_LD200_50_0.75_lmm_res_wuf2.Axis.2.assoc.txt 2

Variables are:
- Rank: rank from 1to 20 within the top twenty candidate SNPs.
- remainder: see legend for file *3-TopSNP_HighCoverage* above.

File:'*S10_Amplicon_Technical_Report*
This file contains the technical report for the sequencing of the bacterial metagenome.

File:'*S11_fastq2_Samples*'
This file contains a list of all samples from which the bacterial metagenome was typed and deposited with ENA (Accession PRJEB52013). We used universal primers for the V3/V4 region of 16S RNA. A key to sample numbering is given.

Rarefaction curves (examples)

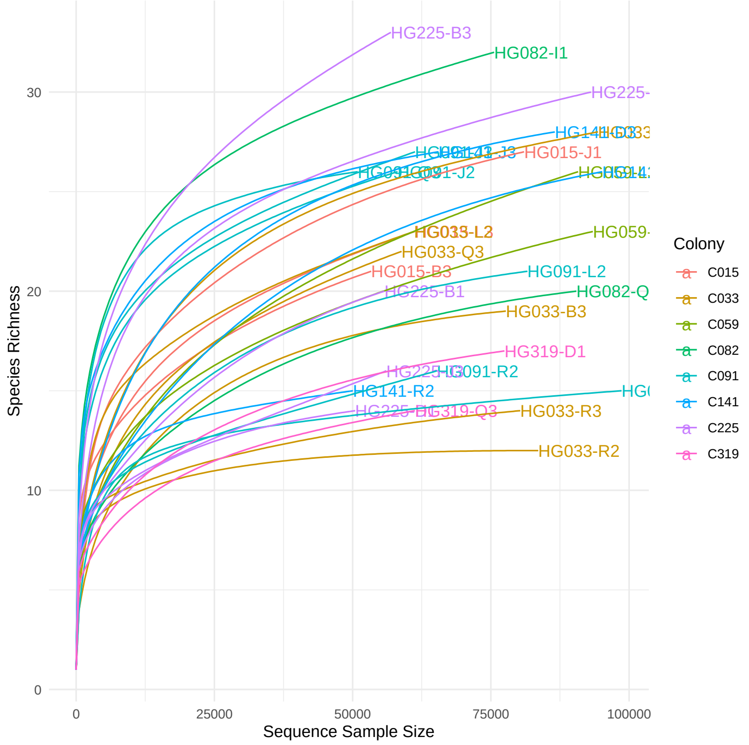

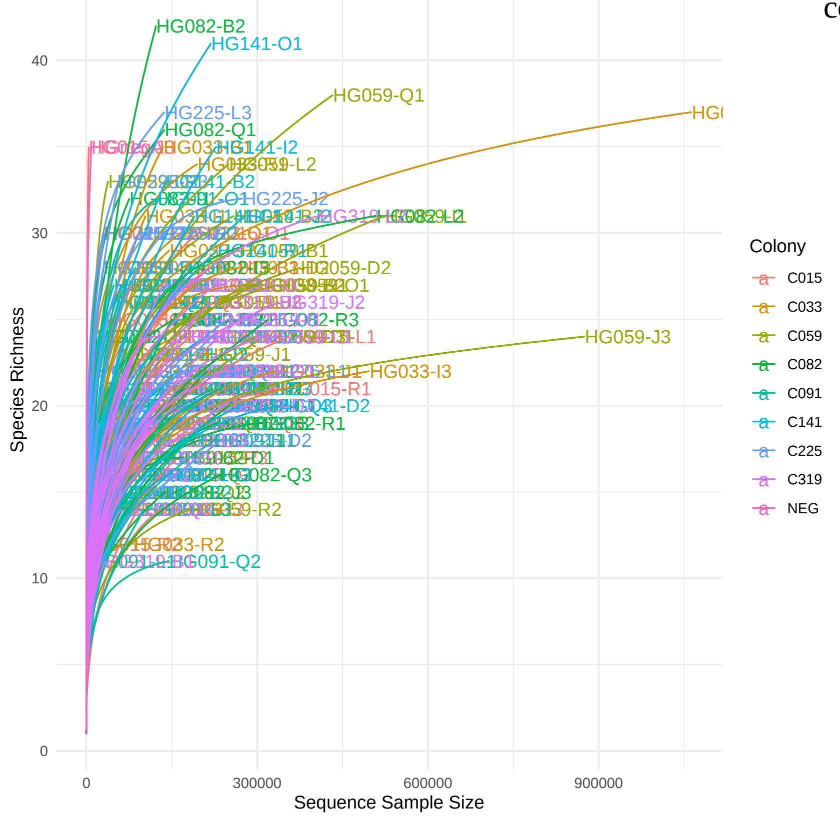

Fig C1. **Examples of rarefaction curves.** Shown are the number of zOTUs (y-axis) as a function of the number of reads considered (x-axis).
(a) Samples where rarefaction converges to a plateau with low numbers of reads. **(b)** Samples where rarefaction acts slowly, i.e. plateau is approached with high number of reads, or where satuartion seems far off (e.g. samples HG082-B2, HG141-01). First number after 'HG' refers to colony, second label (after dash) refers to strain code (see Table S2) and number of the replicate in this combination (1, 2, 3). Colonies are also colour-coded.

(a)

(b)

(c)

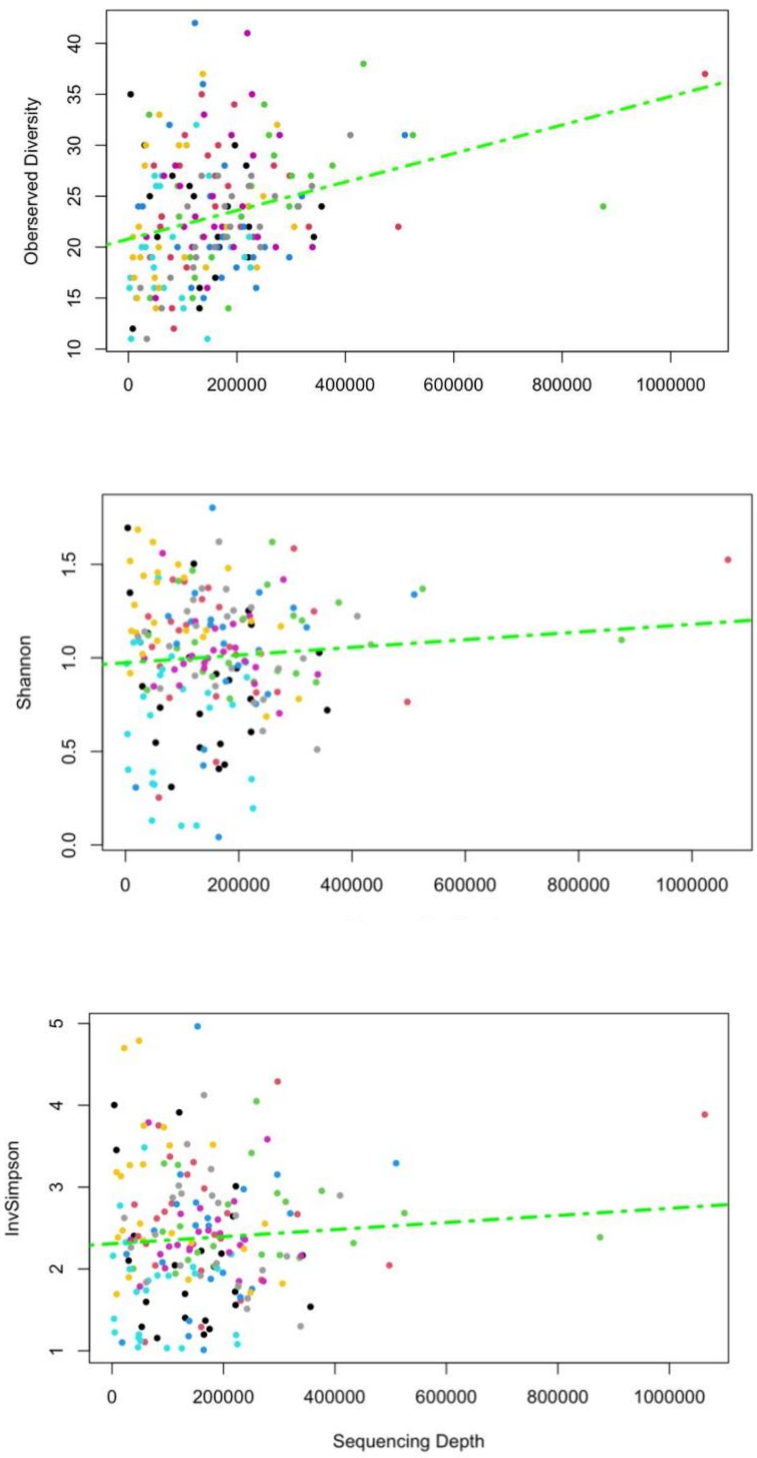

Fig C2. Estimates of zOTU diversity as a function of sequencing depth per sample (number of reads). Shown is the overall relationship across all colonies. (**a)** Observed Diversity (species richness). An ANOVA with depth ($F_{1,140}$ = 18.11, $p < 0.0001$), colony, strain, mean quantity, infection date, and wing size, showed an effect for sequencing depth only. **(b)** Shannon-Index. Only factor colony had an effect ($F_{7,140}$ = 7.27, $p < 0.0001$; see also Figure C3). **(c)** Inverse Simpson-Index. Only factor colony had an effect ($F_{7,140}$ = 4.60, $p <$ 0.001). Colours refer to colony. The green dotted line is the fitted regression line

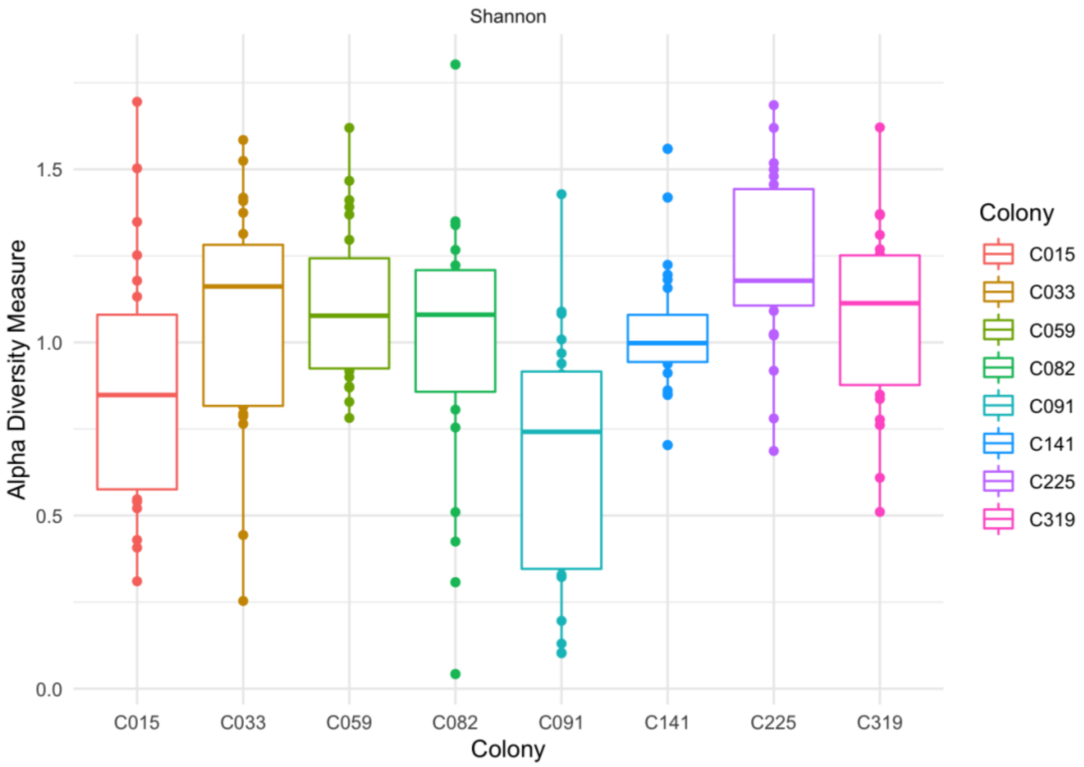

Fig C3. Boxplot for the Shannon-index (*H*) of zOTU diversity. *H* varies with colony ($F_{7,140} = 7.27$, $p < 0.0001$). Legend on right.

(a) Chao1-index

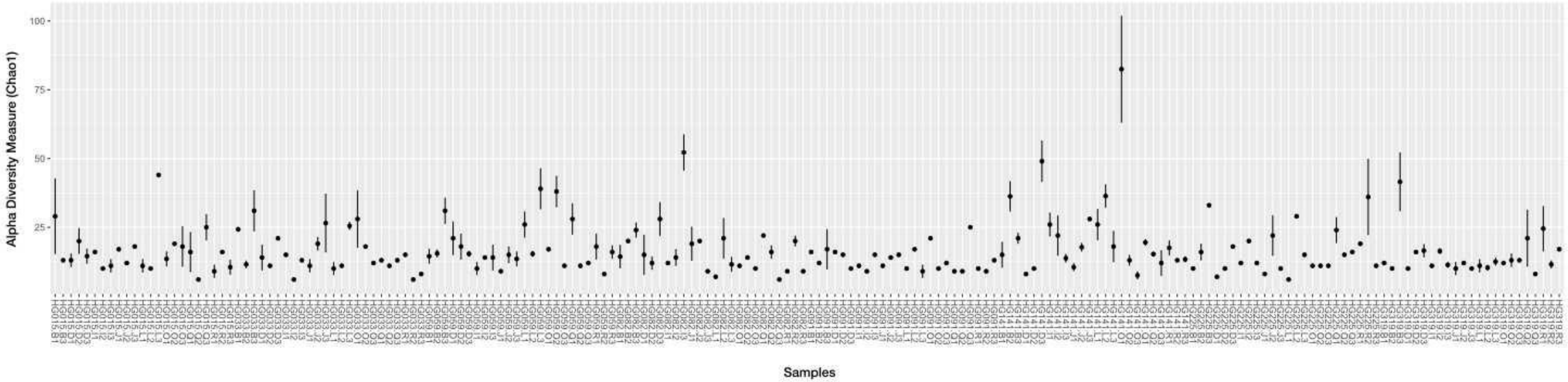

(b) Shannon-Wiener-index

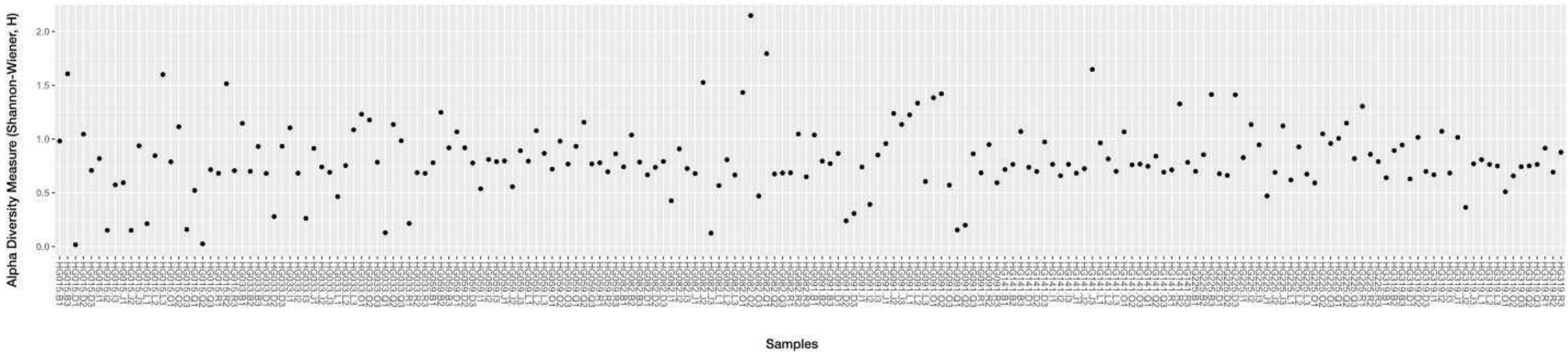

Fig C4. zOTU-diversity across all samples (x-axis). Shown are the estimates for (***a***) the Chao1 index, and (***b***) Shannon-Wiener.

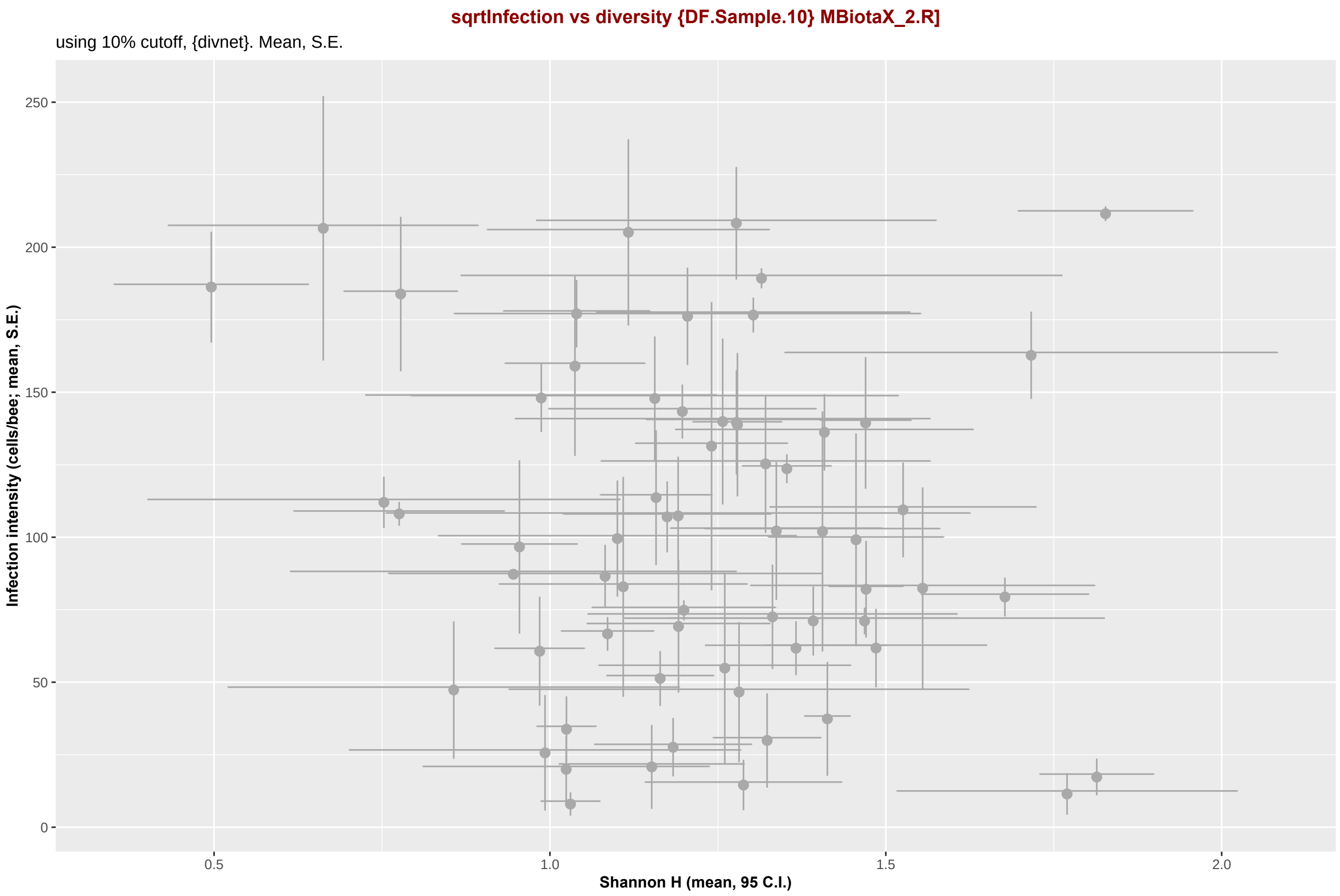

Fig C5. zOTU-diversity vs infection intensity. *x-Axis*: Shannon diversity (*H*) across samples. *y-Axis:* Infection intensity (mean, S.E.) per bee (square-root transformed values). Each dot refers to a Colony x Strain combination in the 8 x8 matrix (yielding 64 cases). The three replicates per combination were used to estimate mean (S.E.) for infection intensity per bee, and mean (S.E.) for *H* with the R-package '*divNet*'. Only zOTUs with reads more than 10 % of the median read number (10 % cut-off) were included (r = -0.141; t = 1.121, df = 63, p = 0.27).

Appendix D:

Microbiota data (8 x 8 matrix) separated by groups

Data files for all data:

File: '*S7-TopSNP_MBiota_Negative*' (.xlsx) File from: 6 May 2021
The file contains data from the top 20 SNPs associated with cases where Axis.1 < 0 ('permissive' group, from MDS-ordination of zOTUs. Values for Axis.1 corrected for structure, sqrt-transformed, and LD-pruned.

Original label for this file:
TotalSNPs_Q20_mac3_maf0.05_minDP3_meanDP10_NA10.50_reduced_NA20.2_QUAL_maxDP30_NA30.95_SNP3_group_negative_LD200_50_0.5_lmm_sqrt_res.asso

Variables: see legend for file *3-TopSNP_HighCoverage* above.

File: '*S8-TopSNP_ Mbiota_Positive*' (.xlsx) File from: 6 May 2021
The file contains data from the top 20 SNPs associated with cases where Axis.1 ≥ 0 ('resistant group'), from MDS-ordination of zOTUs. Values for Axis.1 corrected for structure, sqrt-transformed, and LD-pruned.

Original label for this file:
TotalSNPs_Q20_mac3_maf0.05_minDP3_meanDP10_NA10.50_reduced_NA20.2_QUAL_maxDP30_NA30.95_SNP3_group_positive_LD200_50_0.5_lmm_sqrt_res.assoc

Variables: see legend for file *3-TopSNP_HighCoverage* above.

File: '*S9-16S_ZOTU*' (.fa) File from: 22 Jan 2021
The file contains the nucleotide sequences of all identified zOTUs in the project. Fasta-format.

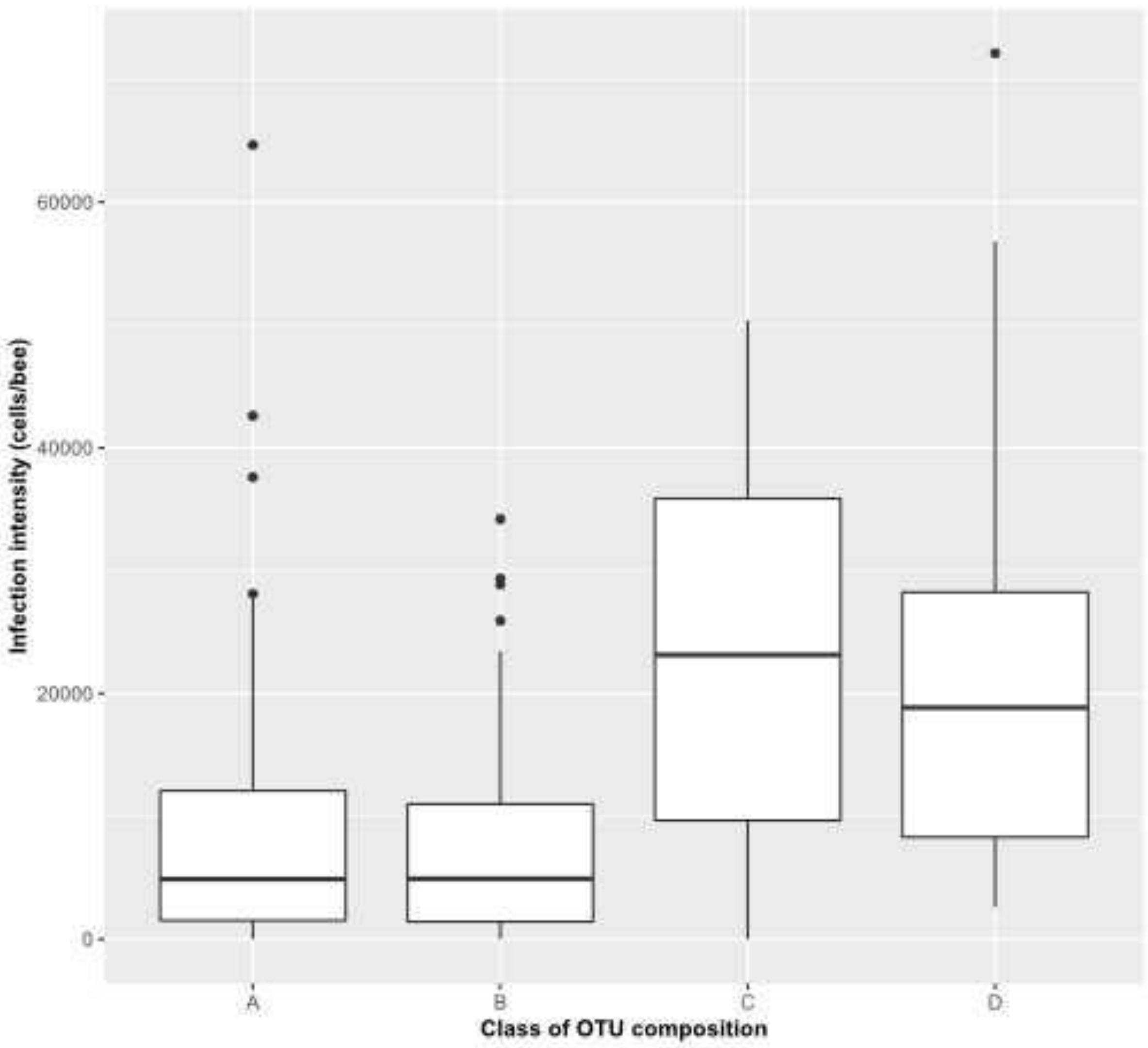

Fig D1. Infection intensity with respect to zOTU composition group. Samples were separated by multi-dimensional scaling (see Suppl Info, Figure C3), and grouped by the value of their first component (Axis.1), combined with the value of the second component (Axis.2). groups were A (Axis.1 ≥ 0, Axis.2 ≥ 0), B (Axis.1 ≥ 0, Axis.2 < 0), C (Axis.1 < 0, Axis.2 ≥ 0), and D (Axis.1 < 0, Axis.2 < 0). There was an overall significant effect ($F_{3, 179} = 6.12$, $p << 0.0001$), but primarily caused by the value of Axis.1 (groups A, B vs. groups C, D). Distances were based on weigthed Unifrac (*wuf*).

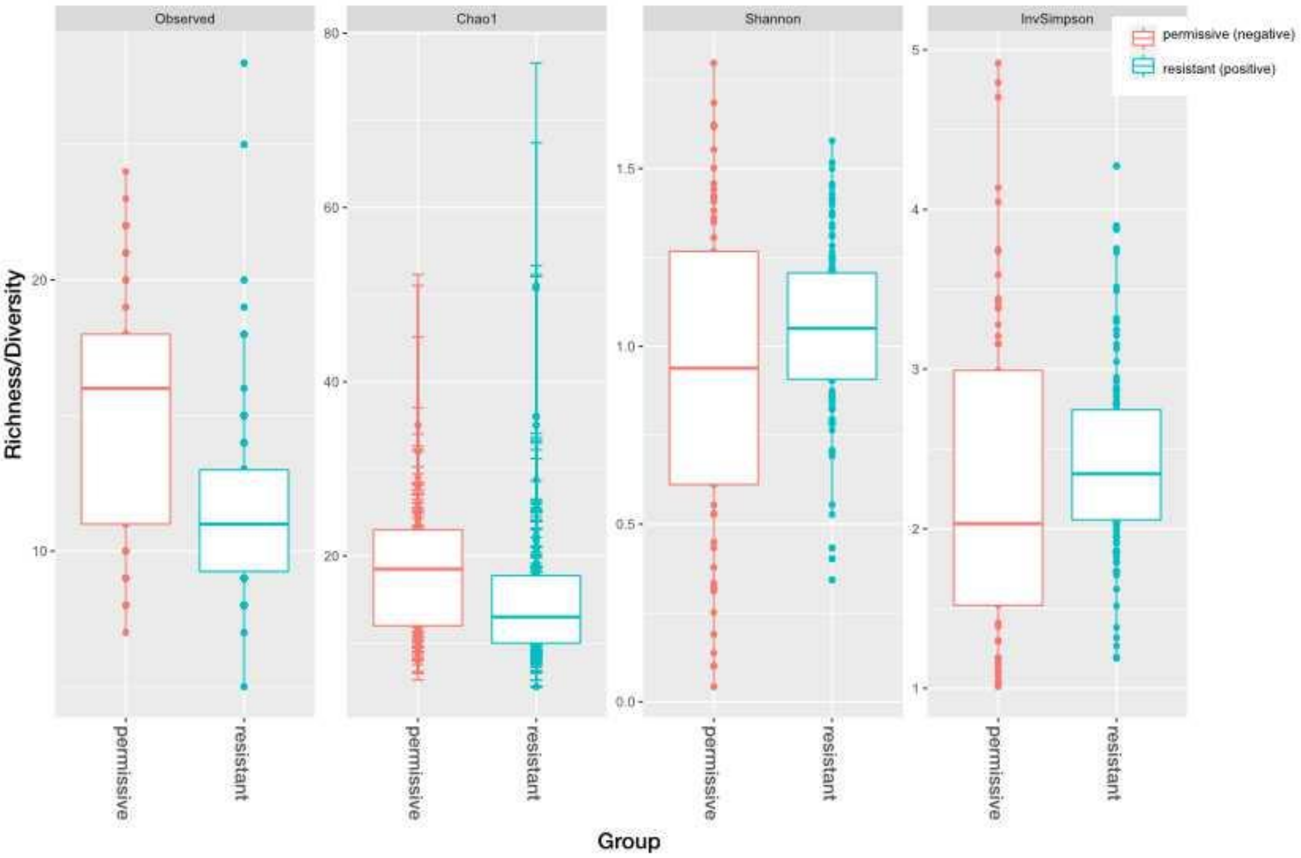

Fig D2. OTU diversity. Shown are the values for 'resistant' (positive, Axis.1 > 0) and 'permissive' (negative, Axis.1 < 0) groups (see text, Fig 4), using different measures. With 'Colony' a random effect within 'Group', the statistics for the difference between groups are - Observed (species richness): $F_{1,167}$ = 26.362, P << 0.001; Chao1: $F_{1,167}$ = 9.635, P =0.002; Shannon: $F_{1,167}$ = 7.871, P = 0.0057; Inverse Simpson: $F_{1,167}$ = 1.544, P =0.21.

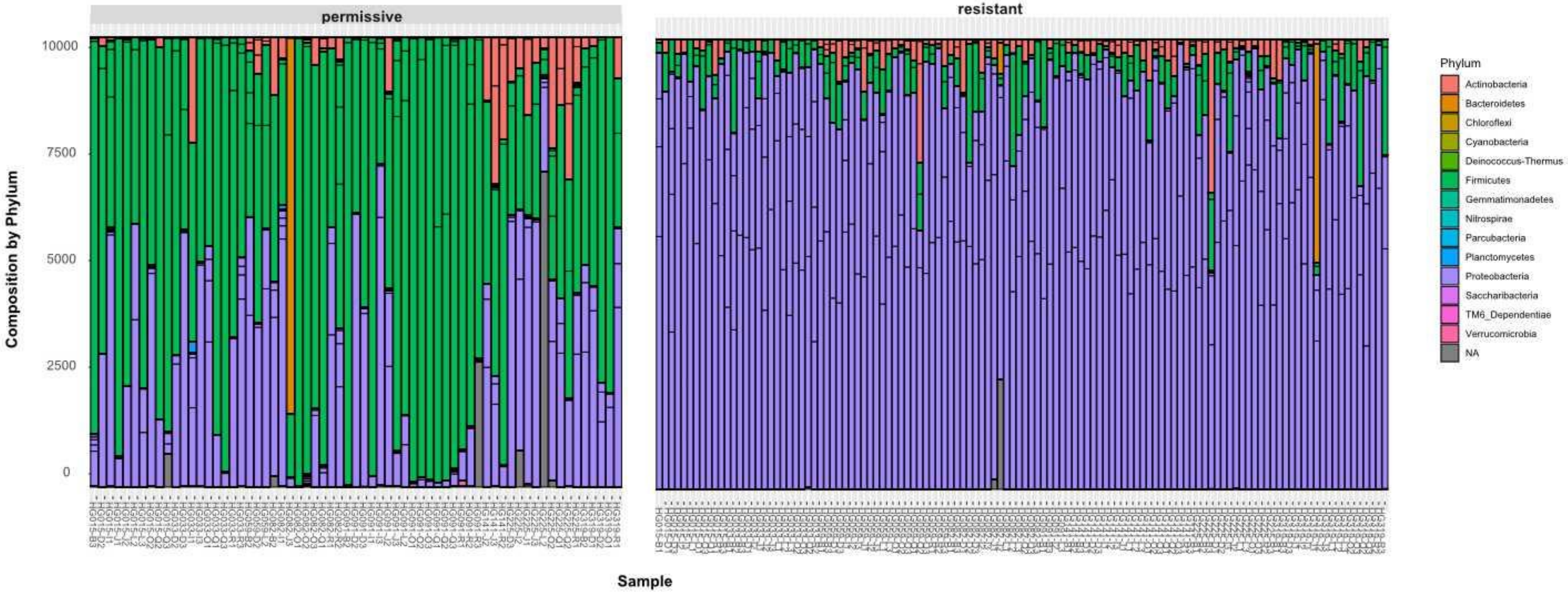

Fig D3. OTU-composition at the systematic level of Phylum. Shown are the samples in the 'permissive' (negative; left panel) and the 'resistant' (positive; right panel) group (see text). Distances based on weighted unifrac (*wuf*).

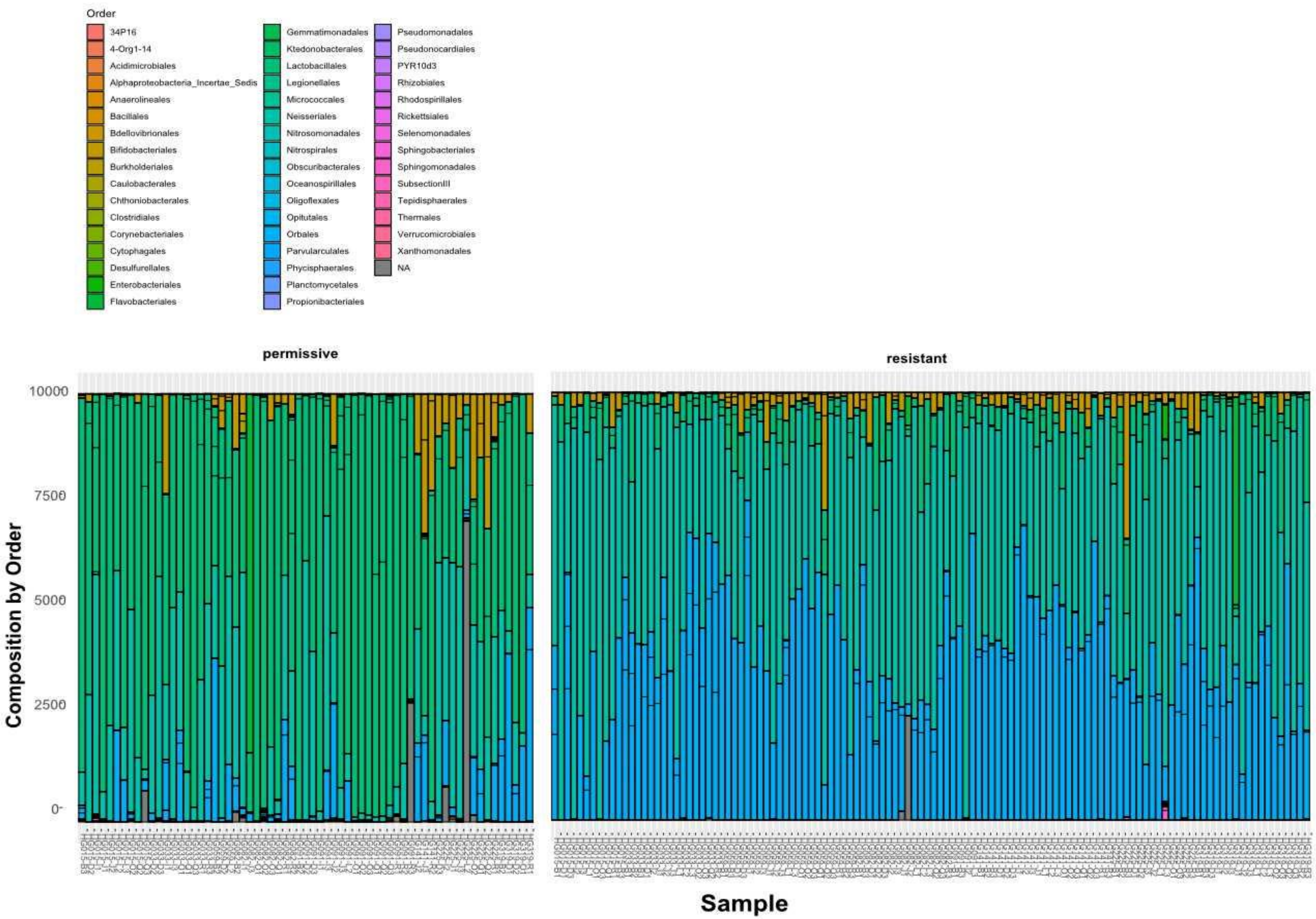

Fig D4. OTU-composition at the systematic level of Order. Shown are the samples in the 'permissive' (negative; left panel) and the 'resistant' (positive; right panel) group (see text). Distances based on weighted unifrac (*wuf*).

Appendix E:

The statistics of the interaction matrix

Table E1. **Varying the interaction matrix.** Shown is the effect of selecting a sub-matrix (envelope size) on the statistical values in an ANOVA . Note that these numbers refer to a particular random picks of Colony x Strain combinations, with 1,000 picks analysed for each envelope (except $k = 20$).

Envelope k[1]	n[2]	Statistical effect (df1, df2) range of *F*, range of *P*		
		Colony	Strain	Interaction
20 x 20	1	--	--	--
18 x 18	190	(17,556) *F*: 11.01, 18.88; *P*: <<0.0001	(17,556) *F*: 13.12, 23.86 *P*: <<0.0001	(286,556) *F*: 0.076, 10.849 *P*: N.S.
16 x 16	4,845	(15,454) *F*: 8.37, 19.74; *P*: <<0.0001	(15,454) *F*: 7.98, 26.35 *P*: << 0.0001	(224,454) *F*: 0.81, 1.25 *P*: 0.026, 0.96
14 x 14	38,760	(13,336) *F*: 5.52, 22.01; *P*: <<0.0001	(13,336) *F*: 5.89, 26.43 *P*: << 0.0001	(166,336) *F*:0.76, 1.35 *P*: 0.01, 0.98
12 x 12	125,970	(11,240) *F*: 4.39, 23.31; *P*: <<0.0001	(11,240) *F*: 3.49, 26.35 *P*: << 0.0001, 0.001	(119,240) *F*: 0.69,1.51 *P*: 0.003, 0.99
10 x 10	184,756	(9,166) *F*: 3.08, 20.61 *P*: <<0.0001, 0.001	(9,166) *F*: 2.02, 28.08 *P*: << 0.0001, 0.039	(80,166) *F*: 0.55, 1.69 *P*: 0.002, 0.99
8 x 8	125,970	(7,115) *F*: 1.74, 25.23; *P*: <<0.0001,0.11	(7,115) *F*: 0.85, 29.95 *P*: << 0.0001, 0.55	(49,115) *F*: 0.51, 1.94 *P*: 0.02, 0.99
6 x 6	38,760	(5,67) *F*: 0.205, 22.85; *P*: <<0.0001	(5,67) *F*: 0.40, 41.51 *P*: << 0.0001, 0.84	(25,67) *F*: 0.33, 2.59 *P*: 0.01, 0.99
4 x 4	4,845	(3,32) *F*: 0.091, 30.00; *P*: <<0.0001, 0.96	(3,32) *F*: 0.03,45.90 *P*: << 0.0001, 0.99	(9,32) *F*: 0.101, 5.61 *P*: << 0.0001, 0.99

[1] k indicates the size of the sub-matrix (k x k).

[2] n is the number of possible permutations $\binom{N}{k}$ for either Colony ($N = 20$) or Strain ($N = 20$).
